# Multiplex Cytological Profiling Assay to Measure Diverse Cellular States

**DOI:** 10.1371/journal.pone.0080999

**Published:** 2013-12-02

**Authors:** Sigrun M. Gustafsdottir, Vebjorn Ljosa, Katherine L. Sokolnicki, J. Anthony Wilson, Deepika Walpita, Melissa M. Kemp, Kathleen Petri Seiler, Hyman A. Carrel, Todd R. Golub, Stuart L. Schreiber, Paul A. Clemons, Anne E. Carpenter, Alykhan F. Shamji

**Affiliations:** Broad Institute of Harvard and MIT, Cambridge, Massachusetts, United States of America; Baylor College of Medicine, United States of America

## Abstract

Computational methods for image-based profiling are under active development, but their success hinges on assays that can capture a wide range of phenotypes. We have developed a multiplex cytological profiling assay that “paints the cell” with as many fluorescent markers as possible without compromising our ability to extract rich, quantitative profiles in high throughput. The assay detects seven major cellular components. In a pilot screen of bioactive compounds, the assay detected a range of cellular phenotypes and it clustered compounds with similar  annotated protein targets or chemical structure based on cytological profiles. The results demonstrate that the assay captures subtle patterns in the combination of morphological labels, thereby detecting the effects of chemical compounds even though their targets are not stained directly. This image-based assay provides an unbiased approach to characterize compound- and disease-associated cell states to support future probe discovery.

## Introduction

Gene-expression profiling, the most established unbiased profiling method, has been used to support small-molecule discovery in number of ways. For example, gene expression has been used to define disease states, such as those caused by genomic alterations in cancer, thereby enabling identification of compounds that reverse the cellular phenotype to a preferable state [1]. Gene expression has also been used to infer compound mechanism of action by revealing that previously unconnected compounds yield similar profiles in cells, or by revealing that sets of genes enriched for those having specific functions are regulated in a concerted manner [2,3]. Microscopy images of cells are increasingly being used for profiling [4,5] because they contain a large amount of quantitative information about a wide range of complex phenotypes, and because image-based assays can be scaled to medium and high throughput with relative ease. It has for some time been possible to measure hundreds of properties of individual cells in microscopy images [6] and to find nonlinear combinations of features that can identify complex phenotypes [7]. Computational methods for image-based profiling are under active development [8-13], but have largely been applied to assays that model particular phenotypes of interest with minimal numbers of labels. Applying these methods in a more unbiased manner to, for example, discover new phenotypes of interest, requires development of an assay that can capture a much wider range of phenotypes.

## Results

**Figure 1:**
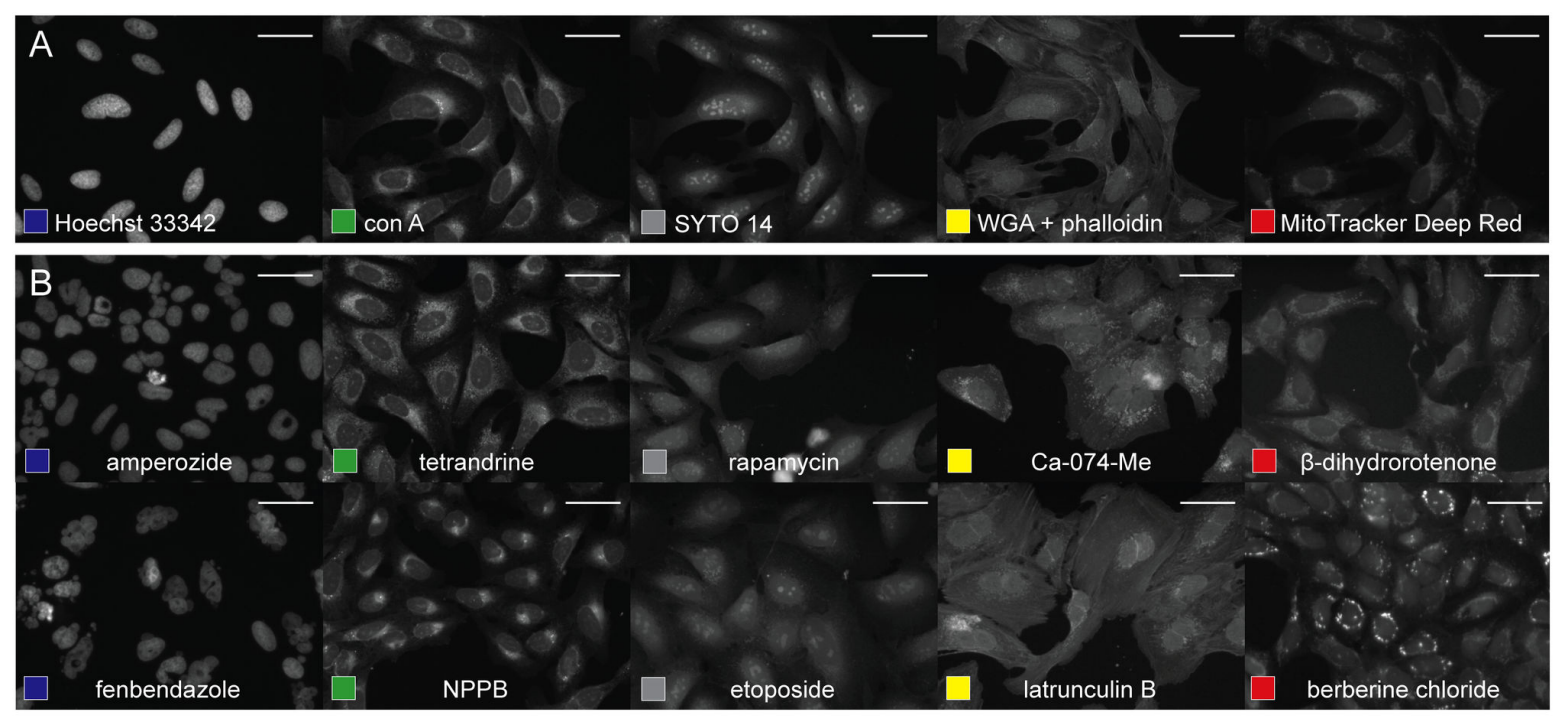
The cell-painting assay applied to U2OS cells. (A) Cells labeled with Hoechst 33342 (nuclei, blue), concanavalin A (ER), SYTO 14 (nucleoli), phalloidin (actin), WGA (Golgi), MitoTracker Deep Red (mitochondria). Scale bars 50 µm. (B) Ten diverse phenotypes in compound-treated U2OS cells: toroid nuclei (amperozide); giant, multinucleated cells (fenbendazole); abundant ER (tetrandrine); redistribution of ER to one side of nucleus (NPPB); reduced nucleolar size (rapamycin); large, flat nucleoli (etoposide); bright, abundant Golgi staining (Ca-074-Me); actin breaks (latrunculin B); extensive mitochondrial fission (Beta-dihydrorotenone); and redistribution of mitochondria (berberine chloride). Scale bars 50 μm.

**Figure 2:**
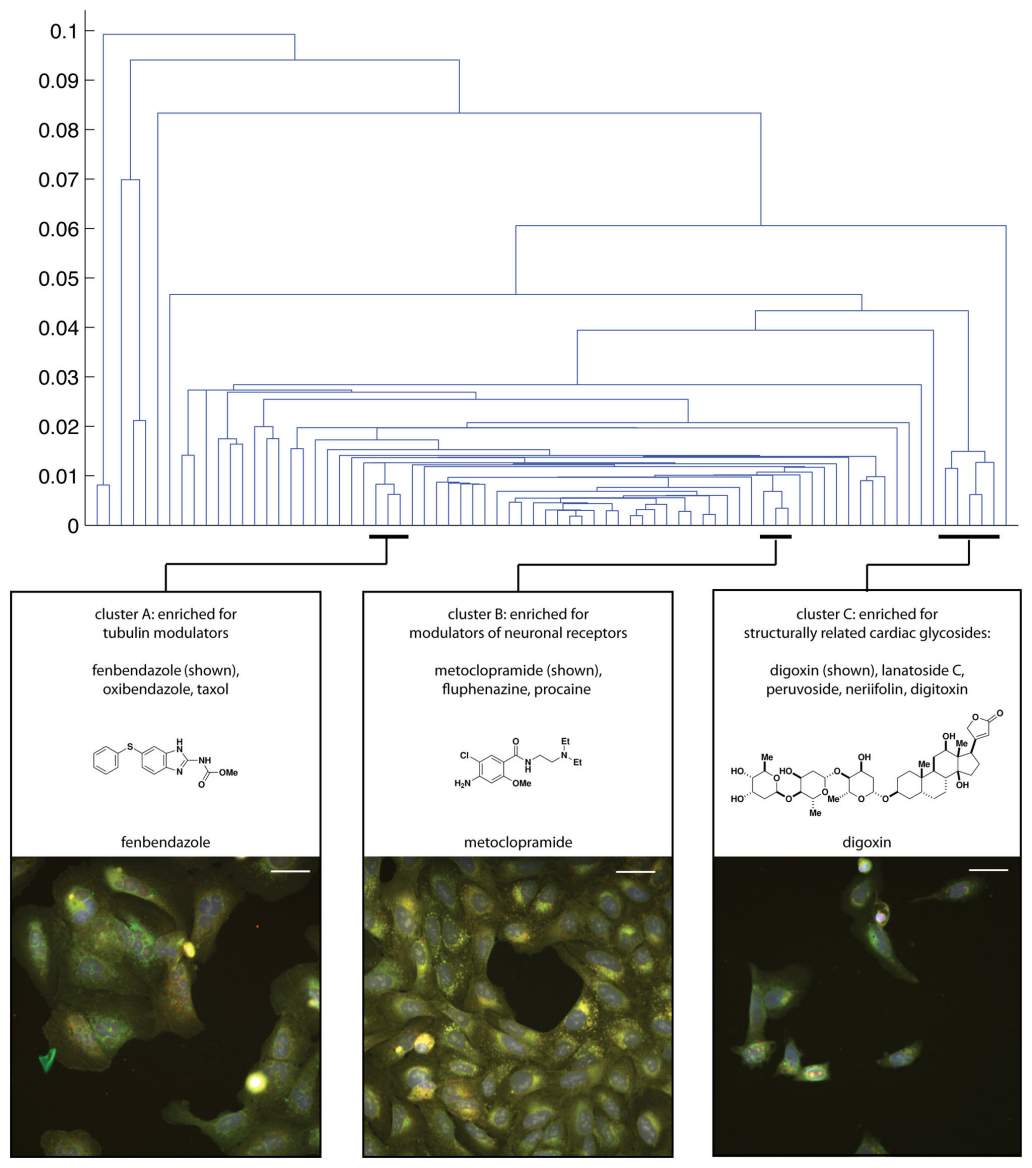
Hierarchical clustering of image-based profiles. Details are shown for three of the clusters that were highly enriched for annotation terms. These enriched clusters contain compounds with similar mechanisms of action, some with similar and some with distinct chemical structure. The presence of these enriched clusters indicates that the assay can identify subtle, physiologically relevant effects of compounds on cultured cells. U2OS cells labeled for nuclei (blue), ER (green), nucleoli (grey), actin and Golgi (yellow), and mitochondria (red). Scale bars 50 µm.

We sought to develop an assay that “paints the cell” with as many fluorescent morphological labels as possible without compromising our ability to extract quantitative image-based profiles in high throughput. We present a multiplex cytological profiling assay that allows detection of seven major cell components (Figure **1A**), and we demonstrate its ability to capture a wide range of cellular phenotypes induced by small molecules (Figure **1B**). Further, we demonstrate the ability of the profiling data to connect compounds with similar mechanisms of action (Figure **2**). Because the profiles capture subtle patterns in the combination of morphological labels, the assay can detect the effects of chemical compounds even though their targets are not stained directly.

We considered only well-characterized, fluorescent, non-antibody dyes suitable for high-throughput application. We first screened a number of potential dyes for those with high signal, low background, assay buffer compatibility, fixation and permeabilization condition compatibility, staining time, and optical spectra. To ensure compatibility with commonly available microscopes, we limited the protocol to detecting stains in five channels. Within that constraint, we increased the degree of multiplexing by including two dyes for a given optimal spectrum if they stained spatially distinct cellular components that could be distinguished during analysis. The staining protocol was optimized largely based on qualitative assessment of cellular features of interest. Particular attention was paid to the relative concentration of WGA and phalloidin to allow visualization of the Golgi apparatus, but not at the expense of detection of actin filaments. Pilot plates were assayed with varying concentrations of WGA and phalloidin. Images were examined by eye to select the optimal concentrations.

 The final protocol involves imaging five channels to detect seven cell components using six stains (Table **1**, Figure **1A**), which were significantly optimized for dye concentration, buffer composition, staining time, and permeabilization, blocking, and washing conditions. The protocol is readily transferable to multiple adherent cell lines (Figure **S1**). 

**Table 1:** Multiplex cytological profiling assay components.

**Cellular component(s)**	**Stain**	**Detection (ex/em)**
**nucleus**	Hoechst 33342	387/447 nm
**endoplasmic reticulum**	concanavalin A (con A) AlexaFluor488 conjugate	472/520 nm
**nucleoli**	SYTO 14 green fluorescent nucleic acid stain	531/593 nm
**Golgi apparatus and plasma membrane**	wheat germ agglutinin (WGA) AlexaFluor594 conjugate	562/642 nm
**F-actin**	phalloidin AlexaFluor594 conjugate	562/642 nm
**mitochondria**	MitoTracker Deep Red	628/692 nm

We validated the assay by profiling 1600 commercially available bioactive compounds (Table **S1**) spanning a range of mechanisms of action. Briefly, U2OS cells were plated in quadruplicate in 384-well plates, incubated for 24 h to allow cells to adhere and resume growth, and then treated with compounds for 48 h (typical concentration 10 µM). Following the multiplex cytological profiling protocol, images were captured at 20x magnification with an automated epifluorescent microscope. We extracted 824 morphological features (Table **S2**) from each cell using the open-source software CellProfiler [6]. A number of cellular phenotypes could be detected by eye (Figure **1B**). The profiles of the 64 mock-treated wells on each plate vary little over the course of the experiment (Figure **S2**, Table **S3**), although some positional effects are evident (Figure **S3**, Table **S4**). Roughly half of the features showed significant response to one or more compounds (Figure **S4**). The group of features that were the least useful for this assay were the Zernike shape features (Table **S5**).

To determine whether image-based profiles derived from the multiplex assay are useful for studying compound mechanism-of-action, we examined whether clustering compounds according to image-based profile similarity would group compounds with similar annotated protein targets or chemical structure. After clustering hierarchically the 75 active compounds for which we had annotations and ranking the clusters' enrichment of annotation terms, we found that several of the most enriched clusters were convincing mechanistic groups (Figure **2**). For example, cluster A contains both structurally related and distinct modulators of tubulin (fenbendazole; oxibendazole; taxol), which lead to large multinucleated cells with fused nucleoli. The promotion of polyploidization and multinucleation by tubulin modulators has been long recognized [14,15]. Cluster B contains modulators of neuronal receptors, all of which lead to enhanced Golgi staining and some cells with fused nucleoli: fluphenazine (D1 and D2 dopamine receptor antagonist), metoclopramide (D2 dopamine antagonist; muscarinic M1 receptor antagonist; 5-hydroxytramine 4 receptor agonist), as well as procaine (sodium channel antagonist), a structural analog of metaclopramide (DrugBank [16] acc. DB01233). It is worth noting that all three compounds contain a basic tertiary amine, which has been linked to compound accumulation in acidic cellular compartments, such as the lysosome and Golgi, with effects on their shape and function [17]. It is possible that this chemical feature and cellular mechanism underlie the shared effect of these compounds on morphology rather than channel inhibition. Cluster C contains a number of structurally related cardenolide glycosides (digoxin; lanatoside C; peruvoside; neriifolin; digitoxin), characterized by reduced cell size, condensed nuclei, plasma membrane blebbing, reduced nucleolar staining, and significant cytotoxicity (Text **S2**). While compounds of this class are thought to affect a range of biological processes, their effects on morphology are consistent with their reported ability to cause cell death [18,19].

## Discussion

A rich multiplex assay, such as our cell-painting assay, is a necessary step towards productively profiling a large collection of small molecules. Profiles from such an experiment could be mined to identify regulators of dozens of different phenotypes without having to design and optimize specific assays for each phenotype. Rather, a large, unbiased profiling experiment could be performed once and then efficiently and inexpensively mined for multiple patterns, including unexpected patterns associated with a perturbation of interest. The rich patterns in the profiles could also be used to group small molecules based on their similarity to generate hypotheses about which small molecules share a common mechanisms of action.

Cellular morphology is affected by a number of factors, such as the genetic and epigenetic state of the cell, physiologic processes such as cell division or metabolism, and changes in environmental cues that alter cell signaling. Extensive measurement of morphological features, treated as a profile, can be applied to study the response of cells to diverse perturbations or to characterize the differences between cells from disease and non-disease states. The multiplex assay described here increases the number of morphological features that can be quantified by microscopy and image analysis to create image-based profiles. We anticipate the assay will be useful for characterizing perturbations whose effects are poorly understood, such as novel small molecules or disease-associated variants emerging in genome-wide association studies. We provide the complete set of images from our experiment as well as source code for computer programs that reproduce our results (Text **S1**). 

## Materials and Methods

### Plating

U2OS cells (#HTB-96, ATCC) were plated at the density of 1500–2000 cells per well in 384-well imager quality black/clear plates (Aurora Biotechnologies/Nexus Biosystems) in 50 µL DMEM supplemented with 10% fetal bovine serum, and 1% penicillin/streptomycin. Cells were grown for 24 h at 37°C.

### Compound Pinning

Compounds were pin-transferred to cells using a CyBi-Well robot (CyBio, Inc.). Cells were treated for 48 h at 37°C. 

### Staining

The samples were stained as follows.

#### Step 1: MitoTracker and Wheat Germ Agglutinin staining.

MitoTracker Deep Red (#M22426, Invitrogen) was dissolved in DMSO to 1 mM. Wheat Germ Agglutinin (WGA) Alexa594 conjugate (#W11262, Invitrogen) was dissolved in dH_2_O to 1 mg/mL. A 500 nM MitoTracker, 60 µg/mL WGA solution was prepared in prewarmed media (DMEM, 10% FBS, 1% penicillin/streptomycin). Media was removed from plates; residual volume was 10 µL in each well. 30 µL of staining solution was added to wells and incubated for 30 min at 37 °C. 

#### Step 2: Fixation.

10 µL of 16% methanol-free paraformaldehyde (#15710-S, Electron Microscopy Services) was added to wells for a final concentration of 3.2%. The plates were then incubated at room temperature for 20 min. Wells were washed once with 70 µL 1xHBSS (#14065-056, Invitrogen). 

#### Step 3: Permeabilization.

A 0.1% solution of Triton X-100 (T8787-100mL, Sigma) was prepared in 1x HBSS. 30 µL of the solution was added to the wells and incubated for 10–20 min. Wells were washed twice with 70 µL 1x HBSS. 

#### Step 4: Phalloidin, ConcanavalinA, Hoechst, and SYTO 14 staining.

Concanavalin A Alexa488 conjugate (#C11252, Invitrogen) was dissolved to 1 mg/mL in 0.1 M sodium bicarbonate (SH30033.01, HyClone), and Phalloidin Alexa594 conjugate (#A12381, Invitrogen) was dissolved in 1.5 mL methanol (67-56-1, BDH) per vial. A 0.025 µL phalloidin/µL solution, 100 µg/mL ConcanavalinA, 5 µg/mL Hoechst33342 (#H3570, Invitrogen), and 3 µM SYTO14 green fluorescent nucleic acid stain (#S7576, Invitrogen) solution was prepared in 1x HBSS, 1% BSA. 30 µL of staining solution was added to wells and incubated for 30 min. Wells were washed three times with 70 µL 1xHBSS, no final aspiration. Plates were sealed with blue Remp thermal seal, at 171 °C for 4 s. 

### Imaging

Images were captured at 20x magnification in 5 fluorescent channels, DAPI (387/447 nm), GFP (472/520 nm), Cy3 (531/593 nm), TexasRed (562/642 nm), Cy5 (628/692 nm) on an ImageXpress Micro epifluorescent microscope (Molecular Devices), 9 sites per well, with laser based autofocus in the DAPI channel, first site of each well. 

### Image analysis

Version 2.0.9925 of the image-analysis software CellProfiler [6] was used to locate and segment the cells and measure many features of each cell (Table **S2**) using the pipelines provided (Text **S1**). After correcting for uneven illumination, the pipeline identifies the nuclei from the DAPI channel and uses the nuclei as seeds to help a segmentation algorithm identify the cytoplasm[20,21]. The pipeline measure size, shape, texture, intensity statistics, and local density of the nuclei, cytoplasms, and entire cells. 

### Annotation

We used annotations that have previously been collected and curated over the course of several projects. Many of the annotations have been deposited into ChemBank [22], but the annotation work has continued after ChemBank became static. The annotations we used are included as supplementary data.

The annotations covered 649 of the 1600 compounds in the experiment (Table **S6**). Some annotations were from the Gene Ontology [23] (including GOMF, GOBP, and GOCC). Others were medical subject headings (MeSH) or product use/class fields from the compounds’ material safety data sheets. There were also a small number of protein targets (Entrez GeneIDs) among the annotations. 

The annotation terms had been “slimmed,” replacing excessively detailed terms with more general terms that give a broader overview. The GO annotations were slimmed using GO slim [23], whereas MeSH and product use/class terms were slimmed by manual inspection. The protein targets were slimmed by assigning the appropriate GOMF, then applying GO slim.

### Finding term-enriched clusters

We identified clusters and scored them for enrichment for annotation terms as follows.

1 Computed a profile for each of the 7680 samples (20 plates with 384 wells per plate) by averaging each CellProfiler-generated feature across the cells in the well. Averaging has been effective for profiling even though it does not explicitly model heterogeneity among cells [4,10]. The entire CellProfiler feature set was used for the analysis; while feature reduction techniques may result in incremental improvements in performance, we chose to transform the data as little as possible in order to focus the evaluation on the assay itself rather than advanced data-analysis methods. For the same reason, we also chose well-known and transparent methods for the subsequent steps of the analysis.
2 Aggregated the 7680 per-sample profiles into 1601 per-compound profiles by computing the element-wise median. The 1601 per-compound profiles include the median mock profile, i.e., the median profile of all DMSO-treated samples.
3 Excluded compounds that were inactive in the assay. Compounds were deemed to be active if their profiles’ Euclidean distance to the median mock profile was above a cutoff. The cutoff was the 95^th^ percentile of the distances from the mock-treated wells to the median mock profile. Of the 1600 compounds, 203 (13%) were active.
4 Excluded compounds that were unannotated. Of the 203 active compounds, 75 were annotated by one or more of 96 slimmed terms (**Table S7**).
5 Performed hierarchical clustering of the compound profiles of the 75 compounds that were active and annotated, using the cosine distance and single linkage.
6 Assessed whether each possible cluster is enriched by each annotation term (**Table S8**). There were 74 possible clusters, one for each non-leaf subtree of the dendrogram produced by the hierarchical clustering. The assessment was by permutation testing: we measured the fraction of random clusters of the same size that had at least the same number of compounds annotated with the term in question. When constructing random clusters for permutation testing, the cluster members were drawn from a uniform distribution over the compounds. It was not necessary to correct for multiple testing because the fractions were only used for ranking and not interpreted as p-values. Enrichment in GO terms has also recently been used to validate clusters of profiles generated from HTS experiments [24]. Table S8 shows the clusters ranked by permutation-testing score, i.e., the fraction of random clusters that had at least the same number of compounds annotated with the term in question. For each cluster, it shows the number of compounds in the cluster, the number of times the enriched term occurs in the cluster, and the number of times the enriched term occurs in the entire dataset. For each compound in the cluster, the table shows whether the compound has the enriched term, as well as the compound’s name and Broad ID (internal identifier from our compound-management department).

### Reproducibility

We provide (Text **S1**) the complete image set, the CellProfiler pipelines used to identify and measure the cells, the database of cellular features, and the source code for the programs that analyze the features and produce the figures and tables in this article.

## Supporting Information

Dataset S1 **CellProfiler pipelines.**
(ZIP)

Dataset S2 **CellProfiler illumination function.**
(ZIP)

Dataset S3 **Image features extracted by CellProfiler.**
(DOCX)

Dataset S4 **Source code to programs that analyze image features.**
(ZIP)

Figure S1 **The cell-painting protocol was developed on U2OS cells, but it is readily transferable to multiple adherent cell lines, viz.** **3T3 fibroblasts, A549 adenocarcinomic human alveolar basal epithelial cells, HTB-9 human bladder carcinoma cell, and MCF-7 breast cancer cells**.
Scale bars 50 µm.
(TIF)

Figure S2 **The plate-to-plate variability in the experiment is small** (<** 0.2**)** for the vast majority of features.**
The histogram shows the distribution of coefficients of variation (absolute value) across the features. Each coefficient was computed across 12 values of the relevant feature: the average across the mock-treated cells on each of the 12 plates in the experiment.
(PDF)

Figure S3 **The well-to-well variability in the experiment is small** (<** 0.2**)** for the vast majority of features.**
The histogram shows the distribution of coefficients of variation (absolute value) across the features. Each coefficient was computed across the 64 well positions in which mock-treated cells appear on each plate in the experiment.
(PDF)

Figure S4 **The magnitude of the compounds’ effects on the features.** The histogram shows the distribution of maximal values of the features across the 75 active compounds in the experiment, standardized by reference to the population of mock-treated cells on the same plate.
(PDF)

Table S1 **The 1600 bioactive compounds profiled using our assay.**
(DOCX)

Table S2 **Image features measured for each cell by CellProfiler (see the CellProfiler manual for descriptions of each feature).**
(DOC)

Table S3 **Features ranked by plate-to-plate coefficient of variation (absolute), limited to mock-treated cells.**
(DOCX)

Table S4 **Features ranked by well-to-well coefficient of variation (absolute), limited to mock-treated cells.**
(DOCX)

Table S5 **Features ranked by maximal value across the compounds.**
(DOCX)

Table S6 **Compounds that were annotated.**
(DOCX)

Table S7 **The compounds that were both active and annotated.**
(DOCX)

Table S8 **The clusters of compounds most highly enriched for annotation terms.**
(DOCX)

Text S1 Data and software.
(DOCX)

Text S2 Cytotoxicity.
(DOC)
