# Supplementary material for: Multiplex Cytological Profiling Assay to Measure Diverse Cellular States
Source: PLoS One. 2013 Dec 2;8(12):e80999. doi: 10.1371/journal.pone.0080999 (PMC3847047; doi:10.1371/journal.pone.0080999)
Supplement: Dataset S3 — Image features extracted by CellProfiler. (DOCX) [file pone.0080999.s003.docx]

Supplementary Data S3 for "Multiplex cytological profiling assay to measure diverse cellular states”,

http://www.broadinstitute.org/pubs/gustafsdottir_plosone_2013/
