# Supplementary figures and images for: Multiplex Cytological Profiling Assay to Measure Diverse Cellular States
Source: PLoS One. 2013 Dec 2;8(12):e80999. doi: 10.1371/journal.pone.0080999 (PMC3847047; doi:10.1371/journal.pone.0080999)

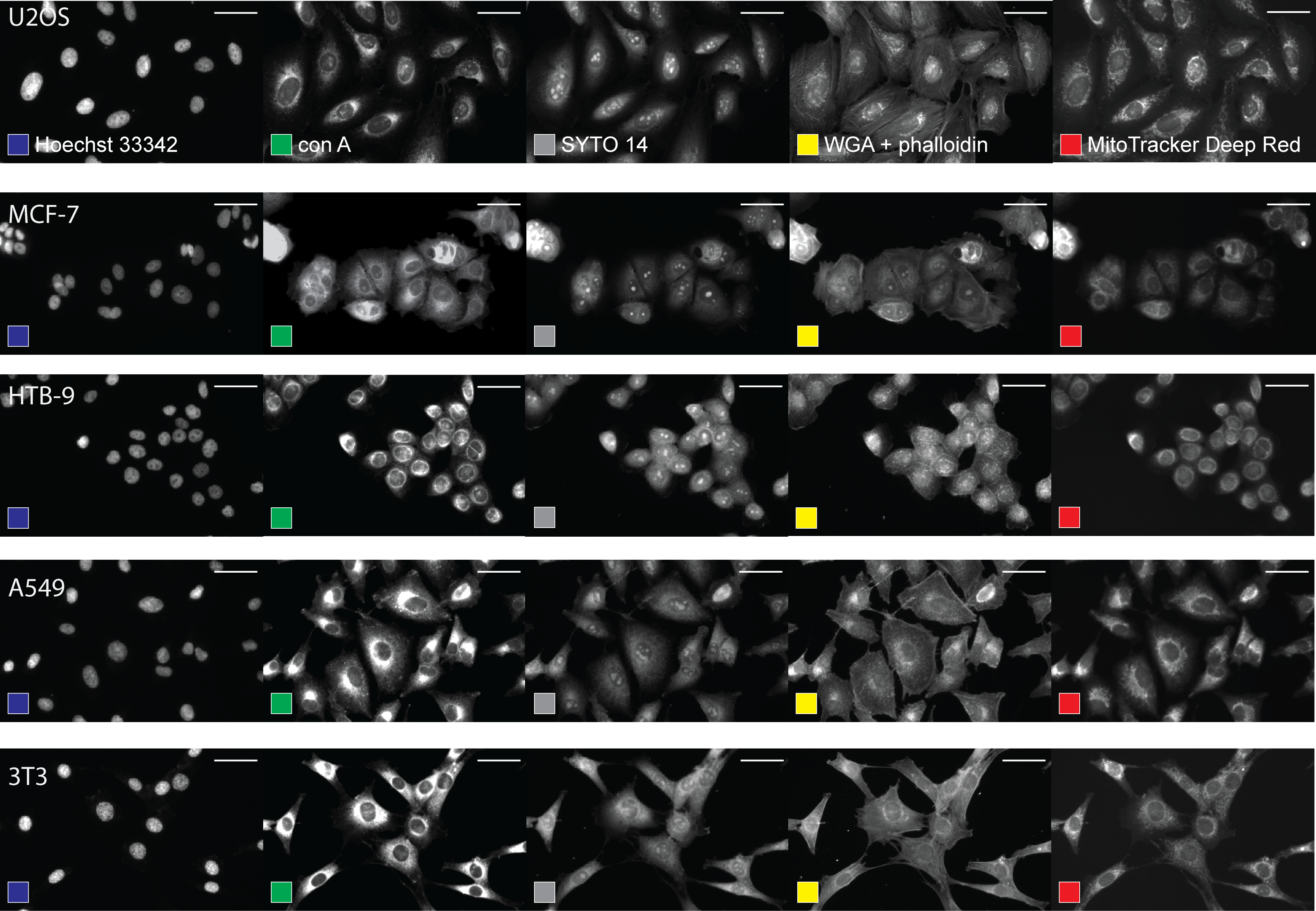

Supplement: Figure S1 — The cell-painting protocol was developed on U2OS cells, but it is readily transferable to multiple adherent cell lines, viz. 3T3 fibroblasts, A549 adenocarcinomic human alveolar basal epithelial cells, HTB-9 human bladder carcinoma cell, and MCF-7 breast cancer cells. Scale bars 50 µm. (TIF) [file pone.0080999.s005.tif]

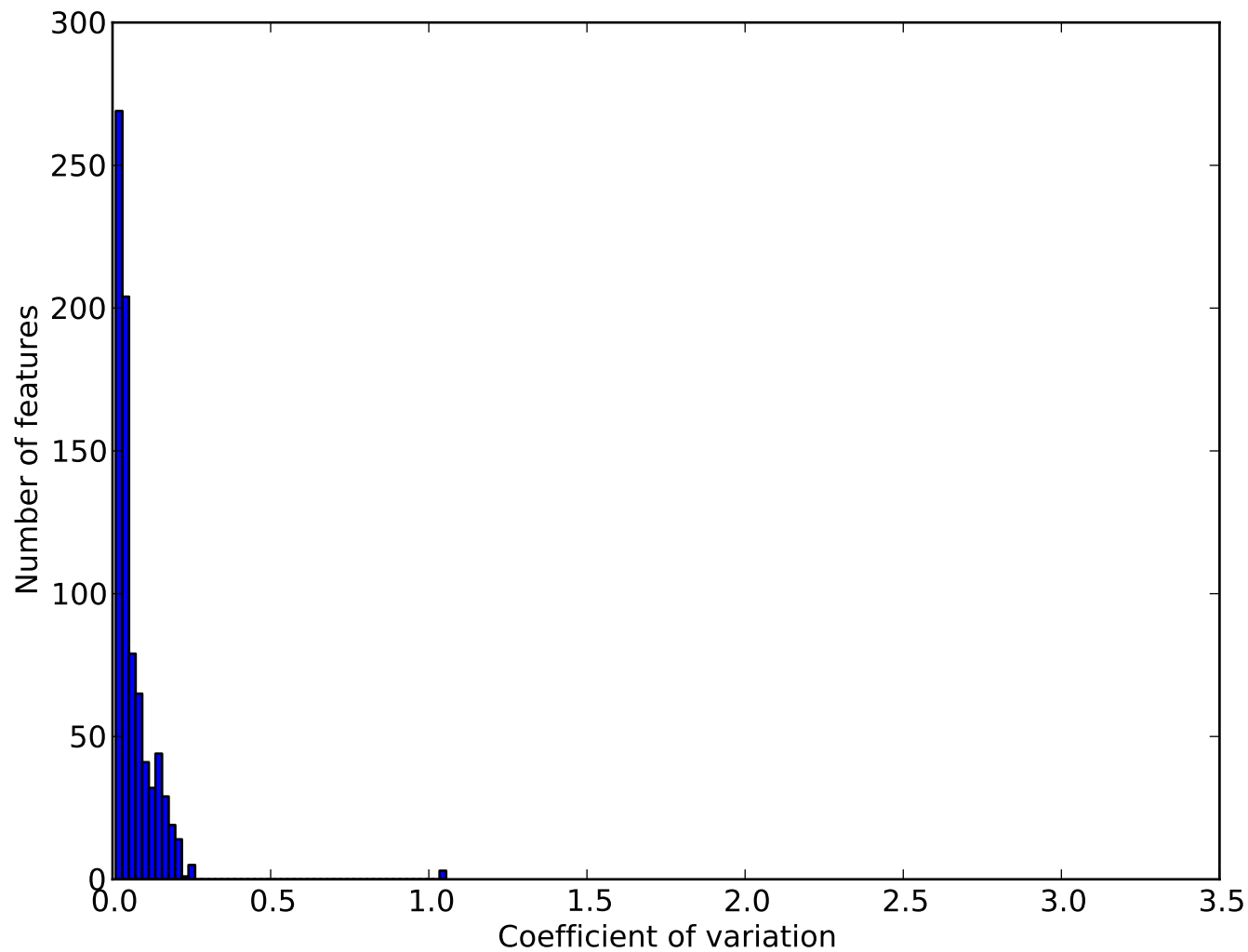

Supplement: Figure S2 — The plate-to-plate variability in the experiment is small (< 0.2) for the vast majority of features. The histogram shows the distribution of coefficients of variation (absolute value) across the features. Each coefficient was computed across 12 values of the relevant feature: the average across the mock-treated cells on each of the 12 plates in the experiment. (PDF) [file pone.0080999.s006.pdf]

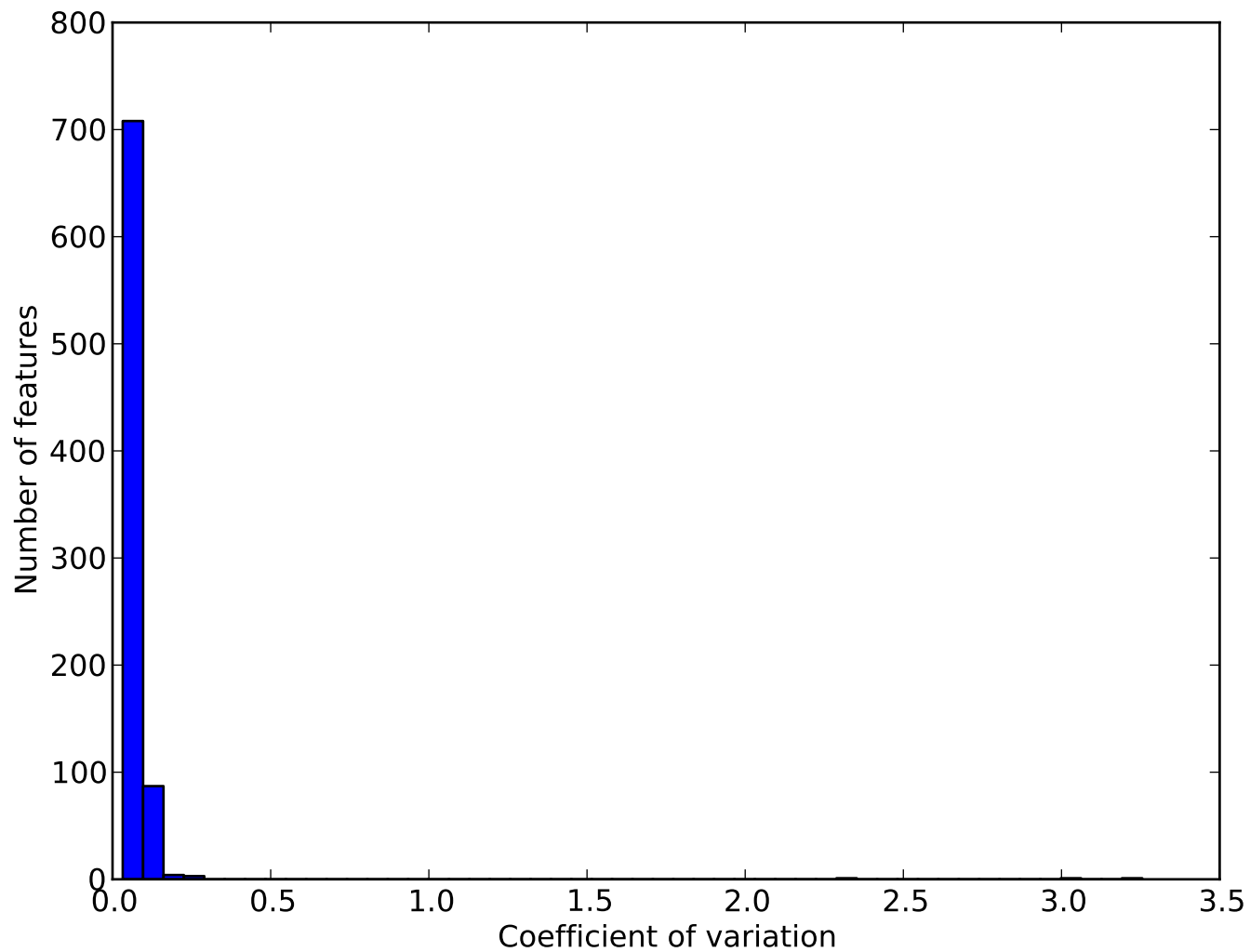

Supplement: Figure S3 — The well-to-well variability in the experiment is small (< 0.2) for the vast majority of features. The histogram shows the distribution of coefficients of variation (absolute value) across the features. Each coefficient was computed across the 64 well positions in which mock-treated cells appear on each plate in the experiment. (PDF) [file pone.0080999.s007.pdf]

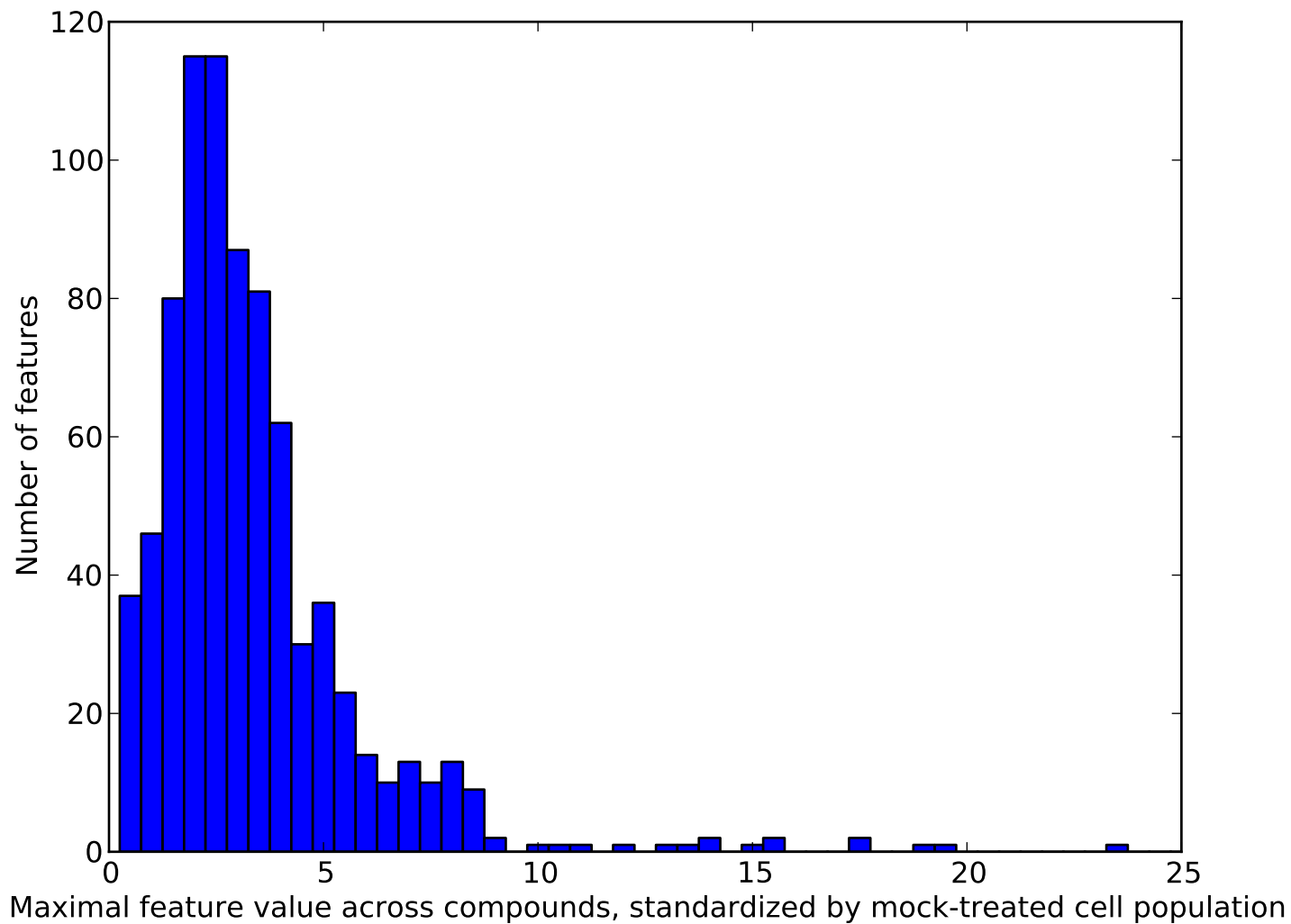

Supplement: Figure S4 — The magnitude of the compounds’ effects on the features. The histogram shows the distribution of maximal values of the features across the 75 active compounds in the experiment, standardized by reference to the population of mock-treated cells on the same plate. (PDF) [file pone.0080999.s008.pdf]
