# Supplementary material for: Multiplex Cytological Profiling Assay to Measure Diverse Cellular States
Source: PLoS One. 2013 Dec 2;8(12):e80999. doi: 10.1371/journal.pone.0080999 (PMC3847047; doi:10.1371/journal.pone.0080999)
Supplement: Table S1 — The 1600 bioactive compounds profiled using our assay. (DOCX) [file pone.0080999.s009.docx]

**Table S1:** The 1600 compounds profiled using our assay.

| Compound name | Concentration [µM] | Source | Broad ID | Structure |
| --- | --- | --- | --- | --- |
|  |  |  |  |  |
| (+ -)-2-(N-PHENYLETHYL-N-PROPYL)AMINO-5-HYDROXYTETRALIN) | 5.00 | Biomol International Inc. | BRD-A82843133 | 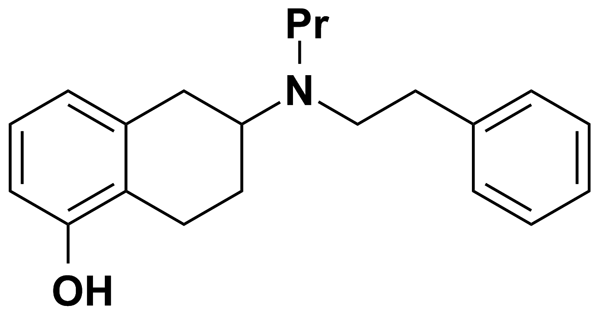 |
| (+ -)-BACLOFEN | 5.00 | Biomol International Inc. | BRD-A84174873 | 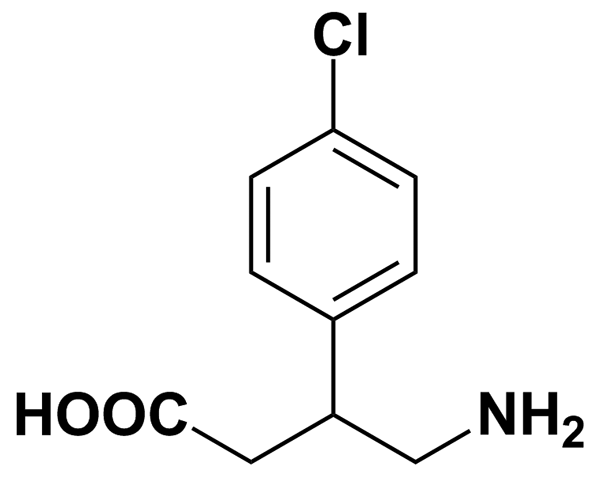 |
| (+ -)-SKF-82958 | 5.00 | Biomol International Inc. | BRD-A04438777 | 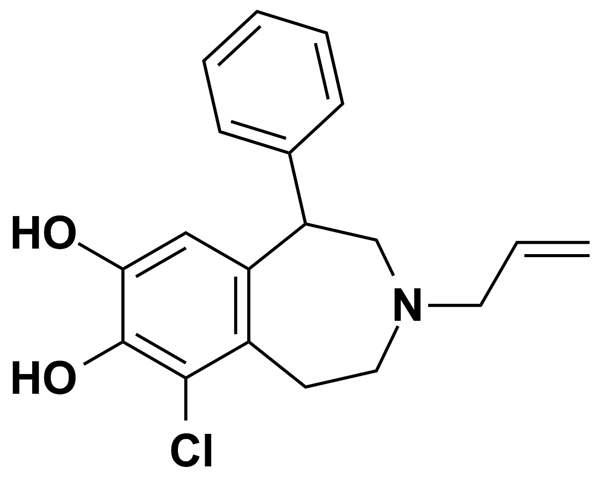 |
| (+)-BUTACLAMOL | 5.00 | Biomol International Inc. | BRD-K20620780 | 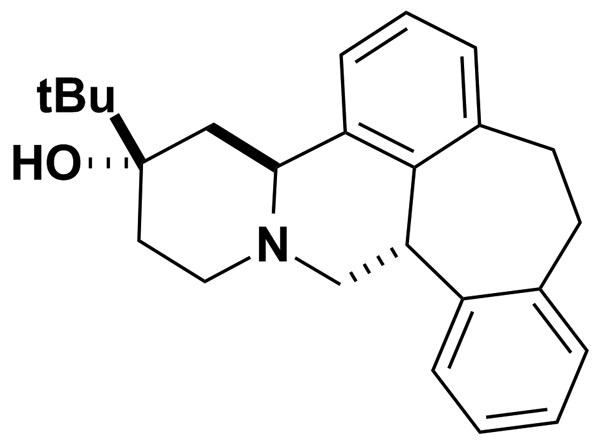 |
| (+)-MK 801 | 5.00 | Biomol International Inc. | BRD-K58930050 | 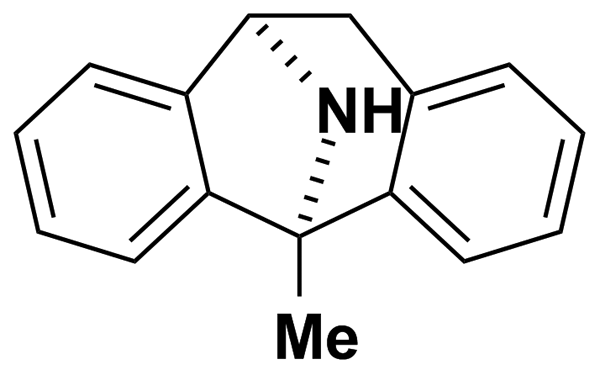 |
| (+)-PD 128907 | 5.00 | Biomol International Inc. | BRD-K62456038 | 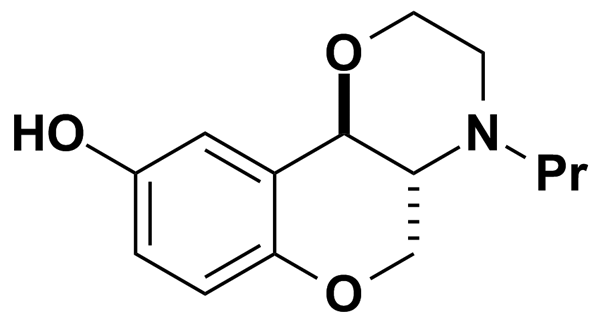 |
| (-)-CINCHONIDINE | 3.40 | Prestwick Chemical Inc. | BRD-K17661460 | 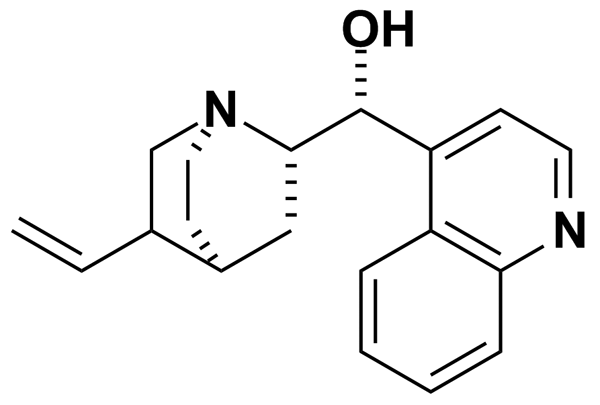 |
| (-)-QUINPIROLE | 5.00 | Biomol International Inc. | BRD-K26548821 | 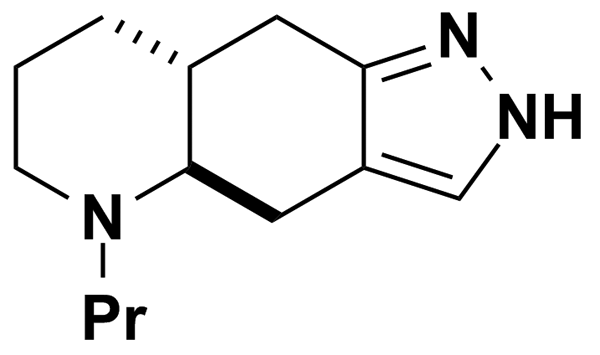 |
| (-)-QUINPIROLE HYDROCHLORIDE | 3.91 | Prestwick Chemical Inc. | BRD-K26548821 | 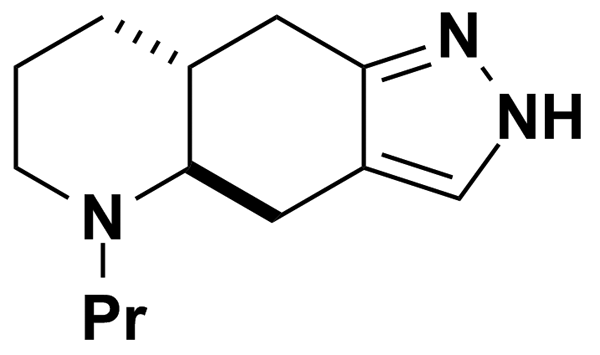 |
| (1S,9R)-BETA-HYDRASTINE | 5.00 | Biomol International Inc. | BRD-K95963688 | 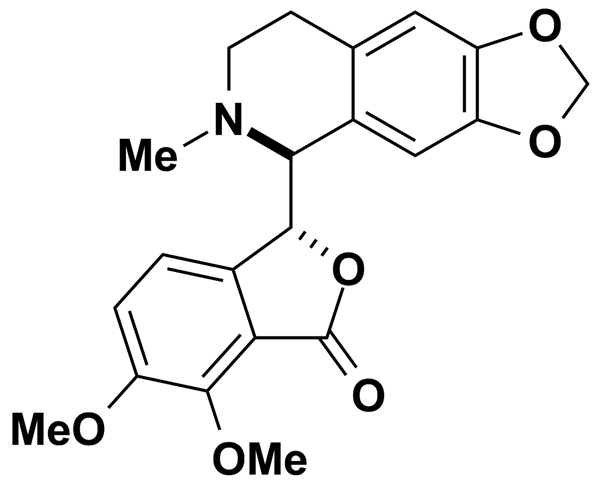 |
| (D,L)-TETRAHYDROBERBERINE | 2.95 | Prestwick Chemical Inc. | BRD-A69950438 | 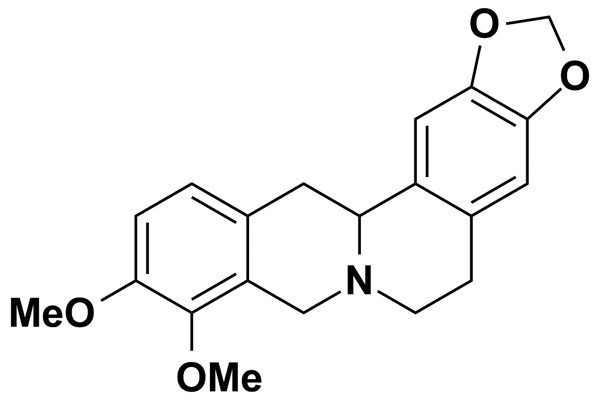 |
| (R)-PROPRANOLOL HYDROCHLORIDE | 3.38 | Prestwick Chemical Inc. | BRD-K13994703 | 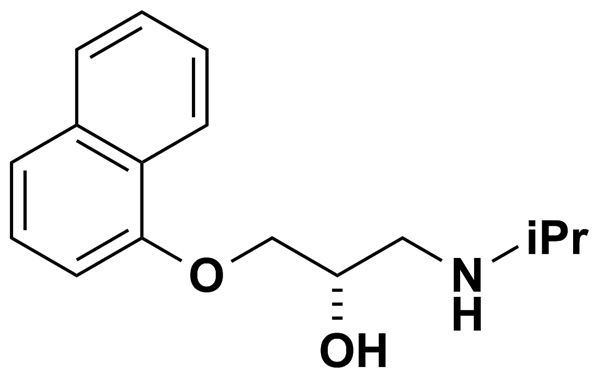 |
| (RS)-(+ -)-SULPIRIDE | 5.00 | Biomol International Inc. | BRD-A55272860 | 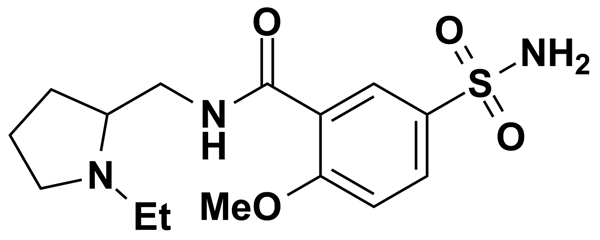 |
| (S)-(-)-ATENOLOL | 3.75 | Prestwick Chemical Inc. | BRD-K68075732 | 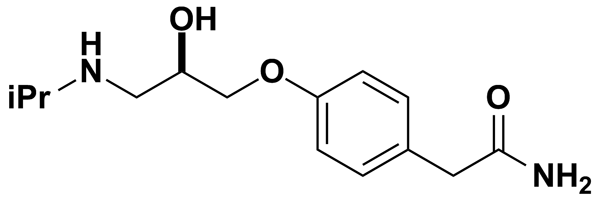 |
| 1,25-DIHYDROXYVITAMIN D3 | 0.50 | Biomol International Inc. | BRD-K27316855 | 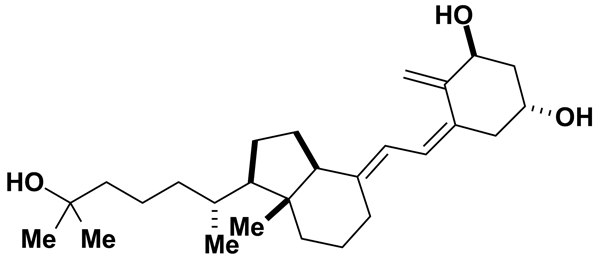 |
| 1,2ALPHA-EPOXY-7-DEACETOXY-7-OXODIHYDROGEDUNIN | 5.00 | MicroSource Discovery Systems Inc. | BRD-A86550573 | 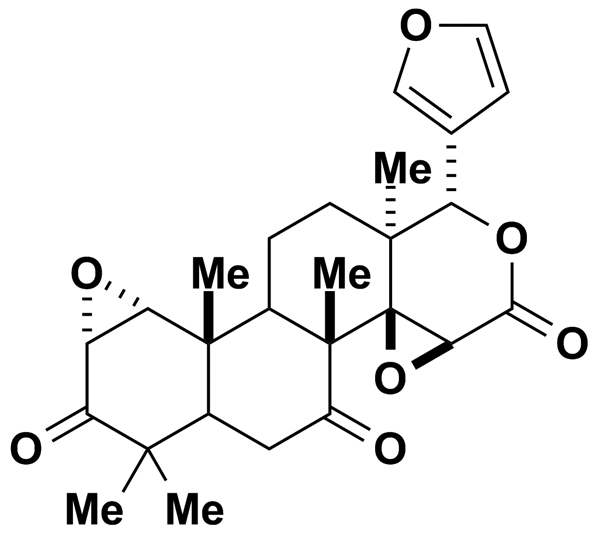 |
| 1,3,5-TRIMETHOXYBENZENE | 5.00 | MicroSource Discovery Systems Inc. | BRD-K81884069 | 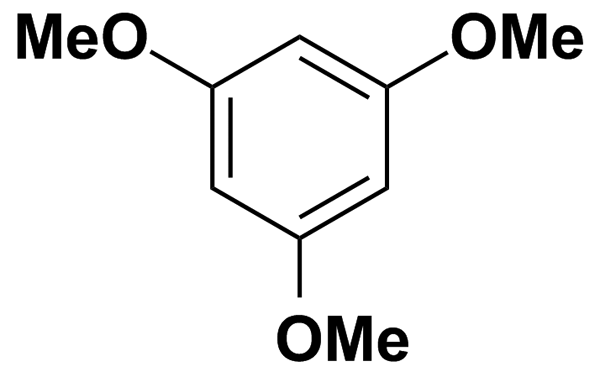 |
| 1,3-DIDEACETYL-7-DEACETOXY-7-OXOKHIVORIN | 5.00 | MicroSource Discovery Systems Inc. | BRD-A34891365 | 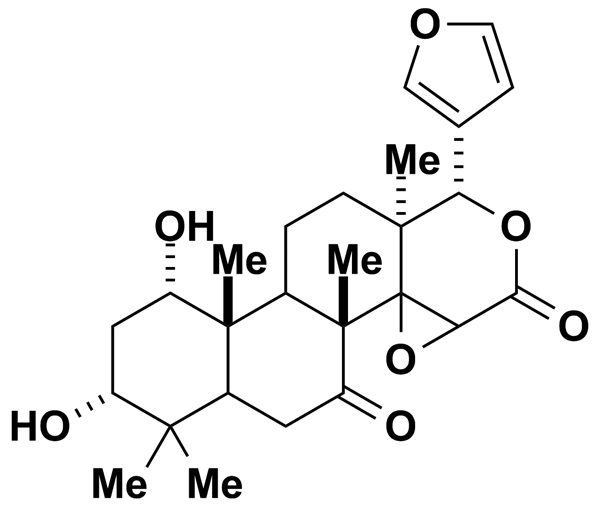 |
| 1,3-DIDEACETYLKHIVORIN | 5.00 | MicroSource Discovery Systems Inc. | BRD-A67787745 | 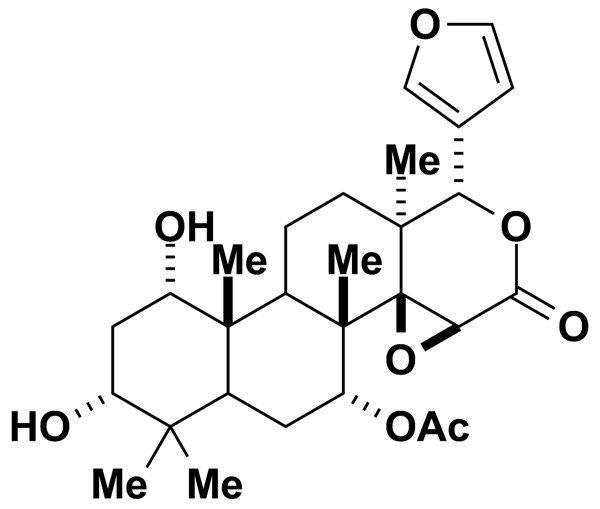 |
| 1,3-DIPROPYL-8-CYCLOPENTYLXANTHINE [DPCPX] | 5.00 | MicroSource Discovery Systems Inc. | BRD-K01824921 | 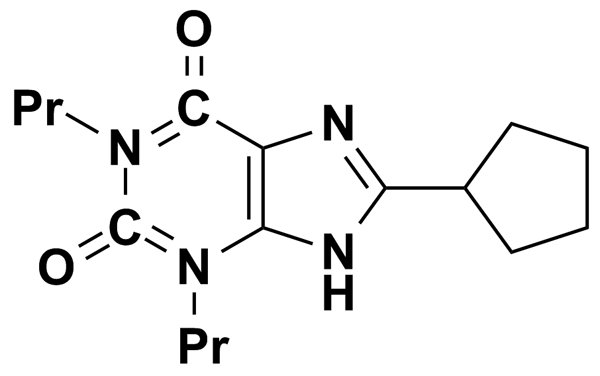 |
| 1,7-DIDEACETOXY-1,7-DIOXO-3-DEACETYLKHIVORIN | 5.00 | MicroSource Discovery Systems Inc. | BRD-A12372458 | 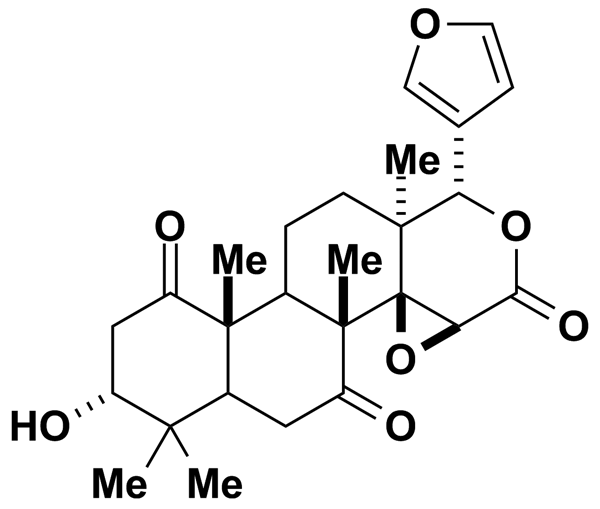 |
| 1- (2-METHOXYPHENYL)PIERAZINE HYDROCHLORIDE | 5.00 | MicroSource Discovery Systems Inc. | BRD-K70343553 | 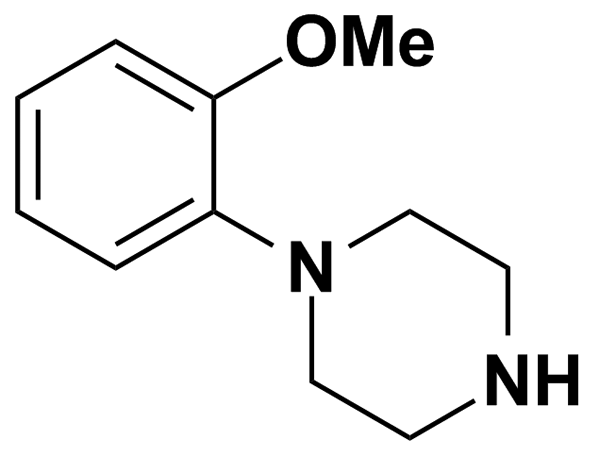 |
| 1-(1,3-BENZODIOXOL-5-YLCARBONYL)PIPERIDINE | 5.00 | Biomol International Inc. | BRD-K25140590 | 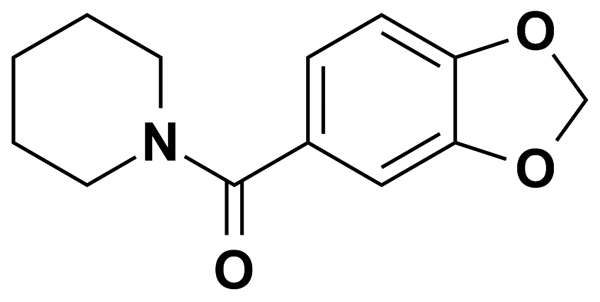 |
| 1-(1-NAPHTHYL)PIPERAZINE | 5.00 | Biomol International Inc. | BRD-K05977380 | 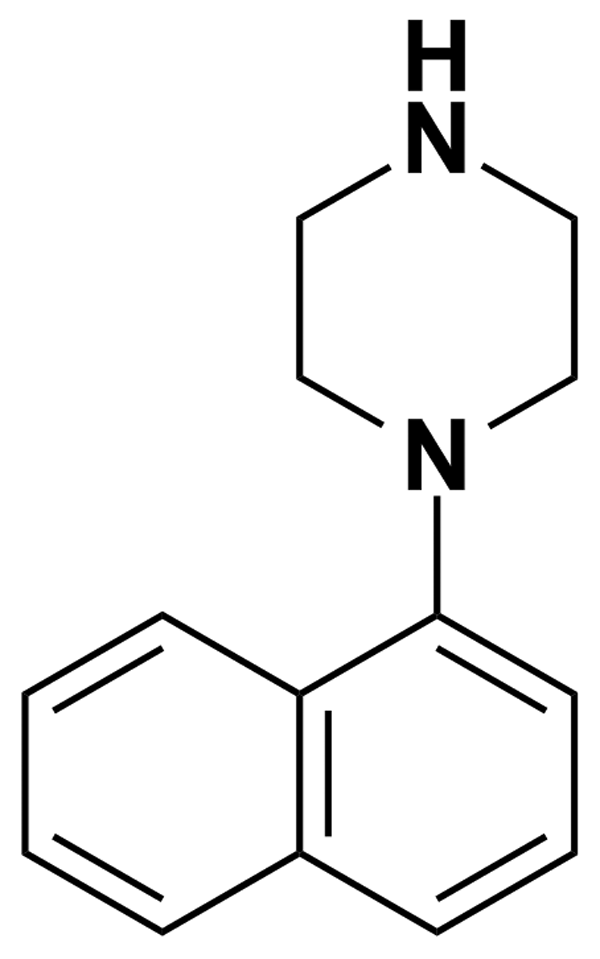 |
| 1-(3-CHLOROPHENYL)PIPERAZINE | 5.00 | Biomol International Inc. | BRD-K75844781 | 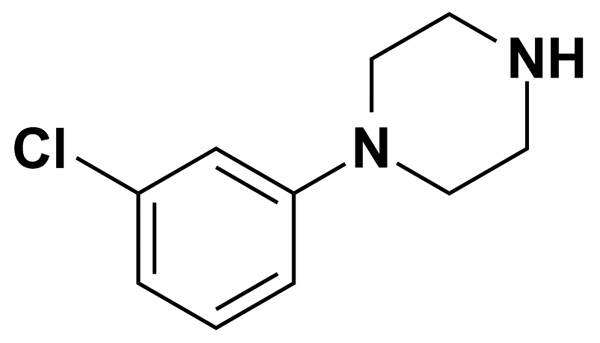 |
| 1-(3-TRIFLUOROMETHYL) PHENYLPIPERAZINE MONOHYDROCHLORIDE | 5.00 | Biomol International Inc. | BRD-K94887716 | 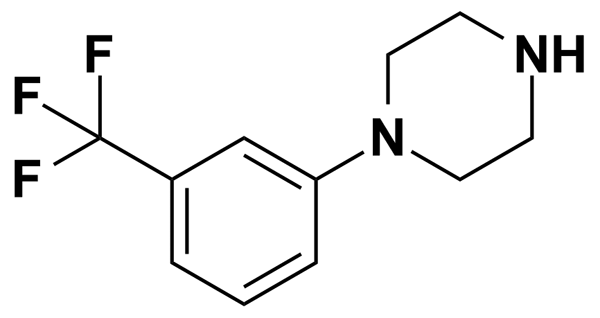 |
| 1-(4'-AMINOPHENYL)-3,5-DIHYDRO-7,8-DIMETHOXY-4H-2,3-BENZODIAZEPIN-4-ONE | 5.00 | Biomol International Inc. | BRD-K42859542 | 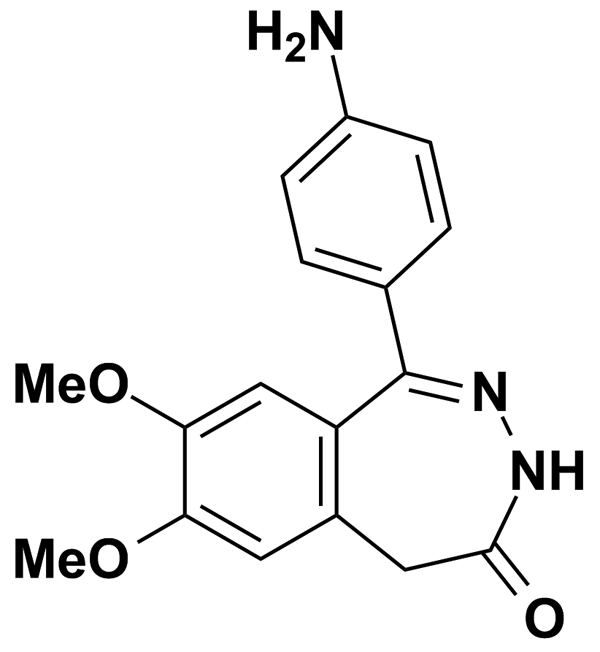 |
| 1-PHENYLBIGUANIDE HYDROCHLORIDE | 5.00 | MicroSource Discovery Systems Inc. | BRD-K31491153 | 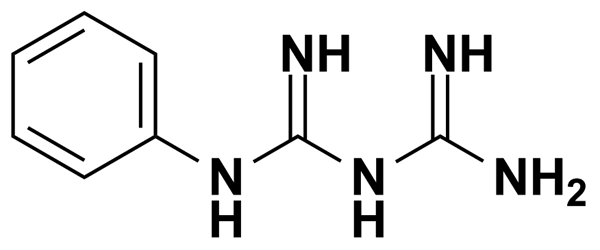 |
| 1-[1-(2-BENZO[B]THIENYL)CYCLOHEXYL)]PIPERIDINE | 5.00 | Biomol International Inc. | BRD-K15519488 | 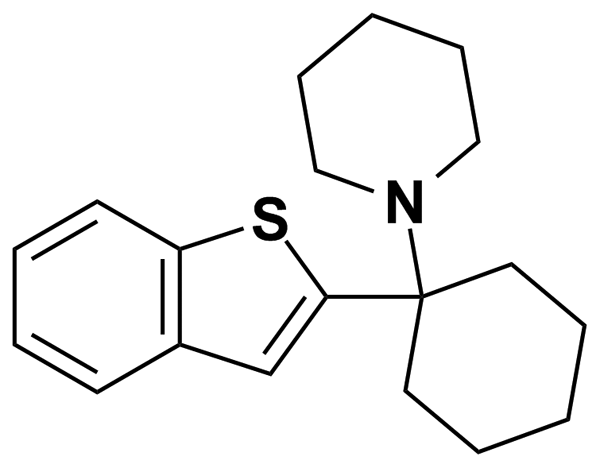 |
| 10-HYDROXYCAMPTOTHECIN | 6.86 | Biomol International Inc. | BRD-K01978082 | 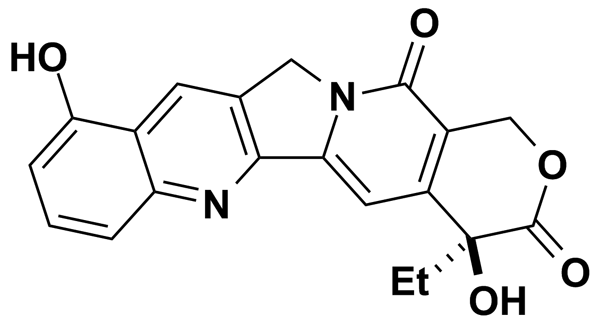 |
| 11A-ACETOXYPROGESTERONE | 5.00 | MicroSource Discovery Systems Inc. | BRD-A56884981 | 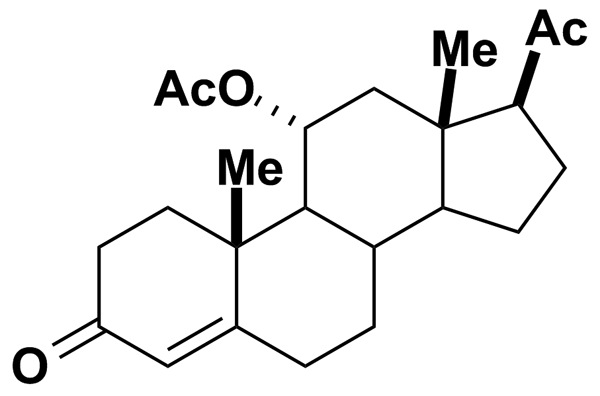 |
| 11ALPHA-ACETOXYKHIVORIN | 5.00 | MicroSource Discovery Systems Inc. | BRD-A83392615 | 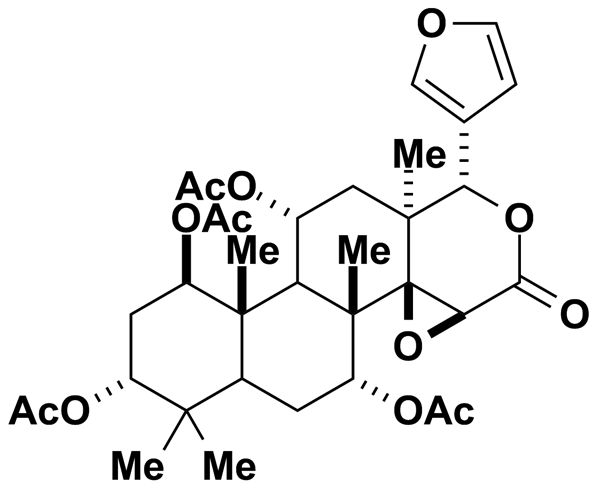 |
| 13-CIS RETINOIC ACID | 0.50 | Biomol International Inc. | BRD-K76723084 | 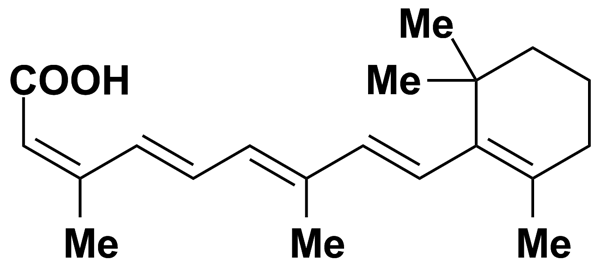 |
| 17-ALLYLAMINO-GELDANAMYCIN | 4.27 | Biomol International Inc. | BRD-K50523832 | 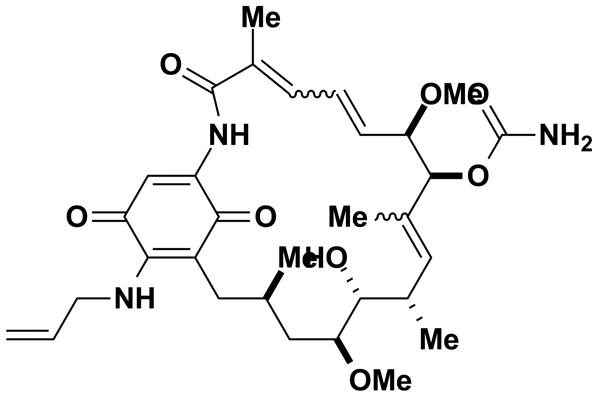 |
| 17-PHENYL-TRINOR-PGE2 | 0.50 | Biomol International Inc. | BRD-K90214371 | 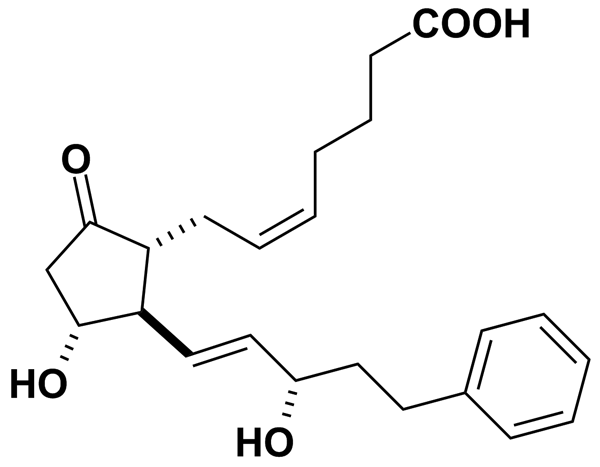 |
| 18-AMINOABIETA-8,11,13-TRIENE SULFATE | 5.00 | MicroSource Discovery Systems Inc. | BRD-A27080191 | 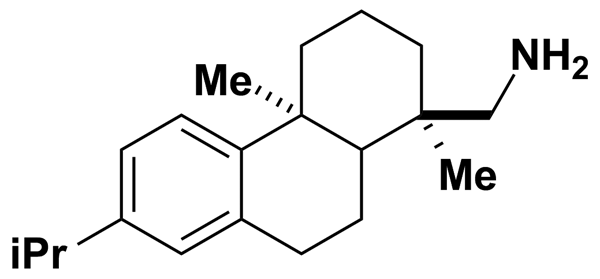 |
| 1R,2S-PHENYLPROPYLAMINE | 5.00 | MicroSource Discovery Systems Inc. | BRD-K27853494 | 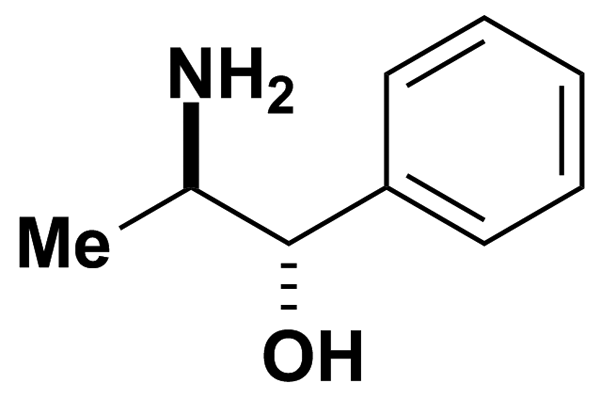 |
| 1R,9S-HYDRASTINE | 5.00 | MicroSource Discovery Systems Inc. | BRD-K77435797 | 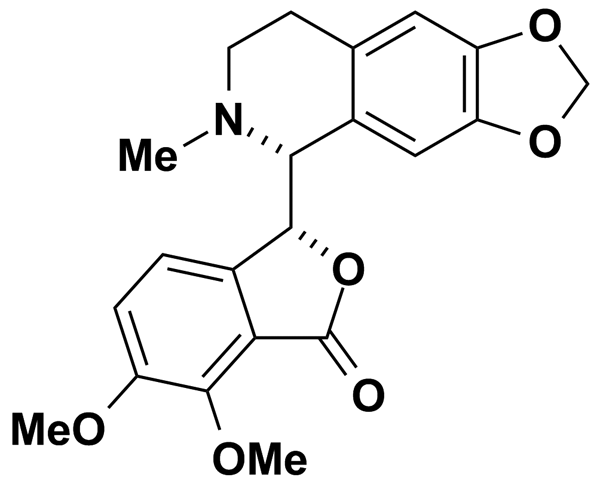 |
| 1S,9R-HYDRASTINE | 5.00 | MicroSource Discovery Systems Inc. | BRD-K61271364 | 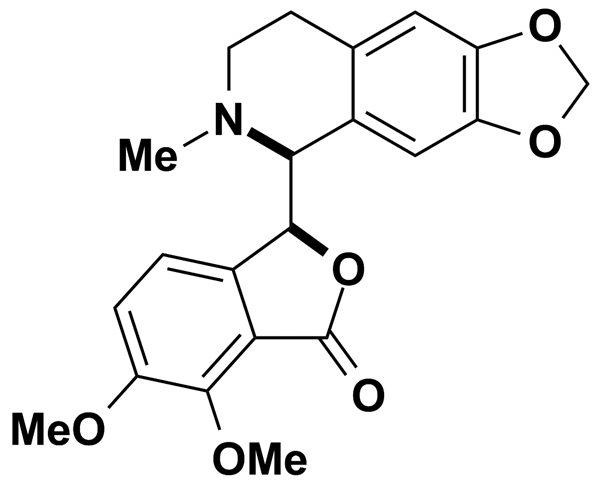 |
| 2',4'-DIHYDROXYCHALCONE 4'-GLUCOSIDE | 5.00 | MicroSource Discovery Systems Inc. | BRD-K55250255 | 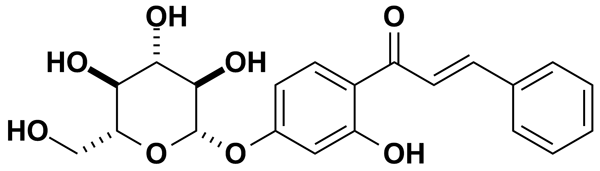 |
| 2',4-DIHYDROXY-3,4',6'-TRIMETHOXYCHALCONE | 5.00 | MicroSource Discovery Systems Inc. | BRD-K88111944 | 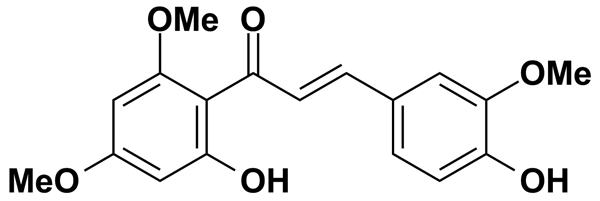 |
| 2',5'-DIHYDROXY-4-METHOXYCHALCONE | 5.00 | MicroSource Discovery Systems Inc. | BRD-K43285163 | 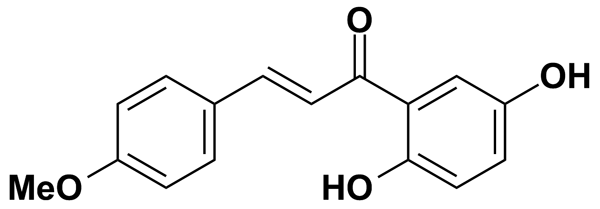 |
| 2',BETA-DIHYDROXYCHALCONE | 5.00 | MicroSource Discovery Systems Inc. | BRD-K95432622 | 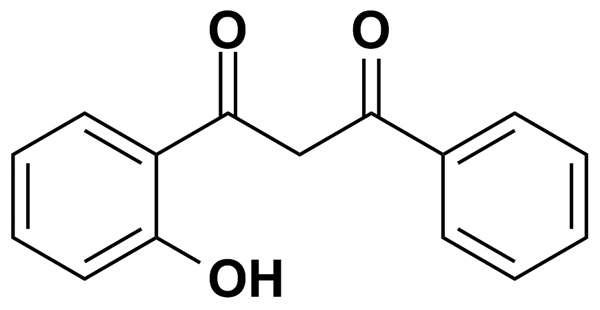 |
| 2'-METHOXYFORMONETIN | 5.00 | MicroSource Discovery Systems Inc. | BRD-K84390227 | 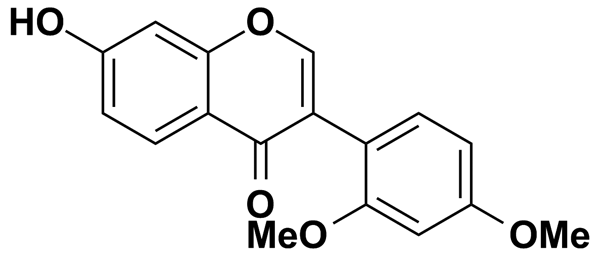 |
| 2,3,4-TRIHYDROXY-4'-ETHOXYBENZOPHENONE | 5.00 | MicroSource Discovery Systems Inc. | BRD-K15464532 | 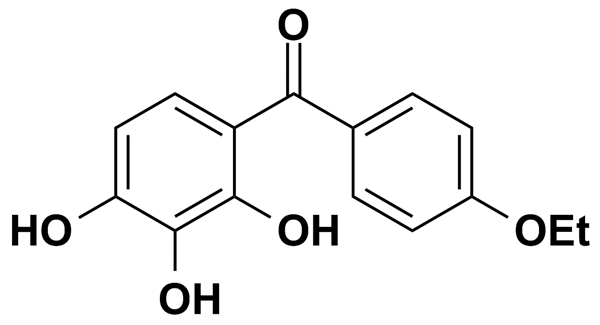 |
| 2,3-DIOXO-6-NITRO-1,2,3,4-TETRAHYDROBENZO[F]QUINOXALINE-7-SULFONAMIDE | 5.00 | Biomol International Inc. | BRD-K11796549 | 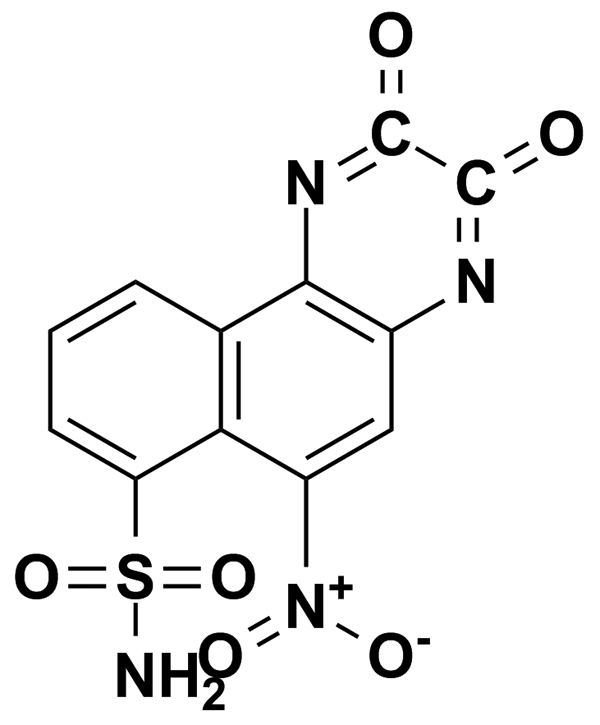 |
| 2,4,5-TRICHLOROPHENOXYACETIC ACID | 5.00 | MicroSource Discovery Systems Inc. | BRD-K92860374 | 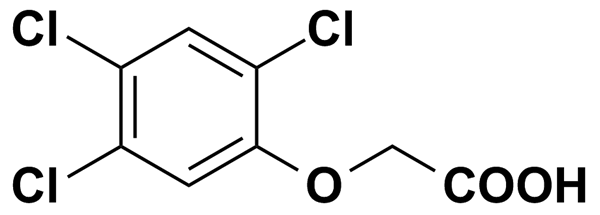 |
| 2,4-DICHLOROPHENOXYACETIC ACID | 5.00 | MicroSource Discovery Systems Inc. | BRD-K01473791 | 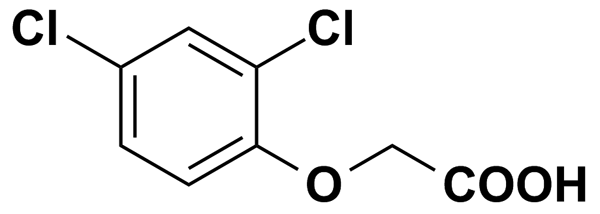 |
| 2,4-DINITROPHENOL | 5.00 | MicroSource Discovery Systems Inc. | BRD-K21910317 | 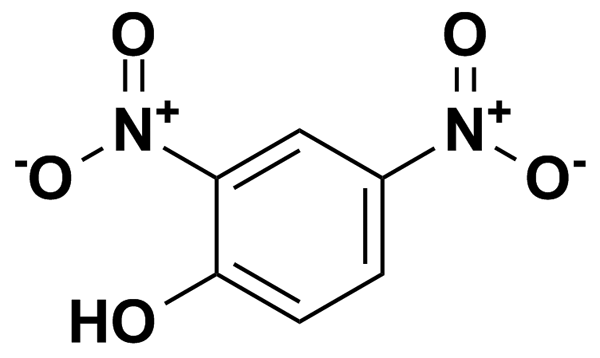 |
| 2,5-DITERTBUTYLHYDROQUINONE | 11.24 | Biomol International Inc. | BRD-K95603879 | 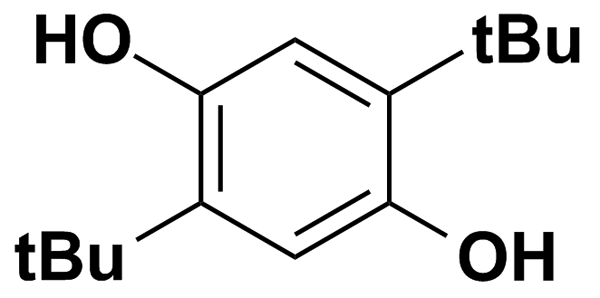 |
| 2,6-DI-T-BUTYL-4-METHYLPHENOL | 5.00 | MicroSource Discovery Systems Inc. | BRD-K53153417 | 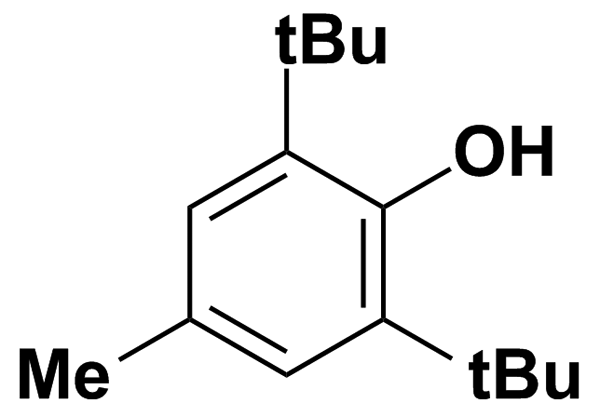 |
| 2,6-DIHYDROXY-4-METHOXYTOLUENE | 5.00 | MicroSource Discovery Systems Inc. | BRD-K35568433 | 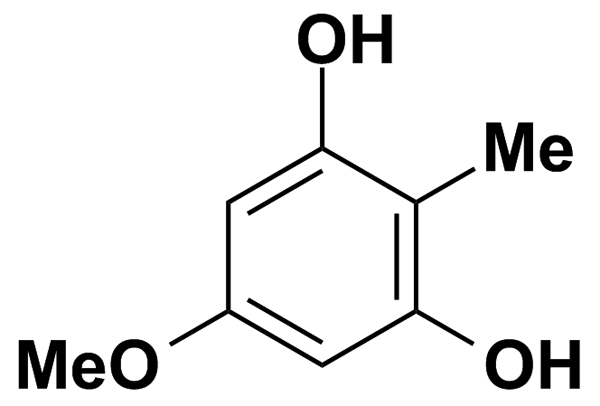 |
| 2- (2,6-DIMETHOXYPHENOXYETHYL)AMINOMETHYL-1,4-BENZODIOXANE HYDROCHLORIDE (WB 4101) | 5.00 | MicroSource Discovery Systems Inc. | BRD-A01493904 | 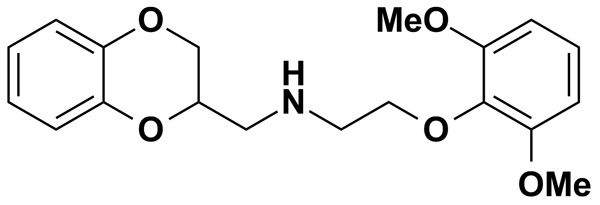 |
| 2-ARACHIDONOYLGLYCEROL | 0.50 | Biomol International Inc. | BRD-K71198913 | 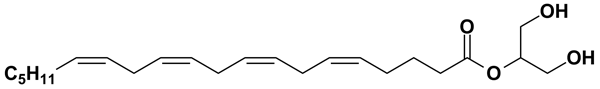 |
| 2-CHLORO-11-(4-METHYLPIPERAZINO)DIBENZ[B,F]OXEPIN | 5.00 | Biomol International Inc. | BRD-K92588747 | 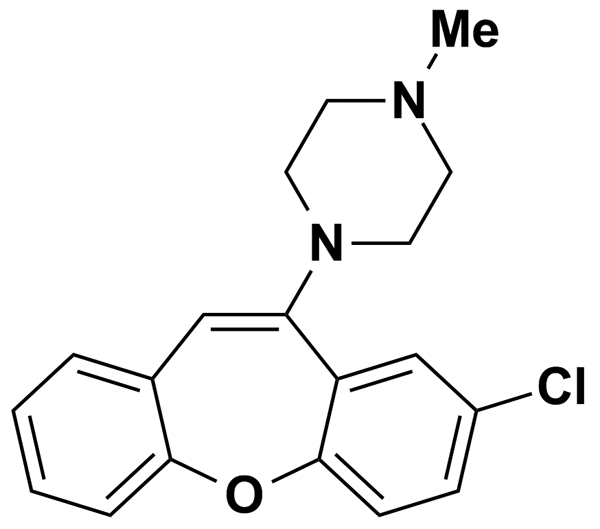 |
| 2-HYDROXYXANTHONE | 5.00 | MicroSource Discovery Systems Inc. | BRD-K12336887 | 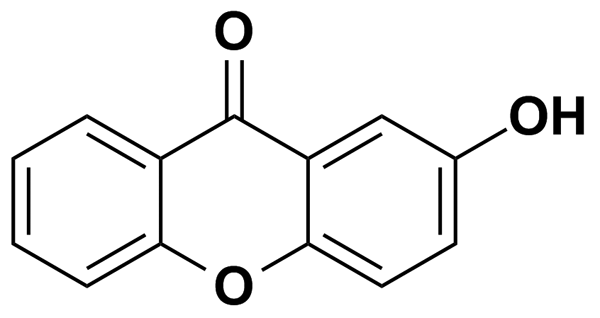 |
| 2-ISOPROPYL-3-METHOXYCINNAMIC ACID | 5.00 | MicroSource Discovery Systems Inc. | BRD-K11813414 | 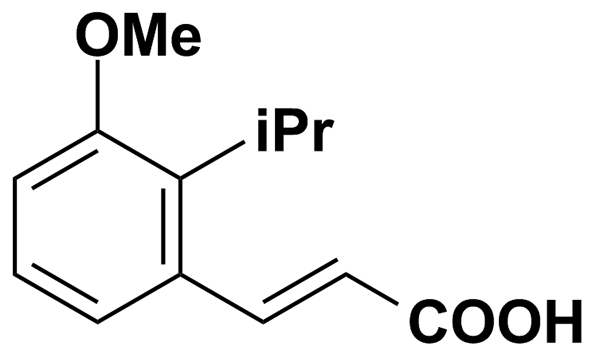 |
| 2-MERCAPTOBENZOTHIAZOLE | 5.00 | MicroSource Discovery Systems Inc. | BRD-K55160477 | 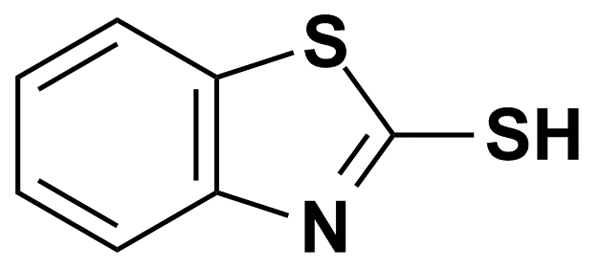 |
| 2-METHYL-3-HYDROXYETHYLENEPYRAN-4-ONE | 5.00 | MicroSource Discovery Systems Inc. | BRD-K49745710 | 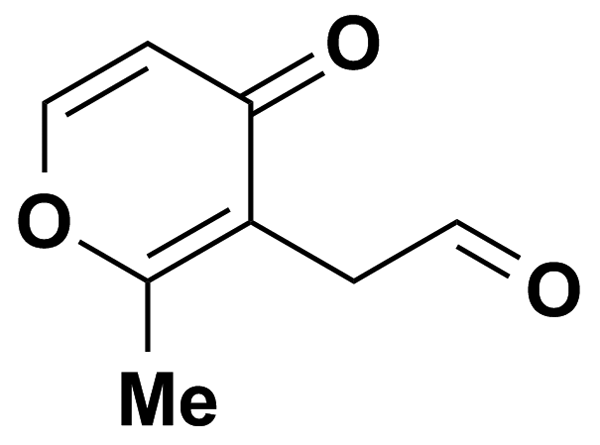 |
| 2-METHYLENE-5-(2,5-DIOXOTETRAHYDROFURAN-3-YL)-6-OXO--10,10-DIMETHYLBICYCLO[7: 2: 0]UNDECANE | 5.00 | MicroSource Discovery Systems Inc. | BRD-A58803929 | 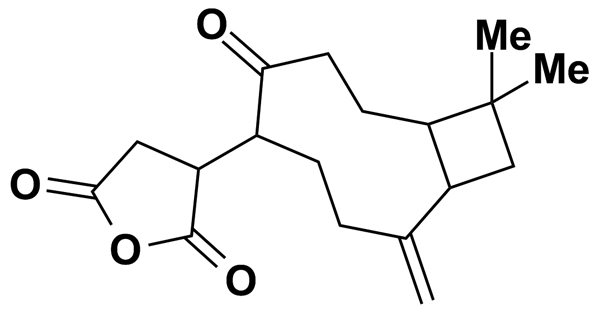 |
| 2-PROPYL-3-HYDROXYETHYLENEPYRAN-4-ONE | 5.00 | MicroSource Discovery Systems Inc. | BRD-K03470412 | 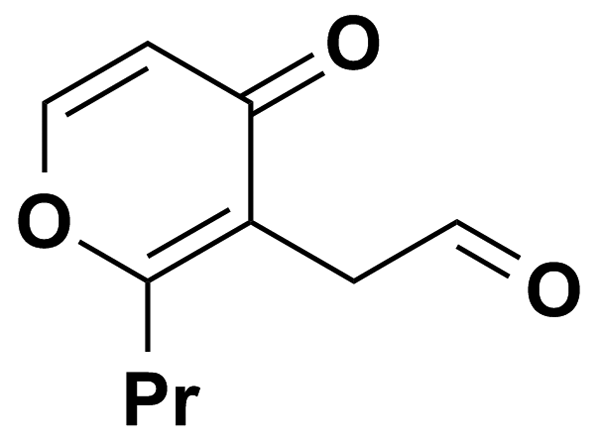 |
| 2-[1-(4-PIPERONYL)PIPERAZINYL]BENZOTHIAZOLE | 5.00 | Biomol International Inc. | BRD-K91868854 | 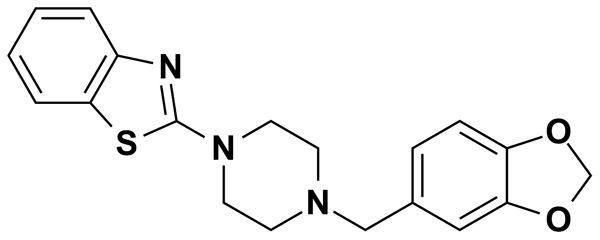 |
| 24,25-DIHYDROXYVITAMIN D3 | 0.50 | Biomol International Inc. | BRD-K02965577 | 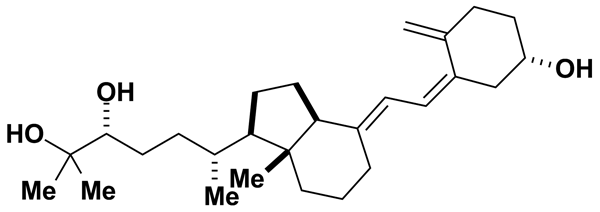 |
| 3'-FLUOROBENZYLSPIPERONE | 5.00 | Biomol International Inc. | BRD-K45662124 | 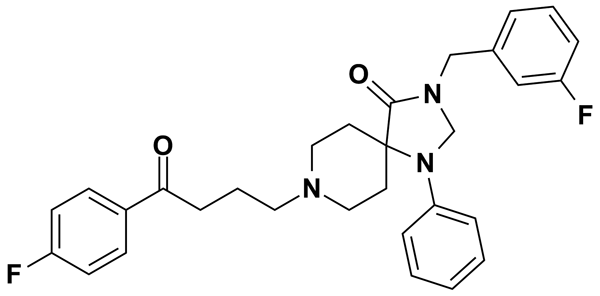 |
| 3,4-DICHLOROISOCOUMARIN | 11.63 | Biomol International Inc. | BRD-K23704908 | 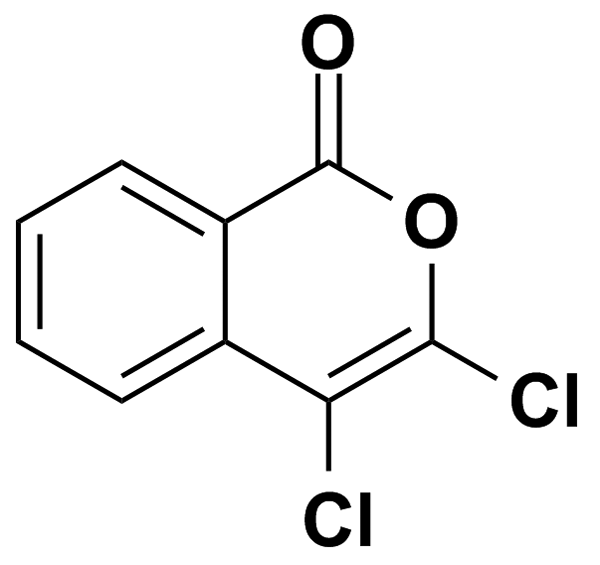 |
| 3,7-DIHYDROXYFLAVONE | 5.00 | MicroSource Discovery Systems Inc. | BRD-K35133923 | 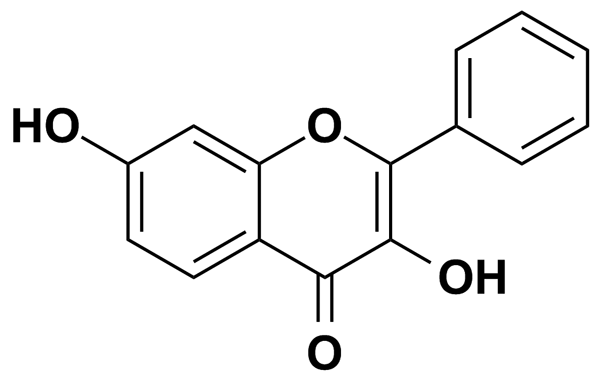 |
| 3-ACETAMIDOCOUMARIN | 4.92 | Prestwick Chemical Inc. | BRD-K85104575 | 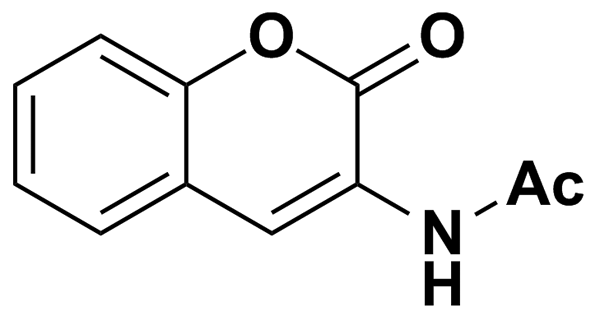 |
| 3-AMINO-BETA-PINENE | 5.00 | MicroSource Discovery Systems Inc. | BRD-A31576716 | 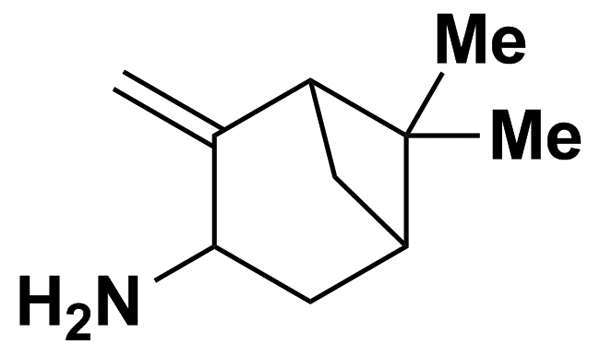 |
| 3-CHLORO-8BETA-HYDROXYCARAPIN, 3,8-HEMIACETAL | 5.00 | MicroSource Discovery Systems Inc. | BRD-A66979321 | 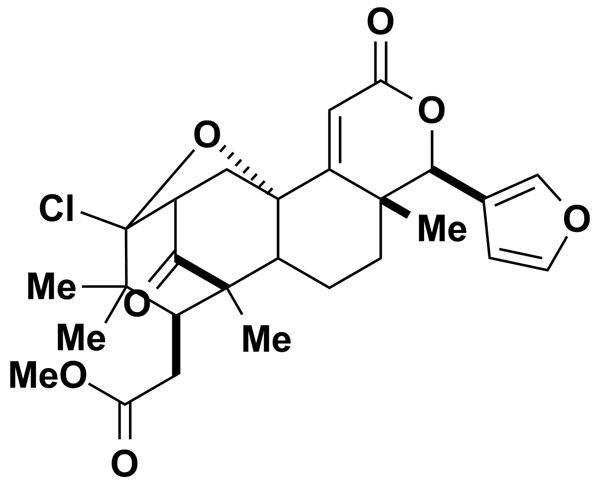 |
| 3-DEOXO-3BETA-ACETOXYDEOXYDIHYDROGEDUNIN | 5.00 | MicroSource Discovery Systems Inc. | BRD-A20182314 | 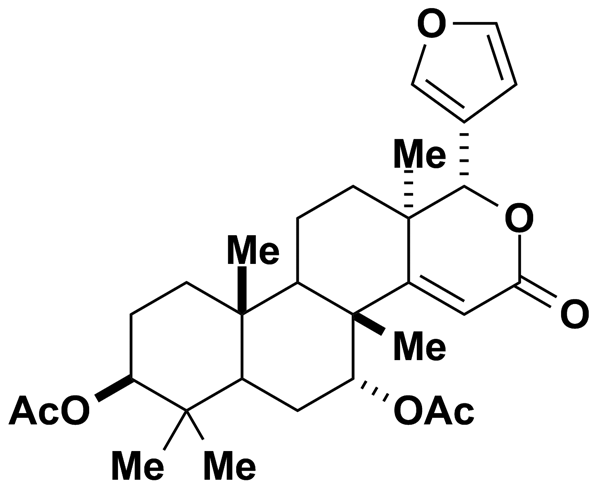 |
| 3-DEOXY-3BETA-HYDROXYANGOLENSIC ACID METHYL ESTER | 5.00 | MicroSource Discovery Systems Inc. | BRD-K54554531 | 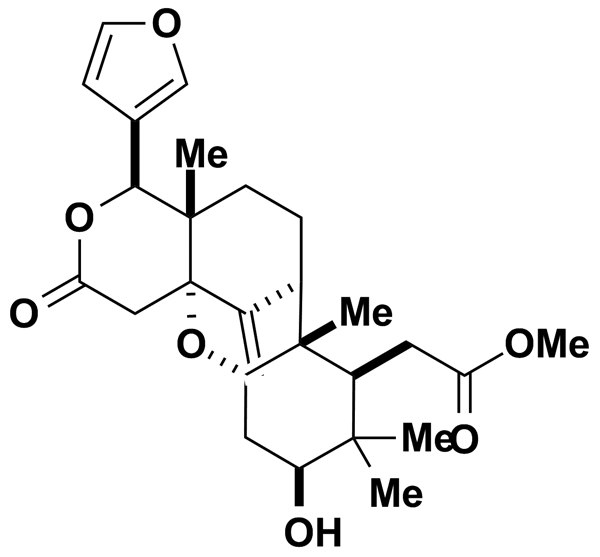 |
| 3-HYDROXYBENZYLHYDRAZINE DIHYDROCHLORIDE | 5.00 | MicroSource Discovery Systems Inc. | BRD-K66416915 | 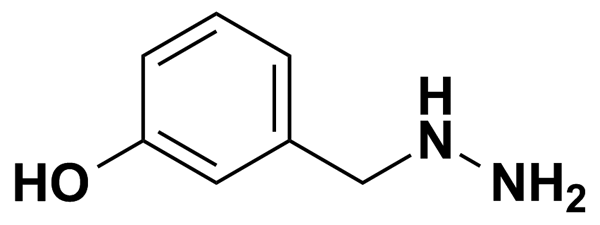 |
| 3-HYDROXYCOUMARIN | 5.00 | MicroSource Discovery Systems Inc. | BRD-K92019075 | 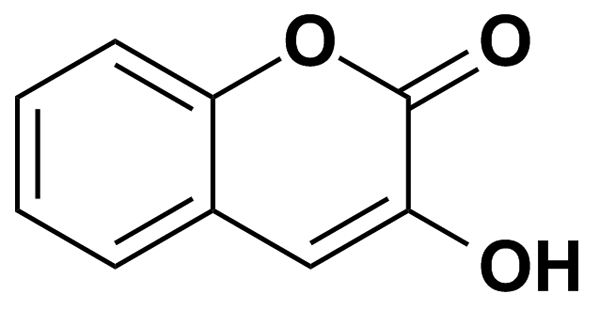 |
| 3-HYDROXYMETHYL-BETA-CARBOLINE | 5.00 | Biomol International Inc. | BRD-K69585439 | 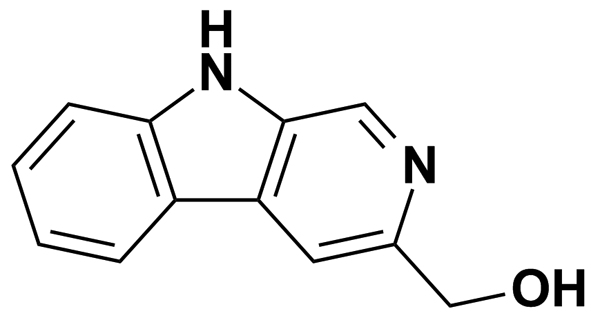 |
| 3-METHYLCHOLANTHRENE | 5.00 | MicroSource Discovery Systems Inc. | BRD-K61463582 | 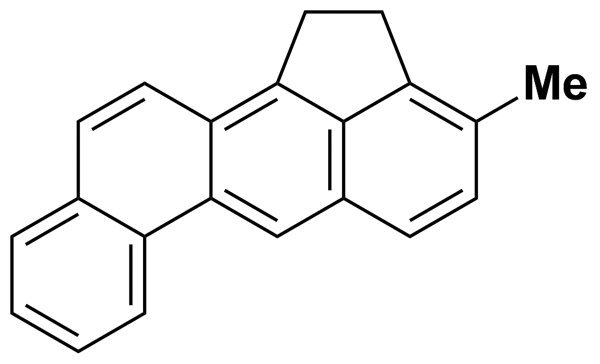 |
| 3-PRENYL-4-HYDROXYACETOPHENONE | 5.00 | MicroSource Discovery Systems Inc. | BRD-K14262397 | 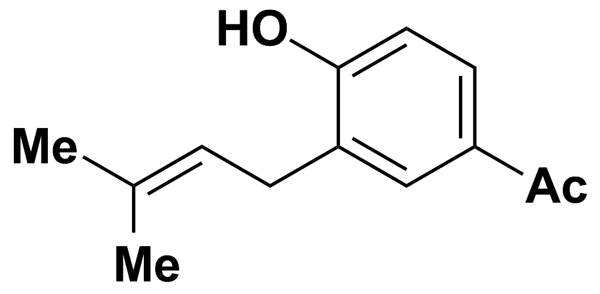 |
| 3-[2-[4-(2-METHOXYPHENYL)PIPERAZIN-1-YL]PYRIMIDO[5,4-B]INDOLE-2,4-DIONE | 5.00 | Biomol International Inc. | BRD-K62581435 | 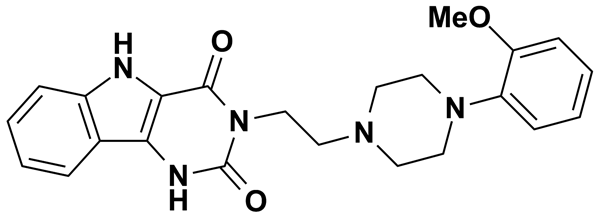 |
| 3ALPHA-ACETOXYDIHYDRODEOXYGEDUNIN | 5.00 | MicroSource Discovery Systems Inc. | BRD-A14098586 | 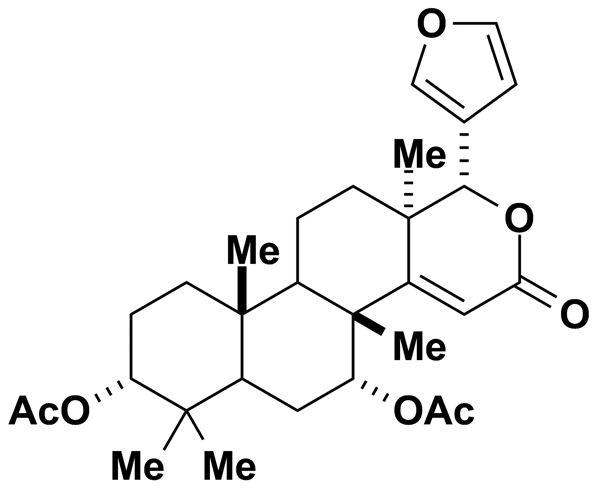 |
| 4'-DEMETHYLEPIPODOPHYLLOTOXIN | 5.00 | MicroSource Discovery Systems Inc. | BRD-A34380114 | 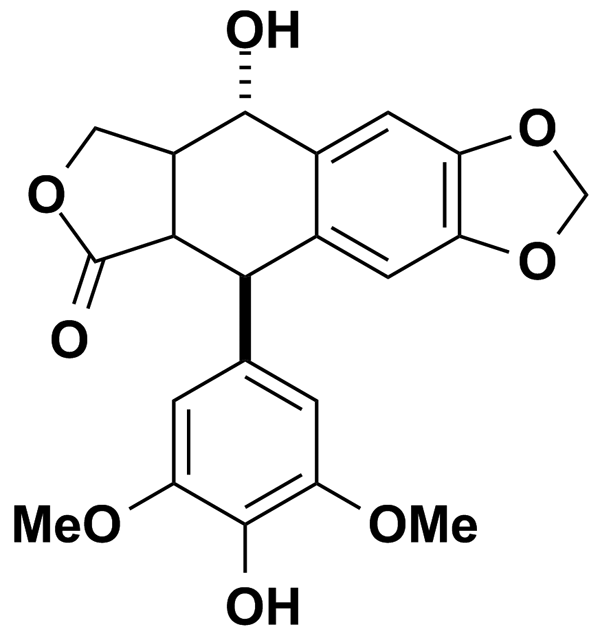 |
| 4'-HYDROXYCHALCONE | 5.00 | MicroSource Discovery Systems Inc. | BRD-K85068298 | 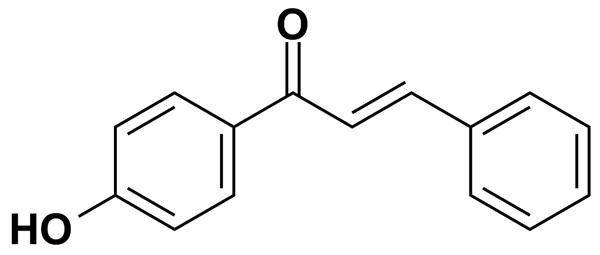 |
| 4'-METHOXYFLAVONE | 5.00 | MicroSource Discovery Systems Inc. | BRD-K76337122 | 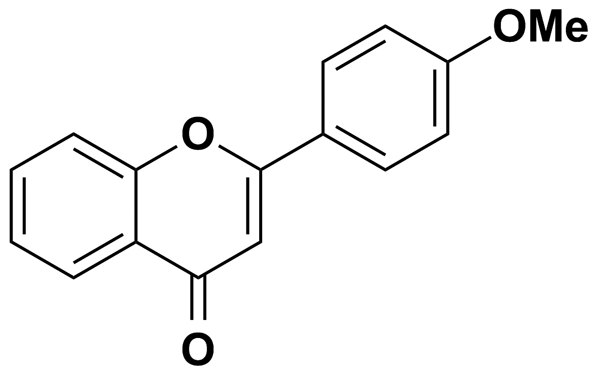 |
| 4,7-DIMETHOXYFLAVONE | 5.00 | MicroSource Discovery Systems Inc. | BRD-K16115965 | 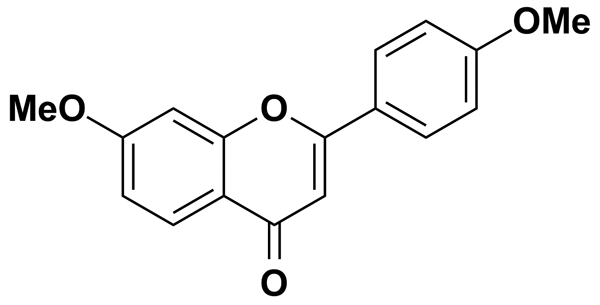 |
| 4-HYDROXY-6-METHYLPYRAN-2-ONE | 5.00 | MicroSource Discovery Systems Inc. | BRD-K77202630 | 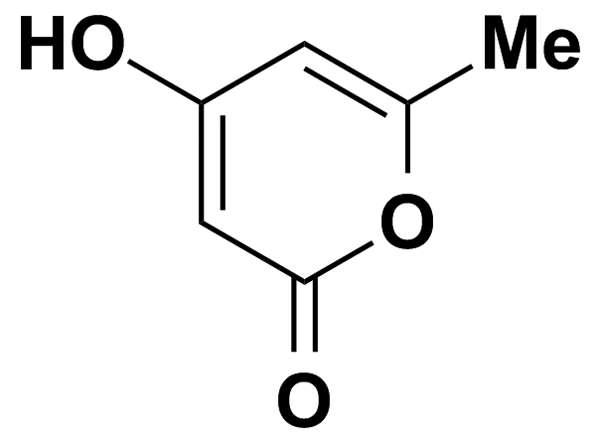 |
| 4-METHYLDAPHNETIN | 5.00 | MicroSource Discovery Systems Inc. | BRD-K49207204 | 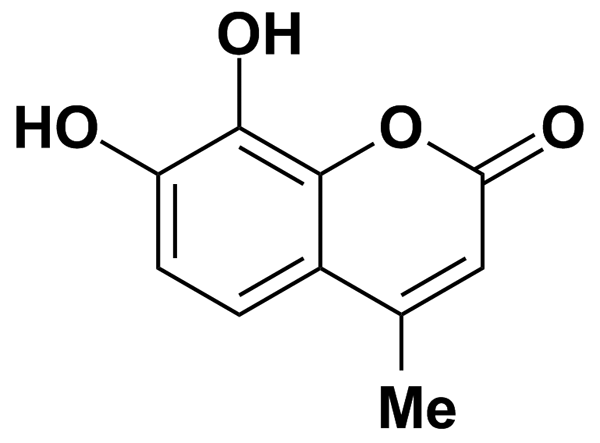 |
| 4-NAPHTHALIMIDOBUTYRIC ACID | 5.00 | MicroSource Discovery Systems Inc. | BRD-K60241851 | 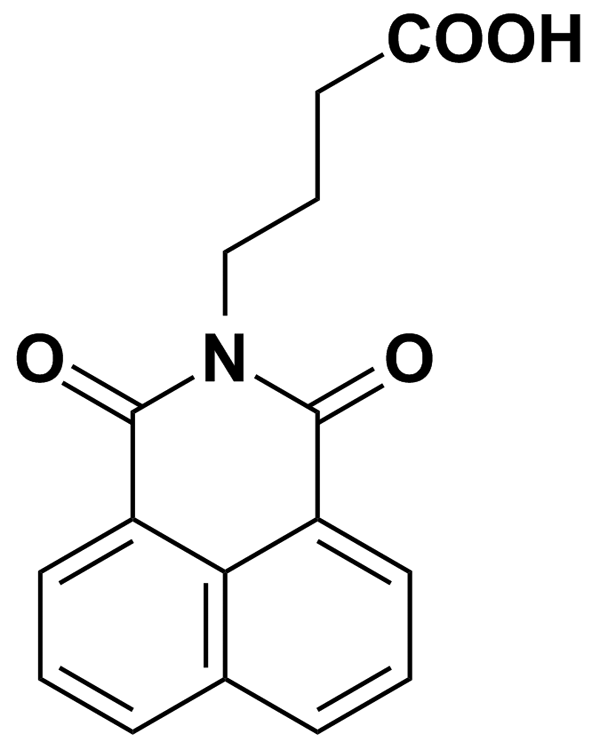 |
| 4-PHENYL-1,2,3,4-TETRAHYDROISOQUINOLINE | 5.00 | Biomol International Inc. | BRD-A40504327 | 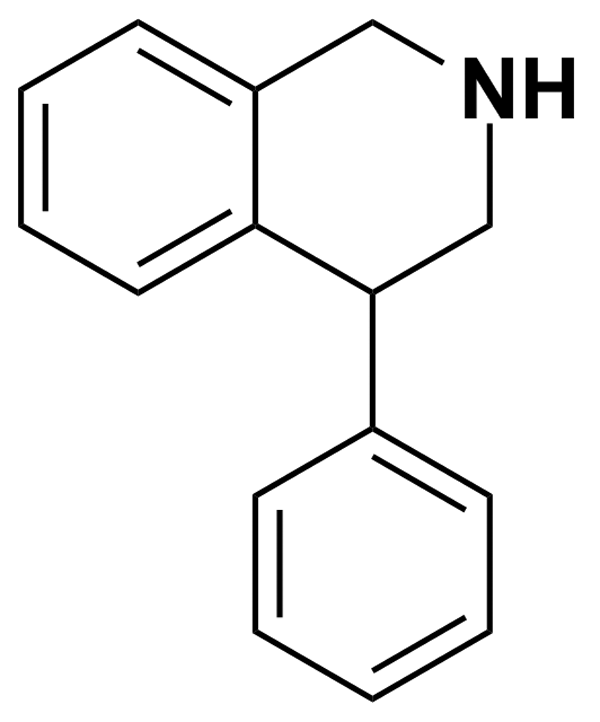 |
| 5'-N-ETHYLCARBOXAMIDOADENOSINE (NECA) | 8.11 | Biomol International Inc. | BRD-A57536034 | 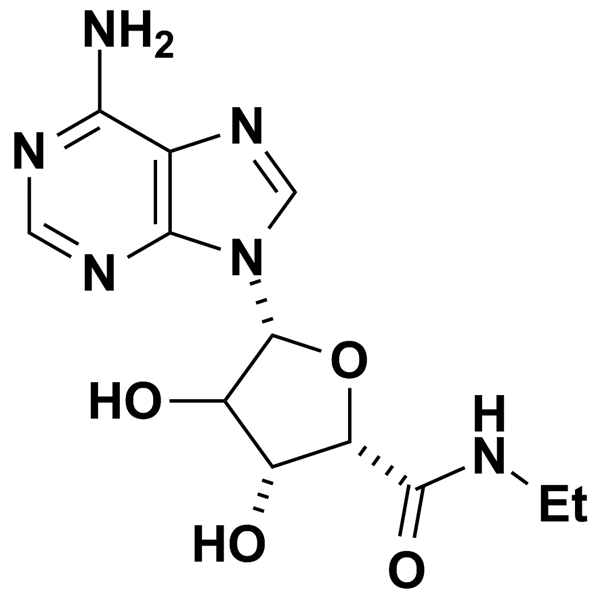 |
| 5,2'-DIMETHOXYFLAVONE | 5.00 | MicroSource Discovery Systems Inc. | BRD-K98582893 | 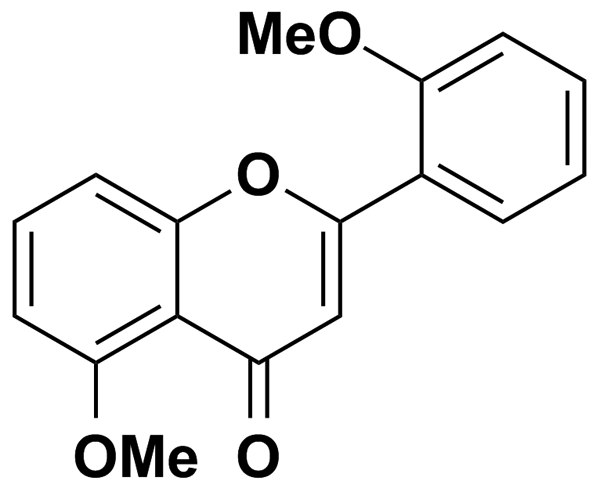 |
| 5,7,4'-TRIMETHOXYFLAVONE | 5.00 | MicroSource Discovery Systems Inc. | BRD-K68806283 | 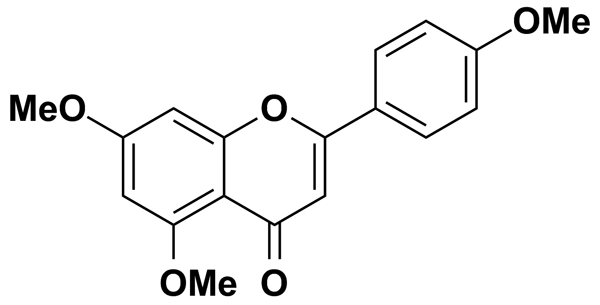 |
| 5,7-DICHLOROKYNURENIC ACID | 5.00 | Biomol International Inc. | BRD-K60287130 | 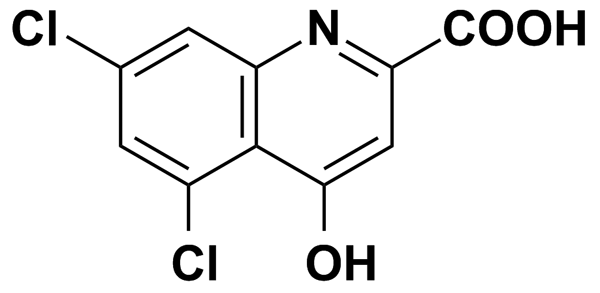 |
| 5,7-DIMETHOXYISOFLAVONE | 5.00 | MicroSource Discovery Systems Inc. | BRD-K41321810 | 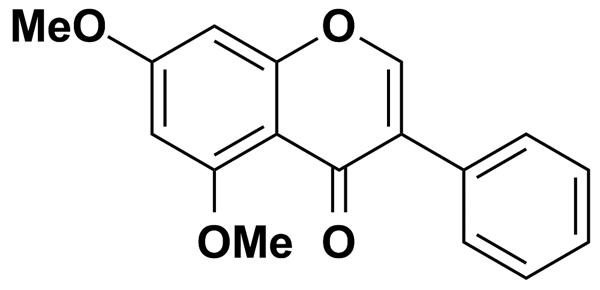 |
| 5-ALPHA-PREGNAN-3-ALPHA-OL-20-ONE | 5.00 | Biomol International Inc. | BRD-K18172896 | 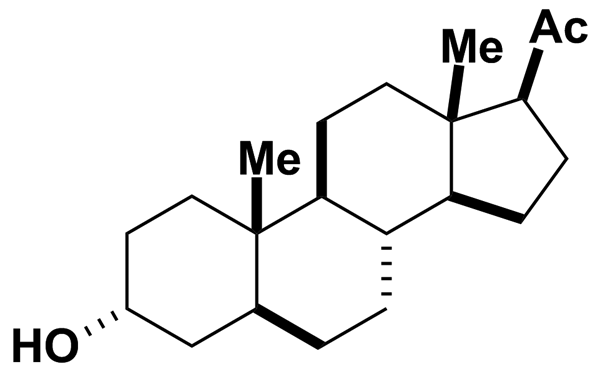 |
| 5-CHLOROINDOLE-2-CARBOXYLIC ACID | 5.00 | Biomol International Inc. | BRD-K57244822 | 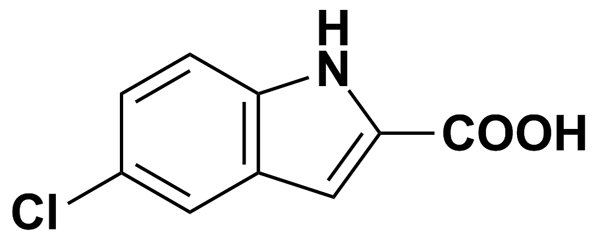 |
| 5-FLUOROINDOLE-2-CARBOXYLIC ACID | 5.00 | MicroSource Discovery Systems Inc. | BRD-K55273157 | 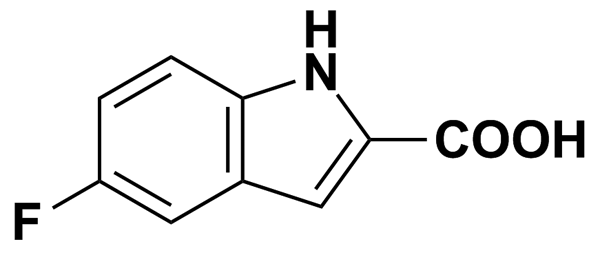 |
| 5-IODOTUBERCIDIN | 6.38 | Biomol International Inc. | BRD-A18497530 | 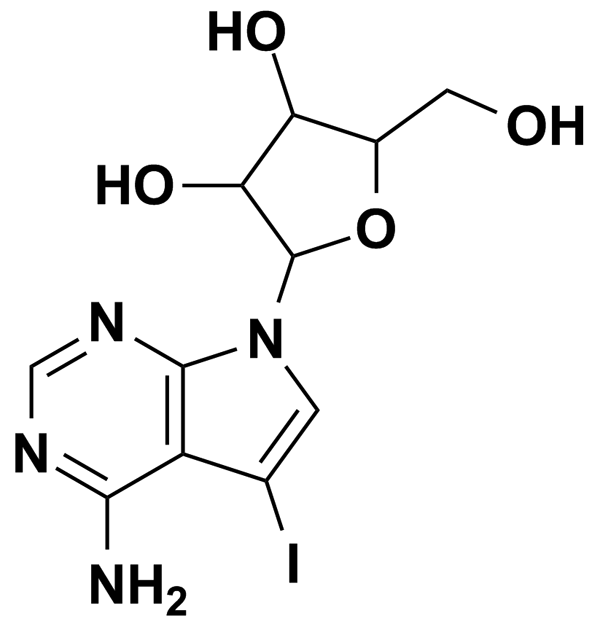 |
| 5-METHOXYTRYPTAMINE | 5.00 | Biomol International Inc. | BRD-K30197592 | 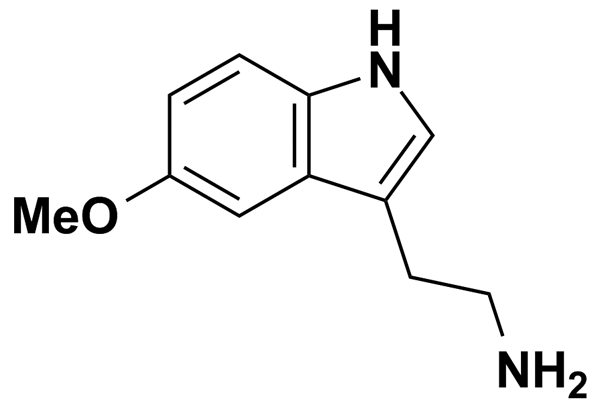 |
| 5-[(DIMETHYLAMINO)METHYL]-3-(1-METHYL-1H-INDOL-3-YL)-1,2,4-OXADIAZOLE | 5.00 | Biomol International Inc. | BRD-K80315422 | 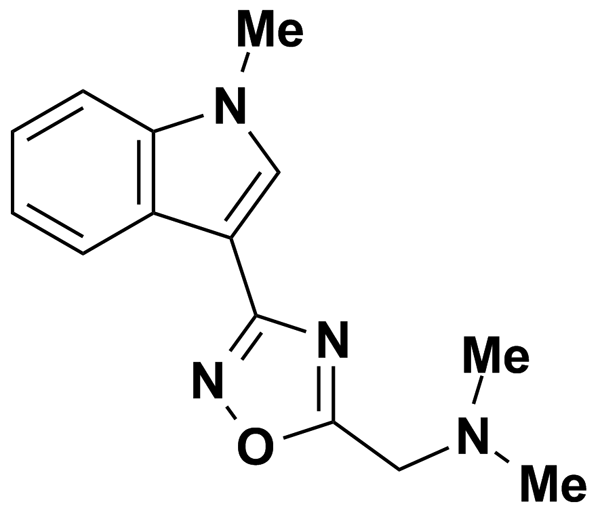 |
| 5BETA-12-METHOXY-4,4-BISNOR-8,11,13-PODOCARPATRIEN-3-ONE | 5.00 | MicroSource Discovery Systems Inc. | BRD-K26376097 | 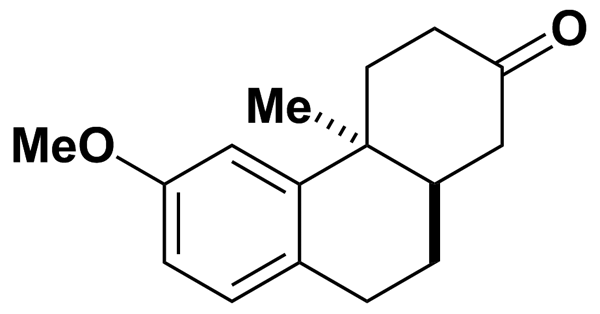 |
| 6,3'-DIMETHOXYFLAVONE | 5.00 | MicroSource Discovery Systems Inc. | BRD-K47724892 | 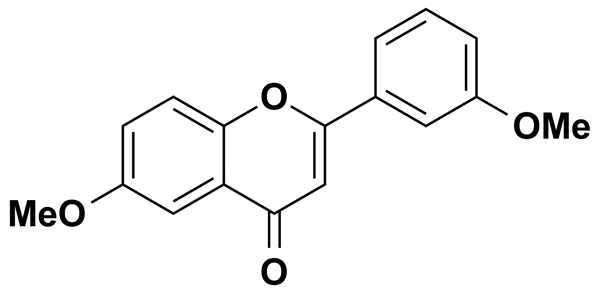 |
| 6,4'-DIMETHOXYFLAVONE | 5.00 | MicroSource Discovery Systems Inc. | BRD-K48629186 | 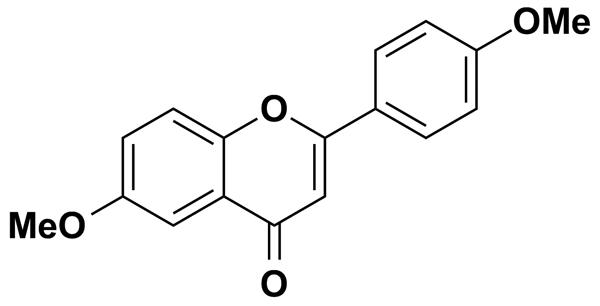 |
| 6,7-DINITROQUINOXALINE-2,3-DIONE | 5.00 | Biomol International Inc. | BRD-K64400208 |  |
| 6-CYANO-7-NITROQUINOXALINE-2,3-DIONE | 5.00 | Biomol International Inc. | BRD-K19438463 |  |
| 6-FORMYLINDOLO [3,2-B] CARBAZOLE | 0.50 | Biomol International Inc. | BRD-K00184207 |  |
| 6-FURFURYLAMINOPURINE | 4.65 | Prestwick Chemical Inc. | BRD-K65667145 |  |
| 6-HYDROXYANGOLENSIC ACID METHYL ESTER | 5.00 | MicroSource Discovery Systems Inc. | BRD-A15454510 |  |
| 6-NITROQUIPAZINE | 5.00 | Biomol International Inc. | BRD-K95821857 |  |
| 7,2'-DIMETHOXYFLAVONE | 5.00 | MicroSource Discovery Systems Inc. | BRD-K90574291 |  |
| 7,7-DIMETHYLEICOSADIENOIC ACID | 0.50 | Biomol International Inc. | BRD-K65207153 |  |
| 7,8-DIHYDROXYFLAVONE | 5.00 | MicroSource Discovery Systems Inc. | BRD-K49535716 |  |
| 7-CHLORO-3-METHYL-3,4-DIHYDRO-2H-1,2,4-BENZOTHIADIAZINE-S,S-DIOXIDE | 5.00 | Biomol International Inc. | BRD-A14344385 |  |
| 7-CHLOROETHYLTHEOPHYLLINE | 5.00 | MicroSource Discovery Systems Inc. | BRD-K52592505 |  |
| 7-CHLOROKYNURENIC ACID | 5.00 | Biomol International Inc. | BRD-K84214706 |  |
| 7-DEACETOXY-7-OXODEOXYGEDUNIN | 5.00 | MicroSource Discovery Systems Inc. | BRD-A37523642 |  |
| 7-DESACETOXY-6,7-DEHYDROGEDUNIN | 5.00 | MicroSource Discovery Systems Inc. | BRD-A97046705 |  |
| 7-HYDROXY-2'-METHOXYISOFLAVONE | 5.00 | MicroSource Discovery Systems Inc. | BRD-K09203354 |  |
| 7-HYDROXY-8,4'-DIMETHOXYISOFLAVONE | 5.00 | MicroSource Discovery Systems Inc. | BRD-K09100418 |  |
| 7-HYDROXY-DPAT | 5.00 | Biomol International Inc. | BRD-A18795974 |  |
| 7-METHOXYCHROMONE | 5.00 | MicroSource Discovery Systems Inc. | BRD-K48213854 |  |
| 7-NITROINDAZOLE | 5.00 | MicroSource Discovery Systems Inc. | BRD-K04430056 |  |
| 7-[2-TRIFLUOROMETHYL-4-(2-HYDROXYPHENYL)-1,3-DIOXAN-CIS-5-YL]-HEPT-5Z-ENOIC ACID | 5.00 | MicroSource Discovery Systems Inc. | BRD-K29133151 |  |
| 8-CYCLOPENTYLTHEOPHYLLINE | 5.00 | MicroSource Discovery Systems Inc. | BRD-K38347298 |  |
| 8-HYDROXY-15,16-BISNOR-11-LABDEN-13-ONE | 5.00 | MicroSource Discovery Systems Inc. | BRD-A61864215 |  |
| 8-HYDROXY-DPAT | 5.00 | Biomol International Inc. | BRD-A48015106 |  |
| 8-HYDROXYCARAPINIC ACID | 5.00 | MicroSource Discovery Systems Inc. | BRD-A73559168 |  |
| 8-IODOCATECHIN TETRAMETHYL ETHER | 5.00 | MicroSource Discovery Systems Inc. | BRD-K07396820 |  |
| 8-METHOXYMETHYL-IBMX | 9.39 | Biomol International Inc. | BRD-K56077740 |  |
| 8BETA-HYDROXYCARAPIN | 5.00 | MicroSource Discovery Systems Inc. | BRD-A56076240 |  |
| 8BETA-HYDROXYCARAPIN, 3,8-HEMIACETAL | 5.00 | MicroSource Discovery Systems Inc. | BRD-A17120244 |  |
| A 68930 | 5.00 | Biomol International Inc. | BRD-K33400588 |  |
| A-23187 | 4.77 | Biomol International Inc. | BRD-A19134330 |  |
| A-3 | 8.78 | Biomol International Inc. | BRD-K51215422 |  |
| A-77636 | 5.00 | Biomol International Inc. | BRD-K24396733 |  |
| ACACETIN | 3.52 | Prestwick Chemical Inc. | BRD-K77685744 |  |
| ACEBUTOLOL HYDROCHLORIDE | 2.68 | Prestwick Chemical Inc. | BRD-A29260609 |  |
| ACECAINIDE HYDROCHLORIDE | 5.00 | MicroSource Discovery Systems Inc. | BRD-K07753030 |  |
| ACECLIDINE HYDROCHLORIDE | 5.00 | MicroSource Discovery Systems Inc. | BRD-A32673558 |  |
| ACECLOFENAC | 2.82 | Prestwick Chemical Inc. | BRD-K68538666 |  |
| ACEMETACIN | 2.40 | Prestwick Chemical Inc. | BRD-K67563174 |  |
| ACENOCOUMAROL | 2.83 | Prestwick Chemical Inc. | BRD-A65051990 |  |
| ACETOCHLOR | 5.00 | MicroSource Discovery Systems Inc. | BRD-K16652251 |  |
| ACETOHEXAMIDE | 3.08 | Prestwick Chemical Inc. | BRD-K52960356 |  |
| ACETOSYRINGONE | 5.00 | MicroSource Discovery Systems Inc. | BRD-K66643401 |  |
| ACETYL (N)-S-FARNESYL-L-CYSTEINE | 6.80 | Biomol International Inc. | BRD-K79437791 |  |
| ACETYL TYROSINE ETHYL ESTER | 5.00 | MicroSource Discovery Systems Inc. | BRD-A55063801 |  |
| ACETYLSALICYLSALICYLIC ACID | 3.33 | Prestwick Chemical Inc. | BRD-K46585355 |  |
| ACETYLSEROTONIN | 5.00 | MicroSource Discovery Systems Inc. | BRD-K26140483 |  |
| ACETYLTRYPTOPHANAMIDE | 5.00 | MicroSource Discovery Systems Inc. | BRD-K10171338 |  |
| ACONITINE | 1.55 | Prestwick Chemical Inc. | BRD-A55025683 |  |
| ACONITINE | 3.87 | Biomol International Inc. | BRD-A00587958 |  |
| ACTINONIN | 5.00 | MicroSource Discovery Systems Inc. | BRD-K24621118 |  |
| ADIPHENINE HYDROCHLORIDE | 2.87 | Prestwick Chemical Inc. | BRD-K60907894 |  |
| ADRENIC ACID (22:4, N-6) | 0.50 | Biomol International Inc. | BRD-K17040651 |  |
| AESCULIN | 5.00 | MicroSource Discovery Systems Inc. | BRD-K51742987 |  |
| AG-1296 | 9.39 | Biomol International Inc. | BRD-K76064317 |  |
| AG-370 | 9.64 | Biomol International Inc. | BRD-K33204703 |  |
| AG-490 | 8.49 | Biomol International Inc. | BRD-K47105409 |  |
| AG-879 | 7.90 | Biomol International Inc. | BRD-K59469039 |  |
| AG1478 | 7.10 | Biomol International Inc. | BRD-K68336408 |  |
| AGC | 0.50 | Biomol International Inc. | BRD-K64729807 |  |
| AGGC | 0.50 | Biomol International Inc. | BRD-K75181824 |  |
| AJ-76 | 5.00 | Biomol International Inc. | BRD-K76677554 |  |
| AJMALICINE HYDROCHLORIDE | 2.57 | Prestwick Chemical Inc. | BRD-K83028735 |  |
| AJMALINE | 3.06 | Prestwick Chemical Inc. | BRD-A89846380 |  |
| ALA-ALA-PHE-CMK | 7.36 | Biomol International Inc. | BRD-K93080877 |  |
| ALACHLOR | 5.00 | MicroSource Discovery Systems Inc. | BRD-K02548315 |  |
| ALBENDAZOLE | 3.77 | Prestwick Chemical Inc. | BRD-K79131256 |  |
| ALCLOMETASONE DIPROPIONATE | 1.92 | Prestwick Chemical Inc. | BRD-A74610643 |  |
| ALCLOMETAZONE DIPROPIONATE | 5.00 | MicroSource Discovery Systems Inc. | BRD-A90131694 |  |
| ALDICARB | 5.00 | MicroSource Discovery Systems Inc. | BRD-K32595626 |  |
| ALFADOLONE ACETATE | 2.56 | Prestwick Chemical Inc. | BRD-K51751936 |  |
| ALFLUZOCIN | 5.00 | MicroSource Discovery Systems Inc. | BRD-A09056319 |  |
| ALFUZOSIN HYDROCHLORIDE | 2.35 | Prestwick Chemical Inc. | BRD-A09056319 |  |
| ALMOTRIPTAN | 5.00 | MicroSource Discovery Systems Inc. | BRD-K67601717 |  |
| ALPHA-CYANO-4-HYDROXYCINNAMIC ACID | 5.00 | MicroSource Discovery Systems Inc. | BRD-K60302405 |  |
| ALPHA-ERGOCRYPTINE | 5.00 | Biomol International Inc. | BRD-K74212935 |  |
| ALPHA-SANTONIN | 4.06 | Prestwick Chemical Inc. | BRD-K58787433 |  |
| ALPINETIN METHYL ETHER | 5.00 | MicroSource Discovery Systems Inc. | BRD-A90249268 |  |
| ALPRENOLOL HYDROCHLORIDE | 3.50 | Prestwick Chemical Inc. | BRD-A00993607 |  |
| ALTHIAZIDE | 2.60 | Prestwick Chemical Inc. | BRD-A56675431 |  |
| ALVERINE CITRATE SALT | 2.11 | Prestwick Chemical Inc. | BRD-K89055274 |  |
| AM 92016 | 5.59 | Biomol International Inc. | BRD-A11813248 |  |
| AM-251 | 0.50 | Biomol International Inc. | BRD-K92000912 |  |
| AM-580 | 0.50 | Biomol International Inc. | BRD-K06854232 |  |
| AMBROXOL HYDROCHLORIDE | 2.41 | Prestwick Chemical Inc. | BRD-K56558538 |  |
| AMCINONIDE | 5.00 | MicroSource Discovery Systems Inc. | BRD-A36010170 |  |
| AMI-193 | 5.00 | Biomol International Inc. | BRD-K94512704 |  |
| AMIDOPYRINE | 4.32 | Prestwick Chemical Inc. | BRD-K12568846 |  |
| AMILORIDE HYDROCHLORIDE DIHYDRATE | 3.31 | Prestwick Chemical Inc. | BRD-K97181089 |  |
| AMINACRINE | 5.00 | MicroSource Discovery Systems Inc. | BRD-K00535541 |  |
| AMINO-1,8-NAPHTHALIMIDE [4-AMINO-1,8-NAPHTHALIMIDE] | 11.78 | Biomol International Inc. | BRD-K50214219 |  |
| AMINOPURINE, 6-BENZYL | 4.44 | Prestwick Chemical Inc. | BRD-K62929068 |  |
| AMITRYPTILINE HYDROCHLORIDE | 3.19 | Prestwick Chemical Inc. | BRD-K53737926 |  |
| AMLODIPINE BESYLATE | 5.00 | MicroSource Discovery Systems Inc. | BRD-A22032524 |  |
| AMOXAPINE | 3.19 | Prestwick Chemical Inc. | BRD-K02265150 |  |
| AMOXICILLIN | 2.74 | Prestwick Chemical Inc. | BRD-K55044200 |  |
| AMPEROZIDE | 5.00 | Biomol International Inc. | BRD-K52397688 |  |
| AMYLEINE HYDROCHLORIDE | 3.68 | Prestwick Chemical Inc. | BRD-A09062839 |  |
| ANABASAMINE HYDROCHLORIDE | 5.00 | MicroSource Discovery Systems Inc. | BRD-A34774643 |  |
| ANABASINE | 6.16 | Prestwick Chemical Inc. | BRD-K36638830 |  |
| ANANDAMIDE (20:3,N-6) | 0.50 | Biomol International Inc. | BRD-K78280988 |  |
| ANANDAMIDE (20:4, N-6) | 0.50 | Biomol International Inc. | BRD-K42352790 |  |
| ANANDAMIDE (22:4,N-6) | 0.50 | Biomol International Inc. | BRD-K30199352 |  |
| ANDIROBIN | 5.00 | MicroSource Discovery Systems Inc. | BRD-A17500982 |  |
| ANDROSTA-1,4-DIEN-3,17-DIONE | 5.00 | MicroSource Discovery Systems Inc. | BRD-A95513702 |  |
| ANGOLENSIC ACID, METHYL ESTER | 5.00 | MicroSource Discovery Systems Inc. | BRD-A32349859 |  |
| ANGOLENSIN (R) | 5.00 | MicroSource Discovery Systems Inc. | BRD-K98281970 |  |
| ANHYDROBRAZILIC ACID | 5.00 | MicroSource Discovery Systems Inc. | BRD-K78414110 |  |
| ANIRACETAM | 5.00 | Biomol International Inc. | BRD-K88611939 |  |
| ANISODAMINE | 5.00 | MicroSource Discovery Systems Inc. | BRD-A25706161 |  |
| ANISOMYCIN | 9.42 | Biomol International Inc. | BRD-K91370081 |  |
| ANPIRTOLINE | 5.00 | Biomol International Inc. | BRD-K55424922 |  |
| ANTAZOLINE HYDROCHLORIDE | 3.31 | Prestwick Chemical Inc. | BRD-K99300445 |  |
| ANTIAROL | 5.00 | MicroSource Discovery Systems Inc. | BRD-K28698314 |  |
| ANTIPYRINE | 5.31 | Prestwick Chemical Inc. | BRD-K46937689 |  |
| ANTIPYRINE, 4-HYDROXY | 4.90 | Prestwick Chemical Inc. | BRD-K49759007 |  |
| APIIN | 5.00 | MicroSource Discovery Systems Inc. | BRD-K52379519 |  |
| ARACHIDONAMIDE | 0.50 | Biomol International Inc. | BRD-K29555132 |  |
| ARACHIDONIC ACID (20:4, N-6) | 0.50 | Biomol International Inc. | BRD-K03070961 |  |
| ARECOLINE HYDROBROMIDE | 4.24 | Prestwick Chemical Inc. | BRD-K88646909 |  |
| ARTICAINE HYDROCHLORIDE | 3.12 | Prestwick Chemical Inc. | BRD-A70514680 |  |
| ARVANIL | 5.69 | Biomol International Inc. | BRD-K34092021 |  |
| ASARYLALDEHYDE | 5.00 | MicroSource Discovery Systems Inc. | BRD-K88219015 |  |
| ASCOMYCIN (FK-520) | 3.16 | Biomol International Inc. | BRD-K88998544 |  |
| ASIATICOSIDE | 1.04 | Prestwick Chemical Inc. | BRD-A66199457 |  |
| ASTEMIZOLE | 2.18 | Prestwick Chemical Inc. | BRD-K37249724 |  |
| ATENOLOL | 5.00 | MicroSource Discovery Systems Inc. | BRD-A20239487 |  |
| ATOVAQUONE | 2.73 | Prestwick Chemical Inc. | BRD-A84493640 |  |
| ATOVAQUONE | 5.00 | MicroSource Discovery Systems Inc. | BRD-A19795905 |  |
| ATRAZINE | 5.00 | MicroSource Discovery Systems Inc. | BRD-K45535217 |  |
| AUSTRICINE | 5.00 | MicroSource Discovery Systems Inc. | BRD-K78838262 |  |
| AUSTRICINE HYDRATE | 3.57 | Prestwick Chemical Inc. | BRD-K64236792 |  |
| AVERMECTIN B1 | 5.00 | MicroSource Discovery Systems Inc. | BRD-A84689408 |  |
| AZACYCLONOL | 3.74 | Prestwick Chemical Inc. | BRD-K97061094 |  |
| AZAPERONE | 3.05 | Prestwick Chemical Inc. | BRD-K45861246 |  |
| AZATHIOPRINE | 3.61 | Prestwick Chemical Inc. | BRD-K32821942 |  |
| AZINPHOS METHYL | 5.00 | MicroSource Discovery Systems Inc. | BRD-K21893543 |  |
| AZLOCILLIN SODIUM SALT | 2.06 | Prestwick Chemical Inc. | BRD-K60663764 |  |
| AZTREONAM | 2.30 | Prestwick Chemical Inc. | BRD-K62607865 |  |
| B-HT 920 | 5.00 | Biomol International Inc. | BRD-K75615183 |  |
| B581 | 5.31 | Biomol International Inc. | BRD-K04877770 |  |
| BACAMPICILLIN HYDROCHLORIDE | 1.99 | Prestwick Chemical Inc. | BRD-A41698174 |  |
| BAFILOMYCIN A1 | 0.40 | Biomol International Inc. | BRD-K74038771 |  |
| BAMBUTEROL HYDROCHLORIDE | 2.48 | Prestwick Chemical Inc. | BRD-A17462676 |  |
| BAPTA-AM | 3.27 | Biomol International Inc. | BRD-K40919711 |  |
| BAY 11-7082 | 12.06 | Biomol International Inc. | BRD-K15025317 |  |
| BAY K-8644 | 7.02 | Biomol International Inc. | BRD-A05457250 |  |
| BENAZEPRIL HYDROCHLORIDE | 5.00 | MicroSource Discovery Systems Inc. | BRD-K49807096 |  |
| BENDROFLUMETHIAZIDE | 2.37 | Prestwick Chemical Inc. | BRD-A80017228 |  |
| BENFLUOREX HYDROCHLORIDE | 2.58 | Prestwick Chemical Inc. | BRD-A22305049 |  |
| BENOXINATE HYDROCHLORIDE | 2.90 | Prestwick Chemical Inc. | BRD-K04185004 |  |
| BENPERIDOL | 2.62 | Prestwick Chemical Inc. | BRD-K79425933 |  |
| BENTAZON | 5.00 | MicroSource Discovery Systems Inc. | BRD-K33986892 |  |
| BENZAMIL | 7.82 | Biomol International Inc. | BRD-K97688263 |  |
| BENZAMIL HYDROCHLORIDE | 2.81 | Prestwick Chemical Inc. | BRD-K97688263 |  |
| BENZANTHRONE | 5.00 | MicroSource Discovery Systems Inc. | BRD-K76872913 |  |
| BENZBROMARONE | 2.36 | Prestwick Chemical Inc. | BRD-K11717138 |  |
| BENZOCAINE | 6.05 | Prestwick Chemical Inc. | BRD-K75466013 |  |
| BENZO[A]PYRENE | 5.00 | MicroSource Discovery Systems Inc. | BRD-K09668667 |  |
| BENZYDAMINE HYDROCHLORIDE | 2.89 | Prestwick Chemical Inc. | BRD-K28542495 |  |
| BENZYL PENICILLIN POTASSIUM | 5.00 | MicroSource Discovery Systems Inc. | BRD-K55191674 |  |
| BENZYLBUTYLPHTHALATE | 5.00 | MicroSource Discovery Systems Inc. | BRD-K34359596 |  |
| BENZYLPENICILLIN SODIUM | 2.80 | Prestwick Chemical Inc. | BRD-K55191674 |  |
| BERBERINE CHLORIDE | 2.68 | Prestwick Chemical Inc. | BRD-K14796088 |  |
| BERGAPTEN | 5.00 | MicroSource Discovery Systems Inc. | BRD-K12968785 |  |
| BERGAPTOL | 5.00 | MicroSource Discovery Systems Inc. | BRD-K36383661 |  |
| BERGENIN | 5.00 | MicroSource Discovery Systems Inc. | BRD-K18550767 |  |
| BESTATIN | 8.11 | Biomol International Inc. | BRD-K59574735 |  |
| BETA-DIHYDROROTENONE | 5.00 | MicroSource Discovery Systems Inc. | BRD-K53672524 |  |
| BETAHISTINE HYDROCHLORIDE | 5.00 | MicroSource Discovery Systems Inc. | BRD-K91315211 |  |
| BETAHISTINE MESYLATE | 3.05 | Prestwick Chemical Inc. | BRD-K91315211 |  |
| BETAMETHASONE | 2.55 | Prestwick Chemical Inc. | BRD-K39188321 |  |
| BETAMETHASONE 17,21-DIPROPIONATE | 5.00 | MicroSource Discovery Systems Inc. | BRD-A66861218 |  |
| BETAMETHASONE VALERATE | 5.00 | MicroSource Discovery Systems Inc. | BRD-A80594172 |  |
| BETAMIPRON | 5.00 | MicroSource Discovery Systems Inc. | BRD-K82142815 |  |
| BETAXOLOL HYDROCHLORIDE | 2.91 | Prestwick Chemical Inc. | BRD-A02759312 |  |
| BETULINIC ACID | 5.47 | Biomol International Inc. | BRD-K45401373 |  |
| BEZAFIBRATE | 6.91 | Biomol International Inc. | BRD-K46018455 |  |
| BIFONAZOLE | 5.00 | MicroSource Discovery Systems Inc. | BRD-A94543220 |  |
| BIOCHANIN A | 5.00 | MicroSource Discovery Systems Inc. | BRD-K73303757 |  |
| BIOTIN | 4.09 | Prestwick Chemical Inc. | BRD-K89210380 |  |
| BIPERIDEN HYDROCHLORIDE | 2.87 | Prestwick Chemical Inc. | BRD-A36471396 |  |
| BISACODYL | 2.77 | Prestwick Chemical Inc. | BRD-K39987650 |  |
| BLEBBISTATIN | 8.55 | Biomol International Inc. | BRD-A75817871 |  |
| BML-190 | 0.50 | Biomol International Inc. | BRD-K94379058 |  |
| BML-257 | 5.00 | Biomol International Inc. | BRD-K32584078 |  |
| BML-259 | 5.00 | Biomol International Inc. | BRD-K71799778 |  |
| BOLDINE | 3.05 | Prestwick Chemical Inc. | BRD-K03440695 |  |
| BOLDINE | 5.00 | MicroSource Discovery Systems Inc. | BRD-A44966919 |  |
| BONGKREKIC ACID | 0.51 | Biomol International Inc. | BRD-A41301928 |  |
| BP 554 | 5.00 | Biomol International Inc. | BRD-K45479396 |  |
| BREFELDIN A | 8.92 | Biomol International Inc. | BRD-A13416979 |  |
| BRINZOLAMIDE | 2.61 | Prestwick Chemical Inc. | BRD-K74913225 |  |
| BRL 15572 | 5.00 | Biomol International Inc. | BRD-A51829654 |  |
| BROMHEXINE HYDROCHLORIDE | 5.00 | MicroSource Discovery Systems Inc. | BRD-K47631482 |  |
| BROMO-7-NITROINDAZOLE [3-BROMO-7-NITROINDAZOLE] | 10.33 | Biomol International Inc. | BRD-K24689407 |  |
| BROMOCRIPTINE | 5.00 | Biomol International Inc. | BRD-K14496212 |  |
| BROMOCRIPTINE MESYLATE | 5.00 | MicroSource Discovery Systems Inc. | BRD-A80151636 |  |
| BROMOCRYPTINE MESYLATE | 1.33 | Prestwick Chemical Inc. | BRD-A60274948 |  |
| BROMOPRIDE | 2.90 | Prestwick Chemical Inc. | BRD-K73642618 |  |
| BROMPERIDOL | 2.38 | Prestwick Chemical Inc. | BRD-K78643075 |  |
| BROMPHENIRAMINE MALEATE | 2.30 | Prestwick Chemical Inc. | BRD-A68723818 |  |
| BRUCINE | 5.00 | MicroSource Discovery Systems Inc. | BRD-A41995253 |  |
| BUDDLEOFLAVONOLOSIDE | 5.00 | MicroSource Discovery Systems Inc. | BRD-K25905511 |  |
| BUDESONIDE | 2.32 | Prestwick Chemical Inc. | BRD-A34299591 |  |
| BUFLOMEDIL HYDROCHLORIDE | 2.91 | Prestwick Chemical Inc. | BRD-K19462402 |  |
| BUMETANIDE | 6.86 | Biomol International Inc. | BRD-K38197229 |  |
| BUPIVACAINE HYDROCHLORIDE | 3.08 | Prestwick Chemical Inc. | BRD-A01636364 |  |
| BUPROPION HYDROCHLORIDE | 3.62 | Prestwick Chemical Inc. | BRD-A05186015 |  |
| BUSPIRONE | 5.00 | Biomol International Inc. | BRD-K93461745 |  |
| BUSPIRONE HYDROCHLORIDE | 2.37 | Prestwick Chemical Inc. | BRD-K93461745 |  |
| BUTAMBEN | 5.17 | Prestwick Chemical Inc. | BRD-K27217864 |  |
| BUTOCONAZOLE NITRATE | 2.11 | Prestwick Chemical Inc. | BRD-A16665823 |  |
| BUTYL-BETA-CARBOLINE-3-CARBOXYLATE | 5.00 | Biomol International Inc. | BRD-K87349602 |  |
| BW-B 70C | 7.90 | Biomol International Inc. | BRD-A55946879 |  |
| C2 CERAMIDE | 0.50 | Biomol International Inc. | BRD-K41707108 |  |
| CA-074-ME | 6.29 | Biomol International Inc. | BRD-A56020723 |  |
| CABERGOLINE | 5.00 | Biomol International Inc. | BRD-K86882815 |  |
| CAFFEINE | 5.00 | MicroSource Discovery Systems Inc. | BRD-K02404261 |  |
| CALPHOSTIN C | 0.32 | Biomol International Inc. | BRD-K63793181 |  |
| CALYCANTHINE | 2.89 | Prestwick Chemical Inc. | BRD-K59851896 |  |
| CALYCULIN A | 0.25 | Biomol International Inc. | BRD-A47513740 |  |
| CAMPTOTHECIN | 7.18 | Biomol International Inc. | BRD-K57055976 |  |
| CANDESARTAN CILEXTIL | 5.00 | MicroSource Discovery Systems Inc. | BRD-A65671304 |  |
| CANRENOIC ACID POTASSIUM SALT | 2.52 | Prestwick Chemical Inc. | BRD-K46556543 |  |
| CANRENOIC ACID, POTASSIUM SALT | 5.00 | MicroSource Discovery Systems Inc. | BRD-A29322418 |  |
| CAPE | 8.79 | Biomol International Inc. | BRD-K96188950 |  |
| CAPSAZEPINE | 6.63 | Biomol International Inc. | BRD-K44849676 |  |
| CAPTOPRIL | 4.60 | Prestwick Chemical Inc. | BRD-K54529596 |  |
| CARBACYCLIN | 0.50 | Biomol International Inc. | BRD-K27499107 |  |
| CARBAMAZEPINE | 4.23 | Prestwick Chemical Inc. | BRD-K71799949 |  |
| CARBETAPENTANE CITRATE | 1.90 | Prestwick Chemical Inc. | BRD-K06181161 |  |
| CARBIMAZOLE | 5.37 | Prestwick Chemical Inc. | BRD-K87156652 |  |
| CARBINOXAMINE MALEATE SALT | 2.46 | Prestwick Chemical Inc. | BRD-A29426959 |  |
| CARTEOLOL HYDROCHLORIDE | 3.04 | Prestwick Chemical Inc. | BRD-A42167015 |  |
| CARVEDILOL TARTRATE | 5.00 | MicroSource Discovery Systems Inc. | BRD-A10977446 |  |
| CATECHIN PENTABENZOATE | 5.00 | MicroSource Discovery Systems Inc. | BRD-K03600606 |  |
| CDC | 7.78 | Biomol International Inc. | BRD-K10870738 |  |
| CEDRELONE | 5.00 | MicroSource Discovery Systems Inc. | BRD-A69143712 |  |
| CEFACLOR | 2.59 | Prestwick Chemical Inc. | BRD-K20338176 |  |
| CEFAMANDOLE SODIUM SALT | 2.06 | Prestwick Chemical Inc. | BRD-K27130738 |  |
| CEFDITORIN PIVOXIL | 5.00 | MicroSource Discovery Systems Inc. | BRD-A39056513 |  |
| CEFIXIME | 2.21 | Prestwick Chemical Inc. | BRD-K71059170 |  |
| CEFOPERAZONE DIHYDRATE | 1.47 | Prestwick Chemical Inc. | BRD-K02292852 |  |
| CEFORANIDE | 1.92 | Prestwick Chemical Inc. | BRD-K37848908 |  |
| CEFOTAXIME SODIUM | 5.00 | MicroSource Discovery Systems Inc. | BRD-K99586414 |  |
| CEFOTAXIME SODIUM SALT | 2.09 | Prestwick Chemical Inc. | BRD-K78364995 |  |
| CEFOTIAM HYDROCHLORIDE | 1.78 | Prestwick Chemical Inc. | BRD-K02275692 |  |
| CEFUROXIME SODIUM SALT | 2.24 | Prestwick Chemical Inc. | BRD-K02733959 |  |
| CELECOXIB | 5.00 | MicroSource Discovery Systems Inc. | BRD-K02637541 |  |
| CEPHAELINE DIHYDROCHLORIDE HEPTAHYDRATE | 1.50 | Prestwick Chemical Inc. | BRD-K80348542 |  |
| CEPHALEXIN MONOHYDRATE | 2.74 | Prestwick Chemical Inc. | BRD-K90733503 |  |
| CEPHALOTHIN SODIUM SALT | 2.38 | Prestwick Chemical Inc. | BRD-K28210218 |  |
| CERULENIN | 11.20 | Biomol International Inc. | BRD-K52075040 |  |
| CETIRIZINE DIHYDROCHLORIDE | 2.17 | Prestwick Chemical Inc. | BRD-A42571354 |  |
| CGP 52432 | 5.00 | Biomol International Inc. | BRD-A04668240 |  |
| CGP 54626 | 5.00 | Biomol International Inc. | BRD-A74208568 |  |
| CGP 55845 | 5.00 | Biomol International Inc. | BRD-A89672324 |  |
| CGP-37157 | 7.71 | Biomol International Inc. | BRD-A35623999 |  |
| CHELERYTHRINE | 7.18 | Biomol International Inc. | BRD-K87904882 |  |
| CHELIDONINE (+) | 2.69 | Prestwick Chemical Inc. | BRD-K32828673 |  |
| CHELIDONINE (+) | 5.00 | MicroSource Discovery Systems Inc. | BRD-A19527356 |  |
| CHLORAMBUCIL | 3.29 | Prestwick Chemical Inc. | BRD-K29458283 |  |
| CHLORAMPHENICOL | 3.09 | Prestwick Chemical Inc. | BRD-K08111712 |  |
| CHLORAMPHENICOL | 5.00 | MicroSource Discovery Systems Inc. | BRD-K22969690 |  |
| CHLORMETHIAZOLE | 5.00 | Biomol International Inc. | BRD-K50422030 |  |
| CHLORMEZANONE | 5.00 | Biomol International Inc. | BRD-A20348246 |  |
| CHLOROGENIC ACID | 2.82 | Prestwick Chemical Inc. | BRD-K47114202 |  |
| CHLOROXYLENOL | 5.00 | MicroSource Discovery Systems Inc. | BRD-K17223896 |  |
| CHLORPHENIRAMINE (S) MALEATE | 5.00 | MicroSource Discovery Systems Inc. | BRD-K89595132 |  |
| CHLORPHENIRAMINE MALEATE | 2.56 | Prestwick Chemical Inc. | BRD-A04553218 |  |
| CHLORPHENSIN CARBAMATE | 4.07 | Prestwick Chemical Inc. | BRD-A39230911 |  |
| CHLORPROPAMIDE | 3.61 | Prestwick Chemical Inc. | BRD-K97746869 |  |
| CHLORPROPHAM | 5.00 | MicroSource Discovery Systems Inc. | BRD-K59590127 |  |
| CHLORPROTHIXENE HYDROCHLORIDE | 2.84 | Prestwick Chemical Inc. | BRD-K59058766 |  |
| CHLORPYRIFOS | 5.00 | MicroSource Discovery Systems Inc. | BRD-K08303368 |  |
| CHLORTHALIDONE | 2.95 | Prestwick Chemical Inc. | BRD-A26384407 |  |
| CHLORZOXAZONE | 5.90 | Prestwick Chemical Inc. | BRD-K98174813 |  |
| CHROMOCARB | 5.00 | MicroSource Discovery Systems Inc. | BRD-K94720315 |  |
| CHRYSANTHEMIC ACID | 5.00 | MicroSource Discovery Systems Inc. | BRD-A52893269 |  |
| CHRYSIN DIMETHYL ETHER | 5.00 | MicroSource Discovery Systems Inc. | BRD-K81298036 |  |
| CHRYSOPHANOL | 5.00 | MicroSource Discovery Systems Inc. | BRD-K59284035 |  |
| CIGLITAZONE | 0.50 | Biomol International Inc. | BRD-A93000692 |  |
| CIMATEROL | 11.40 | Biomol International Inc. | BRD-A65440446 |  |
| CIMETIDINE | 3.96 | Prestwick Chemical Inc. | BRD-K34157611 |  |
| CINANSERIN | 5.00 | Biomol International Inc. | BRD-K40901640 |  |
| CINCHONINE | 3.40 | Prestwick Chemical Inc. | BRD-K90268819 |  |
| CINCHONINE | 5.00 | MicroSource Discovery Systems Inc. | BRD-K18476269 |  |
| CINNARAZINE | 5.00 | MicroSource Discovery Systems Inc. | BRD-K32256916 |  |
| CINNARIZINE | 2.71 | Prestwick Chemical Inc. | BRD-K07220430 |  |
| CINOXACIN | 3.81 | Prestwick Chemical Inc. | BRD-K14704277 |  |
| CIPROFIBRATE | 3.46 | Prestwick Chemical Inc. | BRD-A49358627 |  |
| CIPROFLOXACIN HYDROCHLORIDE | 2.59 | Prestwick Chemical Inc. | BRD-K04804440 |  |
| CIRAZOLINE | 11.56 | Biomol International Inc. | BRD-K54142781 |  |
| CIS-(Z)-FLUPENTHIXOL | 5.00 | Biomol International Inc. | BRD-K70487031 |  |
| CISAPRIDE | 2.15 | Prestwick Chemical Inc. | BRD-K59163477 |  |
| CITROPTEN | 5.00 | MicroSource Discovery Systems Inc. | BRD-K78612426 |  |
| CL 218872 | 5.00 | Biomol International Inc. | BRD-K00662280 |  |
| CLEBOPRIDE MALEATE | 2.04 | Prestwick Chemical Inc. | BRD-K17294426 |  |
| CLEMASTINE FUMARATE | 2.17 | Prestwick Chemical Inc. | BRD-K30240666 |  |
| CLEMIZOLE HYDROCHLORIDE | 2.76 | Prestwick Chemical Inc. | BRD-K04704168 |  |
| CLENBUTEROL HYDROCHLORIDE | 3.19 | Prestwick Chemical Inc. | BRD-A75726477 |  |
| CLINDAMYCIN HYDROCHLORIDE | 2.17 | Prestwick Chemical Inc. | BRD-A23034328 |  |
| CLOBETASOL PROPIONATE | 2.14 | Prestwick Chemical Inc. | BRD-A63894585 |  |
| CLOBETASOL PROPIONATE | 5.00 | MicroSource Discovery Systems Inc. | BRD-A26095496 |  |
| CLOCORTOLONE PIVALATE | 2.02 | Prestwick Chemical Inc. | BRD-K38003476 |  |
| CLOFIBRIC ACID | 4.66 | Prestwick Chemical Inc. | BRD-K19111024 |  |
| CLOMIPHENE CITRATE | 5.00 | MicroSource Discovery Systems Inc. | BRD-K29950728 |  |
| CLOMIPHENE CITRATE (Z,E) | 1.67 | Prestwick Chemical Inc. | BRD-K04218075 |  |
| CLOMIPRAMINE | 5.00 | Biomol International Inc. | BRD-K52989797 |  |
| CLOMIPRAMINE HYDROCHLORIDE | 2.85 | Prestwick Chemical Inc. | BRD-K52989797 |  |
| CLONIDINE | 10.87 | Biomol International Inc. | BRD-K98530306 |  |
| CLONIDINE HYDROCHLORIDE | 3.75 | Prestwick Chemical Inc. | BRD-K98530306 |  |
| CLOPAMIDE | 2.89 | Prestwick Chemical Inc. | BRD-K15567837 |  |
| CLOPAMIDE | 5.00 | MicroSource Discovery Systems Inc. | BRD-A85651701 |  |
| CLOPERASTINE HYDROCHLORIDE | 2.73 | Prestwick Chemical Inc. | BRD-A80908310 |  |
| CLOPIDOGREL SULFATE | 5.00 | MicroSource Discovery Systems Inc. | BRD-K27721098 |  |
| CLOPROSTENOL | 0.50 | Biomol International Inc. | BRD-K17850764 |  |
| CLORGYLINE HYDROCHLORIDE | 3.24 | Prestwick Chemical Inc. | BRD-K73251053 |  |
| CLORSULON | 2.63 | Prestwick Chemical Inc. | BRD-K97521363 |  |
| CLOTHIAPINE | 5.00 | Biomol International Inc. | BRD-K10990317 |  |
| CLOXACILLIN SODIUM SALT | 2.18 | Prestwick Chemical Inc. | BRD-K01244426 |  |
| CLOXYQUIN | 5.00 | MicroSource Discovery Systems Inc. | BRD-K46982791 |  |
| CLOZAPINE | 5.00 | Biomol International Inc. | BRD-K37289225 |  |
| COLCHICINE | 2.50 | Prestwick Chemical Inc. | BRD-K00259736 |  |
| COMPACTIN | 5.00 | MicroSource Discovery Systems Inc. | BRD-A83177971 |  |
| CONDELPHINE | 2.22 | Prestwick Chemical Inc. | BRD-A84941884 |  |
| CONVOLAMINE HYDROCHLORIDE | 2.93 | Prestwick Chemical Inc. | BRD-K67016489 |  |
| CORALYNE CHLORIDE HYDRATE | 2.39 | Prestwick Chemical Inc. | BRD-K24443173 |  |
| CORTICOSTERONE | 2.89 | Prestwick Chemical Inc. | BRD-K73589401 |  |
| CORTISONE | 2.77 | Prestwick Chemical Inc. | BRD-K43736954 |  |
| CORTISONE | 5.00 | MicroSource Discovery Systems Inc. | BRD-A62731508 |  |
| CORYNANTHINE HYDROCHLORIDE | 2.56 | Prestwick Chemical Inc. | BRD-K06467078 |  |
| COSMOSIIN HEXAACETATE | 5.00 | MicroSource Discovery Systems Inc. | BRD-K34844053 |  |
| COTININE (-) | 5.67 | Prestwick Chemical Inc. | BRD-K94144010 |  |
| COUMARIN | 5.00 | MicroSource Discovery Systems Inc. | BRD-K23913458 |  |
| COUMOPHOS | 5.00 | MicroSource Discovery Systems Inc. | BRD-K41567533 |  |
| CRASSIN ACETATE | 5.00 | MicroSource Discovery Systems Inc. | BRD-A92593740 |  |
| CRINAMINE | 5.00 | MicroSource Discovery Systems Inc. | BRD-A08786049 |  |
| CRUSTECDYSONE | 5.00 | MicroSource Discovery Systems Inc. | BRD-A46778020 |  |
| CURCUMIN | 5.00 | MicroSource Discovery Systems Inc. | BRD-K07572174 |  |
| CURCUMIN | 6.79 | Biomol International Inc. | BRD-K74148702 |  |
| CYANOPINDOLOL | 5.00 | Biomol International Inc. | BRD-K40965114 |  |
| CYCLACILLIN | 2.93 | Prestwick Chemical Inc. | BRD-K89046952 |  |
| CYCLIZINE | 5.00 | MicroSource Discovery Systems Inc. | BRD-K79501723 |  |
| CYCLIZINE HYDROCHLORIDE | 3.30 | Prestwick Chemical Inc. | BRD-K79501723 |  |
| CYCLOBENZAPRINE HYDROCHLORIDE | 3.21 | Prestwick Chemical Inc. | BRD-K42348709 |  |
| CYCLOHEXIMIDE | 3.55 | Prestwick Chemical Inc. | BRD-K36055864 |  |
| CYCLOHEXIMIDE | 8.89 | Biomol International Inc. | BRD-A95580639 |  |
| CYCLOHEXIMIDE-N-ETHYLETHANOATE | 6.80 | Biomol International Inc. | BRD-A82329351 |  |
| CYCLOPAMINE | 6.07 | Biomol International Inc. | BRD-K58938839 |  |
| CYCLOPENTHIAZIDE | 2.63 | Prestwick Chemical Inc. | BRD-A41250306 |  |
| CYCLOPENTOLATE HYDROCHLORIDE | 3.05 | Prestwick Chemical Inc. | BRD-A77291778 |  |
| CYCLOPIAZONIC ACID | 5.00 | MicroSource Discovery Systems Inc. | BRD-A26787626 |  |
| CYCLOPIAZONIC ACID | 7.43 | Biomol International Inc. | BRD-A10420615 |  |
| CYCLOSPORIN A | 0.83 | Prestwick Chemical Inc. | BRD-K13533483 |  |
| CYCLOSPORIN A | 2.08 | Biomol International Inc. | BRD-A69815203 |  |
| CYCLOTHIAZIDE | 5.00 | Biomol International Inc. | BRD-A38675539 |  |
| CYCLOVERATRYLENE | 5.00 | MicroSource Discovery Systems Inc. | BRD-K49154891 |  |
| CYPROHEPTADINE | 5.00 | Biomol International Inc. | BRD-K28143534 |  |
| CYPROHEPTADINE HYDROCHLORIDE | 3.09 | Prestwick Chemical Inc. | BRD-K28143534 |  |
| CYPROTERONE ACETATE | 2.40 | Prestwick Chemical Inc. | BRD-A39290993 |  |
| CYPROTERONE ACETATE | 5.00 | MicroSource Discovery Systems Inc. | BRD-A95207036 |  |
| CYTISINE | 5.00 | MicroSource Discovery Systems Inc. | BRD-A55579717 |  |
| CYTISINE (-) | 5.26 | Prestwick Chemical Inc. | BRD-K74186897 |  |
| CYTOCHALASIN B | 5.21 | Biomol International Inc. | BRD-K02157199 |  |
| CYTOCHALASIN D | 4.92 | Biomol International Inc. | BRD-K16135852 |  |
| DACTINOMYCIN | 5.00 | MicroSource Discovery Systems Inc. | BRD-A04541874 |  |
| DAIDZEIN | 5.00 | Biomol International Inc. | BRD-K42095107 |  |
| DANAZOL | 2.96 | Prestwick Chemical Inc. | BRD-K48970916 |  |
| DANAZOL | 5.00 | MicroSource Discovery Systems Inc. | BRD-A92537424 |  |
| DANTROLENE | 7.96 | Biomol International Inc. | BRD-K81272440 |  |
| DANTROLENE SODIUM SALT | 2.97 | Prestwick Chemical Inc. | BRD-K81272440 |  |
| DANTRON | 5.00 | MicroSource Discovery Systems Inc. | BRD-K10065684 |  |
| DAPHNETIN | 5.00 | MicroSource Discovery Systems Inc. | BRD-K61269089 |  |
| DAPSONE | 4.03 | Prestwick Chemical Inc. | BRD-K62363391 |  |
| DAUNORUBICIN HYDROCHLORIDE | 1.77 | Prestwick Chemical Inc. | BRD-K43389675 |  |
| DEACETOXY-7-OXISOGEDUNIN | 5.00 | MicroSource Discovery Systems Inc. | BRD-A17819071 |  |
| DEACETOXY-7-OXOGEDUNIN | 5.00 | MicroSource Discovery Systems Inc. | BRD-A25408073 |  |
| DEACETYLGEDUNIN | 5.00 | MicroSource Discovery Systems Inc. | BRD-A06561405 |  |
| DEFEROXAMINE MESYLATE | 1.52 | Prestwick Chemical Inc. | BRD-K09821361 |  |
| DEGUELIN(-) | 5.00 | MicroSource Discovery Systems Inc. | BRD-K61401890 |  |
| DEHYDRO (11,12)URSOLIC ACID LACTONE | 5.00 | MicroSource Discovery Systems Inc. | BRD-A98888138 |  |
| DEHYDROCHOLIC ACID | 2.48 | Prestwick Chemical Inc. | BRD-K90976994 |  |
| DEHYDRODIHYDROROTENONE | 5.00 | MicroSource Discovery Systems Inc. | BRD-A33119430 |  |
| DEHYDROROTENONE | 5.00 | MicroSource Discovery Systems Inc. | BRD-A84913188 |  |
| DELTALINE | 1.97 | Prestwick Chemical Inc. | BRD-A99177642 |  |
| DEMECLOCYCLINE HYDROCHLORIDE | 1.99 | Prestwick Chemical Inc. | BRD-A34145622 |  |
| DEOXODEOXYDIHYDROGEDUNIN | 5.00 | MicroSource Discovery Systems Inc. | BRD-A98490861 |  |
| DEOXYANDIROBIN | 5.00 | MicroSource Discovery Systems Inc. | BRD-A02799884 |  |
| DEOXYANDIROBIN LACTONE | 5.00 | MicroSource Discovery Systems Inc. | BRD-A58269583 |  |
| DEOXYGEDUNOL ACETATE | 5.00 | MicroSource Discovery Systems Inc. | BRD-A71611160 |  |
| DEOXYKHIVORIN | 5.00 | MicroSource Discovery Systems Inc. | BRD-A39660642 |  |
| DEOXYSAPPANONE B 7,3'-DIMETHYL ETHER ACETATE | 5.00 | MicroSource Discovery Systems Inc. | BRD-A20368188 |  |
| DEOXYSAPPANONE B 7,4'-DIMETHYL ETHER | 5.00 | MicroSource Discovery Systems Inc. | BRD-A46460777 |  |
| DEOXYSAPPANONE B TRIMETHYL ETHER | 5.00 | MicroSource Discovery Systems Inc. | BRD-A77164655 |  |
| DEPRENYL | 13.35 | Biomol International Inc. | BRD-K86434416 |  |
| DERACOXIB | 5.00 | MicroSource Discovery Systems Inc. | BRD-K68558722 |  |
| DERRUBONE | 5.00 | MicroSource Discovery Systems Inc. | BRD-K59272984 |  |
| DERRUSNIN | 5.00 | MicroSource Discovery Systems Inc. | BRD-K72066653 |  |
| DERRUSTONE | 5.00 | MicroSource Discovery Systems Inc. | BRD-K72135530 |  |
| DESACETYL (7)KHIVORINIC ACID, METHYL ESTER | 5.00 | MicroSource Discovery Systems Inc. | BRD-A24436948 |  |
| DESOXYPEGANINE HYDROCHLORIDE | 5.00 | MicroSource Discovery Systems Inc. | BRD-K13819402 |  |
| DEXAMETHASONE | 6.37 | Biomol International Inc. | BRD-K38775274 |  |
| DEXAMETHASONE ACETATE | 2.30 | Prestwick Chemical Inc. | BRD-K47635719 |  |
| DEXAMETHASONE ACETATE | 5.00 | MicroSource Discovery Systems Inc. | BRD-A93424738 |  |
| DEXTROMETHORPHAN HYDROBROMIDE | 5.00 | MicroSource Discovery Systems Inc. | BRD-K24053527 |  |
| DEXTROMETHORPHAN HYDROBROMIDE MONOHYDRATE | 2.70 | Prestwick Chemical Inc. | BRD-K33211335 |  |
| DIAZEPAM | 5.00 | MicroSource Discovery Systems Inc. | BRD-K16508793 |  |
| DIAZOXIDE | 10.84 | Biomol International Inc. | BRD-K73109821 |  |
| DIBENZOYLMETHANE | 5.00 | MicroSource Discovery Systems Inc. | BRD-K01555864 |  |
| DIBUCAINE | 2.91 | Prestwick Chemical Inc. | BRD-K99121711 |  |
| DIBUCAINE HYDROCHLORIDE | 5.00 | MicroSource Discovery Systems Inc. | BRD-K99121711 |  |
| DIBUTYL PHTHALATE | 5.00 | MicroSource Discovery Systems Inc. | BRD-K73477617 |  |
| DIBUTYRYLCYCLIC AMP | 5.08 | Biomol International Inc. | BRD-A94624445 |  |
| DIBUTYRYLCYCLIC GMP | 5.15 | Biomol International Inc. | BRD-A06726973 |  |
| DICHLOBENIL | 5.00 | MicroSource Discovery Systems Inc. | BRD-K43714461 |  |
| DICHLOROBENZAMIL | 6.43 | Biomol International Inc. | BRD-K12906962 |  |
| DICHLORODIPHENYLTRICHLOROETHANE | 5.00 | MicroSource Discovery Systems Inc. | BRD-K51799616 |  |
| DICLOFENAC SODIUM | 3.13 | Prestwick Chemical Inc. | BRD-K08252256 |  |
| DICLOXACILLIN SODIUM SALT | 1.96 | Prestwick Chemical Inc. | BRD-K05673000 |  |
| DICUMAROL | 2.97 | Prestwick Chemical Inc. | BRD-K82236179 |  |
| DIENESTROL | 3.75 | Prestwick Chemical Inc. | BRD-K95309561 |  |
| DIETHYLCARBAMAZINE CITRATE | 2.55 | Prestwick Chemical Inc. | BRD-K45542189 |  |
| DIFFRACTIC ACID | 5.00 | MicroSource Discovery Systems Inc. | BRD-K51370144 |  |
| DIFLORASONE DIACETATE | 2.02 | Prestwick Chemical Inc. | BRD-K17674993 |  |
| DIFLUNISAL | 4.00 | Prestwick Chemical Inc. | BRD-K22031190 |  |
| DIFUCOL HEXAMETHYL ETHER | 5.00 | MicroSource Discovery Systems Inc. | BRD-K64246525 |  |
| DIGITOXIGENIN | 2.67 | Prestwick Chemical Inc. | BRD-K18518344 |  |
| DIGITOXIN | 5.00 | MicroSource Discovery Systems Inc. | BRD-A93236127 |  |
| DIGOXIN | 1.28 | Prestwick Chemical Inc. | BRD-K23478508 |  |
| DIGOXIN | 5.00 | MicroSource Discovery Systems Inc. | BRD-A94756469 |  |
| DIHYDRO-BETA-TUBAIC ACID | 5.00 | MicroSource Discovery Systems Inc. | BRD-K96362535 |  |
| DIHYDROERGOCRISTINE | 5.00 | Biomol International Inc. | BRD-A65076780 |  |
| DIHYDROERGOCRISTINE MESYLATE | 1.41 | Prestwick Chemical Inc. | BRD-K97440753 |  |
| DIHYDROERGOTAMINE MESYLATE | 5.00 | MicroSource Discovery Systems Inc. | BRD-K72166146 |  |
| DIHYDROSAMIDIN | 5.00 | MicroSource Discovery Systems Inc. | BRD-K63945320 |  |
| DIINDOLYLMETHANE | 0.50 | Biomol International Inc. | BRD-K37846922 |  |
| DILAZEP DIHYDROCHLORIDE | 1.48 | Prestwick Chemical Inc. | BRD-K48722258 |  |
| DILTIAZEM | 6.03 | Biomol International Inc. | BRD-K24023109 |  |
| DILTIAZEM HYDROCHLORIDE | 2.22 | Prestwick Chemical Inc. | BRD-K24023109 |  |
| DILTIAZEM HYDROCHLORIDE | 5.00 | MicroSource Discovery Systems Inc. | BRD-A69636825 |  |
| DIMETHISOQUIN HYDROCHLORIDE | 3.24 | Prestwick Chemical Inc. | BRD-K73391359 |  |
| DIMETHOATE | 5.00 | MicroSource Discovery Systems Inc. | BRD-K94763113 |  |
| DIMETHYL 4,4-O-PHENYLENE-BIS (3-THIOPHANATE) | 5.00 | MicroSource Discovery Systems Inc. | BRD-K90168339 |  |
| DIOSMETIN | 5.00 | MicroSource Discovery Systems Inc. | BRD-K26862302 |  |
| DIOXYBENZONE | 4.09 | Prestwick Chemical Inc. | BRD-K22193694 |  |
| DIPERODON HYDROCHLORIDE | 2.30 | Prestwick Chemical Inc. | BRD-A30693873 |  |
| DIPHEMANIL METHYLSULFATE | 2.56 | Prestwick Chemical Inc. | BRD-K93441486 |  |
| DIPHENHYDRAMINE HYDROCHLORIDE | 3.43 | Prestwick Chemical Inc. | BRD-K47278471 |  |
| DIPHENIDOL HYDROCHLORIDE | 2.89 | Prestwick Chemical Inc. | BRD-K01663662 |  |
| DIPHENYLENEIODONIUM | 8.96 | Biomol International Inc. | BRD-K65814004 |  |
| DIPHENYLPYRALINE HYDROCHLORIDE | 3.15 | Prestwick Chemical Inc. | BRD-K22936972 |  |
| DIPIVEFRIN HYDROCHLORIDE | 2.58 | Prestwick Chemical Inc. | BRD-A47494775 |  |
| DIPROPHYLLINE | 3.93 | Prestwick Chemical Inc. | BRD-A00827783 |  |
| DIPROPYLDOPAMINE | 5.00 | Biomol International Inc. | BRD-K82577285 |  |
| DIPYROCETYL | 5.00 | MicroSource Discovery Systems Inc. | BRD-K47608922 |  |
| DIPYRONE | 2.99 | Prestwick Chemical Inc. | BRD-K76812510 |  |
| DISOPYRAMIDE | 2.95 | Prestwick Chemical Inc. | BRD-A29734509 |  |
| DISOPYRAMIDE PHOSPHATE | 5.00 | MicroSource Discovery Systems Inc. | BRD-A29734509 |  |
| DISULFIRAM | 3.37 | Prestwick Chemical Inc. | BRD-K32744045 |  |
| DIURON | 5.00 | MicroSource Discovery Systems Inc. | BRD-K75330923 |  |
| DIZOCILPINE MALEATE | 2.96 | Prestwick Chemical Inc. | BRD-K58930050 |  |
| DL-PDMP | 0.50 | Biomol International Inc. | BRD-K05653692 |  |
| DO 897/99 | 2.04 | Prestwick Chemical Inc. | BRD-K17378184 |  |
| DOBUTAMINE HYDROCHLORIDE | 2.96 | Prestwick Chemical Inc. | BRD-A78322124 |  |
| DOCOSAHEXAENOIC ACID (22:6 N-3) | 0.50 | Biomol International Inc. | BRD-K39965020 |  |
| DOCOSATRIENOIC ACID (22:3 N-3) | 0.50 | Biomol International Inc. | BRD-K01624546 |  |
| DOMPERIDONE | 5.00 | Biomol International Inc. | BRD-K38305202 |  |
| DOSULEPIN HYDROCHLORIDE | 3.01 | Prestwick Chemical Inc. | BRD-K54759182 |  |
| DOXEPIN HYDROCHLORIDE | 3.17 | Prestwick Chemical Inc. | BRD-K54462405 |  |
| DOXEPIN HYDROCHLORIDE | 5.00 | MicroSource Discovery Systems Inc. | BRD-K37694030 |  |
| DOXYLAMINE SUCCINATE | 2.57 | Prestwick Chemical Inc. | BRD-A44008656 |  |
| DRB | 7.83 | Biomol International Inc. | BRD-A74904029 |  |
| DROFENINE HYDROCHLORIDE | 2.83 | Prestwick Chemical Inc. | BRD-A22267103 |  |
| DROPERIDOL | 2.64 | Prestwick Chemical Inc. | BRD-K97158071 |  |
| DROPROPIZINE (R,S) | 4.23 | Prestwick Chemical Inc. | BRD-A29349577 |  |
| DUARTIN (-) | 5.00 | MicroSource Discovery Systems Inc. | BRD-K47814830 |  |
| DUARTIN, DIMETHYL ETHER | 5.00 | MicroSource Discovery Systems Inc. | BRD-K87798455 |  |
| DUBINIDINE | 3.63 | Prestwick Chemical Inc. | BRD-A99571536 |  |
| DYCLONINE HYDROCHLORIDE | 3.07 | Prestwick Chemical Inc. | BRD-K72259270 |  |
| DYDROGESTERONE | 3.20 | Prestwick Chemical Inc. | BRD-K68620903 |  |
| E-4031 | 6.23 | Biomol International Inc. | BRD-K41713976 |  |
| EBSELEN | 9.12 | Biomol International Inc. | BRD-K29359156 |  |
| EBURNAMONINE (-) | 3.40 | Prestwick Chemical Inc. | BRD-K40227168 |  |
| ECONAZOLE NITRATE | 2.25 | Prestwick Chemical Inc. | BRD-A51820102 |  |
| EHNA | 9.01 | Biomol International Inc. | BRD-K27450477 |  |
| EICOSAPENTAENOIC ACID (20:5 N-3) | 0.50 | Biomol International Inc. | BRD-K47192521 |  |
| ELLIPTICINE | 4.06 | Prestwick Chemical Inc. | BRD-K85985071 |  |
| EMETINE DIHYDROCHLORIDE | 1.81 | Prestwick Chemical Inc. | BRD-K03067624 |  |
| EMODIC ACID | 5.00 | MicroSource Discovery Systems Inc. | BRD-K94841585 |  |
| EMODIN | 5.00 | MicroSource Discovery Systems Inc. | BRD-K58685305 |  |
| ENDECAPHYLLIN X | 5.00 | MicroSource Discovery Systems Inc. | BRD-K54728231 |  |
| ENILCONAZOLE | 3.36 | Prestwick Chemical Inc. | BRD-A11776908 |  |
| ENOXACIN | 3.12 | Prestwick Chemical Inc. | BRD-K78113049 |  |
| ENOXOLONE | 5.00 | MicroSource Discovery Systems Inc. | BRD-A20200672 |  |
| EPHEDRINE (1R,2S) HYDROCHLORIDE | 5.00 | MicroSource Discovery Systems Inc. | BRD-A98431941 |  |
| EPIAFZELECHIN (2R,3R)(-) | 5.00 | MicroSource Discovery Systems Inc. | BRD-K90607708 |  |
| EPIBATIDINE (+/-) | 11.98 | Biomol International Inc. | BRD-A39268308 |  |
| EPICATECHIN PENTAACETATE | 5.00 | MicroSource Discovery Systems Inc. | BRD-K93674274 |  |
| EPICATECHIN-(-) | 3.45 | Prestwick Chemical Inc. | BRD-K58736316 |  |
| EPIRIZOLE | 4.27 | Prestwick Chemical Inc. | BRD-K39339537 |  |
| EPIVINCAMINE | 2.82 | Prestwick Chemical Inc. | BRD-K89704198 |  |
| EPOXYGEDUNIN | 5.00 | MicroSource Discovery Systems Inc. | BRD-A06232236 |  |
| ERGOCORNINE | 5.00 | Biomol International Inc. | BRD-K19360254 |  |
| ERGONOVINE | 5.00 | Biomol International Inc. | BRD-A73581086 |  |
| ERGONOVINE MALEATE | 5.00 | MicroSource Discovery Systems Inc. | BRD-A90550479 |  |
| ESCITALOPRAM OXALATE | 5.00 | MicroSource Discovery Systems Inc. | BRD-K70301876 |  |
| ESTRADIOL | 5.00 | MicroSource Discovery Systems Inc. | BRD-A18917088 |  |
| ESTRADIOL | 9.18 | Biomol International Inc. | BRD-K18910433 |  |
| ESTRADIOL ACETATE | 5.00 | MicroSource Discovery Systems Inc. | BRD-A23226398 |  |
| ESTRADIOL BENZOATE | 5.00 | MicroSource Discovery Systems Inc. | BRD-A36066264 |  |
| ESTRADIOL CYPIONATE | 5.00 | MicroSource Discovery Systems Inc. | BRD-A91452556 |  |
| ESTRADIOL DIACETATE | 5.00 | MicroSource Discovery Systems Inc. | BRD-A33614871 |  |
| ESTRADIOL METHYL ETHER | 5.00 | MicroSource Discovery Systems Inc. | BRD-A83613346 |  |
| ESTRADIOL VALERATE | 5.00 | MicroSource Discovery Systems Inc. | BRD-A39747742 |  |
| ESTRADIOL-3-SULFATE, SODIUM SALT | 5.00 | MicroSource Discovery Systems Inc. | BRD-A91702150 |  |
| ESTRIOL | 3.47 | Prestwick Chemical Inc. | BRD-K17016787 |  |
| ESTRIOL | 5.00 | MicroSource Discovery Systems Inc. | BRD-A18620900 |  |
| ESTRIOL METHYL ETHER | 5.00 | MicroSource Discovery Systems Inc. | BRD-A84205515 |  |
| ESTRONE | 3.70 | Prestwick Chemical Inc. | BRD-K81839095 |  |
| ESTRONE | 5.00 | MicroSource Discovery Systems Inc. | BRD-A37959677 |  |
| ESTRONE BENZOATE | 5.00 | MicroSource Discovery Systems Inc. | BRD-A90969585 |  |
| ESTROPIPATE | 2.29 | Prestwick Chemical Inc. | BRD-K51095933 |  |
| ESTROPIPATE | 5.00 | MicroSource Discovery Systems Inc. | BRD-A22143024 |  |
| ETHACRYNIC ACID | 3.30 | Prestwick Chemical Inc. | BRD-K63630713 |  |
| ETHAMIVAN | 4.48 | Prestwick Chemical Inc. | BRD-K38055836 |  |
| ETHINYL ESTRADIOL | 5.00 | MicroSource Discovery Systems Inc. | BRD-A02367930 |  |
| ETHION | 5.00 | MicroSource Discovery Systems Inc. | BRD-K84268861 |  |
| ETHIONAMIDE | 6.02 | Prestwick Chemical Inc. | BRD-K33710385 |  |
| ETHISTERONE | 3.20 | Prestwick Chemical Inc. | BRD-K03981224 |  |
| ETHOPROP | 5.00 | MicroSource Discovery Systems Inc. | BRD-K08556791 |  |
| ETHOSUXIMIDE | 7.08 | Prestwick Chemical Inc. | BRD-A99633051 |  |
| ETHOXYQUIN | 4.60 | Prestwick Chemical Inc. | BRD-K56792340 |  |
| ETHYL 1-BENZYL-3-HYDROXY- 2-OXO[5H]PYRROLE-4-CARBOXYLATE | 5.00 | MicroSource Discovery Systems Inc. | BRD-A96485169 |  |
| ETHYL-BETA-CARBOLINE-3-CARBOXYLATE | 5.00 | Biomol International Inc. | BRD-K49061529 |  |
| ETILEFRINE HYDROCHLORIDE | 4.59 | Prestwick Chemical Inc. | BRD-A09925278 |  |
| ETODOLAC | 3.48 | Prestwick Chemical Inc. | BRD-A74667430 |  |
| ETOFYLLINE | 4.46 | Prestwick Chemical Inc. | BRD-K83064458 |  |
| ETOPOSIDE | 1.70 | Prestwick Chemical Inc. | BRD-K37798499 |  |
| ETOPOSIDE | 4.25 | Biomol International Inc. | BRD-A79067928 |  |
| ETOPOSIDE | 5.00 | MicroSource Discovery Systems Inc. | BRD-A33280134 |  |
| EUCATROPINE HYDROCHLORIDE | 3.05 | Prestwick Chemical Inc. | BRD-A37942872 |  |
| EUCATROPINE HYDROCHLORIDE | 5.00 | MicroSource Discovery Systems Inc. | BRD-A69786436 |  |
| EUGENITOL | 5.00 | MicroSource Discovery Systems Inc. | BRD-K46068882 |  |
| EUPARIN | 5.00 | MicroSource Discovery Systems Inc. | BRD-K26383086 |  |
| EUPATORIOCHROMENE | 5.00 | MicroSource Discovery Systems Inc. | BRD-K55704455 |  |
| EVOXINE | 2.88 | Prestwick Chemical Inc. | BRD-K47693913 |  |
| EVOXINE | 5.00 | MicroSource Discovery Systems Inc. | BRD-A99268498 |  |
| EZETIMIBE | 5.00 | MicroSource Discovery Systems Inc. | BRD-A41519720 |  |
| FAMCICLOVIR | 5.00 | MicroSource Discovery Systems Inc. | BRD-K45033733 |  |
| FAMOTIDINE | 2.96 | Prestwick Chemical Inc. | BRD-K00673382 |  |
| FAMPROFAZONE | 2.65 | Prestwick Chemical Inc. | BRD-A08255417 |  |
| FARNESYLTHIOACETIC ACID | 0.50 | Biomol International Inc. | BRD-K63089472 |  |
| FCCP | 9.84 | Biomol International Inc. | BRD-K14821540 |  |
| FELAMIDIN | 5.00 | MicroSource Discovery Systems Inc. | BRD-K36638198 |  |
| FELBAMATE | 5.00 | Biomol International Inc. | BRD-K99107520 |  |
| FELODIPINE | 2.60 | Prestwick Chemical Inc. | BRD-A30815329 |  |
| FENAMISAL | 5.00 | MicroSource Discovery Systems Inc. | BRD-K73157543 |  |
| FENBENDAZOLE | 3.34 | Prestwick Chemical Inc. | BRD-K51318897 |  |
| FENBUFEN | 3.93 | Prestwick Chemical Inc. | BRD-K12513978 |  |
| FENBUTYRAMIDE | 5.00 | MicroSource Discovery Systems Inc. | BRD-A25537246 |  |
| FENDILINE HYDROCHLORIDE | 2.84 | Prestwick Chemical Inc. | BRD-A71033472 |  |
| FENOFIBRATE | 2.77 | Prestwick Chemical Inc. | BRD-K50388907 |  |
| FENOLDOPAM | 5.00 | Biomol International Inc. | BRD-A50684349 |  |
| FENOTEROL HYDROBROMIDE | 2.60 | Prestwick Chemical Inc. | BRD-A97104540 |  |
| FENSPIRIDE HYDROCHLORIDE | 3.37 | Prestwick Chemical Inc. | BRD-K26739552 |  |
| FENTHION | 5.00 | MicroSource Discovery Systems Inc. | BRD-K67217586 |  |
| FEXOFENADINE | 5.00 | MicroSource Discovery Systems Inc. | BRD-A73368467 |  |
| FG 7142 | 5.00 | Biomol International Inc. | BRD-K61951118 |  |
| FGIN-1-27 | 5.00 | Biomol International Inc. | BRD-K09778810 |  |
| FILLALBIN | 3.43 | Prestwick Chemical Inc. | BRD-K28667196 |  |
| FINASTERIDE | 2.68 | Prestwick Chemical Inc. | BRD-K01095011 |  |
| FIPEXIDE HYDROCHLORIDE | 2.35 | Prestwick Chemical Inc. | BRD-K37688416 |  |
| FIPRONIL | 5.72 | Biomol International Inc. | BRD-A50675702 |  |
| FISSINOLIDE | 5.00 | MicroSource Discovery Systems Inc. | BRD-A29371732 |  |
| FLAVANONE | 5.00 | MicroSource Discovery Systems Inc. | BRD-A07824748 |  |
| FLAVOKAWAIN B | 5.00 | MicroSource Discovery Systems Inc. | BRD-K30296925 |  |
| FLAVOXATE HYDROCHLORIDE | 2.34 | Prestwick Chemical Inc. | BRD-K47639036 |  |
| FLECAINIDE | 6.03 | Biomol International Inc. | BRD-A09472452 |  |
| FLECAINIDE ACETATE | 2.11 | Prestwick Chemical Inc. | BRD-A09472452 |  |
| FLOPROPIONE | 5.00 | MicroSource Discovery Systems Inc. | BRD-K43383936 |  |
| FLUCLOXACILLIN SODIUM | 2.10 | Prestwick Chemical Inc. | BRD-A13650332 |  |
| FLUCONAZOLE | 5.00 | MicroSource Discovery Systems Inc. | BRD-K05977355 |  |
| FLUDROCORTISONE ACETATE | 5.00 | MicroSource Discovery Systems Inc. | BRD-A38749782 |  |
| FLUFENAMIC ACID | 8.89 | Biomol International Inc. | BRD-K44067360 |  |
| FLUMAZENIL | 5.00 | Biomol International Inc. | BRD-K98769987 |  |
| FLUMEQUINE | 3.83 | Prestwick Chemical Inc. | BRD-A69777949 |  |
| FLUMETHASONE | 2.44 | Prestwick Chemical Inc. | BRD-K61496577 |  |
| FLUNARIZINE | 6.18 | Biomol International Inc. | BRD-K29582677 |  |
| FLUNARIZINE DIHYDROCHLORIDE | 2.09 | Prestwick Chemical Inc. | BRD-K29582677 |  |
| FLUNISOLIDE | 5.00 | MicroSource Discovery Systems Inc. | BRD-A65449987 |  |
| FLUNIXIN MEGLUMINE | 2.03 | Prestwick Chemical Inc. | BRD-K99984802 |  |
| FLUNIXIN MEGLUMINE | 5.00 | MicroSource Discovery Systems Inc. | BRD-K99984802 |  |
| FLUOCINONIDE | 2.02 | Prestwick Chemical Inc. | BRD-K57886322 |  |
| FLUOCINONIDE | 5.00 | MicroSource Discovery Systems Inc. | BRD-A15297126 |  |
| FLUOROCURARINE CHLORIDE | 2.91 | Prestwick Chemical Inc. | BRD-A64280577 |  |
| FLUOROMETHOLONE | 2.66 | Prestwick Chemical Inc. | BRD-K64862097 |  |
| FLUOROMETHOLONE | 5.00 | MicroSource Discovery Systems Inc. | BRD-A13133631 |  |
| FLUOXETINE | 5.00 | Biomol International Inc. | BRD-A31159102 |  |
| FLUOXETINE HYDROCHLORIDE | 2.89 | Prestwick Chemical Inc. | BRD-A31159102 |  |
| FLUPERLAPINE | 5.00 | Biomol International Inc. | BRD-K15715913 |  |
| FLUPHENAZINE | 5.00 | Biomol International Inc. | BRD-K55127134 |  |
| FLUPHENAZINE DIHYDROCHLORIDE | 1.96 | Prestwick Chemical Inc. | BRD-K55127134 |  |
| FLUPROSTENOL | 0.50 | Biomol International Inc. | BRD-K31611373 |  |
| FLURANDRENOLIDE | 2.29 | Prestwick Chemical Inc. | BRD-K00824317 |  |
| FLURANDRENOLIDE | 5.00 | MicroSource Discovery Systems Inc. | BRD-A49765801 |  |
| FLUSPIRILINE | 5.26 | Biomol International Inc. | BRD-K77947974 |  |
| FLUTAMIDE | 3.62 | Prestwick Chemical Inc. | BRD-K28307902 |  |
| FLUTICASONE PROPIONATE | 2.00 | Prestwick Chemical Inc. | BRD-K62310379 |  |
| FLUVOXAMINE MALEATE | 2.30 | Prestwick Chemical Inc. | BRD-K53517854 |  |
| FOLIOSIDINE | 3.25 | Prestwick Chemical Inc. | BRD-A49734948 |  |
| FORMONONETIN | 5.00 | MicroSource Discovery Systems Inc. | BRD-K55567017 |  |
| FPL-64176 | 7.20 | Biomol International Inc. | BRD-K78959463 |  |
| FRAXETIN | 5.00 | MicroSource Discovery Systems Inc. | BRD-K76587808 |  |
| FURALTADONE HYDROCHLORIDE | 2.77 | Prestwick Chemical Inc. | BRD-A67514145 |  |
| FURAZOLIDONE | 5.00 | MicroSource Discovery Systems Inc. | BRD-K11756522 |  |
| FUREGRELATE SODIUM | 5.00 | MicroSource Discovery Systems Inc. | BRD-K55529781 |  |
| FUROSEMIDE | 3.02 | Prestwick Chemical Inc. | BRD-K78010432 |  |
| FURSULTIAMINE HYDROCHLORIDE | 2.30 | Prestwick Chemical Inc. | BRD-A71157293 |  |
| FUSARIC ACID | 5.58 | Prestwick Chemical Inc. | BRD-K87049188 |  |
| FUSIDIC ACID | 5.00 | MicroSource Discovery Systems Inc. | BRD-A06935312 |  |
| FUSIDIC ACID SODIUM SALT | 1.85 | Prestwick Chemical Inc. | BRD-A78085235 |  |
| GABAZINE | 2.71 | Prestwick Chemical Inc. | BRD-K93280214 |  |
| GABAZINE | 5.00 | Biomol International Inc. | BRD-K79905821 |  |
| GABEXATE MESILATE | 2.40 | Prestwick Chemical Inc. | BRD-K59256312 |  |
| GALANTHAMINE HYDROBROMIDE | 2.72 | Prestwick Chemical Inc. | BRD-K49481516 |  |
| GAMMA-LINOLENIC ACID (18:3 N-6) | 0.50 | Biomol International Inc. | BRD-K18059238 |  |
| GATIFLOXACIN | 5.00 | MicroSource Discovery Systems Inc. | BRD-A74980173 |  |
| GBR 12909 DIHYDROCHLORIDE | 1.91 | Prestwick Chemical Inc. | BRD-K32501161 |  |
| GBR 12935 | 5.00 | Biomol International Inc. | BRD-K50135270 |  |
| GBR 13069 | 5.00 | Biomol International Inc. | BRD-K92577649 |  |
| GBR-12909 | 5.00 | Biomol International Inc. | BRD-K32501161 |  |
| GEDUNIN | 5.00 | MicroSource Discovery Systems Inc. | BRD-A48397526 |  |
| GELSEMINE | 3.10 | Prestwick Chemical Inc. | BRD-A17535965 |  |
| GEMFIBROZIL | 3.99 | Prestwick Chemical Inc. | BRD-K11129031 |  |
| GEMIFLOXACIN MESYLATE | 5.00 | MicroSource Discovery Systems Inc. | BRD-A40787240 |  |
| GENISTEIN | 5.00 | MicroSource Discovery Systems Inc. | BRD-K43797669 |  |
| GENKWANIN | 5.00 | MicroSource Discovery Systems Inc. | BRD-K29160894 |  |
| GENTIAN VIOLET | 5.00 | MicroSource Discovery Systems Inc. | BRD-K60025295 |  |
| GF-109203X | 6.06 | Biomol International Inc. | BRD-K31342827 |  |
| GIBBERELLIC ACID | 2.89 | Prestwick Chemical Inc. | BRD-K92758126 |  |
| GINGEROL | 8.49 | Biomol International Inc. | BRD-K26117720 |  |
| GINKGETIN, K SALT | 5.00 | MicroSource Discovery Systems Inc. | BRD-K92123432 |  |
| GITOXIGENIN | 5.00 | MicroSource Discovery Systems Inc. | BRD-A29082194 |  |
| GITOXIN | 5.00 | MicroSource Discovery Systems Inc. | BRD-A33810106 |  |
| GLAFENINE HYDROCHLORIDE | 2.44 | Prestwick Chemical Inc. | BRD-A38076815 |  |
| GLICLAZIDE | 3.09 | Prestwick Chemical Inc. | BRD-A61154809 |  |
| GLIMEPIRIDE | 2.04 | Prestwick Chemical Inc. | BRD-K34776109 |  |
| GLIPIZIDE | 5.61 | Biomol International Inc. | BRD-K12219985 |  |
| GLIQUIDONE | 1.90 | Prestwick Chemical Inc. | BRD-K80396088 |  |
| GLUTETHIMIDE, PARA-AMINO | 4.31 | Prestwick Chemical Inc. | BRD-A25234499 |  |
| GLYBURIDE | 5.06 | Biomol International Inc. | BRD-K36927236 |  |
| GLYCOCHOLIC ACID | 2.15 | Prestwick Chemical Inc. | BRD-K54771420 |  |
| GO6976 | 6.61 | Biomol International Inc. | BRD-K59304176 |  |
| GOSSYPOL | 1.93 | Prestwick Chemical Inc. | BRD-K19295594 |  |
| GOSSYPOL-ACETIC ACID COMPLEX | 5.00 | MicroSource Discovery Systems Inc. | BRD-K19295594 |  |
| GR 103691 | 5.00 | Biomol International Inc. | BRD-K50891186 |  |
| GR 55562 | 5.00 | Biomol International Inc. | BRD-K46441700 |  |
| GRAMINE | 5.74 | Prestwick Chemical Inc. | BRD-K26005076 |  |
| GRAVEOLINE | 3.58 | Prestwick Chemical Inc. | BRD-K92683369 |  |
| GRISEOFULVIN | 2.83 | Prestwick Chemical Inc. | BRD-K08273968 |  |
| GUAIFENESIN | 5.05 | Prestwick Chemical Inc. | BRD-A90515964 |  |
| GUANABENZ ACETATE | 5.00 | MicroSource Discovery Systems Inc. | BRD-K56800335 |  |
| GUANFACINE HYDROCHLORIDE | 3.54 | Prestwick Chemical Inc. | BRD-K32830106 |  |
| GW-9662 | 9.04 | Biomol International Inc. | BRD-K93258693 |  |
| H-89 | 5.60 | Biomol International Inc. | BRD-K27737647 |  |
| H7 | 8.58 | Biomol International Inc. | BRD-A55756846 |  |
| HA-1004 | 8.52 | Biomol International Inc. | BRD-K05434375 |  |
| HAEMATOMMIC ACID | 5.00 | MicroSource Discovery Systems Inc. | BRD-K03463894 |  |
| HALCINONIDE | 2.20 | Prestwick Chemical Inc. | BRD-K81709173 |  |
| HALOPERIDOL | 5.00 | Biomol International Inc. | BRD-K67783091 |  |
| HARMALINE | 5.00 | MicroSource Discovery Systems Inc. | BRD-K91317041 |  |
| HARMALINE HYDROCHLORIDE DIHYDRATE | 3.49 | Prestwick Chemical Inc. | BRD-K91317041 |  |
| HARMALOL HYDROCHLORIDE | 5.00 | MicroSource Discovery Systems Inc. | BRD-K14756138 |  |
| HARMALOL HYDROCHLORIDE DIHYDRATE | 3.67 | Prestwick Chemical Inc. | BRD-K14756138 |  |
| HARMANE HYDROCHLORIDE | 4.57 | Prestwick Chemical Inc. | BRD-K12630395 |  |
| HARMINE HYDROCHLORIDE | 4.02 | Prestwick Chemical Inc. | BRD-K30984264 |  |
| HARMOL HYDROCHLORIDE MONOHYDRATE | 3.96 | Prestwick Chemical Inc. | BRD-K01225247 |  |
| HARPAGOSIDE | 2.02 | Prestwick Chemical Inc. | BRD-K07996107 |  |
| HELIOTRINE | 3.19 | Prestwick Chemical Inc. | BRD-A08709697 |  |
| HESPERETIN | 3.31 | Prestwick Chemical Inc. | BRD-K30553453 |  |
| HESPERIDIN | 1.64 | Prestwick Chemical Inc. | BRD-K38903228 |  |
| HEXACHLOROPHENE | 5.00 | MicroSource Discovery Systems Inc. | BRD-K99792991 |  |
| HEXESTROL | 3.70 | Prestwick Chemical Inc. | BRD-A01078468 |  |
| HEXYLCAINE HYDROCHLORIDE | 3.36 | Prestwick Chemical Inc. | BRD-A97730597 |  |
| HEXYLRESORCINOL | 5.00 | MicroSource Discovery Systems Inc. | BRD-K99946902 |  |
| HIPPEASTRINE HYDROBROMIDE | 3.17 | Prestwick Chemical Inc. | BRD-K71003802 |  |
| HOECHST 33342 (CELL PERMEABLE) (BISBENZIMIDE) | 5.52 | Biomol International Inc. | BRD-K08554278 |  |
| HOMATROPINE BROMIDE | 5.00 | MicroSource Discovery Systems Inc. | BRD-A09539288 |  |
| HOMATROPINE HYDROBROMIDE (R,S) | 2.81 | Prestwick Chemical Inc. | BRD-A74975734 |  |
| HOMATROPINE METHYLBROMIDE | 5.00 | MicroSource Discovery Systems Inc. | BRD-A43999749 |  |
| HOMOCHLORCYCLIZINE DIHYDROCHLORIDE | 2.58 | Prestwick Chemical Inc. | BRD-A22769835 |  |
| HUPERZINE A | 5.00 | MicroSource Discovery Systems Inc. | BRD-K62240499 |  |
| HUPERZINE A [(-)-HUPERZINE A] | 10.32 | Biomol International Inc. | BRD-A47065382 |  |
| HYCANTHONE | 2.81 | Prestwick Chemical Inc. | BRD-K50406511 |  |
| HYDRALAZINE HYDROCHLORIDE | 5.00 | MicroSource Discovery Systems Inc. | BRD-K82103381 |  |
| HYDRASTINE HYDROCHLORIDE | 2.38 | Prestwick Chemical Inc. | BRD-K02715688 |  |
| HYDROCHLOROTHIAZIDE | 3.36 | Prestwick Chemical Inc. | BRD-K13078532 |  |
| HYDROCORTISONE | 5.00 | MicroSource Discovery Systems Inc. | BRD-A75172220 |  |
| HYDROCORTISONE ACETATE | 5.00 | MicroSource Discovery Systems Inc. | BRD-A65767837 |  |
| HYDROCORTISONE BASE | 2.76 | Prestwick Chemical Inc. | BRD-K93568044 |  |
| HYDROCORTISONE HEMISUCCINATE | 5.00 | MicroSource Discovery Systems Inc. | BRD-A07000685 |  |
| HYDROCOTARNINE | 5.00 | MicroSource Discovery Systems Inc. | BRD-K37447567 |  |
| HYDROCOTARNINE HYDROBROMIDE | 3.31 | Prestwick Chemical Inc. | BRD-K37447567 |  |
| HYDROFLUMETHIAZIDE | 3.02 | Prestwick Chemical Inc. | BRD-K36862742 |  |
| HYDROQUININE HYDROBROMIDE HYDRATE | 2.35 | Prestwick Chemical Inc. | BRD-A78295502 |  |
| HYDROXYAMPHETAMINE HYDROBROMIDE | 5.00 | MicroSource Discovery Systems Inc. | BRD-A80871782 |  |
| HYDROXYPROGESTERONE | 5.00 | MicroSource Discovery Systems Inc. | BRD-A39791822 |  |
| HYDROXYPROGESTERONE CAPROATE | 5.00 | MicroSource Discovery Systems Inc. | BRD-A29731977 |  |
| HYDROXYTACRINE MALEATE (R,S) | 3.03 | Prestwick Chemical Inc. | BRD-A98299281 |  |
| HYDROXYTOLUIC ACID | 5.00 | MicroSource Discovery Systems Inc. | BRD-K75608666 |  |
| HYDROXYZINE DIHYDROCHLORIDE | 2.23 | Prestwick Chemical Inc. | BRD-A62428732 |  |
| HYDROXYZINE PAMOATE | 5.00 | MicroSource Discovery Systems Inc. | BRD-A62428732 |  |
| HYMECROMONE METHYL ETHER | 5.00 | MicroSource Discovery Systems Inc. | BRD-K55766625 |  |
| HYOSCYAMINE | 5.00 | MicroSource Discovery Systems Inc. | BRD-A78303415 |  |
| HYOSCYAMINE (L) | 3.46 | Prestwick Chemical Inc. | BRD-K40530731 |  |
| IAA-94 | 7.00 | Biomol International Inc. | BRD-K85383046 |  |
| IB-MECA | 4.90 | Biomol International Inc. | BRD-A48809242 |  |
| IBMX | 11.25 | Biomol International Inc. | BRD-K94979336 |  |
| IBUPROFEN | 5.00 | MicroSource Discovery Systems Inc. | BRD-A17655518 |  |
| ICARIIN | 5.00 | MicroSource Discovery Systems Inc. | BRD-K65639003 |  |
| IFENPRODIL TARTRATE | 2.10 | Prestwick Chemical Inc. | BRD-A24191444 |  |
| IMIPRAMINE HYDROCHLORIDE | 3.16 | Prestwick Chemical Inc. | BRD-K38436528 |  |
| INDAPAMIDE | 2.73 | Prestwick Chemical Inc. | BRD-A95869247 |  |
| INDOLE-3-CARBINOL | 5.00 | MicroSource Discovery Systems Inc. | BRD-K01815685 |  |
| IOBENGUANE SULFATE | 2.68 | Prestwick Chemical Inc. | BRD-K43860855 |  |
| IOPANOIC ACID | 1.75 | Prestwick Chemical Inc. | BRD-A42628519 |  |
| IPRATROPIUM BROMIDE | 2.42 | Prestwick Chemical Inc. | BRD-A37432947 |  |
| IPRATROPIUM BROMIDE | 5.00 | MicroSource Discovery Systems Inc. | BRD-A07029265 |  |
| IPRIFLAVONE | 5.00 | MicroSource Discovery Systems Inc. | BRD-K36646537 |  |
| IPRONIAZIDE PHOSPHATE | 3.61 | Prestwick Chemical Inc. | BRD-K88568253 |  |
| IRBESARTAN | 5.00 | MicroSource Discovery Systems Inc. | BRD-K60038276 |  |
| IRIDIN | 5.00 | MicroSource Discovery Systems Inc. | BRD-K22550622 |  |
| IRIFLOPHENONE TRIMETHYL ETHER | 5.00 | MicroSource Discovery Systems Inc. | BRD-K78329127 |  |
| IRIGENIN TRIMETHYL ETHER | 5.00 | MicroSource Discovery Systems Inc. | BRD-K85880973 |  |
| IRIGENIN, 7-BENZYL ETHER | 5.00 | MicroSource Discovery Systems Inc. | BRD-K53634892 |  |
| IRIGENIN, DIBENZYL ETHER | 5.00 | MicroSource Discovery Systems Inc. | BRD-K41762421 |  |
| IRIGENOL | 5.00 | MicroSource Discovery Systems Inc. | BRD-K57398215 |  |
| IRILIN A DIMETHYL ETHER | 5.00 | MicroSource Discovery Systems Inc. | BRD-K92678294 |  |
| ISOBERGAPTENE | 5.00 | MicroSource Discovery Systems Inc. | BRD-K31678817 |  |
| ISOCARBOXAZID | 4.32 | Prestwick Chemical Inc. | BRD-K93332168 |  |
| ISOCONAZOLE | 2.40 | Prestwick Chemical Inc. | BRD-A93353767 |  |
| ISOCORYDINE (+) | 2.93 | Prestwick Chemical Inc. | BRD-K37049577 |  |
| ISOETHARINE MESYLATE SALT | 2.98 | Prestwick Chemical Inc. | BRD-A24587114 |  |
| ISOEUGENITOL | 5.00 | MicroSource Discovery Systems Inc. | BRD-K05737787 |  |
| ISOGINKGETIN | 5.00 | MicroSource Discovery Systems Inc. | BRD-K72661036 |  |
| ISOLIQUIRITIGENIN | 5.00 | MicroSource Discovery Systems Inc. | BRD-K33583600 |  |
| ISOPILOCARPINE NITRATE | 5.00 | MicroSource Discovery Systems Inc. | BRD-K75983763 |  |
| ISOPIMPINELLIN | 5.00 | MicroSource Discovery Systems Inc. | BRD-K72253829 |  |
| ISOQUINOLINE, 6,7-DIMETHOXY-1-METHYL-1,2,3,4-TETRAHYDRO, HYDROCHLORIDE | 4.10 | Prestwick Chemical Inc. | BRD-A75140635 |  |
| ISOTECTORIGENIN TRIMETHYL ETHER | 5.00 | MicroSource Discovery Systems Inc. | BRD-K10505056 |  |
| ISOXICAM | 2.98 | Prestwick Chemical Inc. | BRD-A75552914 |  |
| ISOXSUPRINE HYDROCHLORIDE | 2.96 | Prestwick Chemical Inc. | BRD-A74269027 |  |
| ISOXSUPRINE HYDROCHLORIDE | 5.00 | MicroSource Discovery Systems Inc. | BRD-A75024496 |  |
| ISRADIPINE | 2.69 | Prestwick Chemical Inc. | BRD-A90799790 |  |
| JUAREZIC ACID | 5.00 | MicroSource Discovery Systems Inc. | BRD-K12345912 |  |
| K252A | 0.53 | Biomol International Inc. | BRD-K13079976 |  |
| KAEMPFEROL | 3.49 | Prestwick Chemical Inc. | BRD-K12807006 |  |
| KARAKOLINE | 2.65 | Prestwick Chemical Inc. | BRD-A43849199 |  |
| KAWAIN | 4.34 | Prestwick Chemical Inc. | BRD-K09497549 |  |
| KETANSERIN | 5.00 | Biomol International Inc. | BRD-K49671696 |  |
| KETANSERIN TARTRATE HYDRATE | 1.77 | Prestwick Chemical Inc. | BRD-K49671696 |  |
| KETOCONAZOLE | 1.88 | Prestwick Chemical Inc. | BRD-K29113274 |  |
| KETOCONAZOLE | 5.00 | MicroSource Discovery Systems Inc. | BRD-A38350138 |  |
| KETOPROFEN | 3.93 | Prestwick Chemical Inc. | BRD-A97739905 |  |
| KETOTIFEN FUMARATE | 2.35 | Prestwick Chemical Inc. | BRD-K28936863 |  |
| KHELLIN | 3.84 | Prestwick Chemical Inc. | BRD-K80353807 |  |
| KINETIN RIBOSIDE | 5.00 | MicroSource Discovery Systems Inc. | BRD-K94325918 |  |
| KN-62 | 3.46 | Biomol International Inc. | BRD-A81177136 |  |
| KOPARIN 2'-METHYL ETHER | 5.00 | MicroSource Discovery Systems Inc. | BRD-K43224838 |  |
| KUHLMANNIN | 5.00 | MicroSource Discovery Systems Inc. | BRD-K18149487 |  |
| L-701,252 | 5.00 | Biomol International Inc. | BRD-K10176267 |  |
| L-701,324 | 5.00 | Biomol International Inc. | BRD-K08109516 |  |
| L-741,626 | 5.00 | Biomol International Inc. | BRD-K05181463 |  |
| L-741,742 | 5.00 | Biomol International Inc. | BRD-K13211965 |  |
| L-744,832 | 4.47 | Biomol International Inc. | BRD-A07952294 |  |
| L-745,870 | 5.00 | Biomol International Inc. | BRD-K05528470 |  |
| L-750,667 | 5.00 | Biomol International Inc. | BRD-K28806945 |  |
| L-CIS-DILTIAZEM | 6.03 | Biomol International Inc. | BRD-K81029756 |  |
| L-PHENYLALANINOL | 5.00 | MicroSource Discovery Systems Inc. | BRD-K44204252 |  |
| LABETALOL HYDROCHLORIDE | 2.74 | Prestwick Chemical Inc. | BRD-A07440155 |  |
| LANATOSIDE C | 5.00 | MicroSource Discovery Systems Inc. | BRD-A64242993 |  |
| LANSOPRAZOLE | 2.71 | Prestwick Chemical Inc. | BRD-A49172652 |  |
| LAPACHOL | 5.00 | MicroSource Discovery Systems Inc. | BRD-A06912736 |  |
| LATRUNCULIN B | 6.32 | Biomol International Inc. | BRD-K30455030 |  |
| LAUDANOSINE (R,S) | 2.80 | Prestwick Chemical Inc. | BRD-A24817035 |  |
| LAVENDUSTIN A | 6.56 | Biomol International Inc. | BRD-K23583188 |  |
| LE 300 | 5.00 | Biomol International Inc. | BRD-K01648091 |  |
| LECANORIC ACID | 5.00 | MicroSource Discovery Systems Inc. | BRD-K77578964 |  |
| LEFLUNOMIDE | 3.70 | Prestwick Chemical Inc. | BRD-K78692225 |  |
| LETROZOLE | 3.51 | Prestwick Chemical Inc. | BRD-K88789588 |  |
| LEUCOMISINE | 4.06 | Prestwick Chemical Inc. | BRD-K96119599 |  |
| LEVAMISOLE HYDROCHLORIDE | 4.15 | Prestwick Chemical Inc. | BRD-A46393198 |  |
| LEVAMISOLE HYDROCHLORIDE | 5.00 | MicroSource Discovery Systems Inc. | BRD-K73107279 |  |
| LEVOCABASTINE HYDROCHLORIDE | 2.19 | Prestwick Chemical Inc. | BRD-K33453211 |  |
| LEVULINIC ACID, 3-BENZYLIDENYL- | 5.00 | MicroSource Discovery Systems Inc. | BRD-K33104513 |  |
| LFM-A13 | 6.94 | Biomol International Inc. | BRD-A30655177 |  |
| LIDOCAINE | 10.67 | Biomol International Inc. | BRD-K52662033 |  |
| LIDOCAINE HYDROCHLORIDE | 3.69 | Prestwick Chemical Inc. | BRD-K52662033 |  |
| LIDOFLAZINE | 2.03 | Prestwick Chemical Inc. | BRD-K62996583 |  |
| LIMONIN | 5.00 | MicroSource Discovery Systems Inc. | BRD-K05906022 |  |
| LINCOMYCIN HYDROCHLORIDE | 2.26 | Prestwick Chemical Inc. | BRD-K81447180 |  |
| LINCOMYCIN HYDROCHLORIDE | 5.00 | MicroSource Discovery Systems Inc. | BRD-K08033334 |  |
| LINOLEAMIDE | 0.50 | Biomol International Inc. | BRD-K64044582 |  |
| LINOLEIC ACID | 0.50 | Biomol International Inc. | BRD-K08973992 |  |
| LINOLENIC ACID (18:3 N-3) | 0.50 | Biomol International Inc. | BRD-K33396764 |  |
| LIOTHYRONINE | 5.00 | MicroSource Discovery Systems Inc. | BRD-A74642112 |  |
| LIQUIRITIGENIN DIMETHYL ETHER | 5.00 | MicroSource Discovery Systems Inc. | BRD-A68039575 |  |
| LISINOPRIL | 2.26 | Prestwick Chemical Inc. | BRD-K67966701 |  |
| LOBARIC ACID | 5.00 | MicroSource Discovery Systems Inc. | BRD-K88849294 |  |
| LOBELANIDINE HYDROCHLORIDE | 2.66 | Prestwick Chemical Inc. | BRD-A59215453 |  |
| LOBELINE ALPHA (-) HYDROCHORIDE | 2.67 | Prestwick Chemical Inc. | BRD-K66206289 |  |
| LOMATIN | 5.00 | MicroSource Discovery Systems Inc. | BRD-A19918940 |  |
| LOMEFLOXACIN HYDROCHLORIDE | 2.58 | Prestwick Chemical Inc. | BRD-A75850590 |  |
| LONCHOCARPIC ACID | 5.00 | MicroSource Discovery Systems Inc. | BRD-K32049721 |  |
| LONIDAMINE | 5.00 | MicroSource Discovery Systems Inc. | BRD-K96670504 |  |
| LOPERAMIDE | 5.24 | Biomol International Inc. | BRD-K61250553 |  |
| LOPERAMIDE HYDROCHLORIDE | 1.95 | Prestwick Chemical Inc. | BRD-K61250553 |  |
| LORATADINE | 5.00 | MicroSource Discovery Systems Inc. | BRD-K82795137 |  |
| LOSARTAN | 5.00 | MicroSource Discovery Systems Inc. | BRD-K76205745 |  |
| LOXAPINE SUCCINATE | 2.24 | Prestwick Chemical Inc. | BRD-K39915878 |  |
| LUFENURON | 5.00 | MicroSource Discovery Systems Inc. | BRD-A71774530 |  |
| LUMICOLCHICINE GAMMA | 2.50 | Prestwick Chemical Inc. | BRD-K99411983 |  |
| LUPANINE PERCHLORATE | 5.00 | MicroSource Discovery Systems Inc. | BRD-A92826379 |  |
| LUTEOLIN | 3.49 | Prestwick Chemical Inc. | BRD-K05236810 |  |
| LY-163,502 | 5.00 | Biomol International Inc. | BRD-K13261168 |  |
| LY-165,163 | 5.00 | Biomol International Inc. | BRD-K14282469 |  |
| LY-171883 | 0.50 | Biomol International Inc. | BRD-K74765201 |  |
| LY-278,584 | 5.00 | Biomol International Inc. | BRD-A12016240 |  |
| LY-294002 | 8.13 | Biomol International Inc. | BRD-K27305650 |  |
| LY-83583 | 9.99 | Biomol International Inc. | BRD-K62792802 |  |
| LYCORINE | 5.00 | MicroSource Discovery Systems Inc. | BRD-A10335634 |  |
| LYCORINE | 8.70 | Biomol International Inc. | BRD-K64909280 |  |
| LYCORINE HYDROCHLORIDE | 3.09 | Prestwick Chemical Inc. | BRD-A35215253 |  |
| LYSERGOL | 3.93 | Prestwick Chemical Inc. | BRD-K27871032 |  |
| LYSYLPHENYLALANYLTYROSINE | 5.00 | MicroSource Discovery Systems Inc. | BRD-K47328134 |  |
| M-CHLOROPHENYLBIGUANIDE | 5.00 | Biomol International Inc. | BRD-K36965586 |  |
| MAACKIAIN | 5.00 | MicroSource Discovery Systems Inc. | BRD-A95445494 |  |
| MACLUROXANTHONE | 5.00 | MicroSource Discovery Systems Inc. | BRD-K08362773 |  |
| MADECASSIC ACID | 5.00 | MicroSource Discovery Systems Inc. | BRD-A84189249 |  |
| MANUMYCIN A | 4.54 | Biomol International Inc. | BRD-K78599730 |  |
| MAPP, D-ERYTHRO | 0.50 | Biomol International Inc. | BRD-K76274772 |  |
| MAPP, L-ERYTHRO | 0.50 | Biomol International Inc. | BRD-K09635134 |  |
| MAPROTILINE HYDROCHLORIDE | 3.19 | Prestwick Chemical Inc. | BRD-K03319035 |  |
| MARMESIN | 5.00 | MicroSource Discovery Systems Inc. | BRD-K36377456 |  |
| MARMESIN ACETATE | 5.00 | MicroSource Discovery Systems Inc. | BRD-K57001030 |  |
| MBCQ | 7.97 | Biomol International Inc. | BRD-K64746805 |  |
| MCI-186 | 14.35 | Biomol International Inc. | BRD-K35458079 |  |
| MDL 29951 | 5.00 | Biomol International Inc. | BRD-K59753853 |  |
| MDL 72832 | 5.00 | Biomol International Inc. | BRD-A17453586 |  |
| MDL 73005EF | 5.00 | Biomol International Inc. | BRD-A50764878 |  |
| MDL-28170 | 6.54 | Biomol International Inc. | BRD-K43245338 |  |
| MDL-72222 | 5.00 | Biomol International Inc. | BRD-A85587465 |  |
| MEAD ACID (20:3 N-9) | 0.50 | Biomol International Inc. | BRD-K96144918 |  |
| MEBENDAZOLE | 3.39 | Prestwick Chemical Inc. | BRD-K77987382 |  |
| MEBEVERINE HYDROCHLORIDE | 2.15 | Prestwick Chemical Inc. | BRD-A09467419 |  |
| MEBHYDROLIN NAPHTHALENESULFONATE | 5.00 | MicroSource Discovery Systems Inc. | BRD-K29713308 |  |
| MECLOFENAMATE SODIUM | 5.00 | MicroSource Discovery Systems Inc. | BRD-K50398167 |  |
| MECLOFENAMIC ACID SODIUM SALT MONOHYDRATE | 2.97 | Prestwick Chemical Inc. | BRD-K50398167 |  |
| MECLOFENOXATE HYDROCHLORIDE | 3.40 | Prestwick Chemical Inc. | BRD-K10314788 |  |
| MECLOZINE DIHYDROCHLORIDE | 2.16 | Prestwick Chemical Inc. | BRD-A50311610 |  |
| MEDROXYPROGESTERONE ACETATE | 5.00 | MicroSource Discovery Systems Inc. | BRD-A61221616 |  |
| MEDRYSONE | 2.90 | Prestwick Chemical Inc. | BRD-K56515112 |  |
| MEDRYSONE | 5.00 | MicroSource Discovery Systems Inc. | BRD-A20126139 |  |
| MEFENAMIC ACID | 4.14 | Prestwick Chemical Inc. | BRD-K92778217 |  |
| MEFEXAMIDE HYDROCHLORIDE | 3.16 | Prestwick Chemical Inc. | BRD-K20655524 |  |
| MEFLOQUINE HYDROCHLORIDE | 2.41 | Prestwick Chemical Inc. | BRD-A89585551 |  |
| MEGESTROL ACETATE | 5.00 | MicroSource Discovery Systems Inc. | BRD-A35989968 |  |
| MELATONIN | 4.31 | Prestwick Chemical Inc. | BRD-K97530723 |  |
| MELOXICAM | 5.00 | MicroSource Discovery Systems Inc. | BRD-A84174393 |  |
| MELPHALAN | 5.00 | MicroSource Discovery Systems Inc. | BRD-K87827419 |  |
| MENTHYL BENZOATE | 5.00 | MicroSource Discovery Systems Inc. | BRD-K12427081 |  |
| MEPENZOLATE BROMIDE | 2.37 | Prestwick Chemical Inc. | BRD-A62421304 |  |
| MEPHENYTOIN | 4.58 | Prestwick Chemical Inc. | BRD-A83937277 |  |
| MEPIVACAINE HYDROCHLORIDE | 5.00 | MicroSource Discovery Systems Inc. | BRD-A03216249 |  |
| MEPRYLCAINE HYDROCHLORIDE | 3.68 | Prestwick Chemical Inc. | BRD-K65417056 |  |
| MEPTAZINOL HYDROCHLORIDE | 3.71 | Prestwick Chemical Inc. | BRD-A02710418 |  |
| MESORIDAZINE | 5.00 | Biomol International Inc. | BRD-A14395271 |  |
| MESORIDAZINE BESYLATE | 1.84 | Prestwick Chemical Inc. | BRD-A14395271 |  |
| MESULERGINE | 5.00 | Biomol International Inc. | BRD-K91336023 |  |
| METACETAMOL | 5.00 | MicroSource Discovery Systems Inc. | BRD-K16474819 |  |
| METAMECONINE | 5.00 | MicroSource Discovery Systems Inc. | BRD-K94324294 |  |
| METAXALONE | 5.00 | MicroSource Discovery Systems Inc. | BRD-A94709349 |  |
| METERGOLINE PHENYLMETHYL ESTER | 5.00 | Biomol International Inc. | BRD-K56699285 |  |
| METHACYCLINE HYDROCHLORIDE | 2.09 | Prestwick Chemical Inc. | BRD-A49035384 |  |
| METHANTHELINE BROMIDE | 2.37 | Prestwick Chemical Inc. | BRD-K09859624 |  |
| METHAPYRILENE HYDROCHLORIDE | 3.36 | Prestwick Chemical Inc. | BRD-K47323024 |  |
| METHAZOLAMIDE | 4.23 | Prestwick Chemical Inc. | BRD-K71053238 |  |
| METHIAZOLE | 3.77 | Prestwick Chemical Inc. | BRD-K02764365 |  |
| METHICILLIN SODIUM | 5.00 | MicroSource Discovery Systems Inc. | BRD-K34388247 |  |
| METHIONYL-LEUCYLPHENYLALANINE ACETATE | 5.00 | MicroSource Discovery Systems Inc. | BRD-A02189320 |  |
| METHIOTHEPIN | 5.00 | Biomol International Inc. | BRD-A07932845 |  |
| METHIOTHEPIN MALEATE | 2.12 | Prestwick Chemical Inc. | BRD-A07932845 |  |
| METHOCARBAMOL | 4.15 | Prestwick Chemical Inc. | BRD-A31521121 |  |
| METHOMYL | 5.00 | MicroSource Discovery Systems Inc. | BRD-K87817668 |  |
| METHOPRENE ACID | 0.50 | Biomol International Inc. | BRD-A41145729 |  |
| METHOXY VERAPAMIL | 5.16 | Biomol International Inc. | BRD-A52922642 |  |
| METHOXY-6-HARMALAN | 4.67 | Prestwick Chemical Inc. | BRD-K54906270 |  |
| METHOXY-8-PSORALEN | 4.63 | Prestwick Chemical Inc. | BRD-K63430059 |  |
| METHOXYVONE | 5.00 | MicroSource Discovery Systems Inc. | BRD-K56057104 |  |
| METHSCOPOLAMINE BROMIDE | 5.00 | MicroSource Discovery Systems Inc. | BRD-A03932035 |  |
| METHYL ROBUSTONE | 5.00 | MicroSource Discovery Systems Inc. | BRD-K25811799 |  |
| METHYL-6,7-DIMETHOXY-4-ETHYL-BETA-CARBOLINE-3-CARBOXYLATE | 5.00 | Biomol International Inc. | BRD-K14844937 |  |
| METHYL-BETA-CARBOLINE-3-CARBOXYLATE | 5.00 | Biomol International Inc. | BRD-K28680267 |  |
| METHYLERGOMETRINE | 5.00 | Biomol International Inc. | BRD-K34685430 |  |
| METHYLERGOMETRINE MALEATE | 2.20 | Prestwick Chemical Inc. | BRD-K34685430 |  |
| METHYLORSELLINIC ACID, ETHYL ESTER | 5.00 | MicroSource Discovery Systems Inc. | BRD-K68438316 |  |
| METHYLPREDNISOLONE | 5.00 | MicroSource Discovery Systems Inc. | BRD-A53176877 |  |
| METHYLPREDNISOLONE, 6-ALPHA | 2.67 | Prestwick Chemical Inc. | BRD-K35240538 |  |
| METHYSERGIDE | 5.00 | Biomol International Inc. | BRD-K35941380 |  |
| METICRANE | 3.63 | Prestwick Chemical Inc. | BRD-K58265391 |  |
| METIXENE HYDROCHLORIDE | 2.75 | Prestwick Chemical Inc. | BRD-A33711280 |  |
| METOCLOPRAMIDE | 5.00 | Biomol International Inc. | BRD-K75641298 |  |
| METOCLOPRAMIDE MONOHYDROCHLORIDE | 2.97 | Prestwick Chemical Inc. | BRD-K75641298 |  |
| METOLACHLOR | 5.00 | MicroSource Discovery Systems Inc. | BRD-A43135847 |  |
| METOLAZONE | 2.73 | Prestwick Chemical Inc. | BRD-A61793559 |  |
| METOPROLOL TARTRATE | 5.00 | MicroSource Discovery Systems Inc. | BRD-A03623303 |  |
| MEVINOLIN (LOVASTATIN) | 6.18 | Biomol International Inc. | BRD-K09416995 |  |
| MEXICANOLIDE | 5.00 | MicroSource Discovery Systems Inc. | BRD-A64523751 |  |
| MEXILETINE HYDROCHLORIDE | 4.64 | Prestwick Chemical Inc. | BRD-A64092382 |  |
| MIANSERINE HYDROCHLORIDE | 3.32 | Prestwick Chemical Inc. | BRD-A19661776 |  |
| MICONAZOLE | 2.40 | Prestwick Chemical Inc. | BRD-A82396632 |  |
| MICONAZOLE NITRATE | 5.00 | MicroSource Discovery Systems Inc. | BRD-A82396632 |  |
| MIDECAMYCIN | 1.23 | Prestwick Chemical Inc. | BRD-A34897638 |  |
| MIDODRINE HYDROCHLORIDE | 3.44 | Prestwick Chemical Inc. | BRD-A79981887 |  |
| MIFEPRISTONE | 2.33 | Prestwick Chemical Inc. | BRD-K37270826 |  |
| MINAPRINE DIHYDROCHLORIDE | 2.69 | Prestwick Chemical Inc. | BRD-K02867583 |  |
| MINOXIDIL | 11.95 | Biomol International Inc. | BRD-K06902185 |  |
| MINOXIDIL | 4.78 | Prestwick Chemical Inc. | BRD-K14888893 |  |
| MITOMYCIN C | 5.00 | MicroSource Discovery Systems Inc. | BRD-A48237631 |  |
| MITOMYCIN C | 7.48 | Biomol International Inc. | BRD-K59670716 |  |
| MITOXANTRONE DIHYDROCHLORIDE | 1.93 | Prestwick Chemical Inc. | BRD-K21680192 |  |
| MK 212 | 5.00 | Biomol International Inc. | BRD-K19554809 |  |
| ML7 | 6.01 | Biomol International Inc. | BRD-K93201660 |  |
| ML9 | 7.70 | Biomol International Inc. | BRD-K68402494 |  |
| MODAFINIL | 5.00 | MicroSource Discovery Systems Inc. | BRD-A16332958 |  |
| MOLINDONE HYDROCHLORIDE | 3.20 | Prestwick Chemical Inc. | BRD-A65280694 |  |
| MOLSIDOMINE | 4.13 | Prestwick Chemical Inc. | BRD-K35531059 |  |
| MOLSIDOMINE | 5.00 | MicroSource Discovery Systems Inc. | BRD-K63861289 |  |
| MOMETASONE FUROATE | 1.92 | Prestwick Chemical Inc. | BRD-K60640630 |  |
| MONOCROTALINE | 3.07 | Prestwick Chemical Inc. | BRD-K65508953 |  |
| MORICIZINE HYDROCHLORIDE | 2.16 | Prestwick Chemical Inc. | BRD-K21548250 |  |
| MOXISYLYTE HYDROCHORIDE | 3.17 | Prestwick Chemical Inc. | BRD-K81144366 |  |
| MOXONIDINE | 4.14 | Prestwick Chemical Inc. | BRD-K77771411 |  |
| MUNDOSERONE | 5.00 | MicroSource Discovery Systems Inc. | BRD-K69294151 |  |
| MUNDULONE | 5.00 | MicroSource Discovery Systems Inc. | BRD-A93572202 |  |
| MUNINGIN | 5.00 | MicroSource Discovery Systems Inc. | BRD-K69654927 |  |
| MY-5445 | 7.53 | Biomol International Inc. | BRD-K90524085 |  |
| MYCOPHENOLIC ACID | 3.12 | Prestwick Chemical Inc. | BRD-K63750851 |  |
| MYOSMINE | 6.84 | Prestwick Chemical Inc. | BRD-K67556876 |  |
| MYRICETIN | 3.14 | Prestwick Chemical Inc. | BRD-K43149758 |  |
| N- (2-AMINOETHYL)-4-CHLOROBENZAMIDE (RO-16-6491) | 5.00 | MicroSource Discovery Systems Inc. | BRD-K44899736 |  |
| N- (3-TRIFLUOROMETHYLPHENYL)PIPERAZINE HYDROCHLORIDE (TFMPP) | 5.00 | MicroSource Discovery Systems Inc. | BRD-K94887716 |  |
| N- (9-FLUORENYLMETHOXYCARBONYL)-L-LEUCINE | 5.00 | MicroSource Discovery Systems Inc. | BRD-K65275554 |  |
| N-(4-BROMOBENZYL)-5-METHOXYTRYPTAMINE | 5.00 | Biomol International Inc. | BRD-K37883585 |  |
| N-ARACHIDONOYLGLYCINE | 0.50 | Biomol International Inc. | BRD-K51557114 |  |
| N-DESMETHYLCLOZAPINE | 5.00 | Biomol International Inc. | BRD-K10042277 |  |
| N-FORMYLMETHIONYL-LEUCYLPHENYLALANINE | 5.00 | MicroSource Discovery Systems Inc. | BRD-A27693633 |  |
| N-FORMYLMETHIONYLPHENYLALANINE | 5.00 | MicroSource Discovery Systems Inc. | BRD-A09975616 |  |
| N-LINOLEOYLGLYCINE | 0.50 | Biomol International Inc. | BRD-K76293260 |  |
| N-METHYL (-)EPHEDRINE | 5.00 | MicroSource Discovery Systems Inc. | BRD-K82236108 |  |
| N-METHYLANTHRANILIC ACID | 5.00 | MicroSource Discovery Systems Inc. | BRD-K06439119 |  |
| N-PHENYLANTHRANILIC | 11.72 | Biomol International Inc. | BRD-K80863915 |  |
| N9-ISOPROPYLOLOMOUCINE | 7.66 | Biomol International Inc. | BRD-K71726959 |  |
| NAFCILLIN SODIUM | 5.00 | MicroSource Discovery Systems Inc. | BRD-A99402294 |  |
| NAFRONYL OXALATE | 2.11 | Prestwick Chemical Inc. | BRD-A67862938 |  |
| NAFTIFINE HYDROCHLORIDE | 3.09 | Prestwick Chemical Inc. | BRD-K74141488 |  |
| NAFTOPIDIL DIHYDROCHLORIDE | 2.15 | Prestwick Chemical Inc. | BRD-A01787639 |  |
| NALBUPHINE HYDROCHLORIDE | 2.54 | Prestwick Chemical Inc. | BRD-K66404838 |  |
| NALBUPHINE HYDROCHLORIDE | 5.00 | MicroSource Discovery Systems Inc. | BRD-A92651262 |  |
| NALIDIXIC ACID SODIUM SALT HYDRATE | 3.66 | Prestwick Chemical Inc. | BRD-K47886988 |  |
| NALOXONE HYDROCHLORIDE | 2.75 | Prestwick Chemical Inc. | BRD-K67511046 |  |
| NALOXONE HYDROCHLORIDE | 5.00 | MicroSource Discovery Systems Inc. | BRD-A70461345 |  |
| NALTREXONE HYDROCHLORIDE DIHYDRATE | 2.42 | Prestwick Chemical Inc. | BRD-K88172511 |  |
| NAN-190 | 5.00 | Biomol International Inc. | BRD-K69195780 |  |
| NAPHAZOLINE HYDROCHLORIDE | 4.05 | Prestwick Chemical Inc. | BRD-K77641333 |  |
| NAPROXEN | 4.34 | Prestwick Chemical Inc. | BRD-A87719232 |  |
| NAPROXEN(+) | 5.00 | MicroSource Discovery Systems Inc. | BRD-K59197931 |  |
| NAPROXOL | 5.00 | MicroSource Discovery Systems Inc. | BRD-K34014345 |  |
| NARINGENIN | 5.00 | MicroSource Discovery Systems Inc. | BRD-K08832567 |  |
| NARINGENINE | 3.67 | Prestwick Chemical Inc. | BRD-A94669766 |  |
| NARINGIN | 5.00 | MicroSource Discovery Systems Inc. | BRD-K56759808 |  |
| NARINGIN HYDRATE | 1.67 | Prestwick Chemical Inc. | BRD-K02953697 |  |
| NEFOPAM HYDROCHLORIDE | 3.45 | Prestwick Chemical Inc. | BRD-A78877355 |  |
| NEOHESPERIDIN DIHYDROCHALCONE | 5.00 | MicroSource Discovery Systems Inc. | BRD-A84867566 |  |
| NEOPINE | 5.00 | MicroSource Discovery Systems Inc. | BRD-K34252131 |  |
| NEOSTIGMINE BROMIDE | 3.29 | Prestwick Chemical Inc. | BRD-K18922609 |  |
| NERIIFOLIN | 5.00 | MicroSource Discovery Systems Inc. | BRD-A31385885 |  |
| NIALAMIDE | 3.35 | Prestwick Chemical Inc. | BRD-K12102668 |  |
| NICARDIPINE | 5.21 | Biomol International Inc. | BRD-A26711594 |  |
| NICARDIPINE HYDROCHLORIDE | 1.94 | Prestwick Chemical Inc. | BRD-A26711594 |  |
| NICERGOLINE | 2.06 | Prestwick Chemical Inc. | BRD-K76810206 |  |
| NICLOSAMIDE | 3.06 | Prestwick Chemical Inc. | BRD-K35960502 |  |
| NICOTINE DITARTRATE | 5.00 | MicroSource Discovery Systems Inc. | BRD-K05395900 |  |
| NIFENAZONE | 3.24 | Prestwick Chemical Inc. | BRD-K47407372 |  |
| NIFLUMIC ACID | 8.86 | Biomol International Inc. | BRD-K98763141 |  |
| NIFUROXAZIDE | 3.63 | Prestwick Chemical Inc. | BRD-K68188368 |  |
| NIFURTIMOX | 3.48 | Prestwick Chemical Inc. | BRD-A84020532 |  |
| NIGULDIPINE | 4.10 | Biomol International Inc. | BRD-A62336480 |  |
| NILOTICIN | 5.00 | MicroSource Discovery Systems Inc. | BRD-A57556180 |  |
| NIMODIPINE | 5.97 | Biomol International Inc. | BRD-A58048407 |  |
| NIRIDAZOLE | 4.67 | Prestwick Chemical Inc. | BRD-K53123955 |  |
| NITRARINE DIHYDROCHLORIDE | 2.63 | Prestwick Chemical Inc. | BRD-A60583491 |  |
| NITRENDIPINE | 6.94 | Biomol International Inc. | BRD-A02006392 |  |
| NITROCARAMIPHEN HYDROCHLORIDE | 2.70 | Prestwick Chemical Inc. | BRD-K28453807 |  |
| NITROFURAL | 5.05 | Prestwick Chemical Inc. | BRD-K79092138 |  |
| NITROFURANTOIN | 4.20 | Prestwick Chemical Inc. | BRD-K76927775 |  |
| NITROMIDE | 5.00 | MicroSource Discovery Systems Inc. | BRD-K76381435 |  |
| NO-711 | 5.00 | Biomol International Inc. | BRD-K42221274 |  |
| NOMEGESTROL ACETATE | 2.70 | Prestwick Chemical Inc. | BRD-K27351809 |  |
| NOMIFENSINE MALEATE | 2.82 | Prestwick Chemical Inc. | BRD-A29644307 |  |
| NORCYCLOBENZAPRINE | 3.83 | Prestwick Chemical Inc. | BRD-K63165456 |  |
| NORETHINDRONE | 3.35 | Prestwick Chemical Inc. | BRD-K92073408 |  |
| NORETHINDRONE | 5.00 | MicroSource Discovery Systems Inc. | BRD-A39415247 |  |
| NORETHYNODREL | 3.35 | Prestwick Chemical Inc. | BRD-K80334323 |  |
| NORFLOXACIN | 3.13 | Prestwick Chemical Inc. | BRD-K11196887 |  |
| NORGESTREL-(-)-D | 3.20 | Prestwick Chemical Inc. | BRD-K35189033 |  |
| NORHARMAN | 5.00 | MicroSource Discovery Systems Inc. | BRD-K47467075 |  |
| NORSTICTIC ACID | 5.00 | MicroSource Discovery Systems Inc. | BRD-A80079592 |  |
| NORTRIPTYLINE HYDROCHLORIDE | 3.34 | Prestwick Chemical Inc. | BRD-K91263825 |  |
| NOSCAPINE | 2.42 | Prestwick Chemical Inc. | BRD-K89237706 |  |
| NOSCAPINE HYDROCHLORIDE | 5.00 | MicroSource Discovery Systems Inc. | BRD-K91301684 |  |
| NOVOBIOCIN SODIUM | 5.00 | MicroSource Discovery Systems Inc. | BRD-K85307935 |  |
| NOVOBIOCIN SODIUM SALT | 1.57 | Prestwick Chemical Inc. | BRD-A76478388 |  |
| NPPB | 8.32 | Biomol International Inc. | BRD-K89272762 |  |
| NS-1619 | 6.90 | Biomol International Inc. | BRD-K54210043 |  |
| NSC-95397 | 8.05 | Biomol International Inc. | BRD-K68143200 |  |
| OBACUNOL | 5.00 | MicroSource Discovery Systems Inc. | BRD-A31602150 |  |
| OFLOXACIN | 2.77 | Prestwick Chemical Inc. | BRD-A24228527 |  |
| OMEPRAZOLE | 2.90 | Prestwick Chemical Inc. | BRD-A55962179 |  |
| ONDANSETRON | 5.00 | Biomol International Inc. | BRD-A19736161 |  |
| ONDANSETRON HYDROCHLORIDE | 3.03 | Prestwick Chemical Inc. | BRD-A19736161 |  |
| ONONETIN | 5.00 | MicroSource Discovery Systems Inc. | BRD-K85385671 |  |
| ORNIDAZOLE | 4.55 | Prestwick Chemical Inc. | BRD-A42759514 |  |
| ORSELLINIC ACID, ETHYL ESTER | 5.00 | MicroSource Discovery Systems Inc. | BRD-K97487499 |  |
| ORTHOTHYMOTINIC ACID | 5.00 | MicroSource Discovery Systems Inc. | BRD-K95788524 |  |
| OSAJIN | 5.00 | MicroSource Discovery Systems Inc. | BRD-K01836637 |  |
| OSELTAMIVIR TARTRATE | 5.00 | MicroSource Discovery Systems Inc. | BRD-K76011241 |  |
| OSTHOL | 5.00 | MicroSource Discovery Systems Inc. | BRD-K78294846 |  |
| OUABAIN | 5.00 | MicroSource Discovery Systems Inc. | BRD-A68930007 |  |
| OXACILLIN SODIUM | 5.00 | MicroSource Discovery Systems Inc. | BRD-A66481418 |  |
| OXALAMINE CITRATE SALT | 2.29 | Prestwick Chemical Inc. | BRD-K42596464 |  |
| OXAPROZIN | 3.41 | Prestwick Chemical Inc. | BRD-K25394294 |  |
| OXETHAZAINE | 2.14 | Prestwick Chemical Inc. | BRD-K56940463 |  |
| OXFENDAZOLE | 5.00 | MicroSource Discovery Systems Inc. | BRD-A33447119 |  |
| OXIBENDAZOLE | 5.00 | MicroSource Discovery Systems Inc. | BRD-K52075715 |  |
| OXICONAZOLE NITRATE | 5.00 | MicroSource Discovery Systems Inc. | BRD-K56104152 |  |
| OXPRENOLOL HYDROCHLORIDE | 3.31 | Prestwick Chemical Inc. | BRD-A43671941 |  |
| OXYBUTYNIN CHLORIDE | 2.54 | Prestwick Chemical Inc. | BRD-A65013509 |  |
| OXYMETAZOLINE | 5.00 | Biomol International Inc. | BRD-K16195444 |  |
| OXYPHENBUTAZONE | 3.08 | Prestwick Chemical Inc. | BRD-A33749298 |  |
| OXYQUINOLINE HEMISULFATE | 5.00 | MicroSource Discovery Systems Inc. | BRD-K66808046 |  |
| OZAGREL HYDROCHLORIDE | 3.78 | Prestwick Chemical Inc. | BRD-K53061490 |  |
| PACHYRRHIZIN | 5.00 | MicroSource Discovery Systems Inc. | BRD-K02070226 |  |
| PACLITAXEL | 1.17 | Prestwick Chemical Inc. | BRD-K62008436 |  |
| PAEONOL | 5.00 | MicroSource Discovery Systems Inc. | BRD-K94239562 |  |
| PALMATINE CHLORIDE | 2.57 | Prestwick Chemical Inc. | BRD-K58466253 |  |
| PANTOPRAZOLE | 5.00 | MicroSource Discovery Systems Inc. | BRD-A22380646 |  |
| PAPAVERINE HYDROCHLORIDE | 2.66 | Prestwick Chemical Inc. | BRD-K15567136 |  |
| PARAXANTHINE | 5.00 | MicroSource Discovery Systems Inc. | BRD-K24084088 |  |
| PARBENDAZOLE | 4.04 | Prestwick Chemical Inc. | BRD-K02407574 |  |
| PARGYLINE HYDROCHLORIDE | 5.11 | Prestwick Chemical Inc. | BRD-K83597974 |  |
| PARTHENOLIDE | 10.07 | Biomol International Inc. | BRD-K29883696 |  |
| PARTHENOLIDE | 5.00 | MicroSource Discovery Systems Inc. | BRD-A27666114 |  |
| PAXILLINE | 5.74 | Biomol International Inc. | BRD-K38251852 |  |
| PCA 4248 | 6.92 | Biomol International Inc. | BRD-A29289453 |  |
| PCO-400 | 8.35 | Biomol International Inc. | BRD-K52721684 |  |
| PECTOLINARIN | 5.00 | MicroSource Discovery Systems Inc. | BRD-K20043699 |  |
| PEFLOXACINE MESYLATE | 5.00 | MicroSource Discovery Systems Inc. | BRD-K55034111 |  |
| PENICILLIN V POTASSIUM | 5.00 | MicroSource Discovery Systems Inc. | BRD-K43966364 |  |
| PENITREM A | 3.94 | Biomol International Inc. | BRD-K03842655 |  |
| PENTACHLOROPHENOL | 5.00 | MicroSource Discovery Systems Inc. | BRD-K50711164 |  |
| PENTOXIFYLLINE | 3.59 | Prestwick Chemical Inc. | BRD-K57569181 |  |
| PERGOLIDE MESYLATE | 2.44 | Prestwick Chemical Inc. | BRD-K60770992 |  |
| PERGOLIDE METHANESULFONATE | 5.00 | Biomol International Inc. | BRD-K60770992 |  |
| PERICIAZINE | 5.00 | MicroSource Discovery Systems Inc. | BRD-K89669299 |  |
| PERINDOPRIL ERBUMINE | 5.00 | MicroSource Discovery Systems Inc. | BRD-K92731339 |  |
| PERPHENAZINE | 2.48 | Prestwick Chemical Inc. | BRD-K10995081 |  |
| PERUVOSIDE | 5.00 | MicroSource Discovery Systems Inc. | BRD-A57089740 |  |
| PEUCEDANIN | 5.00 | MicroSource Discovery Systems Inc. | BRD-K72034655 |  |
| PHENACEMIDE | 5.00 | MicroSource Discovery Systems Inc. | BRD-K40905133 |  |
| PHENACETIN | 5.58 | Prestwick Chemical Inc. | BRD-K38323065 |  |
| PHENACYLAMINE HYDROCHLORIDE | 5.00 | MicroSource Discovery Systems Inc. | BRD-K61831307 |  |
| PHENAMIL | 8.18 | Biomol International Inc. | BRD-K21350491 |  |
| PHENAZOPYRIDINE HYDROCHLORIDE | 4.00 | Prestwick Chemical Inc. | BRD-K76304753 |  |
| PHENELZINE SULFATE | 4.27 | Prestwick Chemical Inc. | BRD-K87024524 |  |
| PHENETHICILLIN POTASSIUM SALT | 2.48 | Prestwick Chemical Inc. | BRD-A66025870 |  |
| PHENFORMIN HYDROCHLORIDE | 4.14 | Prestwick Chemical Inc. | BRD-K11399644 |  |
| PHENIRAMINE MALEATE | 2.81 | Prestwick Chemical Inc. | BRD-A23072235 |  |
| PHENOLPHTHALEIN | 5.00 | MicroSource Discovery Systems Inc. | BRD-K19227686 |  |
| PHENOTHRIN | 5.00 | MicroSource Discovery Systems Inc. | BRD-A22106989 |  |
| PHENSUXIMIDE | 5.29 | Prestwick Chemical Inc. | BRD-A18043272 |  |
| PHENTOLAMINE | 8.89 | Biomol International Inc. | BRD-K90333595 |  |
| PHENTOLAMINE HYDROCHLORIDE | 3.15 | Prestwick Chemical Inc. | BRD-K90333595 |  |
| PHENYLACETOHYDROXAMIC ACID | 5.00 | MicroSource Discovery Systems Inc. | BRD-K74112339 |  |
| PHENYLBUTYRIC ACID | 5.00 | MicroSource Discovery Systems Inc. | BRD-K67102207 |  |
| PHENYTOIN | 9.91 | Biomol International Inc. | BRD-K55930204 |  |
| PHLORETIN | 5.00 | MicroSource Discovery Systems Inc. | BRD-K15563106 |  |
| PHLORIDZIN | 5.00 | MicroSource Discovery Systems Inc. | BRD-K73756878 |  |
| PHOSALONE | 5.00 | MicroSource Discovery Systems Inc. | BRD-K71671197 |  |
| PHYSOSTIGMINE SALICYLATE | 5.00 | MicroSource Discovery Systems Inc. | BRD-K25650355 |  |
| PICEATANNOL | 10.24 | Biomol International Inc. | BRD-K91509126 |  |
| PICEID | 5.00 | MicroSource Discovery Systems Inc. | BRD-K14536225 |  |
| PICOTAMIDE MONOHYDRATE | 2.54 | Prestwick Chemical Inc. | BRD-K67277431 |  |
| PICROPODOPHYLLOTOXIN | 5.00 | MicroSource Discovery Systems Inc. | BRD-K81829253 |  |
| PICROPODOPHYLLOTOXIN ACETATE | 5.00 | MicroSource Discovery Systems Inc. | BRD-K99108905 |  |
| PICROTOXININ | 3.42 | Prestwick Chemical Inc. | BRD-A37817666 |  |
| PICROTOXININ | 5.00 | Biomol International Inc. | BRD-K95554982 |  |
| PIFITHRIN | 8.73 | Biomol International Inc. | BRD-K66874953 |  |
| PILOCARPINE NITRATE | 3.69 | Prestwick Chemical Inc. | BRD-K85090592 |  |
| PIMETHIXENE MALEATE | 2.44 | Prestwick Chemical Inc. | BRD-K88090157 |  |
| PIMOZIDE | 5.42 | Biomol International Inc. | BRD-K01292756 |  |
| PIMPINELLIN | 5.00 | MicroSource Discovery Systems Inc. | BRD-K93197368 |  |
| PINACIDIL | 10.19 | Biomol International Inc. | BRD-A43882281 |  |
| PINDOLOL | 4.03 | Prestwick Chemical Inc. | BRD-A97701745 |  |
| PINOCEMBRIN | 5.00 | MicroSource Discovery Systems Inc. | BRD-K94689771 |  |
| PINOSYLVIN | 5.00 | MicroSource Discovery Systems Inc. | BRD-K94645280 |  |
| PINOSYLVIN METHYL ETHER | 5.00 | MicroSource Discovery Systems Inc. | BRD-K18438502 |  |
| PIOGLITAZONE HYDROCHLORIDE | 5.00 | MicroSource Discovery Systems Inc. | BRD-A48430263 |  |
| PIPERACILLIN SODIUM SALT | 1.85 | Prestwick Chemical Inc. | BRD-K86873305 |  |
| PIPERIDOLATE HYDROCHLORIDE | 2.78 | Prestwick Chemical Inc. | BRD-A97479839 |  |
| PIPERLONGUMINE | 3.15 | Prestwick Chemical Inc. | BRD-K24132293 |  |
| PIPERONYLIC ACID | 5.00 | MicroSource Discovery Systems Inc. | BRD-K52148119 |  |
| PIRENPERONE | 2.54 | Prestwick Chemical Inc. | BRD-K25224017 |  |
| PIRENZEPINE DIHYDROCHLORIDE | 2.36 | Prestwick Chemical Inc. | BRD-K89375097 |  |
| PIRETANIDE | 2.76 | Prestwick Chemical Inc. | BRD-K87990216 |  |
| PIRIBEDIL | 5.00 | Biomol International Inc. | BRD-K47936004 |  |
| PIRIBEDIL HYDROCHLORIDE | 2.99 | Prestwick Chemical Inc. | BRD-K47936004 |  |
| PIRLINDOLE MESYLATE | 3.10 | Prestwick Chemical Inc. | BRD-A54490543 |  |
| PIROMIDIC ACID | 3.47 | Prestwick Chemical Inc. | BRD-K37682401 |  |
| PIVMECILLINAM HYDROCHLORIDE | 2.10 | Prestwick Chemical Inc. | BRD-K67100011 |  |
| PIZOTIFEN | 5.00 | Biomol International Inc. | BRD-K75958195 |  |
| PIZOTIFEN MALATE | 2.33 | Prestwick Chemical Inc. | BRD-K75958195 |  |
| PK-11195 | 5.00 | Biomol International Inc. | BRD-A41451487 |  |
| PLUMBAGIN | 5.00 | MicroSource Discovery Systems Inc. | BRD-K36137799 |  |
| PNU 22394 HCL | 5.00 | Biomol International Inc. | BRD-K16551401 |  |
| PNU 96415E | 5.00 | Biomol International Inc. | BRD-A95096829 |  |
| PODOPHYLLOTOXIN | 2.41 | Prestwick Chemical Inc. | BRD-K47869605 |  |
| PONALRESTAT | 5.00 | MicroSource Discovery Systems Inc. | BRD-K68332390 |  |
| PP1 | 8.89 | Biomol International Inc. | BRD-K47598052 |  |
| PRACTOLOL | 3.75 | Prestwick Chemical Inc. | BRD-A41304429 |  |
| PRAMOXINE HYDROCHLORIDE | 3.03 | Prestwick Chemical Inc. | BRD-K46523383 |  |
| PRAVASTATIN SODIUM | 5.00 | MicroSource Discovery Systems Inc. | BRD-A71816415 |  |
| PRAZIQUANTEL | 3.20 | Prestwick Chemical Inc. | BRD-A21858158 |  |
| PREDNICARBATE | 2.05 | Prestwick Chemical Inc. | BRD-K46137903 |  |
| PREDNISOLONE | 2.77 | Prestwick Chemical Inc. | BRD-K98039984 |  |
| PREDNISOLONE | 5.00 | MicroSource Discovery Systems Inc. | BRD-A27887842 |  |
| PREDNISOLONE ACETATE | 5.00 | MicroSource Discovery Systems Inc. | BRD-A01643550 |  |
| PREDNISONE | 2.79 | Prestwick Chemical Inc. | BRD-K85883481 |  |
| PREDNISONE | 5.00 | MicroSource Discovery Systems Inc. | BRD-A62525898 |  |
| PREGNENOLONE 16ALPHA CARBONITRILE | 7.32 | Biomol International Inc. | BRD-K39356024 |  |
| PRENYLETIN | 5.00 | MicroSource Discovery Systems Inc. | BRD-K81750404 |  |
| PRIDINOL METHANESULFONATE SALT | 2.55 | Prestwick Chemical Inc. | BRD-K17565903 |  |
| PRIEURANIN ACETATE | 5.00 | MicroSource Discovery Systems Inc. | BRD-A64374397 |  |
| PRILOCAINE HYDROCHLORIDE | 3.89 | Prestwick Chemical Inc. | BRD-A53952395 |  |
| PRIMIDONE | 5.00 | Biomol International Inc. | BRD-K32247306 |  |
| PROADIFEN HYDROCHLORIDE | 2.56 | Prestwick Chemical Inc. | BRD-K46317332 |  |
| PROBENECID | 3.50 | Prestwick Chemical Inc. | BRD-K95237249 |  |
| PROCAINAMIDE | 10.62 | Biomol International Inc. | BRD-K75089421 |  |
| PROCAINAMIDE HYDROCHLORIDE | 3.68 | Prestwick Chemical Inc. | BRD-K75089421 |  |
| PROCAINE HYDROCHLORIDE | 3.67 | Prestwick Chemical Inc. | BRD-K24616672 |  |
| PROCATEROL HYDROCHLORIDE | 5.00 | MicroSource Discovery Systems Inc. | BRD-A22684332 |  |
| PROCHLORPERAZINE DIMALEATE | 1.65 | Prestwick Chemical Inc. | BRD-K19352500 |  |
| PROCHLORPERAZINE EDISYLATE | 5.00 | MicroSource Discovery Systems Inc. | BRD-K19352500 |  |
| PROGESTERONE | 3.18 | Prestwick Chemical Inc. | BRD-K64994968 |  |
| PROGESTERONE | 5.00 | MicroSource Discovery Systems Inc. | BRD-A67479912 |  |
| PROGLUMIDE | 2.99 | Prestwick Chemical Inc. | BRD-A44863528 |  |
| PROGUANIL HYDROCHLORIDE | 3.45 | Prestwick Chemical Inc. | BRD-K28183345 |  |
| PROMETON | 5.00 | MicroSource Discovery Systems Inc. | BRD-K99029477 |  |
| PROMETRYN | 5.00 | MicroSource Discovery Systems Inc. | BRD-K32582260 |  |
| PRONETHALOL HYDROCHLORIDE | 3.76 | Prestwick Chemical Inc. | BRD-A87715314 |  |
| PROPACHLOR | 5.00 | MicroSource Discovery Systems Inc. | BRD-K50343025 |  |
| PROPAFENONE | 7.32 | Biomol International Inc. | BRD-A26334849 |  |
| PROPAFENONE HYDROCHLORIDE | 2.65 | Prestwick Chemical Inc. | BRD-A26334849 |  |
| PROPANIL | 5.00 | MicroSource Discovery Systems Inc. | BRD-K08618283 |  |
| PROPAZINE | 5.00 | MicroSource Discovery Systems Inc. | BRD-K37595074 |  |
| PROPENTOFYLLINE | 5.00 | MicroSource Discovery Systems Inc. | BRD-K59273480 |  |
| PROPIOMAZINE MALEATE | 5.00 | MicroSource Discovery Systems Inc. | BRD-A10471441 |  |
| PROPOFOL | 5.00 | Biomol International Inc. | BRD-K82255054 |  |
| PROPOXYCAINE HYDROCHLORIDE | 3.02 | Prestwick Chemical Inc. | BRD-K18250272 |  |
| PROPYL-BETA-CARBOLINE-3-CARBOXYLATE | 5.00 | Biomol International Inc. | BRD-K85242180 |  |
| PROPYLTHIOURACIL | 5.87 | Prestwick Chemical Inc. | BRD-K48168960 |  |
| PROSCILLARIDIN A | 1.88 | Prestwick Chemical Inc. | BRD-A34806832 |  |
| PROSTAGLANDIN A1 | 0.50 | Biomol International Inc. | BRD-K04010869 |  |
| PROSTAGLANDIN A2 | 0.50 | Biomol International Inc. | BRD-K34782918 |  |
| PROSTAGLANDIN B2 | 0.50 | Biomol International Inc. | BRD-K82865713 |  |
| PROTOVERATRINE A | 1.26 | Prestwick Chemical Inc. | BRD-A25875308 |  |
| PRUNETIN | 5.00 | MicroSource Discovery Systems Inc. | BRD-K57546357 |  |
| PSEUDOEPHEDRINE HYDROCHLORIDE | 5.00 | MicroSource Discovery Systems Inc. | BRD-K84175871 |  |
| PSEUDOPELLETIERINE HYDROCHLORIDE | 5.27 | Prestwick Chemical Inc. | BRD-A19053259 |  |
| PTERYXIN | 5.00 | MicroSource Discovery Systems Inc. | BRD-K92006759 |  |
| PUROMYCIN DIHYDROCHLORIDE | 1.84 | Prestwick Chemical Inc. | BRD-K36007650 |  |
| PUROMYCIN HYDROCHLORIDE | 5.00 | MicroSource Discovery Systems Inc. | BRD-A28970875 |  |
| PURPUROGALLIN | 5.00 | MicroSource Discovery Systems Inc. | BRD-K31023358 |  |
| PYRILAMINE MALEATE | 2.49 | Prestwick Chemical Inc. | BRD-K97564742 |  |
| PYRIMETHAMINE | 4.02 | Prestwick Chemical Inc. | BRD-K88429204 |  |
| PYRITHYLDIONE | 5.98 | Prestwick Chemical Inc. | BRD-K36116267 |  |
| QUERCETIN TETRAMETHYL (5,7,3',4') ETHER | 5.00 | MicroSource Discovery Systems Inc. | BRD-K08825053 |  |
| QUERCETINE DIHYDRATE | 2.96 | Prestwick Chemical Inc. | BRD-K97399794 |  |
| QUERCITRIN | 5.00 | MicroSource Discovery Systems Inc. | BRD-A76899420 |  |
| QUINACRINE DIHYDROCHLORIDE DIHYDRATE | 1.96 | Prestwick Chemical Inc. | BRD-A45889380 |  |
| QUINETHAZONE | 3.45 | Prestwick Chemical Inc. | BRD-A59303141 |  |
| QUINIDINE | 7.71 | Biomol International Inc. | BRD-A17470778 |  |
| QUINIDINE HYDROCHLORIDE MONOHYDRATE | 2.64 | Prestwick Chemical Inc. | BRD-K70799801 |  |
| QUININE | 7.71 | Biomol International Inc. | BRD-A00821662 |  |
| QUININE SULFATE | 5.00 | MicroSource Discovery Systems Inc. | BRD-K83041223 |  |
| QUIPAZINE | 5.00 | Biomol International Inc. | BRD-K77925998 |  |
| QUIPAZINE DIMALEATE SALT | 2.25 | Prestwick Chemical Inc. | BRD-K77925998 |  |
| QUIPAZINE MALEATE | 5.00 | MicroSource Discovery Systems Inc. | BRD-K77925998 |  |
| QX-314 | 7.26 | Biomol International Inc. | BRD-K56596464 |  |
| R(+)-6-BROMO-APB | 5.00 | Biomol International Inc. | BRD-K60932973 |  |
| R(+)-SKF-81297 | 5.00 | Biomol International Inc. | BRD-K84421793 |  |
| R(-)-2,10,11-TRIHYDROXY-N-PROPYL-NORAPORPHINE | 5.00 | Biomol International Inc. | BRD-K16604360 |  |
| R(-)-2,10,11-TRIHYDROXYAPORPHINE | 5.00 | Biomol International Inc. | BRD-K39187410 |  |
| R(-)-APOMORPHINE | 5.00 | Biomol International Inc. | BRD-K76022557 |  |
| R(-)-N-ALLYLNORAPOMORPHINE | 5.00 | Biomol International Inc. | BRD-K07079548 |  |
| R(-)-PROPYLNORAPOMORPHINE | 5.00 | Biomol International Inc. | BRD-K13544237 |  |
| RACECADOTRIL | 2.59 | Prestwick Chemical Inc. | BRD-A08187463 |  |
| RACEPHEDRINE HYDROCHLORIDE | 5.00 | MicroSource Discovery Systems Inc. | BRD-A54236247 |  |
| RAMELTEON | 5.00 | MicroSource Discovery Systems Inc. | BRD-K28761891 |  |
| RAMIPRIL | 2.40 | Prestwick Chemical Inc. | BRD-A65739223 |  |
| RANITIDINE HYDROCHLORIDE | 2.85 | Prestwick Chemical Inc. | BRD-K14204120 |  |
| RANOLAZINE | 5.00 | MicroSource Discovery Systems Inc. | BRD-A97674275 |  |
| RAPAMYCIN | 5.00 | Biomol International Inc. | BRD-K89366998 |  |
| RAUWOLSCINE HYDROCHLORIDE | 2.56 | Prestwick Chemical Inc. | BRD-K77474816 |  |
| REBAMIPIDE | 5.00 | MicroSource Discovery Systems Inc. | BRD-A15909516 |  |
| REMOXIPRIDE | 5.00 | Biomol International Inc. | BRD-K54094468 |  |
| REMOXIPRIDE HYDROCHLORIDE | 2.45 | Prestwick Chemical Inc. | BRD-K54094468 |  |
| REPAGLINIDE | 2.21 | Prestwick Chemical Inc. | BRD-K82846253 |  |
| RESCINNAMIN | 1.58 | Prestwick Chemical Inc. | BRD-K52930707 |  |
| RESERPINIC ACID HYDROCHLORIDE | 2.29 | Prestwick Chemical Inc. | BRD-K32755366 |  |
| RESVERATROL | 10.95 | Biomol International Inc. | BRD-K80738081 |  |
| RETRORSINE | 2.85 | Prestwick Chemical Inc. | BRD-K42142750 |  |
| RETUSIN 7-METHYL ETHER | 5.00 | MicroSource Discovery Systems Inc. | BRD-K59294488 |  |
| REV-5901 | 0.50 | Biomol International Inc. | BRD-A68281735 |  |
| RHETSININE | 5.00 | MicroSource Discovery Systems Inc. | BRD-K08814982 |  |
| RHOIFOLIN | 5.00 | MicroSource Discovery Systems Inc. | BRD-K23327891 |  |
| RIBOFLAVIN | 5.00 | MicroSource Discovery Systems Inc. | BRD-K70246307 |  |
| RICININE | 6.09 | Prestwick Chemical Inc. | BRD-K82561139 |  |
| RIFABUTIN | 1.18 | Prestwick Chemical Inc. | BRD-A23801136 |  |
| RIFAMPICIN | 1.22 | Prestwick Chemical Inc. | BRD-K48516106 |  |
| RILUZOLE | 5.00 | Biomol International Inc. | BRD-K21283037 |  |
| RILUZOLE HYDROCHLORIDE | 3.69 | Prestwick Chemical Inc. | BRD-K21283037 |  |
| RIMEXOLONE | 2.70 | Prestwick Chemical Inc. | BRD-K31627533 |  |
| RISPERIDONE | 5.00 | Biomol International Inc. | BRD-K53857191 |  |
| RITANSERIN | 5.00 | MicroSource Discovery Systems Inc. | BRD-K40887525 |  |
| RITODRINE HYDROCHLORIDE | 3.09 | Prestwick Chemical Inc. | BRD-K51465424 |  |
| RITODRINE HYDROCHLORIDE | 5.00 | MicroSource Discovery Systems Inc. | BRD-A59174698 |  |
| RO 04-6790 | 5.00 | Biomol International Inc. | BRD-K43290182 |  |
| RO 20-1724 | 8.98 | Biomol International Inc. | BRD-A07207424 |  |
| RO 31-8220 | 5.46 | Biomol International Inc. | BRD-K06543683 |  |
| ROBUSTIC ACID | 5.00 | MicroSource Discovery Systems Inc. | BRD-K54411430 |  |
| ROBUSTONE | 5.00 | MicroSource Discovery Systems Inc. | BRD-K16117851 |  |
| ROFECOXIB | 5.00 | MicroSource Discovery Systems Inc. | BRD-K21733600 |  |
| ROLIPRAM | 9.08 | Biomol International Inc. | BRD-A34255068 |  |
| ROPINIROLE HCL | 5.00 | Biomol International Inc. | BRD-K15933101 |  |
| ROSIGLITAZONE | 5.00 | MicroSource Discovery Systems Inc. | BRD-A97437073 |  |
| ROSOLIC ACID | 5.00 | MicroSource Discovery Systems Inc. | BRD-K24453427 |  |
| RS 23597-190 | 5.00 | Biomol International Inc. | BRD-K01868942 |  |
| RS 39604 | 5.00 | Biomol International Inc. | BRD-K20742498 |  |
| RS 56812 | 5.00 | Biomol International Inc. | BRD-K20714604 |  |
| S(+)-TERGURIDE | 5.00 | Biomol International Inc. | BRD-K05901394 |  |
| S(-)-LISURIDE | 5.00 | Biomol International Inc. | BRD-K88871508 |  |
| S(-)-RACLOPRIDE | 5.00 | Biomol International Inc. | BRD-K04111260 |  |
| S(-)-SULPIRIDE | 5.00 | Biomol International Inc. | BRD-K51671335 |  |
| S(-)ETICLOPRIDE HYDROCHLORIDE | 2.65 | Prestwick Chemical Inc. | BRD-K50417881 |  |
| S,S,S,-TRIBUTYLPHOSPHOROTRITHIOATE | 5.00 | MicroSource Discovery Systems Inc. | BRD-K02837237 |  |
| S-FARNESYL-L-CYSTEINE | 0.50 | Biomol International Inc. | BRD-K38756014 |  |
| SACLOFEN | 5.00 | Biomol International Inc. | BRD-A24122750 |  |
| SALBUTAMOL | 4.18 | Prestwick Chemical Inc. | BRD-A88254928 |  |
| SALICYL ALCOHOL | 5.00 | MicroSource Discovery Systems Inc. | BRD-K08493205 |  |
| SALICYLAMIDE | 5.00 | MicroSource Discovery Systems Inc. | BRD-K81130846 |  |
| SALSOLIDINE | 5.00 | MicroSource Discovery Systems Inc. | BRD-A75140635 |  |
| SALSOLINE | 5.00 | MicroSource Discovery Systems Inc. | BRD-A48323445 |  |
| SAPPANONE A DIMETHYL ETHER | 5.00 | MicroSource Discovery Systems Inc. | BRD-K32857660 |  |
| SAQUINAVIR MESYLATE | 1.30 | Prestwick Chemical Inc. | BRD-K09963420 |  |
| SB 202190 | 7.55 | Biomol International Inc. | BRD-K54330070 |  |
| SB 216641 | 5.00 | Biomol International Inc. | BRD-K30867024 |  |
| SB-415286 | 6.95 | Biomol International Inc. | BRD-K76805682 |  |
| SB-431542 | 6.50 | Biomol International Inc. | BRD-K67298865 |  |
| SCH 23390 | 5.00 | Biomol International Inc. | BRD-K45435259 |  |
| SCOPOLAMIN-N-OXIDE HYDROBROMIDE | 2.50 | Prestwick Chemical Inc. | BRD-A49906757 |  |
| SCOPOLETIN | 5.20 | Prestwick Chemical Inc. | BRD-K96163925 |  |
| SCOULERINE | 3.05 | Prestwick Chemical Inc. | BRD-K62609077 |  |
| SDZ 205,557 | 5.00 | Biomol International Inc. | BRD-K15868788 |  |
| SDZ SER 082 | 5.00 | Biomol International Inc. | BRD-K31339597 |  |
| SDZ-201106 | 5.36 | Biomol International Inc. | BRD-A64553394 |  |
| SECNIDAZOLE | 5.00 | MicroSource Discovery Systems Inc. | BRD-A70083328 |  |
| SECURININE | 5.00 | MicroSource Discovery Systems Inc. | BRD-K49071277 |  |
| SELAMECTIN | 5.00 | MicroSource Discovery Systems Inc. | BRD-A40622617 |  |
| SELEGILINE HYDROCHLORIDE | 4.47 | Prestwick Chemical Inc. | BRD-K86434416 |  |
| SELINIDIN | 5.00 | MicroSource Discovery Systems Inc. | BRD-K07212038 |  |
| SENECIPHYLLINE | 3.00 | Prestwick Chemical Inc. | BRD-K38449220 |  |
| SERICETIN DIACETATE | 5.00 | MicroSource Discovery Systems Inc. | BRD-K91347490 |  |
| SERICETIN DIMETHYL ETHER | 5.00 | MicroSource Discovery Systems Inc. | BRD-K08714182 |  |
| SERTACONAZOLE NITRATE | 2.00 | Prestwick Chemical Inc. | BRD-A95939040 |  |
| SERTRALINE HYDROCHLORIDE | 5.00 | MicroSource Discovery Systems Inc. | BRD-K82036761 |  |
| SIGUAZODAN | 8.79 | Biomol International Inc. | BRD-A62071884 |  |
| SILDENAFIL | 5.00 | MicroSource Discovery Systems Inc. | BRD-K79759585 |  |
| SILIBININ | 5.00 | MicroSource Discovery Systems Inc. | BRD-K80353138 |  |
| SIMAZINE | 5.00 | MicroSource Discovery Systems Inc. | BRD-K01416914 |  |
| SINAPIC ACID METHYL ETHER | 5.00 | MicroSource Discovery Systems Inc. | BRD-K63758740 |  |
| SINOMENINE | 5.00 | MicroSource Discovery Systems Inc. | BRD-K83459933 |  |
| SITAGLIPTIN | 5.00 | MicroSource Discovery Systems Inc. | BRD-K19416115 |  |
| SKF 38393 | 5.00 | Biomol International Inc. | BRD-A88548664 |  |
| SKF 89976A | 5.00 | Biomol International Inc. | BRD-A49046702 |  |
| SKF-96365 | 6.82 | Biomol International Inc. | BRD-A72703248 |  |
| SKIMMIANINE | 3.86 | Prestwick Chemical Inc. | BRD-K25741894 |  |
| SODIUM DEHYDROCHOLATE | 5.00 | MicroSource Discovery Systems Inc. | BRD-A64125466 |  |
| SOLANINE ALPHA | 1.15 | Prestwick Chemical Inc. | BRD-K70881766 |  |
| SOLIDAGENONE | 5.00 | MicroSource Discovery Systems Inc. | BRD-A05666893 |  |
| SOTALOL HYDROCHLORIDE | 3.24 | Prestwick Chemical Inc. | BRD-A33168282 |  |
| SPHONDIN | 5.00 | MicroSource Discovery Systems Inc. | BRD-K33260002 |  |
| SPIPERONE | 2.53 | Prestwick Chemical Inc. | BRD-K55468218 |  |
| SPIRONOLACTONE | 2.40 | Prestwick Chemical Inc. | BRD-K90027355 |  |
| SPIROXATRINE | 5.00 | Biomol International Inc. | BRD-A72483914 |  |
| SPLITOMYCIN | 12.61 | Biomol International Inc. | BRD-K27710560 |  |
| SR 57227A | 5.00 | Biomol International Inc. | BRD-K09397065 |  |
| SR-95639A | 2.52 | Prestwick Chemical Inc. | BRD-K19309090 |  |
| STAUROSPORINE | 5.00 | Biomol International Inc. | BRD-K70549064 |  |
| STAUROSPORINE | 5.36 | Biomol International Inc. | BRD-K17953061 |  |
| STICTIC ACID | 5.00 | MicroSource Discovery Systems Inc. | BRD-A29578214 |  |
| STROPHANTHIDIN | 2.47 | Prestwick Chemical Inc. | BRD-K84595254 |  |
| STROPHANTINE OCTAHYDRATE | 1.37 | Prestwick Chemical Inc. | BRD-K35708212 |  |
| SUCCINYLSULFATHIAZOLE | 2.81 | Prestwick Chemical Inc. | BRD-K01950558 |  |
| SULFABENZAMIDE | 3.62 | Prestwick Chemical Inc. | BRD-K59983611 |  |
| SULFACHLOROPYRIDAZINE | 3.51 | Prestwick Chemical Inc. | BRD-K32021043 |  |
| SULFADIAZINE | 4.00 | Prestwick Chemical Inc. | BRD-K32273377 |  |
| SULFADIMETHOXINE | 3.22 | Prestwick Chemical Inc. | BRD-K71125014 |  |
| SULFADOXINE | 3.22 | Prestwick Chemical Inc. | BRD-K55250441 |  |
| SULFAMERAZINE | 3.78 | Prestwick Chemical Inc. | BRD-K93524252 |  |
| SULFAMETER | 3.57 | Prestwick Chemical Inc. | BRD-K87492696 |  |
| SULFAMETHAZINE | 5.00 | MicroSource Discovery Systems Inc. | BRD-K11640013 |  |
| SULFAMETHAZINE SODIUM SALT | 3.32 | Prestwick Chemical Inc. | BRD-K11640013 |  |
| SULFAMETHIZOLE | 3.70 | Prestwick Chemical Inc. | BRD-K31682896 |  |
| SULFAMETHOXAZOLE | 3.95 | Prestwick Chemical Inc. | BRD-K28494619 |  |
| SULFAMETHOXYPYRIDAZINE | 3.57 | Prestwick Chemical Inc. | BRD-K00938507 |  |
| SULFANITRAN | 5.00 | MicroSource Discovery Systems Inc. | BRD-K76845197 |  |
| SULFAPHENAZOLE | 3.18 | Prestwick Chemical Inc. | BRD-K10671814 |  |
| SULFAPYRIDINE | 4.01 | Prestwick Chemical Inc. | BRD-K41406082 |  |
| SULFAQUINOXALINE SODIUM SALT | 3.09 | Prestwick Chemical Inc. | BRD-K71133585 |  |
| SULFASALAZINE | 2.51 | Prestwick Chemical Inc. | BRD-K10670311 |  |
| SULFATHIAZOLE | 3.92 | Prestwick Chemical Inc. | BRD-K14705039 |  |
| SULFINPYRAZONE | 2.47 | Prestwick Chemical Inc. | BRD-A36217750 |  |
| SULFISOXAZOLE | 3.74 | Prestwick Chemical Inc. | BRD-K50859149 |  |
| SULINDAC | 2.81 | Prestwick Chemical Inc. | BRD-A13946108 |  |
| SULMAZOLE | 3.48 | Prestwick Chemical Inc. | BRD-A22081593 |  |
| SULOCTIDIL | 2.96 | Prestwick Chemical Inc. | BRD-K42635745 |  |
| SULPIRIDE | 5.00 | MicroSource Discovery Systems Inc. | BRD-A10715913 |  |
| SUMATRIPTAN | 5.00 | MicroSource Discovery Systems Inc. | BRD-K50938287 |  |
| SUPROFEN METHYL ESTER | 5.00 | MicroSource Discovery Systems Inc. | BRD-A11154023 |  |
| SUXIBUZONE | 2.28 | Prestwick Chemical Inc. | BRD-K78815826 |  |
| SYRINGIC ACID | 5.00 | MicroSource Discovery Systems Inc. | BRD-K51980294 |  |
| SYROSINGOPINE | 1.50 | Prestwick Chemical Inc. | BRD-K14200658 |  |
| TACRINE HYDROCHLORIDE HYDRATE | 4.26 | Prestwick Chemical Inc. | BRD-K81473089 |  |
| TAMOXIFEN CITRATE | 1.77 | Prestwick Chemical Inc. | BRD-K93754473 |  |
| TANSHINONE IIA | 8.49 | Biomol International Inc. | BRD-K00141480 |  |
| TEBUTHIURON | 5.00 | MicroSource Discovery Systems Inc. | BRD-K34820100 |  |
| TEGASEROD | 5.00 | MicroSource Discovery Systems Inc. | BRD-K88743730 |  |
| TELENZEPINE DIHYDROCHLORIDE | 2.26 | Prestwick Chemical Inc. | BRD-K06147391 |  |
| TELENZEPINE HYDROCHLORIDE | 5.00 | MicroSource Discovery Systems Inc. | BRD-K06147391 |  |
| TELMISARTAN | 5.00 | MicroSource Discovery Systems Inc. | BRD-K73999723 |  |
| TENIPOSIDE | 5.00 | MicroSource Discovery Systems Inc. | BRD-A35588707 |  |
| TENOXICAM | 2.96 | Prestwick Chemical Inc. | BRD-A22844106 |  |
| TERAZOSIN HYDROCHLORIDE | 2.36 | Prestwick Chemical Inc. | BRD-A22256192 |  |
| TERBUTALINE HEMISULFATE | 5.00 | MicroSource Discovery Systems Inc. | BRD-A50157456 |  |
| TERCONAZOLE | 1.88 | Prestwick Chemical Inc. | BRD-K86204871 |  |
| TERFENADINE | 2.12 | Prestwick Chemical Inc. | BRD-A06352418 |  |
| TESTOSTERONE | 5.00 | MicroSource Discovery Systems Inc. | BRD-A55393291 |  |
| TESTOSTERONE PROPIONATE | 2.90 | Prestwick Chemical Inc. | BRD-K90553655 |  |
| TESTOSTERONE PROPIONATE | 5.00 | MicroSource Discovery Systems Inc. | BRD-A48720949 |  |
| TETRAC | 5.00 | MicroSource Discovery Systems Inc. | BRD-K71437774 |  |
| TETRACAINE HYDROCHLORIDE | 3.32 | Prestwick Chemical Inc. | BRD-K45071273 |  |
| TETRAHYDROCANNABINOL-7-OIC ACID | 0.50 | Biomol International Inc. | BRD-A42831637 |  |
| TETRAHYDROCORTISONE-3,21-DIACETATE | 5.00 | MicroSource Discovery Systems Inc. | BRD-A03503284 |  |
| TETRAHYDROPALMATINE | 5.00 | MicroSource Discovery Systems Inc. | BRD-A43940795 |  |
| TETRAHYDROTRIMETHYLHISPIDIN | 5.00 | MicroSource Discovery Systems Inc. | BRD-A86977540 |  |
| TETRAHYDROZOLINE HYDROCHLORIDE | 4.22 | Prestwick Chemical Inc. | BRD-A28856712 |  |
| TETRANDRINE | 4.01 | Biomol International Inc. | BRD-K08078237 |  |
| THAPSIGARGIN | 3.84 | Biomol International Inc. | BRD-K69023402 |  |
| THEOPHYLLINE | 5.00 | MicroSource Discovery Systems Inc. | BRD-K97799481 |  |
| THIAMPHENICOL | 2.81 | Prestwick Chemical Inc. | BRD-K79711234 |  |
| THIAMYLAL SODIUM | 5.00 | MicroSource Discovery Systems Inc. | BRD-A23970436 |  |
| THIMEROSAL | 5.00 | MicroSource Discovery Systems Inc. | BRD-K61443650 |  |
| THIOCOLCHICOSIDE | 1.77 | Prestwick Chemical Inc. | BRD-A11605036 |  |
| THIOPROPERAZINE DIMESYLATE | 1.57 | Prestwick Chemical Inc. | BRD-K08619574 |  |
| THIOTHIXENE | 5.00 | MicroSource Discovery Systems Inc. | BRD-K97309399 |  |
| THONZYLAMINE HYDROCHLORIDE | 5.00 | MicroSource Discovery Systems Inc. | BRD-K88405679 |  |
| THYROXINE (L) | 1.29 | Prestwick Chemical Inc. | BRD-K30685142 |  |
| TIABENDAZOLE | 4.97 | Prestwick Chemical Inc. | BRD-K77695569 |  |
| TIAPRIDE HYDROCHLORIDE | 2.74 | Prestwick Chemical Inc. | BRD-K57432881 |  |
| TIAPROFENIC ACID | 3.84 | Prestwick Chemical Inc. | BRD-A72988804 |  |
| TICLOPIDINE HYDROCHLORIDE | 3.33 | Prestwick Chemical Inc. | BRD-K00603606 |  |
| TIMOLOL MALEATE | 5.00 | MicroSource Discovery Systems Inc. | BRD-K30421593 |  |
| TINIDAZOLE | 4.04 | Prestwick Chemical Inc. | BRD-K89125793 |  |
| TIOXOLONE | 5.00 | MicroSource Discovery Systems Inc. | BRD-K41876534 |  |
| TIRATRICOL, 3,3',5-TRIIODOTHYROACETIC ACID | 1.61 | Prestwick Chemical Inc. | BRD-K61236135 |  |
| TMB-8 | 6.32 | Biomol International Inc. | BRD-K58220566 |  |
| TOCAINIDE HYDROCHLORIDE | 4.37 | Prestwick Chemical Inc. | BRD-A92670106 |  |
| TODRALAZINE HYDROCHLORIDE | 3.72 | Prestwick Chemical Inc. | BRD-K68553471 |  |
| TOLAZAMIDE | 8.03 | Biomol International Inc. | BRD-K32164935 |  |
| TOLAZOLINE HYDROCHLORIDE | 5.08 | Prestwick Chemical Inc. | BRD-K46211610 |  |
| TOLBUTAMIDE | 9.25 | Biomol International Inc. | BRD-K85119730 |  |
| TOLFENAMIC ACID | 3.82 | Prestwick Chemical Inc. | BRD-K50133271 |  |
| TOLMETIN SODIUM | 5.00 | MicroSource Discovery Systems Inc. | BRD-K82562631 |  |
| TOLNAFTATE | 3.25 | Prestwick Chemical Inc. | BRD-K44273375 |  |
| TOLPERISONE HYDROCHLORIDE | 5.00 | MicroSource Discovery Systems Inc. | BRD-A27732521 |  |
| TORSEMIDE | 2.87 | Prestwick Chemical Inc. | BRD-K30480208 |  |
| TOSYL-PHE-CMK (TPCK) | 7.11 | Biomol International Inc. | BRD-K07055988 |  |
| TOTAROL | 5.00 | MicroSource Discovery Systems Inc. | BRD-A62129005 |  |
| TOTAROL ACETATE | 5.00 | MicroSource Discovery Systems Inc. | BRD-A94366206 |  |
| TRACAZOLATE HYDROCHLORIDE | 2.93 | Prestwick Chemical Inc. | BRD-K34154330 |  |
| TRANDOLAPRIL | 5.00 | MicroSource Discovery Systems Inc. | BRD-A32505112 |  |
| TRANS-7-HYDROXY-PIPAT | 5.00 | Biomol International Inc. | BRD-A01295252 |  |
| TRANYLCYPROMINE HYDROCHLORIDE | 5.89 | Prestwick Chemical Inc. | BRD-K88809146 |  |
| TRANYLCYPROMINE SULFATE | 5.00 | MicroSource Discovery Systems Inc. | BRD-A43974575 |  |
| TRAPIDIL | 4.87 | Prestwick Chemical Inc. | BRD-K95763993 |  |
| TRAZODONE HYDROCHLORIDE | 2.45 | Prestwick Chemical Inc. | BRD-K70778732 |  |
| TREQUINSIN | 6.17 | Biomol International Inc. | BRD-K84663978 |  |
| TRIACETYLRESVERATROL | 5.00 | MicroSource Discovery Systems Inc. | BRD-K80946661 |  |
| TRIADIMEFON | 5.00 | MicroSource Discovery Systems Inc. | BRD-A39506880 |  |
| TRIALLATE | 5.00 | MicroSource Discovery Systems Inc. | BRD-K64698045 |  |
| TRIAMCINOLONE | 2.54 | Prestwick Chemical Inc. | BRD-K77554836 |  |
| TRIAMCINOLONE ACETONIDE | 5.00 | MicroSource Discovery Systems Inc. | BRD-A92439610 |  |
| TRIAMTERENE | 3.95 | Prestwick Chemical Inc. | BRD-K92049597 |  |
| TRIFLUOPERAZINE | 5.00 | Biomol International Inc. | BRD-K89732114 |  |
| TRIFLUOPERAZINE DIHYDROCHLORIDE | 2.08 | Prestwick Chemical Inc. | BRD-K89732114 |  |
| TRIFLURIDINE | 3.38 | Prestwick Chemical Inc. | BRD-A64485570 |  |
| TRIHEXYPHENIDYL-D,L HYDROCHLORIDE | 2.96 | Prestwick Chemical Inc. | BRD-A48180038 |  |
| TRIM | 11.78 | Biomol International Inc. | BRD-K36851334 |  |
| TRIMETAZIDINE DIHYDROCHLORIDE | 2.95 | Prestwick Chemical Inc. | BRD-K88366685 |  |
| TRIMETHOBENZAMIDE HYDROCHLORIDE | 2.35 | Prestwick Chemical Inc. | BRD-K34415467 |  |
| TRIMETHOPRIM | 3.44 | Prestwick Chemical Inc. | BRD-K07208025 |  |
| TRIMETHYLCOLCHICINIC ACID | 2.91 | Prestwick Chemical Inc. | BRD-K84300594 |  |
| TRIMIPRAMINE MALEATE SALT | 2.44 | Prestwick Chemical Inc. | BRD-A19195498 |  |
| TRIOXSALEN | 4.38 | Prestwick Chemical Inc. | BRD-K54790157 |  |
| TRIPELENNAMINE CITRATE | 5.00 | MicroSource Discovery Systems Inc. | BRD-K57033106 |  |
| TRIPROLIDINE HYDROCHLORIDE | 3.18 | Prestwick Chemical Inc. | BRD-K11742128 |  |
| TRIPTOLIDE | 6.94 | Biomol International Inc. | BRD-K39484304 |  |
| TRIPTOPHENOLIDE | 5.00 | MicroSource Discovery Systems Inc. | BRD-K73210450 |  |
| TROLOX | 4.00 | Prestwick Chemical Inc. | BRD-A17846016 |  |
| TROPANYL 3,5-DIMETHYLBENZOATE | 5.00 | Biomol International Inc. | BRD-A84389633 |  |
| TROPICAMIDE | 3.52 | Prestwick Chemical Inc. | BRD-A79672927 |  |
| TROPISETRON | 5.00 | Biomol International Inc. | BRD-A83859836 |  |
| TROPISETRON HCL | 3.12 | Prestwick Chemical Inc. | BRD-A79226577 |  |
| TROXERUTIN | 5.00 | MicroSource Discovery Systems Inc. | BRD-A74160776 |  |
| TTNPB | 0.50 | Biomol International Inc. | BRD-K49685476 |  |
| TUBAIC ACID | 5.00 | MicroSource Discovery Systems Inc. | BRD-A02176148 |  |
| TUBOCURARINE CHLORIDE PENTAHYDRATE (+) | 1.29 | Prestwick Chemical Inc. | BRD-K02607075 |  |
| TULOBUTEROL | 5.00 | MicroSource Discovery Systems Inc. | BRD-A37441042 |  |
| TYRPHOSTIN 1 | 13.57 | Biomol International Inc. | BRD-K41996876 |  |
| TYRPHOSTIN 46 | 5.00 | Biomol International Inc. | BRD-K60184833 |  |
| U-0126 | 5.00 | Biomol International Inc. | BRD-K18787491 |  |
| U-50488 | 6.77 | Biomol International Inc. | BRD-K53532120 |  |
| U-74389G | 4.09 | Biomol International Inc. | BRD-A96897502 |  |
| U-99194A | 5.00 | Biomol International Inc. | BRD-K70281171 |  |
| UMBELLIFERONE | 5.00 | MicroSource Discovery Systems Inc. | BRD-K87991767 |  |
| UNGERINE NITRATE | 2.55 | Prestwick Chemical Inc. | BRD-A96820077 |  |
| URSINOIC ACID | 5.00 | MicroSource Discovery Systems Inc. | BRD-K92405402 |  |
| URSOLIC ACID | 2.19 | Prestwick Chemical Inc. | BRD-K68185022 |  |
| VALACYCLOVIR HYDROCHLORIDE | 5.00 | MicroSource Discovery Systems Inc. | BRD-K46435977 |  |
| VALDECOXIB | 5.00 | MicroSource Discovery Systems Inc. | BRD-K12994359 |  |
| VALSARTAN | 5.00 | MicroSource Discovery Systems Inc. | BRD-K45158365 |  |
| VALYLTRYPTOPHAN | 5.00 | MicroSource Discovery Systems Inc. | BRD-K46272620 |  |
| VARDENAFIL HYDROCHLORIDE | 5.00 | MicroSource Discovery Systems Inc. | BRD-K13926615 |  |
| VENLAFAXINE | 5.00 | MicroSource Discovery Systems Inc. | BRD-A51714012 |  |
| VENLAFAXINE HCL | 5.00 | Biomol International Inc. | BRD-A51714012 |  |
| VERAPAMIL | 5.50 | Biomol International Inc. | BRD-A09533288 |  |
| VERAPAMYL HYDROCHLORIDE | 2.04 | Prestwick Chemical Inc. | BRD-A09533288 |  |
| VERATRIC ACID | 5.00 | MicroSource Discovery Systems Inc. | BRD-K14520048 |  |
| VERATRIDINE | 3.71 | Biomol International Inc. | BRD-K60923938 |  |
| VESAMICOL HYDROCHLORIDE | 5.00 | MicroSource Discovery Systems Inc. | BRD-A76904477 |  |
| VINBLASTINE | 2.75 | Biomol International Inc. | BRD-K01188359 |  |
| VINCAMINE | 2.82 | Prestwick Chemical Inc. | BRD-K40902647 |  |
| VINPOCETINE | 2.85 | Prestwick Chemical Inc. | BRD-K53318339 |  |
| VITEXIN | 2.31 | Prestwick Chemical Inc. | BRD-A41941932 |  |
| VULPINIC ACID | 5.00 | MicroSource Discovery Systems Inc. | BRD-A89237309 |  |
| WARFARIN | 5.00 | MicroSource Discovery Systems Inc. | BRD-A24514565 |  |
| WIN 55,212-2 | 0.50 | Biomol International Inc. | BRD-K88282786 |  |
| WISKOSTATIN | 5.87 | Biomol International Inc. | BRD-A18579359 |  |
| WY-14643 | 0.50 | Biomol International Inc. | BRD-K01902415 |  |
| XANTHOXYLIN | 5.00 | MicroSource Discovery Systems Inc. | BRD-K12260308 |  |
| XYLAZINE | 4.54 | Prestwick Chemical Inc. | BRD-K21565985 |  |
| Y-25130 | 5.00 | Biomol International Inc. | BRD-A44133049 |  |
| YOHIMBIC ACID | 5.00 | MicroSource Discovery Systems Inc. | BRD-K37282009 |  |
| YOHIMBINE HYDROCHLORIDE | 2.56 | Prestwick Chemical Inc. | BRD-K35586044 |  |
| YOHIMBINE HYDROCHLORIDE | 5.00 | MicroSource Discovery Systems Inc. | BRD-A87445400 |  |
| YOHIMBINIC ACID MONOHYDRATE | 2.79 | Prestwick Chemical Inc. | BRD-K37282009 |  |
| YS035 | 6.95 | Biomol International Inc. | BRD-K06208435 |  |
| Z-PROLYL-PROLINAL | 7.57 | Biomol International Inc. | BRD-K60174629 |  |
| ZAPRINAST | 9.22 | Biomol International Inc. | BRD-K16542329 |  |
| ZARDAVERINE | 9.32 | Biomol International Inc. | BRD-K37561857 |  |
| ZEARALENONE | 5.00 | MicroSource Discovery Systems Inc. | BRD-K42017082 |  |
| ZIDOVUDINE, AZT | 3.74 | Prestwick Chemical Inc. | BRD-K72903603 |  |
| ZM226600 | 6.70 | Biomol International Inc. | BRD-A62209527 |  |
| ZM336372 | 6.42 | Biomol International Inc. | BRD-K73789395 |  |
| ZOLPIDEM | 5.00 | MicroSource Discovery Systems Inc. | BRD-K44876623 |  |
| ZOMEPIRAC SODIUM | 5.00 | MicroSource Discovery Systems Inc. | BRD-K81326768 |  |
| ZOMEPIRAC SODIUM SALT | 3.18 | Prestwick Chemical Inc. | BRD-K81326768 |  |
| ZOXAZOLAMINE | 5.93 | Prestwick Chemical Inc. | BRD-K66353228 |  |
