# Supplementary material for: Multiplex Cytological Profiling Assay to Measure Diverse Cellular States
Source: PLoS One. 2013 Dec 2;8(12):e80999. doi: 10.1371/journal.pone.0080999 (PMC3847047; doi:10.1371/journal.pone.0080999)
Supplement: Table S2 — Image features measured for each cell by CellProfiler (see the CellProfiler manual for descriptions of each feature). (DOC) [file pone.0080999.s010.doc]

**Table S2:** Image features measured for each cell by CellProfiler (see the CellProfiler manual for descriptions of each feature).

| Measurement name |
| --- |
| Nuclei_AreaShape_Area |
| Nuclei_AreaShape_Eccentricity |
| Nuclei_AreaShape_EulerNumber |
| Nuclei_AreaShape_Extent |
| Nuclei_AreaShape_FormFactor |
| Nuclei_AreaShape_MajorAxisLength |
| Nuclei_AreaShape_MinorAxisLength |
| Nuclei_AreaShape_Orientation |
| Nuclei_AreaShape_Perimeter |
| Nuclei_AreaShape_Solidity |
| Nuclei_AreaShape_Zernike_0_0 |
| Nuclei_AreaShape_Zernike_1_1 |
| Nuclei_AreaShape_Zernike_2_0 |
| Nuclei_AreaShape_Zernike_2_2 |
| Nuclei_AreaShape_Zernike_3_1 |
| Nuclei_AreaShape_Zernike_3_3 |
| Nuclei_AreaShape_Zernike_4_0 |
| Nuclei_AreaShape_Zernike_4_2 |
| Nuclei_AreaShape_Zernike_4_4 |
| Nuclei_AreaShape_Zernike_5_1 |
| Nuclei_AreaShape_Zernike_5_3 |
| Nuclei_AreaShape_Zernike_5_5 |
| Nuclei_AreaShape_Zernike_6_0 |
| Nuclei_AreaShape_Zernike_6_2 |
| Nuclei_AreaShape_Zernike_6_4 |
| Nuclei_AreaShape_Zernike_6_6 |
| Nuclei_AreaShape_Zernike_7_1 |
| Nuclei_AreaShape_Zernike_7_3 |
| Nuclei_AreaShape_Zernike_7_5 |
| Nuclei_AreaShape_Zernike_7_7 |
| Nuclei_AreaShape_Zernike_8_0 |
| Nuclei_AreaShape_Zernike_8_2 |
| Nuclei_AreaShape_Zernike_8_4 |
| Nuclei_AreaShape_Zernike_8_6 |
| Nuclei_AreaShape_Zernike_8_8 |
| Nuclei_AreaShape_Zernike_9_1 |
| Nuclei_AreaShape_Zernike_9_3 |
| Nuclei_AreaShape_Zernike_9_5 |
| Nuclei_AreaShape_Zernike_9_7 |
| Nuclei_AreaShape_Zernike_9_9 |
| Nuclei_Children_Cells_Count |
| Nuclei_Children_Cytoplasm_Count |
| Nuclei_Intensity_IntegratedIntensityEdge_ER |
| Nuclei_Intensity_IntegratedIntensityEdge_Hoechst |
| Nuclei_Intensity_IntegratedIntensityEdge_Mito |
| Nuclei_Intensity_IntegratedIntensityEdge_Ph_golgi |
| Nuclei_Intensity_IntegratedIntensityEdge_Syto |
| Nuclei_Intensity_IntegratedIntensity_ER |
| Nuclei_Intensity_IntegratedIntensity_Hoechst |
| Nuclei_Intensity_IntegratedIntensity_Mito |
| Nuclei_Intensity_IntegratedIntensity_Ph_golgi |
| Nuclei_Intensity_IntegratedIntensity_Syto |
| Nuclei_Intensity_LowerQuartileIntensity_ER |
| Nuclei_Intensity_LowerQuartileIntensity_Hoechst |
| Nuclei_Intensity_LowerQuartileIntensity_Mito |
| Nuclei_Intensity_LowerQuartileIntensity_Ph_golgi |
| Nuclei_Intensity_LowerQuartileIntensity_Syto |
| Nuclei_Intensity_MassDisplacement_ER |
| Nuclei_Intensity_MassDisplacement_Hoechst |
| Nuclei_Intensity_MassDisplacement_Mito |
| Nuclei_Intensity_MassDisplacement_Ph_golgi |
| Nuclei_Intensity_MassDisplacement_Syto |
| Nuclei_Intensity_MaxIntensityEdge_ER |
| Nuclei_Intensity_MaxIntensityEdge_Hoechst |
| Nuclei_Intensity_MaxIntensityEdge_Mito |
| Nuclei_Intensity_MaxIntensityEdge_Ph_golgi |
| Nuclei_Intensity_MaxIntensityEdge_Syto |
| Nuclei_Intensity_MaxIntensity_ER |
| Nuclei_Intensity_MaxIntensity_Hoechst |
| Nuclei_Intensity_MaxIntensity_Mito |
| Nuclei_Intensity_MaxIntensity_Ph_golgi |
| Nuclei_Intensity_MaxIntensity_Syto |
| Nuclei_Intensity_MeanIntensityEdge_ER |
| Nuclei_Intensity_MeanIntensityEdge_Hoechst |
| Nuclei_Intensity_MeanIntensityEdge_Mito |
| Nuclei_Intensity_MeanIntensityEdge_Ph_golgi |
| Nuclei_Intensity_MeanIntensityEdge_Syto |
| Nuclei_Intensity_MeanIntensity_ER |
| Nuclei_Intensity_MeanIntensity_Hoechst |
| Nuclei_Intensity_MeanIntensity_Mito |
| Nuclei_Intensity_MeanIntensity_Ph_golgi |
| Nuclei_Intensity_MeanIntensity_Syto |
| Nuclei_Intensity_MedianIntensity_ER |
| Nuclei_Intensity_MedianIntensity_Hoechst |
| Nuclei_Intensity_MedianIntensity_Mito |
| Nuclei_Intensity_MedianIntensity_Ph_golgi |
| Nuclei_Intensity_MedianIntensity_Syto |
| Nuclei_Intensity_MinIntensityEdge_ER |
| Nuclei_Intensity_MinIntensityEdge_Hoechst |
| Nuclei_Intensity_MinIntensityEdge_Mito |
| Nuclei_Intensity_MinIntensityEdge_Ph_golgi |
| Nuclei_Intensity_MinIntensityEdge_Syto |
| Nuclei_Intensity_MinIntensity_ER |
| Nuclei_Intensity_MinIntensity_Hoechst |
| Nuclei_Intensity_MinIntensity_Mito |
| Nuclei_Intensity_MinIntensity_Ph_golgi |
| Nuclei_Intensity_MinIntensity_Syto |
| Nuclei_Intensity_StdIntensityEdge_ER |
| Nuclei_Intensity_StdIntensityEdge_Hoechst |
| Nuclei_Intensity_StdIntensityEdge_Mito |
| Nuclei_Intensity_StdIntensityEdge_Ph_golgi |
| Nuclei_Intensity_StdIntensityEdge_Syto |
| Nuclei_Intensity_StdIntensity_ER |
| Nuclei_Intensity_StdIntensity_Hoechst |
| Nuclei_Intensity_StdIntensity_Mito |
| Nuclei_Intensity_StdIntensity_Ph_golgi |
| Nuclei_Intensity_StdIntensity_Syto |
| Nuclei_Intensity_UpperQuartileIntensity_ER |
| Nuclei_Intensity_UpperQuartileIntensity_Hoechst |
| Nuclei_Intensity_UpperQuartileIntensity_Mito |
| Nuclei_Intensity_UpperQuartileIntensity_Ph_golgi |
| Nuclei_Intensity_UpperQuartileIntensity_Syto |
| Nuclei_Neighbors_NumberOfNeighbors_1 |
| Nuclei_Neighbors_PercentTouching_1 |
| Nuclei_Texture_AngularSecondMoment_ER_3 |
| Nuclei_Texture_AngularSecondMoment_ER_5 |
| Nuclei_Texture_AngularSecondMoment_Hoechst_3 |
| Nuclei_Texture_AngularSecondMoment_Hoechst_5 |
| Nuclei_Texture_AngularSecondMoment_Mito_3 |
| Nuclei_Texture_AngularSecondMoment_Mito_5 |
| Nuclei_Texture_AngularSecondMoment_Ph_golgi_3 |
| Nuclei_Texture_AngularSecondMoment_Ph_golgi_5 |
| Nuclei_Texture_AngularSecondMoment_Syto_3 |
| Nuclei_Texture_AngularSecondMoment_Syto_5 |
| Nuclei_Texture_Contrast_ER_3 |
| Nuclei_Texture_Contrast_ER_5 |
| Nuclei_Texture_Contrast_Hoechst_3 |
| Nuclei_Texture_Contrast_Hoechst_5 |
| Nuclei_Texture_Contrast_Mito_3 |
| Nuclei_Texture_Contrast_Mito_5 |
| Nuclei_Texture_Contrast_Ph_golgi_3 |
| Nuclei_Texture_Contrast_Ph_golgi_5 |
| Nuclei_Texture_Contrast_Syto_3 |
| Nuclei_Texture_Contrast_Syto_5 |
| Nuclei_Texture_Correlation_ER_3 |
| Nuclei_Texture_Correlation_ER_5 |
| Nuclei_Texture_Correlation_Hoechst_3 |
| Nuclei_Texture_Correlation_Hoechst_5 |
| Nuclei_Texture_Correlation_Mito_3 |
| Nuclei_Texture_Correlation_Mito_5 |
| Nuclei_Texture_Correlation_Ph_golgi_3 |
| Nuclei_Texture_Correlation_Ph_golgi_5 |
| Nuclei_Texture_Correlation_Syto_3 |
| Nuclei_Texture_Correlation_Syto_5 |
| Nuclei_Texture_DifferenceEntropy_ER_3 |
| Nuclei_Texture_DifferenceEntropy_ER_5 |
| Nuclei_Texture_DifferenceEntropy_Hoechst_3 |
| Nuclei_Texture_DifferenceEntropy_Hoechst_5 |
| Nuclei_Texture_DifferenceEntropy_Mito_3 |
| Nuclei_Texture_DifferenceEntropy_Mito_5 |
| Nuclei_Texture_DifferenceEntropy_Ph_golgi_3 |
| Nuclei_Texture_DifferenceEntropy_Ph_golgi_5 |
| Nuclei_Texture_DifferenceEntropy_Syto_3 |
| Nuclei_Texture_DifferenceEntropy_Syto_5 |
| Nuclei_Texture_DifferenceVariance_ER_3 |
| Nuclei_Texture_DifferenceVariance_ER_5 |
| Nuclei_Texture_DifferenceVariance_Hoechst_3 |
| Nuclei_Texture_DifferenceVariance_Hoechst_5 |
| Nuclei_Texture_DifferenceVariance_Mito_3 |
| Nuclei_Texture_DifferenceVariance_Mito_5 |
| Nuclei_Texture_DifferenceVariance_Ph_golgi_3 |
| Nuclei_Texture_DifferenceVariance_Ph_golgi_5 |
| Nuclei_Texture_DifferenceVariance_Syto_3 |
| Nuclei_Texture_DifferenceVariance_Syto_5 |
| Nuclei_Texture_Entropy_ER_3 |
| Nuclei_Texture_Entropy_ER_5 |
| Nuclei_Texture_Entropy_Hoechst_3 |
| Nuclei_Texture_Entropy_Hoechst_5 |
| Nuclei_Texture_Entropy_Mito_3 |
| Nuclei_Texture_Entropy_Mito_5 |
| Nuclei_Texture_Entropy_Ph_golgi_3 |
| Nuclei_Texture_Entropy_Ph_golgi_5 |
| Nuclei_Texture_Entropy_Syto_3 |
| Nuclei_Texture_Entropy_Syto_5 |
| Nuclei_Texture_Gabor_ER_3 |
| Nuclei_Texture_Gabor_ER_5 |
| Nuclei_Texture_Gabor_Hoechst_3 |
| Nuclei_Texture_Gabor_Hoechst_5 |
| Nuclei_Texture_Gabor_Mito_3 |
| Nuclei_Texture_Gabor_Mito_5 |
| Nuclei_Texture_Gabor_Ph_golgi_3 |
| Nuclei_Texture_Gabor_Ph_golgi_5 |
| Nuclei_Texture_Gabor_Syto_3 |
| Nuclei_Texture_Gabor_Syto_5 |
| Nuclei_Texture_InfoMeas1_ER_3 |
| Nuclei_Texture_InfoMeas1_ER_5 |
| Nuclei_Texture_InfoMeas1_Hoechst_3 |
| Nuclei_Texture_InfoMeas1_Hoechst_5 |
| Nuclei_Texture_InfoMeas1_Mito_3 |
| Nuclei_Texture_InfoMeas1_Mito_5 |
| Nuclei_Texture_InfoMeas1_Ph_golgi_3 |
| Nuclei_Texture_InfoMeas1_Ph_golgi_5 |
| Nuclei_Texture_InfoMeas1_Syto_3 |
| Nuclei_Texture_InfoMeas1_Syto_5 |
| Nuclei_Texture_InfoMeas2_ER_3 |
| Nuclei_Texture_InfoMeas2_ER_5 |
| Nuclei_Texture_InfoMeas2_Hoechst_3 |
| Nuclei_Texture_InfoMeas2_Hoechst_5 |
| Nuclei_Texture_InfoMeas2_Mito_3 |
| Nuclei_Texture_InfoMeas2_Mito_5 |
| Nuclei_Texture_InfoMeas2_Ph_golgi_3 |
| Nuclei_Texture_InfoMeas2_Ph_golgi_5 |
| Nuclei_Texture_InfoMeas2_Syto_3 |
| Nuclei_Texture_InfoMeas2_Syto_5 |
| Nuclei_Texture_InverseDifferenceMoment_ER_3 |
| Nuclei_Texture_InverseDifferenceMoment_ER_5 |
| Nuclei_Texture_InverseDifferenceMoment_Hoechst_3 |
| Nuclei_Texture_InverseDifferenceMoment_Hoechst_5 |
| Nuclei_Texture_InverseDifferenceMoment_Mito_3 |
| Nuclei_Texture_InverseDifferenceMoment_Mito_5 |
| Nuclei_Texture_InverseDifferenceMoment_Ph_golgi_3 |
| Nuclei_Texture_InverseDifferenceMoment_Ph_golgi_5 |
| Nuclei_Texture_InverseDifferenceMoment_Syto_3 |
| Nuclei_Texture_InverseDifferenceMoment_Syto_5 |
| Nuclei_Texture_SumAverage_ER_3 |
| Nuclei_Texture_SumAverage_ER_5 |
| Nuclei_Texture_SumAverage_Hoechst_3 |
| Nuclei_Texture_SumAverage_Hoechst_5 |
| Nuclei_Texture_SumAverage_Mito_3 |
| Nuclei_Texture_SumAverage_Mito_5 |
| Nuclei_Texture_SumAverage_Ph_golgi_3 |
| Nuclei_Texture_SumAverage_Ph_golgi_5 |
| Nuclei_Texture_SumAverage_Syto_3 |
| Nuclei_Texture_SumAverage_Syto_5 |
| Nuclei_Texture_SumEntropy_ER_3 |
| Nuclei_Texture_SumEntropy_ER_5 |
| Nuclei_Texture_SumEntropy_Hoechst_3 |
| Nuclei_Texture_SumEntropy_Hoechst_5 |
| Nuclei_Texture_SumEntropy_Mito_3 |
| Nuclei_Texture_SumEntropy_Mito_5 |
| Nuclei_Texture_SumEntropy_Ph_golgi_3 |
| Nuclei_Texture_SumEntropy_Ph_golgi_5 |
| Nuclei_Texture_SumEntropy_Syto_3 |
| Nuclei_Texture_SumEntropy_Syto_5 |
| Nuclei_Texture_SumVariance_ER_3 |
| Nuclei_Texture_SumVariance_ER_5 |
| Nuclei_Texture_SumVariance_Hoechst_3 |
| Nuclei_Texture_SumVariance_Hoechst_5 |
| Nuclei_Texture_SumVariance_Mito_3 |
| Nuclei_Texture_SumVariance_Mito_5 |
| Nuclei_Texture_SumVariance_Ph_golgi_3 |
| Nuclei_Texture_SumVariance_Ph_golgi_5 |
| Nuclei_Texture_SumVariance_Syto_3 |
| Nuclei_Texture_SumVariance_Syto_5 |
| Nuclei_Texture_Variance_ER_3 |
| Nuclei_Texture_Variance_ER_5 |
| Nuclei_Texture_Variance_Hoechst_3 |
| Nuclei_Texture_Variance_Hoechst_5 |
| Nuclei_Texture_Variance_Mito_3 |
| Nuclei_Texture_Variance_Mito_5 |
| Nuclei_Texture_Variance_Ph_golgi_3 |
| Nuclei_Texture_Variance_Ph_golgi_5 |
| Nuclei_Texture_Variance_Syto_3 |
| Nuclei_Texture_Variance_Syto_5 |
| Cells_AreaShape_Area |
| Cells_AreaShape_Eccentricity |
| Cells_AreaShape_EulerNumber |
| Cells_AreaShape_Extent |
| Cells_AreaShape_FormFactor |
| Cells_AreaShape_MajorAxisLength |
| Cells_AreaShape_MinorAxisLength |
| Cells_AreaShape_Orientation |
| Cells_AreaShape_Perimeter |
| Cells_AreaShape_Solidity |
| Cells_AreaShape_Zernike_0_0 |
| Cells_AreaShape_Zernike_1_1 |
| Cells_AreaShape_Zernike_2_0 |
| Cells_AreaShape_Zernike_2_2 |
| Cells_AreaShape_Zernike_3_1 |
| Cells_AreaShape_Zernike_3_3 |
| Cells_AreaShape_Zernike_4_0 |
| Cells_AreaShape_Zernike_4_2 |
| Cells_AreaShape_Zernike_4_4 |
| Cells_AreaShape_Zernike_5_1 |
| Cells_AreaShape_Zernike_5_3 |
| Cells_AreaShape_Zernike_5_5 |
| Cells_AreaShape_Zernike_6_0 |
| Cells_AreaShape_Zernike_6_2 |
| Cells_AreaShape_Zernike_6_4 |
| Cells_AreaShape_Zernike_6_6 |
| Cells_AreaShape_Zernike_7_1 |
| Cells_AreaShape_Zernike_7_3 |
| Cells_AreaShape_Zernike_7_5 |
| Cells_AreaShape_Zernike_7_7 |
| Cells_AreaShape_Zernike_8_0 |
| Cells_AreaShape_Zernike_8_2 |
| Cells_AreaShape_Zernike_8_4 |
| Cells_AreaShape_Zernike_8_6 |
| Cells_AreaShape_Zernike_8_8 |
| Cells_AreaShape_Zernike_9_1 |
| Cells_AreaShape_Zernike_9_3 |
| Cells_AreaShape_Zernike_9_5 |
| Cells_AreaShape_Zernike_9_7 |
| Cells_AreaShape_Zernike_9_9 |
| Cells_Children_Cytoplasm_Count |
| Cells_Intensity_IntegratedIntensityEdge_ER |
| Cells_Intensity_IntegratedIntensityEdge_Hoechst |
| Cells_Intensity_IntegratedIntensityEdge_Mito |
| Cells_Intensity_IntegratedIntensityEdge_Ph_golgi |
| Cells_Intensity_IntegratedIntensityEdge_Syto |
| Cells_Intensity_IntegratedIntensity_ER |
| Cells_Intensity_IntegratedIntensity_Hoechst |
| Cells_Intensity_IntegratedIntensity_Mito |
| Cells_Intensity_IntegratedIntensity_Ph_golgi |
| Cells_Intensity_IntegratedIntensity_Syto |
| Cells_Intensity_LowerQuartileIntensity_ER |
| Cells_Intensity_LowerQuartileIntensity_Hoechst |
| Cells_Intensity_LowerQuartileIntensity_Mito |
| Cells_Intensity_LowerQuartileIntensity_Ph_golgi |
| Cells_Intensity_LowerQuartileIntensity_Syto |
| Cells_Intensity_MassDisplacement_ER |
| Cells_Intensity_MassDisplacement_Hoechst |
| Cells_Intensity_MassDisplacement_Mito |
| Cells_Intensity_MassDisplacement_Ph_golgi |
| Cells_Intensity_MassDisplacement_Syto |
| Cells_Intensity_MaxIntensityEdge_ER |
| Cells_Intensity_MaxIntensityEdge_Hoechst |
| Cells_Intensity_MaxIntensityEdge_Mito |
| Cells_Intensity_MaxIntensityEdge_Ph_golgi |
| Cells_Intensity_MaxIntensityEdge_Syto |
| Cells_Intensity_MaxIntensity_ER |
| Cells_Intensity_MaxIntensity_Hoechst |
| Cells_Intensity_MaxIntensity_Mito |
| Cells_Intensity_MaxIntensity_Ph_golgi |
| Cells_Intensity_MaxIntensity_Syto |
| Cells_Intensity_MeanIntensityEdge_ER |
| Cells_Intensity_MeanIntensityEdge_Hoechst |
| Cells_Intensity_MeanIntensityEdge_Mito |
| Cells_Intensity_MeanIntensityEdge_Ph_golgi |
| Cells_Intensity_MeanIntensityEdge_Syto |
| Cells_Intensity_MeanIntensity_ER |
| Cells_Intensity_MeanIntensity_Hoechst |
| Cells_Intensity_MeanIntensity_Mito |
| Cells_Intensity_MeanIntensity_Ph_golgi |
| Cells_Intensity_MeanIntensity_Syto |
| Cells_Intensity_MedianIntensity_ER |
| Cells_Intensity_MedianIntensity_Hoechst |
| Cells_Intensity_MedianIntensity_Mito |
| Cells_Intensity_MedianIntensity_Ph_golgi |
| Cells_Intensity_MedianIntensity_Syto |
| Cells_Intensity_MinIntensityEdge_ER |
| Cells_Intensity_MinIntensityEdge_Hoechst |
| Cells_Intensity_MinIntensityEdge_Mito |
| Cells_Intensity_MinIntensityEdge_Ph_golgi |
| Cells_Intensity_MinIntensityEdge_Syto |
| Cells_Intensity_MinIntensity_ER |
| Cells_Intensity_MinIntensity_Hoechst |
| Cells_Intensity_MinIntensity_Mito |
| Cells_Intensity_MinIntensity_Ph_golgi |
| Cells_Intensity_MinIntensity_Syto |
| Cells_Intensity_StdIntensityEdge_ER |
| Cells_Intensity_StdIntensityEdge_Hoechst |
| Cells_Intensity_StdIntensityEdge_Mito |
| Cells_Intensity_StdIntensityEdge_Ph_golgi |
| Cells_Intensity_StdIntensityEdge_Syto |
| Cells_Intensity_StdIntensity_ER |
| Cells_Intensity_StdIntensity_Hoechst |
| Cells_Intensity_StdIntensity_Mito |
| Cells_Intensity_StdIntensity_Ph_golgi |
| Cells_Intensity_StdIntensity_Syto |
| Cells_Intensity_UpperQuartileIntensity_ER |
| Cells_Intensity_UpperQuartileIntensity_Hoechst |
| Cells_Intensity_UpperQuartileIntensity_Mito |
| Cells_Intensity_UpperQuartileIntensity_Ph_golgi |
| Cells_Intensity_UpperQuartileIntensity_Syto |
| Cells_Neighbors_NumberOfNeighbors_5 |
| Cells_Neighbors_PercentTouching_5 |
| Cells_RadialDistribution_FracAtD_ER_1of4 |
| Cells_RadialDistribution_FracAtD_ER_2of4 |
| Cells_RadialDistribution_FracAtD_ER_3of4 |
| Cells_RadialDistribution_FracAtD_ER_4of4 |
| Cells_RadialDistribution_FracAtD_Mito_1of4 |
| Cells_RadialDistribution_FracAtD_Mito_2of4 |
| Cells_RadialDistribution_FracAtD_Mito_3of4 |
| Cells_RadialDistribution_FracAtD_Mito_4of4 |
| Cells_RadialDistribution_FracAtD_Ph_golgi_1of4 |
| Cells_RadialDistribution_FracAtD_Ph_golgi_2of4 |
| Cells_RadialDistribution_FracAtD_Ph_golgi_3of4 |
| Cells_RadialDistribution_FracAtD_Ph_golgi_4of4 |
| Cells_RadialDistribution_FracAtD_Syto_1of4 |
| Cells_RadialDistribution_FracAtD_Syto_2of4 |
| Cells_RadialDistribution_FracAtD_Syto_3of4 |
| Cells_RadialDistribution_FracAtD_Syto_4of4 |
| Cells_RadialDistribution_MeanFrac_ER_1of4 |
| Cells_RadialDistribution_MeanFrac_ER_2of4 |
| Cells_RadialDistribution_MeanFrac_ER_3of4 |
| Cells_RadialDistribution_MeanFrac_ER_4of4 |
| Cells_RadialDistribution_MeanFrac_Mito_1of4 |
| Cells_RadialDistribution_MeanFrac_Mito_2of4 |
| Cells_RadialDistribution_MeanFrac_Mito_3of4 |
| Cells_RadialDistribution_MeanFrac_Mito_4of4 |
| Cells_RadialDistribution_MeanFrac_Ph_golgi_1of4 |
| Cells_RadialDistribution_MeanFrac_Ph_golgi_2of4 |
| Cells_RadialDistribution_MeanFrac_Ph_golgi_3of4 |
| Cells_RadialDistribution_MeanFrac_Ph_golgi_4of4 |
| Cells_RadialDistribution_MeanFrac_Syto_1of4 |
| Cells_RadialDistribution_MeanFrac_Syto_2of4 |
| Cells_RadialDistribution_MeanFrac_Syto_3of4 |
| Cells_RadialDistribution_MeanFrac_Syto_4of4 |
| Cells_RadialDistribution_RadialCV_ER_1of4 |
| Cells_RadialDistribution_RadialCV_ER_2of4 |
| Cells_RadialDistribution_RadialCV_ER_3of4 |
| Cells_RadialDistribution_RadialCV_ER_4of4 |
| Cells_RadialDistribution_RadialCV_Mito_1of4 |
| Cells_RadialDistribution_RadialCV_Mito_2of4 |
| Cells_RadialDistribution_RadialCV_Mito_3of4 |
| Cells_RadialDistribution_RadialCV_Mito_4of4 |
| Cells_RadialDistribution_RadialCV_Ph_golgi_1of4 |
| Cells_RadialDistribution_RadialCV_Ph_golgi_2of4 |
| Cells_RadialDistribution_RadialCV_Ph_golgi_3of4 |
| Cells_RadialDistribution_RadialCV_Ph_golgi_4of4 |
| Cells_RadialDistribution_RadialCV_Syto_1of4 |
| Cells_RadialDistribution_RadialCV_Syto_2of4 |
| Cells_RadialDistribution_RadialCV_Syto_3of4 |
| Cells_RadialDistribution_RadialCV_Syto_4of4 |
| Cells_Texture_AngularSecondMoment_ER_3 |
| Cells_Texture_AngularSecondMoment_ER_5 |
| Cells_Texture_AngularSecondMoment_Hoechst_3 |
| Cells_Texture_AngularSecondMoment_Hoechst_5 |
| Cells_Texture_AngularSecondMoment_Mito_3 |
| Cells_Texture_AngularSecondMoment_Mito_5 |
| Cells_Texture_AngularSecondMoment_Ph_golgi_3 |
| Cells_Texture_AngularSecondMoment_Ph_golgi_5 |
| Cells_Texture_AngularSecondMoment_Syto_3 |
| Cells_Texture_AngularSecondMoment_Syto_5 |
| Cells_Texture_Contrast_ER_3 |
| Cells_Texture_Contrast_ER_5 |
| Cells_Texture_Contrast_Hoechst_3 |
| Cells_Texture_Contrast_Hoechst_5 |
| Cells_Texture_Contrast_Mito_3 |
| Cells_Texture_Contrast_Mito_5 |
| Cells_Texture_Contrast_Ph_golgi_3 |
| Cells_Texture_Contrast_Ph_golgi_5 |
| Cells_Texture_Contrast_Syto_3 |
| Cells_Texture_Contrast_Syto_5 |
| Cells_Texture_Correlation_ER_3 |
| Cells_Texture_Correlation_ER_5 |
| Cells_Texture_Correlation_Hoechst_3 |
| Cells_Texture_Correlation_Hoechst_5 |
| Cells_Texture_Correlation_Mito_3 |
| Cells_Texture_Correlation_Mito_5 |
| Cells_Texture_Correlation_Ph_golgi_3 |
| Cells_Texture_Correlation_Ph_golgi_5 |
| Cells_Texture_Correlation_Syto_3 |
| Cells_Texture_Correlation_Syto_5 |
| Cells_Texture_DifferenceEntropy_ER_3 |
| Cells_Texture_DifferenceEntropy_ER_5 |
| Cells_Texture_DifferenceEntropy_Hoechst_3 |
| Cells_Texture_DifferenceEntropy_Hoechst_5 |
| Cells_Texture_DifferenceEntropy_Mito_3 |
| Cells_Texture_DifferenceEntropy_Mito_5 |
| Cells_Texture_DifferenceEntropy_Ph_golgi_3 |
| Cells_Texture_DifferenceEntropy_Ph_golgi_5 |
| Cells_Texture_DifferenceEntropy_Syto_3 |
| Cells_Texture_DifferenceEntropy_Syto_5 |
| Cells_Texture_DifferenceVariance_ER_3 |
| Cells_Texture_DifferenceVariance_ER_5 |
| Cells_Texture_DifferenceVariance_Hoechst_3 |
| Cells_Texture_DifferenceVariance_Hoechst_5 |
| Cells_Texture_DifferenceVariance_Mito_3 |
| Cells_Texture_DifferenceVariance_Mito_5 |
| Cells_Texture_DifferenceVariance_Ph_golgi_3 |
| Cells_Texture_DifferenceVariance_Ph_golgi_5 |
| Cells_Texture_DifferenceVariance_Syto_3 |
| Cells_Texture_DifferenceVariance_Syto_5 |
| Cells_Texture_Entropy_ER_3 |
| Cells_Texture_Entropy_ER_5 |
| Cells_Texture_Entropy_Hoechst_3 |
| Cells_Texture_Entropy_Hoechst_5 |
| Cells_Texture_Entropy_Mito_3 |
| Cells_Texture_Entropy_Mito_5 |
| Cells_Texture_Entropy_Ph_golgi_3 |
| Cells_Texture_Entropy_Ph_golgi_5 |
| Cells_Texture_Entropy_Syto_3 |
| Cells_Texture_Entropy_Syto_5 |
| Cells_Texture_Gabor_ER_3 |
| Cells_Texture_Gabor_ER_5 |
| Cells_Texture_Gabor_Hoechst_3 |
| Cells_Texture_Gabor_Hoechst_5 |
| Cells_Texture_Gabor_Mito_3 |
| Cells_Texture_Gabor_Mito_5 |
| Cells_Texture_Gabor_Ph_golgi_3 |
| Cells_Texture_Gabor_Ph_golgi_5 |
| Cells_Texture_Gabor_Syto_3 |
| Cells_Texture_Gabor_Syto_5 |
| Cells_Texture_InfoMeas1_ER_3 |
| Cells_Texture_InfoMeas1_ER_5 |
| Cells_Texture_InfoMeas1_Hoechst_3 |
| Cells_Texture_InfoMeas1_Hoechst_5 |
| Cells_Texture_InfoMeas1_Mito_3 |
| Cells_Texture_InfoMeas1_Mito_5 |
| Cells_Texture_InfoMeas1_Ph_golgi_3 |
| Cells_Texture_InfoMeas1_Ph_golgi_5 |
| Cells_Texture_InfoMeas1_Syto_3 |
| Cells_Texture_InfoMeas1_Syto_5 |
| Cells_Texture_InfoMeas2_ER_3 |
| Cells_Texture_InfoMeas2_ER_5 |
| Cells_Texture_InfoMeas2_Hoechst_3 |
| Cells_Texture_InfoMeas2_Hoechst_5 |
| Cells_Texture_InfoMeas2_Mito_3 |
| Cells_Texture_InfoMeas2_Mito_5 |
| Cells_Texture_InfoMeas2_Ph_golgi_3 |
| Cells_Texture_InfoMeas2_Ph_golgi_5 |
| Cells_Texture_InfoMeas2_Syto_3 |
| Cells_Texture_InfoMeas2_Syto_5 |
| Cells_Texture_InverseDifferenceMoment_ER_3 |
| Cells_Texture_InverseDifferenceMoment_ER_5 |
| Cells_Texture_InverseDifferenceMoment_Hoechst_3 |
| Cells_Texture_InverseDifferenceMoment_Hoechst_5 |
| Cells_Texture_InverseDifferenceMoment_Mito_3 |
| Cells_Texture_InverseDifferenceMoment_Mito_5 |
| Cells_Texture_InverseDifferenceMoment_Ph_golgi_3 |
| Cells_Texture_InverseDifferenceMoment_Ph_golgi_5 |
| Cells_Texture_InverseDifferenceMoment_Syto_3 |
| Cells_Texture_InverseDifferenceMoment_Syto_5 |
| Cells_Texture_SumAverage_ER_3 |
| Cells_Texture_SumAverage_ER_5 |
| Cells_Texture_SumAverage_Hoechst_3 |
| Cells_Texture_SumAverage_Hoechst_5 |
| Cells_Texture_SumAverage_Mito_3 |
| Cells_Texture_SumAverage_Mito_5 |
| Cells_Texture_SumAverage_Ph_golgi_3 |
| Cells_Texture_SumAverage_Ph_golgi_5 |
| Cells_Texture_SumAverage_Syto_3 |
| Cells_Texture_SumAverage_Syto_5 |
| Cells_Texture_SumEntropy_ER_3 |
| Cells_Texture_SumEntropy_ER_5 |
| Cells_Texture_SumEntropy_Hoechst_3 |
| Cells_Texture_SumEntropy_Hoechst_5 |
| Cells_Texture_SumEntropy_Mito_3 |
| Cells_Texture_SumEntropy_Mito_5 |
| Cells_Texture_SumEntropy_Ph_golgi_3 |
| Cells_Texture_SumEntropy_Ph_golgi_5 |
| Cells_Texture_SumEntropy_Syto_3 |
| Cells_Texture_SumEntropy_Syto_5 |
| Cells_Texture_SumVariance_ER_3 |
| Cells_Texture_SumVariance_ER_5 |
| Cells_Texture_SumVariance_Hoechst_3 |
| Cells_Texture_SumVariance_Hoechst_5 |
| Cells_Texture_SumVariance_Mito_3 |
| Cells_Texture_SumVariance_Mito_5 |
| Cells_Texture_SumVariance_Ph_golgi_3 |
| Cells_Texture_SumVariance_Ph_golgi_5 |
| Cells_Texture_SumVariance_Syto_3 |
| Cells_Texture_SumVariance_Syto_5 |
| Cells_Texture_Variance_ER_3 |
| Cells_Texture_Variance_ER_5 |
| Cells_Texture_Variance_Hoechst_3 |
| Cells_Texture_Variance_Hoechst_5 |
| Cells_Texture_Variance_Mito_3 |
| Cells_Texture_Variance_Mito_5 |
| Cells_Texture_Variance_Ph_golgi_3 |
| Cells_Texture_Variance_Ph_golgi_5 |
| Cells_Texture_Variance_Syto_3 |
| Cells_Texture_Variance_Syto_5 |
| Cytoplasm_AreaShape_Area |
| Cytoplasm_AreaShape_Eccentricity |
| Cytoplasm_AreaShape_EulerNumber |
| Cytoplasm_AreaShape_Extent |
| Cytoplasm_AreaShape_FormFactor |
| Cytoplasm_AreaShape_MajorAxisLength |
| Cytoplasm_AreaShape_MinorAxisLength |
| Cytoplasm_AreaShape_Orientation |
| Cytoplasm_AreaShape_Perimeter |
| Cytoplasm_AreaShape_Solidity |
| Cytoplasm_AreaShape_Zernike_0_0 |
| Cytoplasm_AreaShape_Zernike_1_1 |
| Cytoplasm_AreaShape_Zernike_2_0 |
| Cytoplasm_AreaShape_Zernike_2_2 |
| Cytoplasm_AreaShape_Zernike_3_1 |
| Cytoplasm_AreaShape_Zernike_3_3 |
| Cytoplasm_AreaShape_Zernike_4_0 |
| Cytoplasm_AreaShape_Zernike_4_2 |
| Cytoplasm_AreaShape_Zernike_4_4 |
| Cytoplasm_AreaShape_Zernike_5_1 |
| Cytoplasm_AreaShape_Zernike_5_3 |
| Cytoplasm_AreaShape_Zernike_5_5 |
| Cytoplasm_AreaShape_Zernike_6_0 |
| Cytoplasm_AreaShape_Zernike_6_2 |
| Cytoplasm_AreaShape_Zernike_6_4 |
| Cytoplasm_AreaShape_Zernike_6_6 |
| Cytoplasm_AreaShape_Zernike_7_1 |
| Cytoplasm_AreaShape_Zernike_7_3 |
| Cytoplasm_AreaShape_Zernike_7_5 |
| Cytoplasm_AreaShape_Zernike_7_7 |
| Cytoplasm_AreaShape_Zernike_8_0 |
| Cytoplasm_AreaShape_Zernike_8_2 |
| Cytoplasm_AreaShape_Zernike_8_4 |
| Cytoplasm_AreaShape_Zernike_8_6 |
| Cytoplasm_AreaShape_Zernike_8_8 |
| Cytoplasm_AreaShape_Zernike_9_1 |
| Cytoplasm_AreaShape_Zernike_9_3 |
| Cytoplasm_AreaShape_Zernike_9_5 |
| Cytoplasm_AreaShape_Zernike_9_7 |
| Cytoplasm_AreaShape_Zernike_9_9 |
| Cytoplasm_Intensity_IntegratedIntensityEdge_ER |
| Cytoplasm_Intensity_IntegratedIntensityEdge_Hoechst |
| Cytoplasm_Intensity_IntegratedIntensityEdge_Mito |
| Cytoplasm_Intensity_IntegratedIntensityEdge_Ph_golgi |
| Cytoplasm_Intensity_IntegratedIntensityEdge_Syto |
| Cytoplasm_Intensity_IntegratedIntensity_ER |
| Cytoplasm_Intensity_IntegratedIntensity_Hoechst |
| Cytoplasm_Intensity_IntegratedIntensity_Mito |
| Cytoplasm_Intensity_IntegratedIntensity_Ph_golgi |
| Cytoplasm_Intensity_IntegratedIntensity_Syto |
| Cytoplasm_Intensity_LowerQuartileIntensity_ER |
| Cytoplasm_Intensity_LowerQuartileIntensity_Hoechst |
| Cytoplasm_Intensity_LowerQuartileIntensity_Mito |
| Cytoplasm_Intensity_LowerQuartileIntensity_Ph_golgi |
| Cytoplasm_Intensity_LowerQuartileIntensity_Syto |
| Cytoplasm_Intensity_MassDisplacement_ER |
| Cytoplasm_Intensity_MassDisplacement_Hoechst |
| Cytoplasm_Intensity_MassDisplacement_Mito |
| Cytoplasm_Intensity_MassDisplacement_Ph_golgi |
| Cytoplasm_Intensity_MassDisplacement_Syto |
| Cytoplasm_Intensity_MaxIntensityEdge_ER |
| Cytoplasm_Intensity_MaxIntensityEdge_Hoechst |
| Cytoplasm_Intensity_MaxIntensityEdge_Mito |
| Cytoplasm_Intensity_MaxIntensityEdge_Ph_golgi |
| Cytoplasm_Intensity_MaxIntensityEdge_Syto |
| Cytoplasm_Intensity_MaxIntensity_ER |
| Cytoplasm_Intensity_MaxIntensity_Hoechst |
| Cytoplasm_Intensity_MaxIntensity_Mito |
| Cytoplasm_Intensity_MaxIntensity_Ph_golgi |
| Cytoplasm_Intensity_MaxIntensity_Syto |
| Cytoplasm_Intensity_MeanIntensityEdge_ER |
| Cytoplasm_Intensity_MeanIntensityEdge_Hoechst |
| Cytoplasm_Intensity_MeanIntensityEdge_Mito |
| Cytoplasm_Intensity_MeanIntensityEdge_Ph_golgi |
| Cytoplasm_Intensity_MeanIntensityEdge_Syto |
| Cytoplasm_Intensity_MeanIntensity_ER |
| Cytoplasm_Intensity_MeanIntensity_Hoechst |
| Cytoplasm_Intensity_MeanIntensity_Mito |
| Cytoplasm_Intensity_MeanIntensity_Ph_golgi |
| Cytoplasm_Intensity_MeanIntensity_Syto |
| Cytoplasm_Intensity_MedianIntensity_ER |
| Cytoplasm_Intensity_MedianIntensity_Hoechst |
| Cytoplasm_Intensity_MedianIntensity_Mito |
| Cytoplasm_Intensity_MedianIntensity_Ph_golgi |
| Cytoplasm_Intensity_MedianIntensity_Syto |
| Cytoplasm_Intensity_MinIntensityEdge_ER |
| Cytoplasm_Intensity_MinIntensityEdge_Hoechst |
| Cytoplasm_Intensity_MinIntensityEdge_Mito |
| Cytoplasm_Intensity_MinIntensityEdge_Ph_golgi |
| Cytoplasm_Intensity_MinIntensityEdge_Syto |
| Cytoplasm_Intensity_MinIntensity_ER |
| Cytoplasm_Intensity_MinIntensity_Hoechst |
| Cytoplasm_Intensity_MinIntensity_Mito |
| Cytoplasm_Intensity_MinIntensity_Ph_golgi |
| Cytoplasm_Intensity_MinIntensity_Syto |
| Cytoplasm_Intensity_StdIntensityEdge_ER |
| Cytoplasm_Intensity_StdIntensityEdge_Hoechst |
| Cytoplasm_Intensity_StdIntensityEdge_Mito |
| Cytoplasm_Intensity_StdIntensityEdge_Ph_golgi |
| Cytoplasm_Intensity_StdIntensityEdge_Syto |
| Cytoplasm_Intensity_StdIntensity_ER |
| Cytoplasm_Intensity_StdIntensity_Hoechst |
| Cytoplasm_Intensity_StdIntensity_Mito |
| Cytoplasm_Intensity_StdIntensity_Ph_golgi |
| Cytoplasm_Intensity_StdIntensity_Syto |
| Cytoplasm_Intensity_UpperQuartileIntensity_ER |
| Cytoplasm_Intensity_UpperQuartileIntensity_Hoechst |
| Cytoplasm_Intensity_UpperQuartileIntensity_Mito |
| Cytoplasm_Intensity_UpperQuartileIntensity_Ph_golgi |
| Cytoplasm_Intensity_UpperQuartileIntensity_Syto |
| Cytoplasm_Texture_AngularSecondMoment_ER_3 |
| Cytoplasm_Texture_AngularSecondMoment_ER_5 |
| Cytoplasm_Texture_AngularSecondMoment_Hoechst_3 |
| Cytoplasm_Texture_AngularSecondMoment_Hoechst_5 |
| Cytoplasm_Texture_AngularSecondMoment_Mito_3 |
| Cytoplasm_Texture_AngularSecondMoment_Mito_5 |
| Cytoplasm_Texture_AngularSecondMoment_Ph_golgi_3 |
| Cytoplasm_Texture_AngularSecondMoment_Ph_golgi_5 |
| Cytoplasm_Texture_AngularSecondMoment_Syto_3 |
| Cytoplasm_Texture_AngularSecondMoment_Syto_5 |
| Cytoplasm_Texture_Contrast_ER_3 |
| Cytoplasm_Texture_Contrast_ER_5 |
| Cytoplasm_Texture_Contrast_Hoechst_3 |
| Cytoplasm_Texture_Contrast_Hoechst_5 |
| Cytoplasm_Texture_Contrast_Mito_3 |
| Cytoplasm_Texture_Contrast_Mito_5 |
| Cytoplasm_Texture_Contrast_Ph_golgi_3 |
| Cytoplasm_Texture_Contrast_Ph_golgi_5 |
| Cytoplasm_Texture_Contrast_Syto_3 |
| Cytoplasm_Texture_Contrast_Syto_5 |
| Cytoplasm_Texture_Correlation_ER_3 |
| Cytoplasm_Texture_Correlation_ER_5 |
| Cytoplasm_Texture_Correlation_Hoechst_3 |
| Cytoplasm_Texture_Correlation_Hoechst_5 |
| Cytoplasm_Texture_Correlation_Mito_3 |
| Cytoplasm_Texture_Correlation_Mito_5 |
| Cytoplasm_Texture_Correlation_Ph_golgi_3 |
| Cytoplasm_Texture_Correlation_Ph_golgi_5 |
| Cytoplasm_Texture_Correlation_Syto_3 |
| Cytoplasm_Texture_Correlation_Syto_5 |
| Cytoplasm_Texture_DifferenceEntropy_ER_3 |
| Cytoplasm_Texture_DifferenceEntropy_ER_5 |
| Cytoplasm_Texture_DifferenceEntropy_Hoechst_3 |
| Cytoplasm_Texture_DifferenceEntropy_Hoechst_5 |
| Cytoplasm_Texture_DifferenceEntropy_Mito_3 |
| Cytoplasm_Texture_DifferenceEntropy_Mito_5 |
| Cytoplasm_Texture_DifferenceEntropy_Ph_golgi_3 |
| Cytoplasm_Texture_DifferenceEntropy_Ph_golgi_5 |
| Cytoplasm_Texture_DifferenceEntropy_Syto_3 |
| Cytoplasm_Texture_DifferenceEntropy_Syto_5 |
| Cytoplasm_Texture_DifferenceVariance_ER_3 |
| Cytoplasm_Texture_DifferenceVariance_ER_5 |
| Cytoplasm_Texture_DifferenceVariance_Hoechst_3 |
| Cytoplasm_Texture_DifferenceVariance_Hoechst_5 |
| Cytoplasm_Texture_DifferenceVariance_Mito_3 |
| Cytoplasm_Texture_DifferenceVariance_Mito_5 |
| Cytoplasm_Texture_DifferenceVariance_Ph_golgi_3 |
| Cytoplasm_Texture_DifferenceVariance_Ph_golgi_5 |
| Cytoplasm_Texture_DifferenceVariance_Syto_3 |
| Cytoplasm_Texture_DifferenceVariance_Syto_5 |
| Cytoplasm_Texture_Entropy_ER_3 |
| Cytoplasm_Texture_Entropy_ER_5 |
| Cytoplasm_Texture_Entropy_Hoechst_3 |
| Cytoplasm_Texture_Entropy_Hoechst_5 |
| Cytoplasm_Texture_Entropy_Mito_3 |
| Cytoplasm_Texture_Entropy_Mito_5 |
| Cytoplasm_Texture_Entropy_Ph_golgi_3 |
| Cytoplasm_Texture_Entropy_Ph_golgi_5 |
| Cytoplasm_Texture_Entropy_Syto_3 |
| Cytoplasm_Texture_Entropy_Syto_5 |
| Cytoplasm_Texture_Gabor_ER_3 |
| Cytoplasm_Texture_Gabor_ER_5 |
| Cytoplasm_Texture_Gabor_Hoechst_3 |
| Cytoplasm_Texture_Gabor_Hoechst_5 |
| Cytoplasm_Texture_Gabor_Mito_3 |
| Cytoplasm_Texture_Gabor_Mito_5 |
| Cytoplasm_Texture_Gabor_Ph_golgi_3 |
| Cytoplasm_Texture_Gabor_Ph_golgi_5 |
| Cytoplasm_Texture_Gabor_Syto_3 |
| Cytoplasm_Texture_Gabor_Syto_5 |
| Cytoplasm_Texture_InfoMeas1_ER_3 |
| Cytoplasm_Texture_InfoMeas1_ER_5 |
| Cytoplasm_Texture_InfoMeas1_Hoechst_3 |
| Cytoplasm_Texture_InfoMeas1_Hoechst_5 |
| Cytoplasm_Texture_InfoMeas1_Mito_3 |
| Cytoplasm_Texture_InfoMeas1_Mito_5 |
| Cytoplasm_Texture_InfoMeas1_Ph_golgi_3 |
| Cytoplasm_Texture_InfoMeas1_Ph_golgi_5 |
| Cytoplasm_Texture_InfoMeas1_Syto_3 |
| Cytoplasm_Texture_InfoMeas1_Syto_5 |
| Cytoplasm_Texture_InfoMeas2_ER_3 |
| Cytoplasm_Texture_InfoMeas2_ER_5 |
| Cytoplasm_Texture_InfoMeas2_Hoechst_3 |
| Cytoplasm_Texture_InfoMeas2_Hoechst_5 |
| Cytoplasm_Texture_InfoMeas2_Mito_3 |
| Cytoplasm_Texture_InfoMeas2_Mito_5 |
| Cytoplasm_Texture_InfoMeas2_Ph_golgi_3 |
| Cytoplasm_Texture_InfoMeas2_Ph_golgi_5 |
| Cytoplasm_Texture_InfoMeas2_Syto_3 |
| Cytoplasm_Texture_InfoMeas2_Syto_5 |
| Cytoplasm_Texture_InverseDifferenceMoment_ER_3 |
| Cytoplasm_Texture_InverseDifferenceMoment_ER_5 |
| Cytoplasm_Texture_InverseDifferenceMoment_Hoechst_3 |
| Cytoplasm_Texture_InverseDifferenceMoment_Hoechst_5 |
| Cytoplasm_Texture_InverseDifferenceMoment_Mito_3 |
| Cytoplasm_Texture_InverseDifferenceMoment_Mito_5 |
| Cytoplasm_Texture_InverseDifferenceMoment_Ph_golgi_3 |
| Cytoplasm_Texture_InverseDifferenceMoment_Ph_golgi_5 |
| Cytoplasm_Texture_InverseDifferenceMoment_Syto_3 |
| Cytoplasm_Texture_InverseDifferenceMoment_Syto_5 |
| Cytoplasm_Texture_SumAverage_ER_3 |
| Cytoplasm_Texture_SumAverage_ER_5 |
| Cytoplasm_Texture_SumAverage_Hoechst_3 |
| Cytoplasm_Texture_SumAverage_Hoechst_5 |
| Cytoplasm_Texture_SumAverage_Mito_3 |
| Cytoplasm_Texture_SumAverage_Mito_5 |
| Cytoplasm_Texture_SumAverage_Ph_golgi_3 |
| Cytoplasm_Texture_SumAverage_Ph_golgi_5 |
| Cytoplasm_Texture_SumAverage_Syto_3 |
| Cytoplasm_Texture_SumAverage_Syto_5 |
| Cytoplasm_Texture_SumEntropy_ER_3 |
| Cytoplasm_Texture_SumEntropy_ER_5 |
| Cytoplasm_Texture_SumEntropy_Hoechst_3 |
| Cytoplasm_Texture_SumEntropy_Hoechst_5 |
| Cytoplasm_Texture_SumEntropy_Mito_3 |
| Cytoplasm_Texture_SumEntropy_Mito_5 |
| Cytoplasm_Texture_SumEntropy_Ph_golgi_3 |
| Cytoplasm_Texture_SumEntropy_Ph_golgi_5 |
| Cytoplasm_Texture_SumEntropy_Syto_3 |
| Cytoplasm_Texture_SumEntropy_Syto_5 |
| Cytoplasm_Texture_SumVariance_ER_3 |
| Cytoplasm_Texture_SumVariance_ER_5 |
| Cytoplasm_Texture_SumVariance_Hoechst_3 |
| Cytoplasm_Texture_SumVariance_Hoechst_5 |
| Cytoplasm_Texture_SumVariance_Mito_3 |
| Cytoplasm_Texture_SumVariance_Mito_5 |
| Cytoplasm_Texture_SumVariance_Ph_golgi_3 |
| Cytoplasm_Texture_SumVariance_Ph_golgi_5 |
| Cytoplasm_Texture_SumVariance_Syto_3 |
| Cytoplasm_Texture_SumVariance_Syto_5 |
| Cytoplasm_Texture_Variance_ER_3 |
| Cytoplasm_Texture_Variance_ER_5 |
| Cytoplasm_Texture_Variance_Hoechst_3 |
| Cytoplasm_Texture_Variance_Hoechst_5 |
| Cytoplasm_Texture_Variance_Mito_3 |
| Cytoplasm_Texture_Variance_Mito_5 |
| Cytoplasm_Texture_Variance_Ph_golgi_3 |
| Cytoplasm_Texture_Variance_Ph_golgi_5 |
| Cytoplasm_Texture_Variance_Syto_3 |
| Cytoplasm_Texture_Variance_Syto_5 |
