# Supplementary material for: Multiplex Cytological Profiling Assay to Measure Diverse Cellular States
Source: PLoS One. 2013 Dec 2;8(12):e80999. doi: 10.1371/journal.pone.0080999 (PMC3847047; doi:10.1371/journal.pone.0080999)
Supplement: Table S3 — Features ranked by plate-to-plate coefficient of variation (absolute), limited to mock-treated cells. (DOCX) [file pone.0080999.s011.docx]

**Table S3:** Features ranked by plate-to-plate coefficient of variation (absolute), limited to mock-treated cells.

| **Rank** | **CV** | **Feature name** |
| --- | --- | --- |
| 1 | 1.044415 | Cells_AreaShape_Orientation |
| 2 | 1.042658 | Nuclei_AreaShape_Orientation |
| 3 | 1.042080 | Cytoplasm_AreaShape_Orientation |
| 4 | 0.242673 | Nuclei_Intensity_StdIntensityEdge_Mito |
| 5 | 0.240786 | Cytoplasm_Intensity_StdIntensityEdge_Mito |
| 6 | 0.234766 | Cells_Intensity_StdIntensity_Mito |
| 7 | 0.232615 | Cytoplasm_Intensity_StdIntensity_Mito |
| 8 | 0.231964 | Nuclei_Intensity_StdIntensity_Mito |
| 9 | 0.222669 | Cells_Intensity_StdIntensityEdge_Mito |
| 10 | 0.202502 | Nuclei_Intensity_StdIntensityEdge_ER |
| 11 | 0.202254 | Cytoplasm_Intensity_StdIntensityEdge_ER |
| 12 | 0.200603 | Nuclei_Intensity_StdIntensity_ER |
| 13 | 0.200030 | Nuclei_Intensity_MaxIntensityEdge_Mito |
| 14 | 0.199956 | Nuclei_Intensity_MaxIntensity_Mito |
| 15 | 0.199094 | Cells_Intensity_MaxIntensity_Mito |
| 16 | 0.198893 | Cytoplasm_Intensity_MaxIntensity_Mito |
| 17 | 0.198218 | Cytoplasm_Intensity_MaxIntensityEdge_Mito |
| 18 | 0.195590 | Cytoplasm_Intensity_IntegratedIntensity_Mito |
| 19 | 0.194209 | Cells_Intensity_StdIntensity_ER |
| 20 | 0.193199 | Cells_Intensity_StdIntensity_Ph_golgi |
| 21 | 0.191458 | Cytoplasm_Intensity_StdIntensity_ER |
| 22 | 0.190720 | Cells_Intensity_IntegratedIntensity_Mito |
| 23 | 0.188702 | Nuclei_Intensity_StdIntensityEdge_Ph_golgi |
| 24 | 0.183180 | Nuclei_Intensity_StdIntensity_Ph_golgi |
| 25 | 0.182565 | Nuclei_Intensity_IntegratedIntensity_Mito |
| 26 | 0.182506 | Cells_Intensity_MaxIntensityEdge_Mito |
| 27 | 0.180661 | Cytoplasm_Intensity_StdIntensityEdge_Ph_golgi |
| 28 | 0.180009 | Nuclei_Intensity_IntegratedIntensityEdge_Mito |
| 29 | 0.179958 | Cells_Intensity_StdIntensityEdge_ER |
| 30 | 0.178743 | Nuclei_Intensity_StdIntensity_Hoechst |
| 31 | 0.178196 | Cells_Intensity_MaxIntensity_ER |
| 32 | 0.178177 | Nuclei_Intensity_MeanIntensityEdge_Mito |
| 33 | 0.177745 | Cytoplasm_Intensity_MaxIntensity_ER |
| 34 | 0.177420 | Nuclei_Intensity_StdIntensity_Syto |
| 35 | 0.177267 | Nuclei_Intensity_MaxIntensity_ER |
| 36 | 0.176691 | Nuclei_Intensity_MaxIntensityEdge_ER |
| 37 | 0.176490 | Nuclei_Intensity_UpperQuartileIntensity_Mito |
| 38 | 0.174842 | Nuclei_Intensity_MeanIntensity_Mito |
| 39 | 0.173420 | Nuclei_Intensity_MedianIntensity_Mito |
| 40 | 0.173410 | Cytoplasm_Intensity_MaxIntensityEdge_ER |
| 41 | 0.170652 | Cells_Intensity_UpperQuartileIntensity_Mito |
| 42 | 0.170359 | Nuclei_Intensity_LowerQuartileIntensity_Mito |
| 43 | 0.166434 | Cytoplasm_Intensity_UpperQuartileIntensity_Mito |
| 44 | 0.166183 | Cells_Texture_InfoMeas1_Ph_golgi_5 |
| 45 | 0.165116 | Cytoplasm_Intensity_StdIntensity_Ph_golgi |
| 46 | 0.164450 | Cytoplasm_Intensity_IntegratedIntensity_ER |
| 47 | 0.163986 | Cells_Intensity_MeanIntensity_Mito |
| 48 | 0.162923 | Nuclei_Intensity_StdIntensityEdge_Hoechst |
| 49 | 0.162600 | Cytoplasm_Intensity_MeanIntensityEdge_Mito |
| 50 | 0.162085 | Cells_Intensity_StdIntensity_Hoechst |
| 51 | 0.161486 | Cytoplasm_Intensity_MeanIntensity_Mito |
| 52 | 0.160649 | Nuclei_Intensity_MinIntensity_Mito |
| 53 | 0.160452 | Nuclei_Intensity_MinIntensityEdge_Mito |
| 54 | 0.160135 | Cells_Intensity_IntegratedIntensity_ER |
| 55 | 0.158826 | Cells_Intensity_MedianIntensity_Mito |
| 56 | 0.157319 | Cytoplasm_Intensity_IntegratedIntensityEdge_Mito |
| 57 | 0.157045 | Cells_Intensity_StdIntensityEdge_Ph_golgi |
| 58 | 0.156301 | Nuclei_Intensity_IntegratedIntensityEdge_ER |
| 59 | 0.154503 | Cells_Intensity_MeanIntensityEdge_Mito |
| 60 | 0.154261 | Cells_Intensity_StdIntensity_Syto |
| 61 | 0.153934 | Cytoplasm_Intensity_MedianIntensity_Mito |
| 62 | 0.153757 | Nuclei_Intensity_MeanIntensityEdge_ER |
| 63 | 0.153670 | Nuclei_Intensity_IntegratedIntensity_ER |
| 64 | 0.150514 | Cells_Intensity_StdIntensityEdge_Hoechst |
| 65 | 0.149925 | Cells_Intensity_LowerQuartileIntensity_Mito |
| 66 | 0.148968 | Nuclei_Intensity_MaxIntensity_Ph_golgi |
| 67 | 0.148925 | Cells_Intensity_MaxIntensityEdge_ER |
| 68 | 0.147697 | Cytoplasm_Intensity_LowerQuartileIntensity_Mito |
| 69 | 0.146818 | Nuclei_Intensity_UpperQuartileIntensity_ER |
| 70 | 0.146764 | Cytoplasm_Intensity_IntegratedIntensity_Ph_golgi |
| 71 | 0.146642 | Nuclei_Intensity_MaxIntensityEdge_Ph_golgi |
| 72 | 0.145806 | Nuclei_Intensity_IntegratedIntensity_Ph_golgi |
| 73 | 0.145771 | Cells_Intensity_IntegratedIntensityEdge_Mito |
| 74 | 0.145530 | Nuclei_Intensity_MeanIntensity_ER |
| 75 | 0.145490 | Cells_Intensity_MaxIntensity_Ph_golgi |
| 76 | 0.144692 | Nuclei_Intensity_MaxIntensity_Hoechst |
| 77 | 0.144557 | Cells_Intensity_MaxIntensity_Hoechst |
| 78 | 0.144139 | Nuclei_Intensity_IntegratedIntensity_Hoechst |
| 79 | 0.143582 | Cells_Intensity_IntegratedIntensity_Ph_golgi |
| 80 | 0.143346 | Cytoplasm_Intensity_MaxIntensity_Ph_golgi |
| 81 | 0.142644 | Cytoplasm_Intensity_StdIntensityEdge_Hoechst |
| 82 | 0.142313 | Nuclei_Intensity_UpperQuartileIntensity_Ph_golgi |
| 83 | 0.142089 | Nuclei_Intensity_UpperQuartileIntensity_Hoechst |
| 84 | 0.142003 | Nuclei_Intensity_MedianIntensity_ER |
| 85 | 0.141268 | Cytoplasm_Intensity_MaxIntensityEdge_Ph_golgi |
| 86 | 0.140853 | Nuclei_Intensity_MedianIntensity_Ph_golgi |
| 87 | 0.140104 | Cells_Intensity_UpperQuartileIntensity_ER |
| 88 | 0.139726 | Nuclei_Intensity_MeanIntensity_Ph_golgi |
| 89 | 0.139559 | Cytoplasm_AreaShape_EulerNumber |
| 90 | 0.139203 | Nuclei_Intensity_LowerQuartileIntensity_ER |
| 91 | 0.139019 | Cells_Intensity_MinIntensityEdge_Mito |
| 92 | 0.139014 | Cytoplasm_Intensity_MinIntensityEdge_Mito |
| 93 | 0.138236 | Cells_Intensity_MinIntensity_Mito |
| 94 | 0.138234 | Cytoplasm_Intensity_MinIntensity_Mito |
| 95 | 0.138180 | Nuclei_Intensity_LowerQuartileIntensity_Ph_golgi |
| 96 | 0.137883 | Nuclei_Intensity_MedianIntensity_Hoechst |
| 97 | 0.137344 | Cytoplasm_Intensity_StdIntensityEdge_Syto |
| 98 | 0.137066 | Cytoplasm_Intensity_UpperQuartileIntensity_ER |
| 99 | 0.135133 | Nuclei_Intensity_MeanIntensity_Hoechst |
| 100 | 0.134048 | Cytoplasm_Intensity_MeanIntensityEdge_ER |
| 101 | 0.133600 | Cells_Intensity_MeanIntensity_ER |
| 102 | 0.132474 | Nuclei_Intensity_StdIntensityEdge_Syto |
| 103 | 0.132387 | Nuclei_Intensity_IntegratedIntensityEdge_Ph_golgi |
| 104 | 0.131525 | Cytoplasm_Intensity_StdIntensity_Hoechst |
| 105 | 0.131371 | Cytoplasm_Intensity_MeanIntensity_ER |
| 106 | 0.130858 | Nuclei_Intensity_MeanIntensityEdge_Ph_golgi |
| 107 | 0.129638 | Cells_Texture_InfoMeas1_Ph_golgi_3 |
| 108 | 0.129282 | Cytoplasm_Intensity_IntegratedIntensityEdge_ER |
| 109 | 0.128600 | Cells_Intensity_UpperQuartileIntensity_Ph_golgi |
| 110 | 0.128594 | Nuclei_Intensity_LowerQuartileIntensity_Hoechst |
| 111 | 0.127620 | Cells_Intensity_MedianIntensity_ER |
| 112 | 0.127534 | Cells_Intensity_MaxIntensityEdge_Ph_golgi |
| 113 | 0.127302 | Nuclei_Intensity_MinIntensityEdge_ER |
| 114 | 0.126161 | Nuclei_Intensity_MinIntensity_ER |
| 115 | 0.125981 | Cytoplasm_Intensity_StdIntensity_Syto |
| 116 | 0.124783 | Nuclei_Intensity_MaxIntensity_Syto |
| 117 | 0.124289 | Cells_Intensity_IntegratedIntensity_Hoechst |
| 118 | 0.123281 | Cytoplasm_Intensity_MedianIntensity_ER |
| 119 | 0.122748 | Cells_Intensity_MeanIntensity_Ph_golgi |
| 120 | 0.122459 | Cells_Intensity_MaxIntensity_Syto |
| 121 | 0.122339 | Cells_Intensity_MeanIntensityEdge_ER |
| 122 | 0.120828 | Cells_Intensity_MedianIntensity_Ph_golgi |
| 123 | 0.120052 | Cytoplasm_Intensity_UpperQuartileIntensity_Ph_golgi |
| 124 | 0.119841 | Cytoplasm_Intensity_MeanIntensityEdge_Ph_golgi |
| 125 | 0.119202 | Cytoplasm_Intensity_MaxIntensityEdge_Hoechst |
| 126 | 0.119059 | Cytoplasm_Intensity_MaxIntensity_Hoechst |
| 127 | 0.118633 | Nuclei_Intensity_MinIntensity_Ph_golgi |
| 128 | 0.118620 | Nuclei_Intensity_MaxIntensityEdge_Hoechst |
| 129 | 0.118208 | Cells_RadialDistribution_RadialCV_Ph_golgi_3of4 |
| 130 | 0.117790 | Cytoplasm_Intensity_MeanIntensity_Ph_golgi |
| 131 | 0.117720 | Nuclei_Intensity_MinIntensityEdge_Ph_golgi |
| 132 | 0.117176 | Cells_Intensity_LowerQuartileIntensity_ER |
| 133 | 0.117153 | Cells_Intensity_StdIntensityEdge_Syto |
| 134 | 0.116493 | Cells_Intensity_UpperQuartileIntensity_Hoechst |
| 135 | 0.116464 | Cytoplasm_Intensity_MedianIntensity_Ph_golgi |
| 136 | 0.115510 | Nuclei_Texture_Gabor_Mito_3 |
| 137 | 0.115496 | Cells_Intensity_MeanIntensityEdge_Ph_golgi |
| 138 | 0.115440 | Cells_Intensity_LowerQuartileIntensity_Ph_golgi |
| 139 | 0.114407 | Cytoplasm_Intensity_IntegratedIntensityEdge_Ph_golgi |
| 140 | 0.114271 | Cells_Intensity_IntegratedIntensityEdge_ER |
| 141 | 0.114191 | Cells_RadialDistribution_RadialCV_Ph_golgi_2of4 |
| 142 | 0.114167 | Cytoplasm_Intensity_LowerQuartileIntensity_ER |
| 143 | 0.113405 | Cytoplasm_Intensity_LowerQuartileIntensity_Ph_golgi |
| 144 | 0.111706 | Cytoplasm_Texture_AngularSecondMoment_Ph_golgi_5 |
| 145 | 0.110185 | Cytoplasm_Texture_AngularSecondMoment_Ph_golgi_3 |
| 146 | 0.106894 | Cells_Intensity_IntegratedIntensityEdge_Ph_golgi |
| 147 | 0.105657 | Cytoplasm_Intensity_IntegratedIntensity_Hoechst |
| 148 | 0.104082 | Nuclei_Intensity_IntegratedIntensityEdge_Hoechst |
| 149 | 0.102676 | Cells_Intensity_MinIntensityEdge_Ph_golgi |
| 150 | 0.102505 | Cytoplasm_Intensity_MinIntensityEdge_Ph_golgi |
| 151 | 0.102277 | Nuclei_Neighbors_NumberOfNeighbors_1 |
| 152 | 0.101638 | Cells_Intensity_MeanIntensity_Hoechst |
| 153 | 0.101478 | Nuclei_Intensity_MeanIntensityEdge_Hoechst |
| 154 | 0.101385 | Cells_Intensity_MinIntensity_Ph_golgi |
| 155 | 0.101372 | Cytoplasm_Intensity_MinIntensity_Ph_golgi |
| 156 | 0.101246 | Cells_RadialDistribution_RadialCV_Syto_1of4 |
| 157 | 0.101211 | Nuclei_Intensity_IntegratedIntensity_Syto |
| 158 | 0.101057 | Cells_Intensity_MaxIntensityEdge_Hoechst |
| 159 | 0.100915 | Cells_Intensity_MinIntensityEdge_ER |
| 160 | 0.100902 | Cytoplasm_Intensity_MinIntensityEdge_ER |
| 161 | 0.100356 | Cytoplasm_Intensity_IntegratedIntensity_Syto |
| 162 | 0.099548 | Cytoplasm_Intensity_MinIntensity_ER |
| 163 | 0.099544 | Cells_Intensity_MinIntensity_ER |
| 164 | 0.099153 | Nuclei_Intensity_UpperQuartileIntensity_Syto |
| 165 | 0.097249 | Cells_Intensity_IntegratedIntensity_Syto |
| 166 | 0.097021 | Nuclei_Neighbors_PercentTouching_1 |
| 167 | 0.096774 | Cells_Texture_Correlation_Ph_golgi_5 |
| 168 | 0.095554 | Cells_Texture_SumVariance_Ph_golgi_5 |
| 169 | 0.095349 | Nuclei_Intensity_MeanIntensity_Syto |
| 170 | 0.094939 | Nuclei_Texture_AngularSecondMoment_Mito_5 |
| 171 | 0.094829 | Nuclei_Texture_AngularSecondMoment_Mito_3 |
| 172 | 0.094368 | Cells_RadialDistribution_RadialCV_Syto_2of4 |
| 173 | 0.094267 | Nuclei_Intensity_MaxIntensityEdge_Syto |
| 174 | 0.094246 | Cytoplasm_Intensity_MaxIntensity_Syto |
| 175 | 0.094145 | Cytoplasm_Intensity_MaxIntensityEdge_Syto |
| 176 | 0.093776 | Nuclei_Intensity_MedianIntensity_Syto |
| 177 | 0.092429 | Cells_Intensity_MassDisplacement_Ph_golgi |
| 178 | 0.092010 | Cells_RadialDistribution_RadialCV_Mito_2of4 |
| 179 | 0.091430 | Cells_Texture_Gabor_Mito_3 |
| 180 | 0.090223 | Cells_RadialDistribution_RadialCV_Mito_3of4 |
| 181 | 0.089405 | Cells_Intensity_MassDisplacement_Mito |
| 182 | 0.087764 | Nuclei_Intensity_LowerQuartileIntensity_Syto |
| 183 | 0.087532 | Cytoplasm_AreaShape_FormFactor |
| 184 | 0.086270 | Nuclei_Intensity_MinIntensity_Hoechst |
| 185 | 0.086092 | Nuclei_Intensity_MinIntensityEdge_Hoechst |
| 186 | 0.084853 | Cells_RadialDistribution_RadialCV_Mito_1of4 |
| 187 | 0.084422 | Cytoplasm_Intensity_MassDisplacement_Mito |
| 188 | 0.083979 | Cells_Texture_SumVariance_Ph_golgi_3 |
| 189 | 0.082642 | Nuclei_Texture_InfoMeas1_Ph_golgi_3 |
| 190 | 0.082164 | Cytoplasm_Texture_AngularSecondMoment_Mito_5 |
| 191 | 0.082105 | Nuclei_Texture_InfoMeas1_Ph_golgi_5 |
| 192 | 0.081999 | Nuclei_Intensity_IntegratedIntensityEdge_Syto |
| 193 | 0.081848 | Cytoplasm_Texture_AngularSecondMoment_Mito_3 |
| 194 | 0.080786 | Cells_RadialDistribution_RadialCV_Syto_3of4 |
| 195 | 0.080763 | Nuclei_Intensity_MeanIntensityEdge_Syto |
| 196 | 0.080139 | Cells_Intensity_UpperQuartileIntensity_Syto |
| 197 | 0.078896 | Cytoplasm_Intensity_MeanIntensityEdge_Hoechst |
| 198 | 0.078372 | Nuclei_Texture_DifferenceVariance_Syto_5 |
| 199 | 0.077449 | Nuclei_Texture_DifferenceVariance_Syto_3 |
| 200 | 0.077266 | Cells_Texture_AngularSecondMoment_Syto_3 |
| 201 | 0.077196 | Nuclei_Intensity_MassDisplacement_Ph_golgi |
| 202 | 0.077076 | Cells_RadialDistribution_RadialCV_Mito_4of4 |
| 203 | 0.076893 | Cells_Texture_AngularSecondMoment_Syto_5 |
| 204 | 0.076882 | Cytoplasm_Texture_Variance_Hoechst_3 |
| 205 | 0.076390 | Nuclei_Intensity_MassDisplacement_Mito |
| 206 | 0.075356 | Cytoplasm_Texture_SumVariance_Hoechst_3 |
| 207 | 0.075355 | Cells_Intensity_MaxIntensityEdge_Syto |
| 208 | 0.075315 | Cytoplasm_Texture_Variance_Hoechst_5 |
| 209 | 0.075007 | Cells_Intensity_MassDisplacement_ER |
| 210 | 0.074900 | Cells_RadialDistribution_RadialCV_ER_2of4 |
| 211 | 0.074858 | Cytoplasm_Texture_SumVariance_Hoechst_5 |
| 212 | 0.074631 | Cells_Texture_InfoMeas2_Ph_golgi_5 |
| 213 | 0.074420 | Cytoplasm_Texture_Contrast_Hoechst_3 |
| 214 | 0.073744 | Cells_RadialDistribution_RadialCV_ER_3of4 |
| 215 | 0.073594 | Cells_AreaShape_FormFactor |
| 216 | 0.073321 | Cytoplasm_Texture_InfoMeas1_Ph_golgi_5 |
| 217 | 0.073297 | Cytoplasm_Texture_Contrast_Hoechst_5 |
| 218 | 0.073102 | Cytoplasm_Intensity_MassDisplacement_Hoechst |
| 219 | 0.072999 | Cells_Texture_Gabor_Syto_5 |
| 220 | 0.072781 | Cytoplasm_Intensity_IntegratedIntensityEdge_Hoechst |
| 221 | 0.072539 | Cytoplasm_Texture_AngularSecondMoment_ER_5 |
| 222 | 0.071903 | Cytoplasm_AreaShape_Area |
| 223 | 0.071728 | Cells_Intensity_MeanIntensity_Syto |
| 224 | 0.071585 | Cells_Intensity_MassDisplacement_Syto |
| 225 | 0.071537 | Cells_Texture_AngularSecondMoment_Hoechst_5 |
| 226 | 0.071344 | Cytoplasm_Intensity_MassDisplacement_ER |
| 227 | 0.070985 | Cells_RadialDistribution_RadialCV_Syto_4of4 |
| 228 | 0.070573 | Cytoplasm_Intensity_MassDisplacement_Syto |
| 229 | 0.070499 | Cytoplasm_Texture_AngularSecondMoment_ER_3 |
| 230 | 0.068881 | Cells_RadialDistribution_RadialCV_ER_1of4 |
| 231 | 0.068740 | Cells_Texture_Gabor_Syto_3 |
| 232 | 0.068580 | Nuclei_Texture_AngularSecondMoment_ER_5 |
| 233 | 0.067103 | Cytoplasm_Intensity_MassDisplacement_Ph_golgi |
| 234 | 0.066691 | Nuclei_Intensity_MassDisplacement_ER |
| 235 | 0.066442 | Nuclei_Texture_AngularSecondMoment_ER_3 |
| 236 | 0.066384 | Cells_Texture_AngularSecondMoment_Mito_3 |
| 237 | 0.066283 | Cells_Texture_AngularSecondMoment_Ph_golgi_5 |
| 238 | 0.066084 | Cells_RadialDistribution_RadialCV_ER_4of4 |
| 239 | 0.065895 | Cells_Texture_AngularSecondMoment_Ph_golgi_3 |
| 240 | 0.065860 | Cells_RadialDistribution_RadialCV_Ph_golgi_1of4 |
| 241 | 0.065644 | Cells_Texture_AngularSecondMoment_Mito_5 |
| 242 | 0.065279 | Cells_Intensity_MedianIntensity_Hoechst |
| 243 | 0.065178 | Nuclei_Texture_Correlation_Ph_golgi_5 |
| 244 | 0.064980 | Nuclei_Texture_Gabor_ER_3 |
| 245 | 0.064857 | Nuclei_Texture_Gabor_Ph_golgi_3 |
| 246 | 0.064539 | Cytoplasm_Intensity_UpperQuartileIntensity_Syto |
| 247 | 0.064158 | Cytoplasm_Intensity_MeanIntensityEdge_Syto |
| 248 | 0.064036 | Nuclei_Intensity_MinIntensity_Syto |
| 249 | 0.063903 | Nuclei_Intensity_MinIntensityEdge_Syto |
| 250 | 0.063815 | Nuclei_Texture_Gabor_Mito_5 |
| 251 | 0.063465 | Cells_Intensity_MedianIntensity_Syto |
| 252 | 0.063376 | Cells_Texture_Variance_Ph_golgi_5 |
| 253 | 0.063266 | Nuclei_Texture_Correlation_Syto_5 |
| 254 | 0.062619 | Cells_Texture_AngularSecondMoment_Hoechst_3 |
| 255 | 0.061978 | Cytoplasm_Texture_InfoMeas1_Ph_golgi_3 |
| 256 | 0.061787 | Nuclei_Intensity_MassDisplacement_Syto |
| 257 | 0.061499 | Cytoplasm_Intensity_MeanIntensity_Hoechst |
| 258 | 0.060704 | Cells_RadialDistribution_RadialCV_Ph_golgi_4of4 |
| 259 | 0.060550 | Cytoplasm_Texture_DifferenceVariance_Hoechst_3 |
| 260 | 0.060464 | Cells_Texture_Correlation_Ph_golgi_3 |
| 261 | 0.060439 | Cytoplasm_Intensity_MeanIntensity_Syto |
| 262 | 0.059959 | Cells_Texture_Variance_Ph_golgi_3 |
| 263 | 0.059716 | Nuclei_Texture_Gabor_Syto_5 |
| 264 | 0.059196 | Cytoplasm_Intensity_IntegratedIntensityEdge_Syto |
| 265 | 0.059174 | Cytoplasm_Intensity_UpperQuartileIntensity_Hoechst |
| 266 | 0.059106 | Cells_Intensity_MassDisplacement_Hoechst |
| 267 | 0.058251 | Cells_Texture_Gabor_Hoechst_3 |
| 268 | 0.058000 | Cytoplasm_Texture_Entropy_Hoechst_3 |
| 269 | 0.057868 | Nuclei_Texture_Gabor_Syto_3 |
| 270 | 0.057792 | Cytoplasm_Texture_DifferenceVariance_Hoechst_5 |
| 271 | 0.057463 | Cytoplasm_Texture_Gabor_Ph_golgi_3 |
| 272 | 0.056951 | Cytoplasm_Intensity_MedianIntensity_Syto |
| 273 | 0.056910 | Cells_Intensity_MeanIntensityEdge_Hoechst |
| 274 | 0.056710 | Nuclei_Texture_Contrast_Syto_5 |
| 275 | 0.056523 | Cytoplasm_Texture_Entropy_Hoechst_5 |
| 276 | 0.056418 | Cells_AreaShape_Area |
| 277 | 0.055728 | Nuclei_Texture_Gabor_ER_5 |
| 278 | 0.055542 | Cytoplasm_Texture_Gabor_Ph_golgi_5 |
| 279 | 0.055351 | Cytoplasm_Texture_SumEntropy_Hoechst_3 |
| 280 | 0.055044 | Cells_Intensity_MeanIntensityEdge_Syto |
| 281 | 0.054615 | Nuclei_Texture_Contrast_Syto_3 |
| 282 | 0.054329 | Nuclei_Texture_InfoMeas1_Mito_3 |
| 283 | 0.054029 | Cells_Intensity_LowerQuartileIntensity_Syto |
| 284 | 0.054025 | Nuclei_Texture_InfoMeas1_Mito_5 |
| 285 | 0.053958 | Cells_Texture_AngularSecondMoment_ER_5 |
| 286 | 0.053483 | Cells_Texture_Contrast_Hoechst_3 |
| 287 | 0.053298 | Cells_Texture_AngularSecondMoment_ER_3 |
| 288 | 0.053246 | Cytoplasm_Texture_SumEntropy_Hoechst_5 |
| 289 | 0.053033 | Cytoplasm_Texture_Contrast_Ph_golgi_3 |
| 290 | 0.052499 | Cells_Texture_Gabor_Ph_golgi_3 |
| 291 | 0.052158 | Cells_Texture_InfoMeas2_Ph_golgi_3 |
| 292 | 0.052140 | Cytoplasm_Intensity_MedianIntensity_Hoechst |
| 293 | 0.051760 | Cytoplasm_Intensity_LowerQuartileIntensity_Syto |
| 294 | 0.051489 | Cells_Intensity_LowerQuartileIntensity_Hoechst |
| 295 | 0.051162 | Cells_Texture_Contrast_Ph_golgi_3 |
| 296 | 0.050298 | Cytoplasm_Intensity_LowerQuartileIntensity_Hoechst |
| 297 | 0.049958 | Nuclei_Texture_AngularSecondMoment_Ph_golgi_3 |
| 298 | 0.049794 | Cells_Texture_Contrast_Hoechst_5 |
| 299 | 0.049282 | Nuclei_Texture_Contrast_Ph_golgi_3 |
| 300 | 0.049062 | Cytoplasm_Texture_DifferenceEntropy_Hoechst_3 |
| 301 | 0.048839 | Cells_Texture_Gabor_ER_3 |
| 302 | 0.048604 | Cytoplasm_Texture_Contrast_Ph_golgi_5 |
| 303 | 0.048343 | Cytoplasm_Intensity_MinIntensityEdge_Hoechst |
| 304 | 0.048343 | Cells_Intensity_MinIntensityEdge_Hoechst |
| 305 | 0.048074 | Cells_Intensity_IntegratedIntensityEdge_Hoechst |
| 306 | 0.048027 | Cytoplasm_Intensity_MinIntensity_Hoechst |
| 307 | 0.048026 | Cells_Intensity_MinIntensity_Hoechst |
| 308 | 0.047931 | Cytoplasm_Texture_DifferenceEntropy_Hoechst_5 |
| 309 | 0.047624 | Cells_Intensity_IntegratedIntensityEdge_Syto |
| 310 | 0.047351 | Cytoplasm_Texture_Contrast_ER_3 |
| 311 | 0.047173 | Nuclei_Texture_AngularSecondMoment_Ph_golgi_5 |
| 312 | 0.046819 | Cytoplasm_Texture_SumVariance_ER_5 |
| 313 | 0.045994 | Nuclei_Texture_SumVariance_Mito_5 |
| 314 | 0.045639 | Cytoplasm_Texture_Correlation_Ph_golgi_5 |
| 315 | 0.045570 | Nuclei_Texture_Correlation_Ph_golgi_3 |
| 316 | 0.045466 | Cytoplasm_Texture_SumVariance_ER_3 |
| 317 | 0.044037 | Nuclei_Texture_Gabor_Hoechst_3 |
| 318 | 0.043528 | Nuclei_Texture_SumVariance_Mito_3 |
| 319 | 0.043494 | Cytoplasm_Texture_AngularSecondMoment_Syto_3 |
| 320 | 0.043219 | Cytoplasm_Texture_SumAverage_Ph_golgi_3 |
| 321 | 0.043018 | Cytoplasm_Texture_SumAverage_Ph_golgi_5 |
| 322 | 0.042737 | Nuclei_Texture_Gabor_Ph_golgi_5 |
| 323 | 0.042609 | Cytoplasm_Texture_Contrast_ER_5 |
| 324 | 0.042552 | Nuclei_Texture_SumAverage_Mito_5 |
| 325 | 0.042417 | Cells_Texture_SumVariance_Syto_5 |
| 326 | 0.042407 | Cytoplasm_Texture_AngularSecondMoment_Syto_5 |
| 327 | 0.042261 | Cytoplasm_Texture_AngularSecondMoment_Hoechst_5 |
| 328 | 0.042081 | Cytoplasm_Texture_Variance_ER_3 |
| 329 | 0.041997 | Cells_Intensity_MinIntensityEdge_Syto |
| 330 | 0.041988 | Cytoplasm_Intensity_MinIntensityEdge_Syto |
| 331 | 0.041913 | Nuclei_Texture_SumAverage_Mito_3 |
| 332 | 0.041879 | Cells_Texture_Contrast_Syto_3 |
| 333 | 0.041535 | Cytoplasm_Texture_Variance_ER_5 |
| 334 | 0.041174 | Cells_Texture_SumVariance_Syto_3 |
| 335 | 0.040975 | Nuclei_Texture_Contrast_Ph_golgi_5 |
| 336 | 0.040786 | Cells_Intensity_MinIntensity_Syto |
| 337 | 0.040785 | Cytoplasm_Intensity_MinIntensity_Syto |
| 338 | 0.040289 | Cytoplasm_Texture_Contrast_Syto_3 |
| 339 | 0.040037 | Nuclei_Texture_InfoMeas1_ER_5 |
| 340 | 0.039512 | Cytoplasm_Texture_AngularSecondMoment_Hoechst_3 |
| 341 | 0.039360 | Nuclei_Texture_Contrast_Mito_3 |
| 342 | 0.039228 | Cells_Texture_InfoMeas1_Mito_5 |
| 343 | 0.039076 | Cytoplasm_Texture_Gabor_Hoechst_5 |
| 344 | 0.039039 | Cells_Texture_DifferenceVariance_Hoechst_3 |
| 345 | 0.038686 | Cells_Texture_Entropy_Hoechst_3 |
| 346 | 0.038626 | Cytoplasm_Texture_SumVariance_Syto_5 |
| 347 | 0.038552 | Cytoplasm_AreaShape_Zernike_2_0 |
| 348 | 0.038292 | Cells_Texture_Contrast_Ph_golgi_5 |
| 349 | 0.038270 | Cells_RadialDistribution_FracAtD_Ph_golgi_1of4 |
| 350 | 0.037990 | Cells_RadialDistribution_FracAtD_Syto_1of4 |
| 351 | 0.037951 | Nuclei_Texture_AngularSecondMoment_Syto_3 |
| 352 | 0.037932 | Cytoplasm_Texture_Contrast_Mito_3 |
| 353 | 0.037881 | Cells_Texture_Gabor_Hoechst_5 |
| 354 | 0.037751 | Nuclei_Texture_SumAverage_Ph_golgi_5 |
| 355 | 0.037599 | Cytoplasm_Texture_SumVariance_Syto_3 |
| 356 | 0.037410 | Cells_Texture_Variance_Syto_3 |
| 357 | 0.037359 | Cytoplasm_Texture_InfoMeas2_Hoechst_5 |
| 358 | 0.037287 | Cells_Texture_Entropy_Hoechst_5 |
| 359 | 0.037213 | Cells_Texture_Gabor_Ph_golgi_5 |
| 360 | 0.037178 | Cells_Texture_SumAverage_Syto_3 |
| 361 | 0.037041 | Cytoplasm_Texture_Contrast_Syto_5 |
| 362 | 0.036854 | Cells_Texture_Variance_Syto_5 |
| 363 | 0.036837 | Cells_Texture_SumAverage_Syto_5 |
| 364 | 0.036664 | Cytoplasm_Texture_Correlation_Hoechst_5 |
| 365 | 0.036498 | Nuclei_Texture_SumAverage_Ph_golgi_3 |
| 366 | 0.036451 | Cells_Texture_Contrast_Syto_5 |
| 367 | 0.036431 | Nuclei_Texture_InfoMeas1_ER_3 |
| 368 | 0.036429 | Cells_Texture_InfoMeas1_Mito_3 |
| 369 | 0.036284 | Nuclei_Texture_InfoMeas1_Syto_5 |
| 370 | 0.036074 | Cytoplasm_Texture_DifferenceVariance_Ph_golgi_3 |
| 371 | 0.035966 | Nuclei_AreaShape_Zernike_3_3 |
| 372 | 0.035501 | Cells_Texture_Gabor_ER_5 |
| 373 | 0.035499 | Cells_Texture_SumEntropy_Hoechst_3 |
| 374 | 0.035343 | Cells_Texture_DifferenceVariance_Ph_golgi_3 |
| 375 | 0.034905 | Nuclei_Texture_Correlation_ER_5 |
| 376 | 0.034859 | Nuclei_Texture_InfoMeas1_Syto_3 |
| 377 | 0.034736 | Nuclei_Texture_SumAverage_Syto_3 |
| 378 | 0.034489 | Nuclei_Texture_InfoMeas2_Ph_golgi_5 |
| 379 | 0.034330 | Nuclei_Texture_DifferenceVariance_Ph_golgi_3 |
| 380 | 0.034248 | Cytoplasm_Texture_Contrast_Mito_5 |
| 381 | 0.034223 | Nuclei_AreaShape_Zernike_5_5 |
| 382 | 0.034147 | Cytoplasm_Texture_Gabor_Hoechst_3 |
| 383 | 0.033935 | Nuclei_Texture_SumAverage_Syto_5 |
| 384 | 0.033824 | Cells_AreaShape_Perimeter |
| 385 | 0.033783 | Cytoplasm_Texture_SumAverage_Mito_5 |
| 386 | 0.033747 | Nuclei_Texture_InfoMeas2_Ph_golgi_3 |
| 387 | 0.033467 | Cells_Texture_SumEntropy_Hoechst_5 |
| 388 | 0.033460 | Cytoplasm_Texture_SumAverage_Mito_3 |
| 389 | 0.033458 | Cells_Texture_InfoMeas1_Syto_5 |
| 390 | 0.033394 | Cells_Texture_DifferenceVariance_Syto_3 |
| 391 | 0.033268 | Cells_Texture_SumAverage_Hoechst_5 |
| 392 | 0.033202 | Cells_Texture_SumAverage_Mito_3 |
| 393 | 0.033153 | Cells_Texture_SumAverage_Mito_5 |
| 394 | 0.032849 | Cells_Texture_InfoMeas1_ER_5 |
| 395 | 0.032661 | Cytoplasm_Texture_DifferenceVariance_Ph_golgi_5 |
| 396 | 0.032541 | Cytoplasm_Texture_InfoMeas1_Syto_3 |
| 397 | 0.032528 | Cells_Texture_SumAverage_Hoechst_3 |
| 398 | 0.032490 | Cells_Texture_DifferenceVariance_Hoechst_5 |
| 399 | 0.032325 | Cytoplasm_Texture_InfoMeas1_Syto_5 |
| 400 | 0.032273 | Cells_RadialDistribution_MeanFrac_Ph_golgi_1of4 |
| 401 | 0.032220 | Nuclei_AreaShape_Zernike_7_7 |
| 402 | 0.032096 | Cells_Texture_Contrast_ER_3 |
| 403 | 0.032056 | Cytoplasm_Texture_Variance_Syto_3 |
| 404 | 0.032034 | Cells_Texture_DifferenceEntropy_Hoechst_3 |
| 405 | 0.031968 | Nuclei_Texture_Entropy_Mito_3 |
| 406 | 0.031953 | Nuclei_Texture_Entropy_Mito_5 |
| 407 | 0.031590 | Cytoplasm_Texture_Variance_Syto_5 |
| 408 | 0.031468 | Cells_Texture_Gabor_Mito_5 |
| 409 | 0.031344 | Nuclei_Texture_DifferenceVariance_Ph_golgi_5 |
| 410 | 0.031171 | Nuclei_AreaShape_Zernike_5_3 |
| 411 | 0.031059 | Nuclei_Texture_SumVariance_ER_5 |
| 412 | 0.030843 | Cells_RadialDistribution_FracAtD_ER_1of4 |
| 413 | 0.030744 | Cells_Texture_InfoMeas1_Syto_3 |
| 414 | 0.030568 | Nuclei_Texture_Variance_Mito_3 |
| 415 | 0.030460 | Cytoplasm_Texture_DifferenceVariance_ER_3 |
| 416 | 0.030373 | Nuclei_Texture_Contrast_Mito_5 |
| 417 | 0.030296 | Nuclei_AreaShape_Zernike_9_9 |
| 418 | 0.030136 | Cytoplasm_Texture_Gabor_Mito_3 |
| 419 | 0.030097 | Cells_Texture_DifferenceEntropy_Hoechst_5 |
| 420 | 0.029869 | Nuclei_Texture_Correlation_Syto_3 |
| 421 | 0.029754 | Cytoplasm_Texture_SumAverage_ER_5 |
| 422 | 0.029698 | Cells_RadialDistribution_FracAtD_Ph_golgi_2of4 |
| 423 | 0.029543 | Nuclei_Texture_Variance_Mito_5 |
| 424 | 0.029536 | Nuclei_Texture_Correlation_Mito_5 |
| 425 | 0.029427 | Cells_Texture_InfoMeas1_ER_3 |
| 426 | 0.029235 | Cytoplasm_Texture_SumAverage_ER_3 |
| 427 | 0.029227 | Cells_RadialDistribution_FracAtD_Mito_1of4 |
| 428 | 0.029170 | Nuclei_Texture_AngularSecondMoment_Syto_5 |
| 429 | 0.029054 | Cytoplasm_Texture_DifferenceVariance_Syto_3 |
| 430 | 0.029011 | Cells_RadialDistribution_FracAtD_Syto_2of4 |
| 431 | 0.028884 | Cells_Texture_DifferenceVariance_Syto_5 |
| 432 | 0.028694 | Cells_Texture_SumVariance_Mito_5 |
| 433 | 0.028656 | Nuclei_AreaShape_Zernike_1_1 |
| 434 | 0.028320 | Nuclei_Texture_SumVariance_Ph_golgi_3 |
| 435 | 0.028241 | Cytoplasm_Texture_Gabor_Syto_5 |
| 436 | 0.028113 | Cells_RadialDistribution_MeanFrac_Syto_1of4 |
| 437 | 0.028094 | Cytoplasm_Texture_Gabor_Syto_3 |
| 438 | 0.028075 | Cytoplasm_Texture_Entropy_ER_3 |
| 439 | 0.027905 | Nuclei_Intensity_MassDisplacement_Hoechst |
| 440 | 0.027746 | Nuclei_AreaShape_Zernike_3_1 |
| 441 | 0.027653 | Cells_AreaShape_MajorAxisLength |
| 442 | 0.027623 | Cells_RadialDistribution_FracAtD_ER_2of4 |
| 443 | 0.027030 | Cytoplasm_Texture_Correlation_Ph_golgi_3 |
| 444 | 0.026994 | Cytoplasm_AreaShape_Solidity |
| 445 | 0.026889 | Cells_Texture_Contrast_Mito_3 |
| 446 | 0.026778 | Nuclei_Texture_SumVariance_ER_3 |
| 447 | 0.026740 | Cytoplasm_Texture_InfoMeas1_Mito_3 |
| 448 | 0.026676 | Nuclei_Texture_SumVariance_Ph_golgi_5 |
| 449 | 0.026675 | Cells_Texture_SumVariance_Mito_3 |
| 450 | 0.026644 | Cytoplasm_Texture_InfoMeas2_Hoechst_3 |
| 451 | 0.026597 | Nuclei_Texture_Contrast_ER_3 |
| 452 | 0.026566 | Cytoplasm_Texture_Variance_Ph_golgi_3 |
| 453 | 0.026504 | Cytoplasm_Texture_Entropy_ER_5 |
| 454 | 0.026381 | Cytoplasm_Texture_Entropy_Ph_golgi_3 |
| 455 | 0.026298 | Cells_Texture_DifferenceVariance_Ph_golgi_5 |
| 456 | 0.026252 | Cytoplasm_Texture_Gabor_ER_5 |
| 457 | 0.026133 | Cytoplasm_Texture_DifferenceVariance_Syto_5 |
| 458 | 0.026024 | Cells_Texture_Variance_Hoechst_3 |
| 459 | 0.025911 | Cytoplasm_Texture_InfoMeas1_Hoechst_5 |
| 460 | 0.025898 | Cells_Texture_SumVariance_ER_3 |
| 461 | 0.025876 | Cells_Texture_Contrast_ER_5 |
| 462 | 0.025845 | Cytoplasm_AreaShape_Extent |
| 463 | 0.025797 | Cells_Texture_SumVariance_ER_5 |
| 464 | 0.025715 | Cytoplasm_Texture_InfoMeas1_ER_5 |
| 465 | 0.025709 | Cytoplasm_Texture_Variance_Ph_golgi_5 |
| 466 | 0.025667 | Cytoplasm_Texture_Entropy_Ph_golgi_5 |
| 467 | 0.025627 | Cells_RadialDistribution_FracAtD_Mito_2of4 |
| 468 | 0.025607 | Nuclei_Texture_SumAverage_ER_5 |
| 469 | 0.025461 | Cytoplasm_Texture_SumEntropy_ER_3 |
| 470 | 0.025298 | Cytoplasm_Texture_SumVariance_Ph_golgi_3 |
| 471 | 0.025295 | Cells_AreaShape_MinorAxisLength |
| 472 | 0.025110 | Cells_Texture_InfoMeas1_Hoechst_5 |
| 473 | 0.025110 | Cytoplasm_Texture_InfoMeas1_ER_3 |
| 474 | 0.025069 | Nuclei_Texture_Entropy_ER_5 |
| 475 | 0.024942 | Cells_Texture_SumAverage_Ph_golgi_3 |
| 476 | 0.024922 | Cells_Texture_Variance_Hoechst_5 |
| 477 | 0.024755 | Cells_Texture_SumVariance_Hoechst_3 |
| 478 | 0.024708 | Cells_RadialDistribution_MeanFrac_Ph_golgi_2of4 |
| 479 | 0.024647 | Cytoplasm_Texture_Gabor_ER_3 |
| 480 | 0.024643 | Nuclei_Texture_SumAverage_ER_3 |
| 481 | 0.024638 | Cytoplasm_Texture_DifferenceVariance_ER_5 |
| 482 | 0.024520 | Cytoplasm_AreaShape_Zernike_6_0 |
| 483 | 0.024473 | Cytoplasm_Texture_InfoMeas2_Ph_golgi_5 |
| 484 | 0.024463 | Cytoplasm_Texture_DifferenceVariance_Mito_3 |
| 485 | 0.024390 | Cytoplasm_AreaShape_Perimeter |
| 486 | 0.024186 | Cytoplasm_Texture_DifferenceVariance_Mito_5 |
| 487 | 0.024179 | Cytoplasm_AreaShape_Zernike_4_0 |
| 488 | 0.024167 | Nuclei_AreaShape_Zernike_7_5 |
| 489 | 0.024125 | Nuclei_Texture_Entropy_ER_3 |
| 490 | 0.024115 | Cytoplasm_Texture_SumVariance_Ph_golgi_5 |
| 491 | 0.024091 | Cytoplasm_Texture_SumEntropy_ER_5 |
| 492 | 0.023866 | Cytoplasm_AreaShape_MajorAxisLength |
| 493 | 0.023813 | Cytoplasm_Texture_SumVariance_Mito_3 |
| 494 | 0.023748 | Cells_Texture_SumAverage_Ph_golgi_5 |
| 495 | 0.023710 | Nuclei_Texture_InfoMeas2_Mito_5 |
| 496 | 0.023597 | Nuclei_Texture_Correlation_Mito_3 |
| 497 | 0.023483 | Cytoplasm_Texture_Variance_Mito_3 |
| 498 | 0.023448 | Cytoplasm_AreaShape_Zernike_0_0 |
| 499 | 0.023425 | Cells_Texture_Variance_ER_3 |
| 500 | 0.023258 | Cytoplasm_Texture_InfoMeas1_Hoechst_3 |
| 501 | 0.023236 | Cytoplasm_Texture_SumVariance_Mito_5 |
| 502 | 0.023189 | Nuclei_Texture_SumEntropy_Mito_3 |
| 503 | 0.023080 | Nuclei_Texture_InfoMeas2_ER_5 |
| 504 | 0.023043 | Cytoplasm_Texture_Variance_Mito_5 |
| 505 | 0.022935 | Cytoplasm_AreaShape_MinorAxisLength |
| 506 | 0.022821 | Cells_Texture_Variance_ER_5 |
| 507 | 0.022799 | Cells_Texture_SumAverage_ER_3 |
| 508 | 0.022789 | Cytoplasm_Texture_InfoMeas1_Mito_5 |
| 509 | 0.022732 | Cytoplasm_Texture_SumAverage_Hoechst_3 |
| 510 | 0.022694 | Cells_Texture_Variance_Mito_5 |
| 511 | 0.022678 | Nuclei_Texture_SumVariance_Syto_3 |
| 512 | 0.022574 | Cytoplasm_Texture_SumAverage_Hoechst_5 |
| 513 | 0.022550 | Cells_Texture_SumAverage_ER_5 |
| 514 | 0.022544 | Cytoplasm_Texture_Correlation_Hoechst_3 |
| 515 | 0.022466 | Cells_Texture_Variance_Mito_3 |
| 516 | 0.022464 | Nuclei_Texture_Variance_Syto_5 |
| 517 | 0.022412 | Cells_Texture_DifferenceVariance_Mito_5 |
| 518 | 0.022275 | Cells_Texture_SumVariance_Hoechst_5 |
| 519 | 0.022126 | Cytoplasm_Texture_DifferenceEntropy_Ph_golgi_3 |
| 520 | 0.022124 | Nuclei_Texture_Variance_Ph_golgi_3 |
| 521 | 0.022102 | Nuclei_Texture_SumEntropy_Mito_5 |
| 522 | 0.022046 | Nuclei_Texture_Variance_Ph_golgi_5 |
| 523 | 0.022031 | Cytoplasm_Texture_DifferenceEntropy_ER_3 |
| 524 | 0.021935 | Nuclei_Texture_InfoMeas2_Mito_3 |
| 525 | 0.021850 | Cytoplasm_Texture_Entropy_Mito_3 |
| 526 | 0.021827 | Nuclei_Texture_Correlation_ER_3 |
| 527 | 0.021734 | Nuclei_AreaShape_Zernike_5_1 |
| 528 | 0.021644 | Cytoplasm_Texture_SumAverage_Syto_5 |
| 529 | 0.021566 | Cytoplasm_Texture_SumAverage_Syto_3 |
| 530 | 0.021560 | Nuclei_Texture_Contrast_ER_5 |
| 531 | 0.021376 | Cells_Texture_DifferenceEntropy_Ph_golgi_3 |
| 532 | 0.021321 | Nuclei_Texture_DifferenceVariance_Mito_3 |
| 533 | 0.021109 | Nuclei_Texture_InfoMeas1_Hoechst_3 |
| 534 | 0.021101 | Cells_RadialDistribution_FracAtD_Syto_4of4 |
| 535 | 0.021018 | Cells_RadialDistribution_FracAtD_ER_4of4 |
| 536 | 0.020918 | Cytoplasm_AreaShape_Zernike_2_2 |
| 537 | 0.020658 | Cells_RadialDistribution_MeanFrac_Syto_2of4 |
| 538 | 0.020620 | Nuclei_Texture_Variance_Syto_3 |
| 539 | 0.020518 | Nuclei_Texture_SumVariance_Syto_5 |
| 540 | 0.020463 | Cytoplasm_Texture_InverseDifferenceMoment_Ph_golgi_5 |
| 541 | 0.020298 | Nuclei_Texture_SumEntropy_ER_3 |
| 542 | 0.020201 | Cells_Neighbors_PercentTouching_5 |
| 543 | 0.020122 | Cytoplasm_Texture_Entropy_Mito_5 |
| 544 | 0.020055 | Cytoplasm_Texture_DifferenceEntropy_Ph_golgi_5 |
| 545 | 0.019974 | Nuclei_Texture_SumEntropy_ER_5 |
| 546 | 0.019919 | Nuclei_AreaShape_Zernike_9_7 |
| 547 | 0.019892 | Cells_Texture_Correlation_Mito_5 |
| 548 | 0.019727 | Cells_Texture_Contrast_Mito_5 |
| 549 | 0.019709 | Nuclei_AreaShape_Zernike_4_2 |
| 550 | 0.019671 | Nuclei_Texture_InverseDifferenceMoment_Mito_5 |
| 551 | 0.019379 | Cells_Texture_DifferenceVariance_ER_3 |
| 552 | 0.019342 | Cells_Texture_Entropy_Syto_3 |
| 553 | 0.019291 | Cells_Texture_Correlation_Syto_5 |
| 554 | 0.019242 | Cells_Texture_DifferenceVariance_Mito_3 |
| 555 | 0.019063 | Nuclei_AreaShape_Zernike_8_8 |
| 556 | 0.018846 | Cytoplasm_Texture_SumEntropy_Mito_3 |
| 557 | 0.018740 | Nuclei_Texture_SumVariance_Hoechst_3 |
| 558 | 0.018658 | Cytoplasm_Texture_Gabor_Mito_5 |
| 559 | 0.018656 | Nuclei_Texture_DifferenceVariance_Mito_5 |
| 560 | 0.018640 | Cytoplasm_Texture_DifferenceEntropy_ER_5 |
| 561 | 0.018621 | Cells_RadialDistribution_FracAtD_Mito_4of4 |
| 562 | 0.018590 | Cytoplasm_Texture_InverseDifferenceMoment_Ph_golgi_3 |
| 563 | 0.018506 | Cells_RadialDistribution_MeanFrac_ER_1of4 |
| 564 | 0.018457 | Cytoplasm_Texture_InfoMeas2_Ph_golgi_3 |
| 565 | 0.018256 | Cytoplasm_AreaShape_Zernike_8_0 |
| 566 | 0.018184 | Cytoplasm_Texture_SumEntropy_Ph_golgi_3 |
| 567 | 0.018086 | Cells_Texture_InfoMeas1_Hoechst_3 |
| 568 | 0.017985 | Nuclei_Texture_InverseDifferenceMoment_Mito_3 |
| 569 | 0.017981 | Nuclei_Texture_InfoMeas2_ER_3 |
| 570 | 0.017967 | Cells_Texture_Entropy_ER_3 |
| 571 | 0.017878 | Nuclei_Texture_SumVariance_Hoechst_5 |
| 572 | 0.017791 | Nuclei_Texture_DifferenceEntropy_Syto_3 |
| 573 | 0.017747 | Nuclei_Texture_DifferenceEntropy_Syto_5 |
| 574 | 0.017600 | Nuclei_Texture_Gabor_Hoechst_5 |
| 575 | 0.017574 | Nuclei_Texture_DifferenceVariance_Hoechst_5 |
| 576 | 0.017434 | Cells_RadialDistribution_FracAtD_Ph_golgi_4of4 |
| 577 | 0.017326 | Nuclei_Texture_DifferenceEntropy_Mito_3 |
| 578 | 0.017315 | Nuclei_Texture_DifferenceEntropy_Ph_golgi_3 |
| 579 | 0.017189 | Nuclei_Texture_Variance_ER_3 |
| 580 | 0.017126 | Cells_Texture_Entropy_Syto_5 |
| 581 | 0.017085 | Cells_Texture_Entropy_Mito_3 |
| 582 | 0.017071 | Cytoplasm_Texture_SumEntropy_Mito_5 |
| 583 | 0.016965 | Cells_RadialDistribution_MeanFrac_Mito_1of4 |
| 584 | 0.016874 | Cells_Texture_InverseDifferenceMoment_Hoechst_5 |
| 585 | 0.016770 | Nuclei_Texture_InfoMeas1_Hoechst_5 |
| 586 | 0.016561 | Cytoplasm_Texture_Correlation_Syto_5 |
| 587 | 0.016494 | Nuclei_AreaShape_Zernike_6_6 |
| 588 | 0.016444 | Cytoplasm_Texture_SumEntropy_Ph_golgi_5 |
| 589 | 0.016442 | Cells_Texture_DifferenceEntropy_Syto_3 |
| 590 | 0.016429 | Cells_Texture_DifferenceVariance_ER_5 |
| 591 | 0.016323 | Cells_Texture_DifferenceEntropy_Ph_golgi_5 |
| 592 | 0.016231 | Nuclei_Texture_DifferenceVariance_Hoechst_3 |
| 593 | 0.016162 | Nuclei_Texture_Variance_ER_5 |
| 594 | 0.016122 | Nuclei_Texture_Variance_Hoechst_3 |
| 595 | 0.016032 | Nuclei_Texture_InfoMeas2_Syto_5 |
| 596 | 0.015967 | Cells_Texture_Correlation_ER_5 |
| 597 | 0.015938 | Cells_Texture_Entropy_ER_5 |
| 598 | 0.015926 | Cells_RadialDistribution_MeanFrac_ER_2of4 |
| 599 | 0.015910 | Cells_Texture_DifferenceEntropy_ER_3 |
| 600 | 0.015908 | Cytoplasm_Texture_Correlation_ER_5 |
| 601 | 0.015843 | Nuclei_Texture_Variance_Hoechst_5 |
| 602 | 0.015788 | Cells_AreaShape_Zernike_2_2 |
| 603 | 0.015768 | Cells_Texture_InverseDifferenceMoment_Ph_golgi_3 |
| 604 | 0.015667 | Nuclei_Texture_Entropy_Ph_golgi_3 |
| 605 | 0.015627 | Cells_Texture_SumEntropy_ER_3 |
| 606 | 0.015479 | Nuclei_AreaShape_Zernike_9_5 |
| 607 | 0.015451 | Cells_Texture_InfoMeas2_Mito_5 |
| 608 | 0.015365 | Nuclei_Texture_InverseDifferenceMoment_Ph_golgi_3 |
| 609 | 0.015357 | Cells_Texture_Entropy_Mito_5 |
| 610 | 0.015342 | Nuclei_AreaShape_Zernike_8_0 |
| 611 | 0.015294 | Cells_Texture_SumEntropy_Mito_3 |
| 612 | 0.015294 | Nuclei_AreaShape_Zernike_7_3 |
| 613 | 0.015249 | Nuclei_AreaShape_Zernike_6_0 |
| 614 | 0.015179 | Cells_RadialDistribution_MeanFrac_Syto_4of4 |
| 615 | 0.014964 | Nuclei_Texture_InfoMeas2_Syto_3 |
| 616 | 0.014915 | Nuclei_Texture_DifferenceVariance_ER_3 |
| 617 | 0.014869 | Cytoplasm_Texture_DifferenceEntropy_Mito_3 |
| 618 | 0.014803 | Cytoplasm_AreaShape_Zernike_7_1 |
| 619 | 0.014727 | Nuclei_Texture_DifferenceEntropy_Mito_5 |
| 620 | 0.014688 | Cytoplasm_Texture_InfoMeas2_ER_5 |
| 621 | 0.014600 | Cells_Texture_InverseDifferenceMoment_Ph_golgi_5 |
| 622 | 0.014600 | Nuclei_Texture_Entropy_Ph_golgi_5 |
| 623 | 0.014505 | Cytoplasm_Texture_DifferenceEntropy_Syto_3 |
| 624 | 0.014464 | Nuclei_Texture_DifferenceEntropy_Ph_golgi_5 |
| 625 | 0.014413 | Cells_Texture_SumEntropy_Syto_3 |
| 626 | 0.014344 | Cytoplasm_AreaShape_Zernike_4_4 |
| 627 | 0.014281 | Cytoplasm_Texture_InfoMeas2_Syto_5 |
| 628 | 0.014070 | Cytoplasm_Texture_InverseDifferenceMoment_Mito_5 |
| 629 | 0.013942 | Cells_Texture_SumEntropy_Ph_golgi_5 |
| 630 | 0.013852 | Cells_Texture_SumEntropy_ER_5 |
| 631 | 0.013830 | Cells_RadialDistribution_MeanFrac_Ph_golgi_4of4 |
| 632 | 0.013801 | Cells_RadialDistribution_MeanFrac_Mito_2of4 |
| 633 | 0.013720 | Cells_RadialDistribution_FracAtD_ER_3of4 |
| 634 | 0.013709 | Nuclei_Texture_Correlation_Hoechst_5 |
| 635 | 0.013685 | Cytoplasm_Texture_InverseDifferenceMoment_Hoechst_5 |
| 636 | 0.013668 | Cytoplasm_Texture_InverseDifferenceMoment_ER_5 |
| 637 | 0.013613 | Cytoplasm_Texture_Entropy_Syto_3 |
| 638 | 0.013598 | Cells_Texture_Correlation_Mito_3 |
| 639 | 0.013580 | Nuclei_Texture_InverseDifferenceMoment_Ph_golgi_5 |
| 640 | 0.013580 | Cells_Texture_SumEntropy_Mito_5 |
| 641 | 0.013573 | Nuclei_AreaShape_Zernike_4_0 |
| 642 | 0.013438 | Cells_RadialDistribution_FracAtD_Mito_3of4 |
| 643 | 0.013341 | Nuclei_AreaShape_Zernike_4_4 |
| 644 | 0.013279 | Cells_RadialDistribution_MeanFrac_ER_4of4 |
| 645 | 0.013035 | Cells_Texture_InverseDifferenceMoment_Syto_5 |
| 646 | 0.013021 | Cells_Neighbors_NumberOfNeighbors_5 |
| 647 | 0.012900 | Cells_Texture_Correlation_Syto_3 |
| 648 | 0.012872 | Nuclei_Texture_Contrast_Hoechst_5 |
| 649 | 0.012813 | Cytoplasm_AreaShape_Zernike_1_1 |
| 650 | 0.012781 | Nuclei_Texture_DifferenceVariance_ER_5 |
| 651 | 0.012781 | Cells_Texture_DifferenceEntropy_Syto_5 |
| 652 | 0.012689 | Nuclei_Texture_InverseDifferenceMoment_ER_5 |
| 653 | 0.012685 | Nuclei_AreaShape_Zernike_8_4 |
| 654 | 0.012554 | Cytoplasm_Texture_Entropy_Syto_5 |
| 655 | 0.012546 | Cytoplasm_Texture_InverseDifferenceMoment_Mito_3 |
| 656 | 0.012535 | Cells_Texture_InfoMeas2_ER_5 |
| 657 | 0.012499 | Cells_Texture_Entropy_Ph_golgi_3 |
| 658 | 0.012479 | Cells_Texture_SumEntropy_Syto_5 |
| 659 | 0.012446 | Cells_Texture_DifferenceEntropy_ER_5 |
| 660 | 0.012388 | Cells_AreaShape_Zernike_4_4 |
| 661 | 0.012383 | Nuclei_AreaShape_Zernike_8_2 |
| 662 | 0.012251 | Cytoplasm_Texture_Correlation_Syto_3 |
| 663 | 0.012145 | Cells_Texture_InfoMeas2_Mito_3 |
| 664 | 0.012127 | Cells_AreaShape_Solidity |
| 665 | 0.012041 | Cytoplasm_Texture_DifferenceEntropy_Mito_5 |
| 666 | 0.012020 | Cytoplasm_Texture_InfoMeas2_Syto_3 |
| 667 | 0.012008 | Cells_Texture_Correlation_ER_3 |
| 668 | 0.011958 | Cytoplasm_AreaShape_Zernike_6_6 |
| 669 | 0.011927 | Cells_Texture_InverseDifferenceMoment_Syto_3 |
| 670 | 0.011902 | Cytoplasm_Texture_Correlation_Mito_5 |
| 671 | 0.011883 | Cells_Texture_InverseDifferenceMoment_Hoechst_3 |
| 672 | 0.011830 | Cytoplasm_Texture_Correlation_ER_3 |
| 673 | 0.011790 | Nuclei_AreaShape_Area |
| 674 | 0.011758 | Cells_Texture_SumEntropy_Ph_golgi_3 |
| 675 | 0.011596 | Cytoplasm_Texture_DifferenceEntropy_Syto_5 |
| 676 | 0.011406 | Cytoplasm_Texture_SumEntropy_Syto_3 |
| 677 | 0.011405 | Cytoplasm_Texture_InverseDifferenceMoment_Hoechst_3 |
| 678 | 0.011362 | Cells_Texture_Entropy_Ph_golgi_5 |
| 679 | 0.011326 | Cells_AreaShape_Extent |
| 680 | 0.011244 | Cells_RadialDistribution_FracAtD_Ph_golgi_3of4 |
| 681 | 0.011142 | Cells_Texture_DifferenceEntropy_Mito_3 |
| 682 | 0.011114 | Cytoplasm_Texture_InverseDifferenceMoment_ER_3 |
| 683 | 0.011110 | Cytoplasm_Texture_InfoMeas2_ER_3 |
| 684 | 0.011034 | Cells_AreaShape_Zernike_6_6 |
| 685 | 0.011032 | Cells_Texture_InverseDifferenceMoment_Mito_5 |
| 686 | 0.011006 | Cells_AreaShape_Zernike_3_1 |
| 687 | 0.011000 | Cytoplasm_AreaShape_Zernike_8_2 |
| 688 | 0.011000 | Nuclei_Texture_DifferenceEntropy_ER_3 |
| 689 | 0.010990 | Nuclei_Texture_InverseDifferenceMoment_ER_3 |
| 690 | 0.010886 | Cytoplasm_Texture_SumEntropy_Syto_5 |
| 691 | 0.010774 | Cells_AreaShape_Zernike_4_2 |
| 692 | 0.010670 | Cytoplasm_AreaShape_Zernike_3_3 |
| 693 | 0.010561 | Cells_Texture_InverseDifferenceMoment_Mito_3 |
| 694 | 0.010555 | Nuclei_AreaShape_Zernike_6_2 |
| 695 | 0.010544 | Nuclei_AreaShape_Zernike_9_3 |
| 696 | 0.010512 | Cytoplasm_Texture_InverseDifferenceMoment_Syto_5 |
| 697 | 0.010456 | Nuclei_Texture_Contrast_Hoechst_3 |
| 698 | 0.010405 | Cytoplasm_AreaShape_Zernike_9_7 |
| 699 | 0.010380 | Nuclei_Texture_AngularSecondMoment_Hoechst_5 |
| 700 | 0.010291 | Cells_AreaShape_Zernike_0_0 |
| 701 | 0.010207 | Cells_Texture_InfoMeas2_Syto_5 |
| 702 | 0.010157 | Cells_RadialDistribution_MeanFrac_Mito_4of4 |
| 703 | 0.010029 | Cells_RadialDistribution_FracAtD_Syto_3of4 |
| 704 | 0.009971 | Nuclei_Texture_AngularSecondMoment_Hoechst_3 |
| 705 | 0.009897 | Cytoplasm_AreaShape_Zernike_8_8 |
| 706 | 0.009758 | Nuclei_Texture_DifferenceEntropy_ER_5 |
| 707 | 0.009736 | Cytoplasm_Texture_Correlation_Mito_3 |
| 708 | 0.009693 | Nuclei_Texture_Correlation_Hoechst_3 |
| 709 | 0.009683 | Cells_AreaShape_Zernike_9_7 |
| 710 | 0.009666 | Cells_Texture_InfoMeas2_ER_3 |
| 711 | 0.009624 | Cytoplasm_AreaShape_Zernike_3_1 |
| 712 | 0.009568 | Cells_Texture_InverseDifferenceMoment_ER_5 |
| 713 | 0.009466 | Cytoplasm_Texture_InverseDifferenceMoment_Syto_3 |
| 714 | 0.009463 | Cytoplasm_AreaShape_Zernike_5_5 |
| 715 | 0.009367 | Cells_AreaShape_Zernike_8_8 |
| 716 | 0.009233 | Nuclei_AreaShape_Zernike_7_1 |
| 717 | 0.008992 | Nuclei_Texture_InfoMeas2_Hoechst_3 |
| 718 | 0.008980 | Cytoplasm_AreaShape_Zernike_6_2 |
| 719 | 0.008691 | Cells_AreaShape_Zernike_5_5 |
| 720 | 0.008667 | Cells_AreaShape_Zernike_3_3 |
| 721 | 0.008667 | Cytoplasm_AreaShape_Zernike_4_2 |
| 722 | 0.008418 | Cells_Texture_InverseDifferenceMoment_ER_3 |
| 723 | 0.008348 | Cytoplasm_AreaShape_Zernike_5_1 |
| 724 | 0.008212 | Nuclei_Texture_SumEntropy_Ph_golgi_3 |
| 725 | 0.008086 | Cytoplasm_AreaShape_Zernike_7_5 |
| 726 | 0.008053 | Cytoplasm_Texture_InfoMeas2_Mito_5 |
| 727 | 0.007884 | Nuclei_Texture_SumEntropy_Ph_golgi_5 |
| 728 | 0.007880 | Nuclei_Texture_InfoMeas2_Hoechst_5 |
| 729 | 0.007840 | Cells_RadialDistribution_MeanFrac_Ph_golgi_3of4 |
| 730 | 0.007797 | Nuclei_AreaShape_Zernike_6_4 |
| 731 | 0.007749 | Cells_Texture_Correlation_Hoechst_5 |
| 732 | 0.007574 | Cells_Texture_InfoMeas2_Syto_3 |
| 733 | 0.007533 | Cytoplasm_AreaShape_Zernike_7_7 |
| 734 | 0.007476 | Cells_AreaShape_Zernike_6_2 |
| 735 | 0.007364 | Cytoplasm_Texture_InfoMeas2_Mito_3 |
| 736 | 0.007286 | Cells_AreaShape_Zernike_7_7 |
| 737 | 0.007120 | Cells_AreaShape_Zernike_4_0 |
| 738 | 0.007083 | Cells_Texture_DifferenceEntropy_Mito_5 |
| 739 | 0.007083 | Cytoplasm_AreaShape_Zernike_9_1 |
| 740 | 0.006992 | Nuclei_AreaShape_Zernike_0_0 |
| 741 | 0.006884 | Nuclei_AreaShape_Zernike_2_2 |
| 742 | 0.006805 | Nuclei_AreaShape_Zernike_2_0 |
| 743 | 0.006803 | Nuclei_AreaShape_MinorAxisLength |
| 744 | 0.006699 | Nuclei_AreaShape_MajorAxisLength |
| 745 | 0.006682 | Cells_RadialDistribution_MeanFrac_Syto_3of4 |
| 746 | 0.006506 | Cells_AreaShape_Zernike_9_9 |
| 747 | 0.006497 | Cytoplasm_AreaShape_Zernike_9_9 |
| 748 | 0.006455 | Nuclei_AreaShape_Eccentricity |
| 749 | 0.006220 | Cells_RadialDistribution_MeanFrac_ER_3of4 |
| 750 | 0.006154 | Cells_AreaShape_Zernike_7_5 |
| 751 | 0.006151 | Cytoplasm_AreaShape_Zernike_8_6 |
| 752 | 0.006137 | Nuclei_Texture_SumAverage_Hoechst_5 |
| 753 | 0.006020 | Nuclei_Texture_SumAverage_Hoechst_3 |
| 754 | 0.006011 | Cytoplasm_AreaShape_Zernike_5_3 |
| 755 | 0.006010 | Nuclei_Texture_InverseDifferenceMoment_Syto_5 |
| 756 | 0.005962 | Nuclei_AreaShape_Zernike_9_1 |
| 757 | 0.005688 | Nuclei_Texture_InverseDifferenceMoment_Syto_3 |
| 758 | 0.005646 | Cytoplasm_AreaShape_Zernike_9_5 |
| 759 | 0.005587 | Nuclei_AreaShape_Zernike_8_6 |
| 760 | 0.005561 | Cells_AreaShape_Zernike_8_6 |
| 761 | 0.005393 | Cells_AreaShape_Zernike_5_1 |
| 762 | 0.005390 | Cells_AreaShape_Zernike_8_4 |
| 763 | 0.005345 | Nuclei_Texture_Entropy_Syto_3 |
| 764 | 0.005236 | Cells_Texture_InfoMeas2_Hoechst_3 |
| 765 | 0.005130 | Cells_AreaShape_Zernike_6_4 |
| 766 | 0.005073 | Cytoplasm_AreaShape_Zernike_7_3 |
| 767 | 0.005024 | Cells_AreaShape_Zernike_7_3 |
| 768 | 0.004952 | Nuclei_Texture_Entropy_Syto_5 |
| 769 | 0.004879 | Nuclei_AreaShape_FormFactor |
| 770 | 0.004748 | Cytoplasm_AreaShape_Zernike_6_4 |
| 771 | 0.004682 | Cells_AreaShape_Zernike_8_0 |
| 772 | 0.004679 | Nuclei_Texture_SumEntropy_Syto_3 |
| 773 | 0.004652 | Cells_RadialDistribution_MeanFrac_Mito_3of4 |
| 774 | 0.004590 | Cytoplasm_AreaShape_Zernike_9_3 |
| 775 | 0.004556 | Cytoplasm_AreaShape_Zernike_8_4 |
| 776 | 0.004457 | Nuclei_AreaShape_Perimeter |
| 777 | 0.004285 | Nuclei_Texture_SumEntropy_Syto_5 |
| 778 | 0.004197 | Cells_AreaShape_Zernike_5_3 |
| 779 | 0.003916 | Cells_Texture_InfoMeas2_Hoechst_5 |
| 780 | 0.003715 | Cells_AreaShape_Eccentricity |
| 781 | 0.003628 | Cells_AreaShape_Zernike_6_0 |
| 782 | 0.003572 | Cells_Texture_Correlation_Hoechst_3 |
| 783 | 0.003438 | Nuclei_Texture_DifferenceEntropy_Hoechst_5 |
| 784 | 0.003319 | Cells_AreaShape_Zernike_7_1 |
| 785 | 0.003255 | Nuclei_AreaShape_Extent |
| 786 | 0.003159 | Nuclei_Texture_DifferenceEntropy_Hoechst_3 |
| 787 | 0.003051 | Cells_AreaShape_Zernike_2_0 |
| 788 | 0.003023 | Cells_AreaShape_Zernike_9_5 |
| 789 | 0.002974 | Cells_AreaShape_Zernike_9_1 |
| 790 | 0.002933 | Nuclei_Texture_SumEntropy_Hoechst_3 |
| 791 | 0.002903 | Nuclei_Texture_SumEntropy_Hoechst_5 |
| 792 | 0.002900 | Cytoplasm_AreaShape_Eccentricity |
| 793 | 0.002789 | Nuclei_Texture_InverseDifferenceMoment_Hoechst_5 |
| 794 | 0.002722 | Cells_AreaShape_Zernike_8_2 |
| 795 | 0.002428 | Cells_AreaShape_Zernike_9_3 |
| 796 | 0.002397 | Cells_AreaShape_Zernike_1_1 |
| 797 | 0.002065 | Nuclei_AreaShape_Solidity |
| 798 | 0.001925 | Nuclei_Texture_InverseDifferenceMoment_Hoechst_3 |
| 799 | 0.001690 | Nuclei_Texture_Entropy_Hoechst_5 |
| 800 | 0.001507 | Nuclei_Texture_Entropy_Hoechst_3 |
| 801 | 0.000027 | Cells_AreaShape_EulerNumber |
| 802 | 0.000000 | Nuclei_Children_Cytoplasm_Count |
| 803 | 0.000000 | Nuclei_AreaShape_EulerNumber |
| 804 | 0.000000 | Nuclei_Children_Cells_Count |
| 805 | 0.000000 | Cells_Children_Cytoplasm_Count |
