# Supplementary material for: Multiplex Cytological Profiling Assay to Measure Diverse Cellular States
Source: PLoS One. 2013 Dec 2;8(12):e80999. doi: 10.1371/journal.pone.0080999 (PMC3847047; doi:10.1371/journal.pone.0080999)
Supplement: Table S4 — Features ranked by well-to-well coefficient of variation (absolute), limited to mock-treated cells. (DOCX) [file pone.0080999.s012.docx]

**Table S4:** Features ranked by well-to-well coefficient of variation (absolute), limited to mock-treated cells

| **Rank** | **CV** | **Feature name** |
| --- | --- | --- |
| 1 | 3.223039 | Cytoplasm_AreaShape_Orientation |
| 2 | 2.988894 | Cells_AreaShape_Orientation |
| 3 | 2.288370 | Nuclei_AreaShape_Orientation |
| 4 | 0.224684 | Cytoplasm_AreaShape_EulerNumber |
| 5 | 0.207754 | Nuclei_Neighbors_NumberOfNeighbors_1 |
| 6 | 0.198611 | Nuclei_Neighbors_PercentTouching_1 |
| 7 | 0.164703 | Cells_Intensity_StdIntensityEdge_Hoechst |
| 8 | 0.152553 | Cells_Texture_InfoMeas1_Ph_golgi_5 |
| 9 | 0.140802 | Cytoplasm_Texture_AngularSecondMoment_ER_5 |
| 10 | 0.135892 | Cytoplasm_Texture_AngularSecondMoment_ER_3 |
| 11 | 0.127007 | Cytoplasm_AreaShape_Area |
| 12 | 0.124321 | Cells_RadialDistribution_RadialCV_Syto_2of4 |
| 13 | 0.122387 | Cells_Texture_AngularSecondMoment_Hoechst_5 |
| 14 | 0.118047 | Cells_Texture_InfoMeas1_Ph_golgi_3 |
| 15 | 0.117559 | Cells_Intensity_UpperQuartileIntensity_Hoechst |
| 16 | 0.114758 | Cells_RadialDistribution_RadialCV_Ph_golgi_2of4 |
| 17 | 0.108567 | Cells_Texture_AngularSecondMoment_Syto_5 |
| 18 | 0.106557 | Cells_Texture_AngularSecondMoment_Hoechst_3 |
| 19 | 0.105696 | Cytoplasm_Intensity_IntegratedIntensity_ER |
| 20 | 0.105416 | Cells_Texture_AngularSecondMoment_Syto_3 |
| 21 | 0.104250 | Cytoplasm_AreaShape_FormFactor |
| 22 | 0.103937 | Cells_Texture_AngularSecondMoment_ER_5 |
| 23 | 0.102670 | Cytoplasm_Texture_Contrast_Hoechst_5 |
| 24 | 0.101353 | Cytoplasm_Texture_Variance_Hoechst_3 |
| 25 | 0.101331 | Cells_Texture_AngularSecondMoment_ER_3 |
| 26 | 0.100744 | Cells_RadialDistribution_RadialCV_Syto_3of4 |
| 27 | 0.100661 | Cytoplasm_Intensity_IntegratedIntensity_Hoechst |
| 28 | 0.100642 | Cytoplasm_Texture_Variance_Hoechst_5 |
| 29 | 0.100614 | Cytoplasm_Texture_SumVariance_Hoechst_3 |
| 30 | 0.100143 | Cells_Texture_Correlation_Ph_golgi_5 |
| 31 | 0.099512 | Cytoplasm_Texture_Contrast_Hoechst_3 |
| 32 | 0.098750 | Cytoplasm_Texture_SumVariance_Hoechst_5 |
| 33 | 0.095751 | Cells_AreaShape_Area |
| 34 | 0.095562 | Cells_Texture_SumVariance_Ph_golgi_5 |
| 35 | 0.095120 | Cytoplasm_Intensity_IntegratedIntensity_Mito |
| 36 | 0.094600 | Cells_Intensity_MaxIntensityEdge_Hoechst |
| 37 | 0.093537 | Cells_RadialDistribution_RadialCV_Ph_golgi_3of4 |
| 38 | 0.092028 | Cytoplasm_Intensity_IntegratedIntensity_Syto |
| 39 | 0.090770 | Nuclei_Intensity_StdIntensity_Syto |
| 40 | 0.089660 | Cells_Texture_Gabor_Syto_5 |
| 41 | 0.089286 | Cytoplasm_Intensity_IntegratedIntensity_Ph_golgi |
| 42 | 0.088746 | Cytoplasm_Texture_AngularSecondMoment_Mito_5 |
| 43 | 0.087682 | Cytoplasm_Texture_AngularSecondMoment_Mito_3 |
| 44 | 0.086860 | Cells_Intensity_StdIntensityEdge_Mito |
| 45 | 0.086488 | Cells_Texture_Gabor_Syto_3 |
| 46 | 0.085784 | Cytoplasm_Texture_Contrast_ER_3 |
| 47 | 0.085363 | Cells_RadialDistribution_RadialCV_Syto_1of4 |
| 48 | 0.084639 | Cells_Intensity_MassDisplacement_Ph_golgi |
| 49 | 0.084626 | Cytoplasm_Texture_Entropy_Hoechst_3 |
| 50 | 0.084230 | Cells_Texture_SumVariance_Ph_golgi_3 |
| 51 | 0.083466 | Cells_Intensity_IntegratedIntensity_ER |
| 52 | 0.083462 | Cells_RadialDistribution_RadialCV_ER_2of4 |
| 53 | 0.083362 | Cytoplasm_Texture_Entropy_Hoechst_5 |
| 54 | 0.082142 | Cytoplasm_Texture_DifferenceVariance_Hoechst_5 |
| 55 | 0.081740 | Cytoplasm_Texture_DifferenceVariance_Hoechst_3 |
| 56 | 0.080923 | Cytoplasm_Texture_SumEntropy_Hoechst_3 |
| 57 | 0.079957 | Cells_Texture_Contrast_Hoechst_3 |
| 58 | 0.078668 | Cytoplasm_Texture_SumEntropy_Hoechst_5 |
| 59 | 0.078393 | Cells_Intensity_StdIntensity_Syto |
| 60 | 0.078026 | Cytoplasm_Texture_AngularSecondMoment_Syto_3 |
| 61 | 0.077999 | Cytoplasm_AreaShape_Zernike_2_0 |
| 62 | 0.077975 | Cytoplasm_Texture_Contrast_ER_5 |
| 63 | 0.077898 | Cells_Texture_Contrast_Hoechst_5 |
| 64 | 0.077635 | Cells_RadialDistribution_RadialCV_ER_1of4 |
| 65 | 0.077184 | Nuclei_Intensity_StdIntensityEdge_ER |
| 66 | 0.076491 | Cytoplasm_Texture_AngularSecondMoment_Syto_5 |
| 67 | 0.074515 | Cytoplasm_Texture_DifferenceEntropy_Hoechst_3 |
| 68 | 0.074273 | Cells_Intensity_MassDisplacement_Syto |
| 69 | 0.074072 | Cytoplasm_Texture_DifferenceEntropy_Hoechst_5 |
| 70 | 0.073954 | Cells_RadialDistribution_RadialCV_ER_3of4 |
| 71 | 0.072866 | Cytoplasm_Texture_AngularSecondMoment_Ph_golgi_3 |
| 72 | 0.072646 | Cytoplasm_Texture_AngularSecondMoment_Ph_golgi_5 |
| 73 | 0.072640 | Cytoplasm_Intensity_StdIntensityEdge_ER |
| 74 | 0.071903 | Nuclei_Intensity_StdIntensity_ER |
| 75 | 0.071783 | Cells_RadialDistribution_RadialCV_Mito_1of4 |
| 76 | 0.071165 | Cells_Intensity_MassDisplacement_ER |
| 77 | 0.070669 | Cells_Texture_InfoMeas2_Ph_golgi_5 |
| 78 | 0.070340 | Cells_Intensity_IntegratedIntensity_Syto |
| 79 | 0.070299 | Cytoplasm_Intensity_StdIntensityEdge_Syto |
| 80 | 0.069953 | Cells_Texture_Contrast_Syto_3 |
| 81 | 0.069813 | Cells_Intensity_IntegratedIntensity_Mito |
| 82 | 0.068621 | Cells_Texture_Gabor_Mito_5 |
| 83 | 0.067924 | Cells_Intensity_IntegratedIntensity_Ph_golgi |
| 84 | 0.067436 | Cells_RadialDistribution_RadialCV_Mito_2of4 |
| 85 | 0.067064 | Cells_Intensity_StdIntensity_ER |
| 86 | 0.067019 | Cytoplasm_Texture_Contrast_Ph_golgi_3 |
| 87 | 0.066782 | Cells_Texture_Contrast_ER_3 |
| 88 | 0.066204 | Cytoplasm_Intensity_MassDisplacement_Hoechst |
| 89 | 0.066163 | Cytoplasm_Texture_Contrast_Ph_golgi_5 |
| 90 | 0.065856 | Cytoplasm_Texture_SumVariance_ER_3 |
| 91 | 0.065833 | Cytoplasm_Texture_Variance_ER_3 |
| 92 | 0.065686 | Cytoplasm_Texture_SumVariance_ER_5 |
| 93 | 0.065502 | Cells_Texture_Entropy_Hoechst_3 |
| 94 | 0.064962 | Cells_Texture_Gabor_Hoechst_3 |
| 95 | 0.064582 | Cytoplasm_Texture_Variance_ER_5 |
| 96 | 0.064530 | Cells_Intensity_MaxIntensity_ER |
| 97 | 0.064497 | Cytoplasm_Texture_Contrast_Mito_3 |
| 98 | 0.064450 | Cells_RadialDistribution_RadialCV_Ph_golgi_1of4 |
| 99 | 0.064359 | Cytoplasm_Intensity_MaxIntensity_ER |
| 100 | 0.064297 | Cells_Intensity_StdIntensityEdge_Syto |
| 101 | 0.063886 | Cytoplasm_Texture_Contrast_Syto_3 |
| 102 | 0.063850 | Cells_Texture_Entropy_Hoechst_5 |
| 103 | 0.063186 | Cells_Intensity_StdIntensityEdge_ER |
| 104 | 0.063098 | Cells_Texture_Variance_Ph_golgi_5 |
| 105 | 0.062955 | Cells_Texture_Gabor_Ph_golgi_5 |
| 106 | 0.062772 | Cytoplasm_Texture_AngularSecondMoment_Hoechst_5 |
| 107 | 0.062152 | Cells_Texture_InfoMeas1_ER_5 |
| 108 | 0.062094 | Cells_Intensity_StdIntensity_Ph_golgi |
| 109 | 0.061576 | Cells_Texture_SumEntropy_Hoechst_3 |
| 110 | 0.061205 | Cells_Texture_Correlation_Ph_golgi_3 |
| 111 | 0.060998 | Cells_Texture_Gabor_ER_3 |
| 112 | 0.060756 | Cells_Texture_Gabor_ER_5 |
| 113 | 0.060489 | Cells_Texture_Variance_Ph_golgi_3 |
| 114 | 0.059934 | Cytoplasm_Intensity_StdIntensity_Hoechst |
| 115 | 0.059713 | Nuclei_Intensity_MaxIntensity_ER |
| 116 | 0.059309 | Nuclei_Intensity_MaxIntensityEdge_ER |
| 117 | 0.059109 | Cells_Texture_Contrast_ER_5 |
| 118 | 0.058901 | Cells_AreaShape_FormFactor |
| 119 | 0.058582 | Cells_Texture_SumEntropy_Hoechst_5 |
| 120 | 0.058343 | Cells_Texture_Gabor_Mito_3 |
| 121 | 0.058341 | Cytoplasm_Intensity_MinIntensity_Ph_golgi |
| 122 | 0.058306 | Cells_Intensity_MinIntensity_Ph_golgi |
| 123 | 0.058273 | Nuclei_Intensity_MaxIntensity_Syto |
| 124 | 0.058006 | Cells_Texture_DifferenceVariance_Hoechst_3 |
| 125 | 0.057858 | Cells_Texture_Gabor_Ph_golgi_3 |
| 126 | 0.057787 | Cytoplasm_Texture_AngularSecondMoment_Hoechst_3 |
| 127 | 0.057284 | Cells_Texture_InfoMeas1_Syto_5 |
| 128 | 0.057139 | Cells_Intensity_MinIntensityEdge_Ph_golgi |
| 129 | 0.056879 | Cells_Texture_SumAverage_Hoechst_5 |
| 130 | 0.056555 | Cells_Intensity_MaxIntensityEdge_Mito |
| 131 | 0.056351 | Cells_Texture_AngularSecondMoment_Mito_5 |
| 132 | 0.056332 | Cells_Texture_Gabor_Hoechst_5 |
| 133 | 0.056091 | Cytoplasm_Intensity_StdIntensityEdge_Ph_golgi |
| 134 | 0.055946 | Cytoplasm_Texture_Contrast_Mito_5 |
| 135 | 0.055905 | Cytoplasm_Intensity_MinIntensityEdge_Ph_golgi |
| 136 | 0.055843 | Cytoplasm_Texture_InfoMeas1_Ph_golgi_3 |
| 137 | 0.055716 | Cells_Texture_SumVariance_Mito_5 |
| 138 | 0.055644 | Cells_Texture_SumAverage_Hoechst_3 |
| 139 | 0.055553 | Nuclei_Texture_SumVariance_Mito_3 |
| 140 | 0.055474 | Cells_Texture_AngularSecondMoment_Mito_3 |
| 141 | 0.055451 | Cytoplasm_Texture_DifferenceVariance_ER_3 |
| 142 | 0.055435 | Cells_Texture_Contrast_Syto_5 |
| 143 | 0.055211 | Nuclei_Intensity_StdIntensity_Ph_golgi |
| 144 | 0.055138 | Nuclei_Texture_SumVariance_Mito_5 |
| 145 | 0.055107 | Cells_Intensity_MaxIntensity_Syto |
| 146 | 0.054807 | Nuclei_Intensity_StdIntensityEdge_Ph_golgi |
| 147 | 0.054784 | Cytoplasm_Texture_Entropy_ER_3 |
| 148 | 0.054522 | Nuclei_Texture_AngularSecondMoment_ER_5 |
| 149 | 0.054503 | Cells_Intensity_MedianIntensity_Syto |
| 150 | 0.054285 | Cytoplasm_Texture_Contrast_Syto_5 |
| 151 | 0.053984 | Nuclei_AreaShape_Zernike_3_3 |
| 152 | 0.053805 | Cytoplasm_Texture_SumAverage_ER_5 |
| 153 | 0.053472 | Cytoplasm_Texture_InfoMeas1_Ph_golgi_5 |
| 154 | 0.053360 | Cytoplasm_Texture_SumAverage_ER_3 |
| 155 | 0.053336 | Cells_Intensity_MedianIntensity_Hoechst |
| 156 | 0.053268 | Nuclei_Texture_AngularSecondMoment_Mito_5 |
| 157 | 0.053029 | Cells_Texture_DifferenceEntropy_Hoechst_3 |
| 158 | 0.052577 | Nuclei_Texture_AngularSecondMoment_ER_3 |
| 159 | 0.052492 | Cytoplasm_Intensity_MinIntensity_Syto |
| 160 | 0.052488 | Cells_Intensity_MinIntensity_Syto |
| 161 | 0.052433 | Cytoplasm_Intensity_StdIntensity_ER |
| 162 | 0.052429 | Cytoplasm_Intensity_MaxIntensityEdge_ER |
| 163 | 0.052278 | Nuclei_Texture_SumAverage_Ph_golgi_5 |
| 164 | 0.052241 | Cells_Intensity_MeanIntensityEdge_Syto |
| 165 | 0.052213 | Cells_AreaShape_MajorAxisLength |
| 166 | 0.052090 | Cytoplasm_Intensity_StdIntensity_Ph_golgi |
| 167 | 0.052063 | Cells_Texture_Variance_Hoechst_3 |
| 168 | 0.052045 | Cytoplasm_Texture_Entropy_ER_5 |
| 169 | 0.051992 | Cytoplasm_Texture_DifferenceVariance_Ph_golgi_5 |
| 170 | 0.051931 | Cells_RadialDistribution_RadialCV_Mito_3of4 |
| 171 | 0.051861 | Cells_Texture_InfoMeas1_ER_3 |
| 172 | 0.051648 | Cells_Intensity_LowerQuartileIntensity_Syto |
| 173 | 0.051374 | Cells_Texture_DifferenceEntropy_Hoechst_5 |
| 174 | 0.051298 | Cells_Texture_SumVariance_Mito_3 |
| 175 | 0.051256 | Cells_Intensity_MinIntensityEdge_Syto |
| 176 | 0.051222 | Cytoplasm_Intensity_MinIntensityEdge_Syto |
| 177 | 0.051172 | Cells_Intensity_MaxIntensityEdge_Syto |
| 178 | 0.051144 | Cytoplasm_Texture_DifferenceVariance_Ph_golgi_3 |
| 179 | 0.051057 | Nuclei_Texture_SumAverage_Ph_golgi_3 |
| 180 | 0.051013 | Nuclei_Texture_AngularSecondMoment_Mito_3 |
| 181 | 0.050910 | Nuclei_Intensity_StdIntensityEdge_Syto |
| 182 | 0.050899 | Cells_Texture_DifferenceVariance_Hoechst_5 |
| 183 | 0.050789 | Cells_Texture_SumVariance_Hoechst_3 |
| 184 | 0.050445 | Cells_Texture_Contrast_Ph_golgi_3 |
| 185 | 0.050255 | Cells_Texture_Variance_Hoechst_5 |
| 186 | 0.050145 | Cells_Texture_InfoMeas1_Syto_3 |
| 187 | 0.050053 | Cells_Texture_InfoMeas2_Ph_golgi_3 |
| 188 | 0.049942 | Cytoplasm_Intensity_StdIntensity_Syto |
| 189 | 0.049785 | Nuclei_AreaShape_Zernike_5_5 |
| 190 | 0.049401 | Cytoplasm_Texture_DifferenceVariance_Mito_3 |
| 191 | 0.049209 | Cytoplasm_Texture_SumEntropy_ER_3 |
| 192 | 0.049208 | Cells_Texture_InfoMeas1_Mito_5 |
| 193 | 0.049131 | Cells_Intensity_StdIntensityEdge_Ph_golgi |
| 194 | 0.048647 | Cytoplasm_Intensity_MedianIntensity_Syto |
| 195 | 0.048579 | Cells_Intensity_MeanIntensity_Hoechst |
| 196 | 0.048516 | Cells_Intensity_MeanIntensityEdge_Mito |
| 197 | 0.048410 | Cytoplasm_Texture_InfoMeas2_Hoechst_5 |
| 198 | 0.048118 | Cytoplasm_Intensity_MassDisplacement_Ph_golgi |
| 199 | 0.047955 | Cytoplasm_Intensity_LowerQuartileIntensity_Syto |
| 200 | 0.047933 | Cells_Intensity_MaxIntensityEdge_ER |
| 201 | 0.047859 | Cells_Intensity_MeanIntensityEdge_ER |
| 202 | 0.047668 | Cells_Texture_DifferenceVariance_Syto_3 |
| 203 | 0.047414 | Cytoplasm_Intensity_UpperQuartileIntensity_Syto |
| 204 | 0.047220 | Cells_Texture_SumVariance_Hoechst_5 |
| 205 | 0.046877 | Cells_Intensity_MaxIntensityEdge_Ph_golgi |
| 206 | 0.046751 | Cells_Intensity_MeanIntensityEdge_Ph_golgi |
| 207 | 0.046728 | Nuclei_Intensity_MassDisplacement_ER |
| 208 | 0.046705 | Cytoplasm_Intensity_MassDisplacement_ER |
| 209 | 0.046541 | Nuclei_Intensity_UpperQuartileIntensity_Syto |
| 210 | 0.046277 | Cells_Intensity_MassDisplacement_Mito |
| 211 | 0.046226 | Cytoplasm_Texture_SumEntropy_ER_5 |
| 212 | 0.046031 | Nuclei_Intensity_StdIntensity_Mito |
| 213 | 0.046022 | Cytoplasm_Intensity_MassDisplacement_Syto |
| 214 | 0.045236 | Cytoplasm_Intensity_MeanIntensity_Syto |
| 215 | 0.045088 | Nuclei_Intensity_StdIntensityEdge_Mito |
| 216 | 0.044584 | Cytoplasm_AreaShape_MajorAxisLength |
| 217 | 0.044512 | Cytoplasm_Texture_Gabor_Hoechst_5 |
| 218 | 0.044463 | Cells_Intensity_MeanIntensity_Syto |
| 219 | 0.044427 | Cells_Intensity_LowerQuartileIntensity_Ph_golgi |
| 220 | 0.044375 | Cytoplasm_Intensity_UpperQuartileIntensity_Ph_golgi |
| 221 | 0.044318 | Cytoplasm_Intensity_UpperQuartileIntensity_Mito |
| 222 | 0.044311 | Cytoplasm_Intensity_MeanIntensity_Ph_golgi |
| 223 | 0.044251 | Cells_Texture_Variance_Mito_5 |
| 224 | 0.044170 | Nuclei_Intensity_MeanIntensity_Syto |
| 225 | 0.044149 | Cells_Texture_DifferenceVariance_ER_3 |
| 226 | 0.044139 | Cytoplasm_Intensity_LowerQuartileIntensity_Ph_golgi |
| 227 | 0.043932 | Cytoplasm_Texture_SumVariance_Mito_3 |
| 228 | 0.043791 | Cytoplasm_Intensity_MedianIntensity_Ph_golgi |
| 229 | 0.043753 | Nuclei_Intensity_IntegratedIntensity_Syto |
| 230 | 0.043549 | Cells_Intensity_UpperQuartileIntensity_Syto |
| 231 | 0.043519 | Cells_Texture_Contrast_Mito_3 |
| 232 | 0.043404 | Cells_Texture_InfoMeas1_Mito_3 |
| 233 | 0.043355 | Cytoplasm_Texture_DifferenceVariance_ER_5 |
| 234 | 0.043325 | Cytoplasm_Texture_SumVariance_Ph_golgi_3 |
| 235 | 0.043270 | Nuclei_Intensity_MedianIntensity_Syto |
| 236 | 0.043195 | Nuclei_Texture_InfoMeas1_Mito_5 |
| 237 | 0.043106 | Cells_Intensity_IntegratedIntensity_Hoechst |
| 238 | 0.043090 | Cells_Texture_Variance_Mito_3 |
| 239 | 0.042961 | Cytoplasm_Texture_SumVariance_Mito_5 |
| 240 | 0.042782 | Cells_Intensity_LowerQuartileIntensity_Mito |
| 241 | 0.042697 | Cytoplasm_Intensity_MedianIntensity_Mito |
| 242 | 0.042674 | Cytoplasm_Texture_DifferenceEntropy_ER_3 |
| 243 | 0.042531 | Cells_Texture_Contrast_Ph_golgi_5 |
| 244 | 0.042460 | Cells_RadialDistribution_FracAtD_ER_1of4 |
| 245 | 0.042427 | Cytoplasm_Texture_Variance_Ph_golgi_3 |
| 246 | 0.042387 | Nuclei_AreaShape_Zernike_5_3 |
| 247 | 0.042316 | Cells_Intensity_LowerQuartileIntensity_ER |
| 248 | 0.042246 | Cytoplasm_Intensity_MedianIntensity_ER |
| 249 | 0.042158 | Nuclei_Texture_InfoMeas1_Ph_golgi_3 |
| 250 | 0.042035 | Cytoplasm_Texture_Correlation_Ph_golgi_5 |
| 251 | 0.041995 | Cytoplasm_Texture_Variance_Mito_3 |
| 252 | 0.041903 | Cytoplasm_Intensity_MeanIntensityEdge_Syto |
| 253 | 0.041896 | Nuclei_Texture_Gabor_Syto_3 |
| 254 | 0.041881 | Nuclei_AreaShape_Zernike_7_7 |
| 255 | 0.041867 | Cytoplasm_Texture_Variance_Ph_golgi_5 |
| 256 | 0.041835 | Cells_Intensity_MedianIntensity_Ph_golgi |
| 257 | 0.041565 | Cells_Intensity_MedianIntensity_Mito |
| 258 | 0.041245 | Cytoplasm_Texture_Gabor_Hoechst_3 |
| 259 | 0.040895 | Cytoplasm_Texture_SumVariance_Ph_golgi_5 |
| 260 | 0.040846 | Cytoplasm_Texture_InfoMeas2_Hoechst_3 |
| 261 | 0.040676 | Cytoplasm_Intensity_MeanIntensityEdge_Ph_golgi |
| 262 | 0.040352 | Cytoplasm_Intensity_MaxIntensityEdge_Ph_golgi |
| 263 | 0.040311 | Cells_AreaShape_MinorAxisLength |
| 264 | 0.040304 | Cells_Texture_SumAverage_ER_3 |
| 265 | 0.040229 | Cytoplasm_Texture_Variance_Mito_5 |
| 266 | 0.040197 | Cytoplasm_Texture_DifferenceVariance_Syto_3 |
| 267 | 0.040079 | Cytoplasm_Intensity_LowerQuartileIntensity_Mito |
| 268 | 0.039975 | Cytoplasm_Texture_InfoMeas1_Syto_3 |
| 269 | 0.039923 | Cells_Intensity_MeanIntensity_Ph_golgi |
| 270 | 0.039879 | Cytoplasm_Intensity_MaxIntensity_Ph_golgi |
| 271 | 0.039841 | Cells_Texture_SumVariance_ER_3 |
| 272 | 0.039816 | Nuclei_AreaShape_Zernike_1_1 |
| 273 | 0.039750 | Nuclei_Texture_InfoMeas1_Mito_3 |
| 274 | 0.039749 | Cytoplasm_Intensity_MeanIntensity_Mito |
| 275 | 0.039701 | Cells_Intensity_StdIntensity_Mito |
| 276 | 0.039677 | Cells_Intensity_MaxIntensity_Ph_golgi |
| 277 | 0.039676 | Cells_Texture_SumAverage_ER_5 |
| 278 | 0.039675 | Cytoplasm_Intensity_StdIntensityEdge_Mito |
| 279 | 0.039656 | Nuclei_Texture_Variance_Mito_3 |
| 280 | 0.039651 | Cytoplasm_Intensity_MinIntensity_ER |
| 281 | 0.039637 | Cells_Intensity_MinIntensity_ER |
| 282 | 0.039526 | Cells_Texture_DifferenceVariance_Ph_golgi_3 |
| 283 | 0.039303 | Cytoplasm_Intensity_MinIntensity_Mito |
| 284 | 0.039300 | Cells_Texture_AngularSecondMoment_Ph_golgi_5 |
| 285 | 0.039300 | Cells_Intensity_MinIntensity_Mito |
| 286 | 0.039251 | Nuclei_Texture_Correlation_Mito_5 |
| 287 | 0.039233 | Nuclei_Intensity_LowerQuartileIntensity_Syto |
| 288 | 0.039207 | Cytoplasm_Intensity_LowerQuartileIntensity_ER |
| 289 | 0.039192 | Nuclei_AreaShape_Zernike_9_9 |
| 290 | 0.039012 | Cells_Texture_SumVariance_ER_5 |
| 291 | 0.038888 | Cells_Texture_Variance_ER_3 |
| 292 | 0.038697 | Cells_Intensity_MinIntensityEdge_Mito |
| 293 | 0.038672 | Cytoplasm_Intensity_MinIntensityEdge_Mito |
| 294 | 0.038637 | Nuclei_Intensity_MeanIntensityEdge_ER |
| 295 | 0.038599 | Cytoplasm_Texture_DifferenceVariance_Mito_5 |
| 296 | 0.038518 | Cells_Intensity_MinIntensityEdge_ER |
| 297 | 0.038508 | Cytoplasm_AreaShape_Solidity |
| 298 | 0.038491 | Cytoplasm_Texture_Gabor_Mito_5 |
| 299 | 0.038490 | Cytoplasm_Intensity_MinIntensityEdge_ER |
| 300 | 0.038379 | Nuclei_Intensity_MaxIntensityEdge_Ph_golgi |
| 301 | 0.038376 | Cytoplasm_AreaShape_Zernike_8_0 |
| 302 | 0.038273 | Cytoplasm_Intensity_MeanIntensityEdge_Mito |
| 303 | 0.038106 | Nuclei_Intensity_MaxIntensity_Ph_golgi |
| 304 | 0.038004 | Nuclei_Texture_InfoMeas1_ER_5 |
| 305 | 0.037939 | Cells_Texture_DifferenceVariance_Mito_3 |
| 306 | 0.037901 | Nuclei_Texture_Variance_Mito_5 |
| 307 | 0.037840 | Cells_Intensity_IntegratedIntensityEdge_Syto |
| 308 | 0.037816 | Nuclei_AreaShape_Zernike_4_2 |
| 309 | 0.037736 | Cells_Neighbors_PercentTouching_5 |
| 310 | 0.037607 | Cells_Texture_Variance_ER_5 |
| 311 | 0.037339 | Cytoplasm_Intensity_UpperQuartileIntensity_ER |
| 312 | 0.037232 | Cytoplasm_Texture_Gabor_ER_5 |
| 313 | 0.037068 | Cells_Texture_AngularSecondMoment_Ph_golgi_3 |
| 314 | 0.037050 | Nuclei_Intensity_MaxIntensityEdge_Syto |
| 315 | 0.037008 | Cytoplasm_Intensity_MaxIntensityEdge_Syto |
| 316 | 0.036940 | Cells_Intensity_UpperQuartileIntensity_Ph_golgi |
| 317 | 0.036912 | Nuclei_Texture_Gabor_ER_5 |
| 318 | 0.036847 | Cells_Texture_SumAverage_Syto_3 |
| 319 | 0.036807 | Cells_RadialDistribution_MeanFrac_ER_1of4 |
| 320 | 0.036767 | Cells_Texture_SumAverage_Syto_5 |
| 321 | 0.036642 | Cytoplasm_Texture_DifferenceEntropy_ER_5 |
| 322 | 0.036624 | Cytoplasm_Texture_InfoMeas1_Syto_5 |
| 323 | 0.036549 | Nuclei_Intensity_MassDisplacement_Hoechst |
| 324 | 0.036404 | Nuclei_Texture_Gabor_ER_3 |
| 325 | 0.036319 | Nuclei_Texture_Gabor_Syto_5 |
| 326 | 0.036301 | Cells_Intensity_MeanIntensity_Mito |
| 327 | 0.036197 | Cytoplasm_Texture_Correlation_Hoechst_5 |
| 328 | 0.036174 | Nuclei_Intensity_UpperQuartileIntensity_ER |
| 329 | 0.036087 | Nuclei_AreaShape_Zernike_5_1 |
| 330 | 0.036029 | Cytoplasm_Texture_SumAverage_Mito_5 |
| 331 | 0.035998 | Nuclei_Texture_InfoMeas1_ER_3 |
| 332 | 0.035956 | Nuclei_Texture_InfoMeas1_Ph_golgi_5 |
| 333 | 0.035893 | Cells_Intensity_MedianIntensity_ER |
| 334 | 0.035844 | Cells_Texture_InfoMeas1_Hoechst_5 |
| 335 | 0.035782 | Cytoplasm_Texture_SumAverage_Mito_3 |
| 336 | 0.035702 | Cells_Intensity_IntegratedIntensityEdge_Ph_golgi |
| 337 | 0.035672 | Nuclei_AreaShape_Zernike_7_5 |
| 338 | 0.035624 | Cytoplasm_Texture_InfoMeas1_Hoechst_3 |
| 339 | 0.035525 | Nuclei_Intensity_MeanIntensity_ER |
| 340 | 0.035493 | Cytoplasm_AreaShape_MinorAxisLength |
| 341 | 0.035485 | Cytoplasm_Intensity_MaxIntensity_Syto |
| 342 | 0.035460 | Cytoplasm_Intensity_StdIntensity_Mito |
| 343 | 0.035414 | Cytoplasm_AreaShape_Extent |
| 344 | 0.035414 | Cells_Texture_DifferenceVariance_ER_5 |
| 345 | 0.035185 | Cells_Texture_Entropy_ER_3 |
| 346 | 0.035125 | Cells_Texture_SumVariance_Syto_5 |
| 347 | 0.035107 | Nuclei_Intensity_IntegratedIntensityEdge_Syto |
| 348 | 0.035049 | Nuclei_Texture_DifferenceVariance_Syto_5 |
| 349 | 0.034935 | Nuclei_Texture_Correlation_Ph_golgi_5 |
| 350 | 0.034887 | Cells_Intensity_IntegratedIntensityEdge_Mito |
| 351 | 0.034838 | Nuclei_Intensity_MeanIntensityEdge_Syto |
| 352 | 0.034812 | Cytoplasm_Texture_Gabor_Mito_3 |
| 353 | 0.034658 | Nuclei_Texture_SumVariance_ER_5 |
| 354 | 0.034639 | Nuclei_Intensity_IntegratedIntensityEdge_ER |
| 355 | 0.034539 | Cells_RadialDistribution_FracAtD_ER_2of4 |
| 356 | 0.034421 | Cytoplasm_Intensity_UpperQuartileIntensity_Hoechst |
| 357 | 0.034376 | Nuclei_Texture_SumVariance_ER_3 |
| 358 | 0.034308 | Nuclei_Texture_Correlation_ER_5 |
| 359 | 0.034139 | Cells_Intensity_IntegratedIntensityEdge_ER |
| 360 | 0.034071 | Cells_RadialDistribution_RadialCV_Syto_4of4 |
| 361 | 0.033995 | Cells_Texture_Contrast_Mito_5 |
| 362 | 0.033856 | Cells_Texture_DifferenceVariance_Ph_golgi_5 |
| 363 | 0.033833 | Nuclei_Texture_SumVariance_Ph_golgi_3 |
| 364 | 0.033799 | Nuclei_Intensity_MedianIntensity_ER |
| 365 | 0.033709 | Nuclei_Intensity_UpperQuartileIntensity_Ph_golgi |
| 366 | 0.033602 | Cytoplasm_Texture_Gabor_ER_3 |
| 367 | 0.033587 | Cytoplasm_Texture_InfoMeas1_Hoechst_5 |
| 368 | 0.033583 | Nuclei_Texture_AngularSecondMoment_Ph_golgi_5 |
| 369 | 0.033563 | Nuclei_Intensity_IntegratedIntensity_Ph_golgi |
| 370 | 0.033532 | Cells_Texture_DifferenceEntropy_ER_3 |
| 371 | 0.033381 | Cytoplasm_Texture_InfoMeas1_Mito_3 |
| 372 | 0.033357 | Cells_Intensity_UpperQuartileIntensity_Mito |
| 373 | 0.033246 | Cells_RadialDistribution_FracAtD_Syto_1of4 |
| 374 | 0.033243 | Nuclei_Intensity_IntegratedIntensityEdge_Ph_golgi |
| 375 | 0.033064 | Nuclei_Texture_DifferenceVariance_Syto_3 |
| 376 | 0.032857 | Cells_Texture_DifferenceVariance_Syto_5 |
| 377 | 0.032713 | Nuclei_Intensity_MedianIntensity_Ph_golgi |
| 378 | 0.032512 | Nuclei_Texture_Contrast_Syto_5 |
| 379 | 0.032499 | Nuclei_Texture_SumAverage_Mito_5 |
| 380 | 0.032479 | Nuclei_Intensity_MassDisplacement_Ph_golgi |
| 381 | 0.032343 | Nuclei_Intensity_LowerQuartileIntensity_ER |
| 382 | 0.032207 | Nuclei_Texture_AngularSecondMoment_Ph_golgi_3 |
| 383 | 0.032178 | Nuclei_Intensity_MinIntensityEdge_Syto |
| 384 | 0.032177 | Nuclei_Intensity_MinIntensity_Syto |
| 385 | 0.032171 | Cells_Texture_Correlation_Mito_5 |
| 386 | 0.032144 | Nuclei_Texture_SumAverage_Mito_3 |
| 387 | 0.032140 | Nuclei_Intensity_MeanIntensity_Ph_golgi |
| 388 | 0.031982 | Nuclei_Intensity_MaxIntensity_Mito |
| 389 | 0.031970 | Nuclei_Texture_Gabor_Hoechst_3 |
| 390 | 0.031954 | Cytoplasm_Intensity_MeanIntensity_ER |
| 391 | 0.031836 | Cells_Texture_SumVariance_Syto_3 |
| 392 | 0.031805 | Cytoplasm_Intensity_IntegratedIntensityEdge_Syto |
| 393 | 0.031762 | Cells_Texture_Entropy_ER_5 |
| 394 | 0.031595 | Nuclei_Texture_Gabor_Mito_5 |
| 395 | 0.031594 | Nuclei_Texture_Correlation_Ph_golgi_3 |
| 396 | 0.031591 | Cells_RadialDistribution_FracAtD_Ph_golgi_1of4 |
| 397 | 0.031562 | Cytoplasm_Intensity_IntegratedIntensityEdge_Ph_golgi |
| 398 | 0.031537 | Nuclei_Intensity_MaxIntensityEdge_Mito |
| 399 | 0.031427 | Cells_RadialDistribution_RadialCV_Ph_golgi_4of4 |
| 400 | 0.031414 | Nuclei_Intensity_UpperQuartileIntensity_Mito |
| 401 | 0.031410 | Cytoplasm_Texture_InfoMeas1_ER_3 |
| 402 | 0.031150 | Cytoplasm_Texture_SumAverage_Hoechst_3 |
| 403 | 0.031047 | Nuclei_Intensity_LowerQuartileIntensity_Ph_golgi |
| 404 | 0.031018 | Cytoplasm_Texture_SumAverage_Hoechst_5 |
| 405 | 0.030863 | Nuclei_Intensity_MeanIntensityEdge_Ph_golgi |
| 406 | 0.030817 | Nuclei_Texture_SumVariance_Ph_golgi_5 |
| 407 | 0.030744 | Nuclei_Intensity_IntegratedIntensity_ER |
| 408 | 0.030593 | Nuclei_Intensity_MassDisplacement_Mito |
| 409 | 0.030437 | Cytoplasm_Texture_DifferenceVariance_Syto_5 |
| 410 | 0.030340 | Cells_RadialDistribution_FracAtD_Mito_1of4 |
| 411 | 0.030287 | Cells_RadialDistribution_RadialCV_Mito_4of4 |
| 412 | 0.030208 | Cytoplasm_Intensity_MaxIntensityEdge_Mito |
| 413 | 0.029666 | Cytoplasm_Texture_SumVariance_Syto_3 |
| 414 | 0.029664 | Cytoplasm_Texture_Variance_Syto_3 |
| 415 | 0.029627 | Nuclei_Texture_Correlation_Mito_3 |
| 416 | 0.029615 | Nuclei_Intensity_MedianIntensity_Mito |
| 417 | 0.029532 | Cells_Texture_SumEntropy_ER_3 |
| 418 | 0.029359 | Cytoplasm_Texture_Correlation_Hoechst_3 |
| 419 | 0.029202 | Nuclei_Intensity_MeanIntensity_Mito |
| 420 | 0.029159 | Nuclei_Intensity_MinIntensityEdge_Ph_golgi |
| 421 | 0.029110 | Nuclei_Intensity_IntegratedIntensity_Mito |
| 422 | 0.029010 | Cells_Texture_DifferenceEntropy_Syto_3 |
| 423 | 0.028974 | Cytoplasm_Texture_SumVariance_Syto_5 |
| 424 | 0.028853 | Cells_Intensity_MaxIntensity_Mito |
| 425 | 0.028679 | Cytoplasm_Intensity_MaxIntensity_Mito |
| 426 | 0.028642 | Cells_Intensity_MeanIntensityEdge_Hoechst |
| 427 | 0.028594 | Nuclei_Intensity_MinIntensity_Ph_golgi |
| 428 | 0.028574 | Cytoplasm_Texture_Gabor_Ph_golgi_5 |
| 429 | 0.028535 | Cells_RadialDistribution_MeanFrac_ER_2of4 |
| 430 | 0.028533 | Cytoplasm_Texture_SumAverage_Syto_3 |
| 431 | 0.028505 | Cytoplasm_Texture_Variance_Syto_5 |
| 432 | 0.028492 | Cytoplasm_Texture_SumAverage_Syto_5 |
| 433 | 0.028481 | Cytoplasm_Intensity_MeanIntensityEdge_ER |
| 434 | 0.028415 | Nuclei_Texture_Correlation_Syto_5 |
| 435 | 0.028334 | Cytoplasm_Texture_InfoMeas1_Mito_5 |
| 436 | 0.027933 | Nuclei_Texture_SumVariance_Syto_3 |
| 437 | 0.027827 | Nuclei_Texture_AngularSecondMoment_Syto_5 |
| 438 | 0.027786 | Cells_Texture_InverseDifferenceMoment_Hoechst_5 |
| 439 | 0.027777 | Cells_Texture_SumAverage_Mito_3 |
| 440 | 0.027760 | Cytoplasm_Intensity_MeanIntensity_Hoechst |
| 441 | 0.027739 | Cells_Intensity_StdIntensity_Hoechst |
| 442 | 0.027733 | Cells_Texture_DifferenceEntropy_ER_5 |
| 443 | 0.027612 | Cells_Texture_DifferenceVariance_Mito_5 |
| 444 | 0.027600 | Cytoplasm_AreaShape_Zernike_0_0 |
| 445 | 0.027553 | Nuclei_AreaShape_Zernike_9_7 |
| 446 | 0.027478 | Cells_Texture_SumAverage_Mito_5 |
| 447 | 0.027461 | Cytoplasm_Texture_Correlation_Ph_golgi_3 |
| 448 | 0.027429 | Nuclei_Intensity_LowerQuartileIntensity_Mito |
| 449 | 0.027153 | Nuclei_Texture_Variance_Ph_golgi_5 |
| 450 | 0.027014 | Cells_Texture_Variance_Syto_5 |
| 451 | 0.026949 | Cells_Texture_Correlation_Syto_5 |
| 452 | 0.026828 | Nuclei_Intensity_MinIntensityEdge_ER |
| 453 | 0.026802 | Nuclei_Intensity_MeanIntensityEdge_Mito |
| 454 | 0.026733 | Cells_RadialDistribution_MeanFrac_Syto_1of4 |
| 455 | 0.026691 | Cytoplasm_Intensity_IntegratedIntensityEdge_Mito |
| 456 | 0.026632 | Nuclei_Intensity_IntegratedIntensityEdge_Mito |
| 457 | 0.026607 | Nuclei_AreaShape_Zernike_4_4 |
| 458 | 0.026472 | Nuclei_Intensity_MinIntensity_ER |
| 459 | 0.026466 | Nuclei_Texture_Variance_Syto_5 |
| 460 | 0.026355 | Nuclei_Texture_Variance_Ph_golgi_3 |
| 461 | 0.026318 | Cells_Intensity_MeanIntensity_ER |
| 462 | 0.026293 | Cytoplasm_Texture_Entropy_Mito_3 |
| 463 | 0.026289 | Cytoplasm_Texture_InverseDifferenceMoment_ER_5 |
| 464 | 0.026287 | Cytoplasm_Texture_Gabor_Ph_golgi_3 |
| 465 | 0.026267 | Nuclei_Texture_InfoMeas2_Mito_5 |
| 466 | 0.026266 | Cytoplasm_AreaShape_Zernike_6_0 |
| 467 | 0.026224 | Nuclei_AreaShape_Zernike_8_0 |
| 468 | 0.026212 | Cytoplasm_AreaShape_Zernike_7_1 |
| 469 | 0.026142 | Cytoplasm_Texture_Gabor_Syto_5 |
| 470 | 0.026055 | Cells_Texture_Variance_Syto_3 |
| 471 | 0.025978 | Cells_RadialDistribution_MeanFrac_Ph_golgi_1of4 |
| 472 | 0.025961 | Nuclei_AreaShape_Zernike_6_6 |
| 473 | 0.025737 | Cells_Texture_SumEntropy_ER_5 |
| 474 | 0.025616 | Cytoplasm_Texture_DifferenceEntropy_Mito_3 |
| 475 | 0.025447 | Cytoplasm_Intensity_MassDisplacement_Mito |
| 476 | 0.025358 | Cells_Texture_Correlation_ER_5 |
| 477 | 0.025160 | Cytoplasm_Texture_InfoMeas1_ER_5 |
| 478 | 0.025137 | Nuclei_Intensity_MassDisplacement_Syto |
| 479 | 0.024951 | Cells_Texture_InfoMeas1_Hoechst_3 |
| 480 | 0.024898 | Nuclei_Texture_SumVariance_Syto_5 |
| 481 | 0.024709 | Cells_RadialDistribution_FracAtD_Mito_2of4 |
| 482 | 0.024661 | Cytoplasm_Texture_DifferenceEntropy_Ph_golgi_3 |
| 483 | 0.024639 | Nuclei_Texture_Variance_ER_3 |
| 484 | 0.024594 | Nuclei_Texture_Variance_Syto_3 |
| 485 | 0.024396 | Nuclei_Texture_AngularSecondMoment_Syto_3 |
| 486 | 0.024246 | Cells_RadialDistribution_FracAtD_ER_4of4 |
| 487 | 0.024210 | Cytoplasm_Texture_DifferenceEntropy_Syto_3 |
| 488 | 0.024045 | Nuclei_Texture_Variance_ER_5 |
| 489 | 0.024035 | Cytoplasm_Texture_Entropy_Mito_5 |
| 490 | 0.023961 | Nuclei_Texture_Correlation_ER_3 |
| 491 | 0.023949 | Cytoplasm_AreaShape_Zernike_2_2 |
| 492 | 0.023900 | Nuclei_Intensity_MinIntensity_Mito |
| 493 | 0.023890 | Cells_AreaShape_Zernike_4_2 |
| 494 | 0.023888 | Cells_Texture_Entropy_Syto_3 |
| 495 | 0.023828 | Cells_RadialDistribution_FracAtD_Syto_2of4 |
| 496 | 0.023794 | Nuclei_Intensity_StdIntensityEdge_Hoechst |
| 497 | 0.023749 | Nuclei_Intensity_MinIntensityEdge_Mito |
| 498 | 0.023570 | Nuclei_Texture_Contrast_ER_5 |
| 499 | 0.023518 | Cells_RadialDistribution_MeanFrac_Mito_1of4 |
| 500 | 0.023518 | Nuclei_Texture_Contrast_ER_3 |
| 501 | 0.023274 | Nuclei_Texture_Contrast_Syto_3 |
| 502 | 0.023117 | Nuclei_Texture_InfoMeas1_Syto_3 |
| 503 | 0.023106 | Cytoplasm_AreaShape_Zernike_4_0 |
| 504 | 0.023089 | Cells_Intensity_UpperQuartileIntensity_ER |
| 505 | 0.023062 | Nuclei_AreaShape_Zernike_8_4 |
| 506 | 0.023060 | Cells_RadialDistribution_FracAtD_Ph_golgi_2of4 |
| 507 | 0.023028 | Cytoplasm_Texture_DifferenceEntropy_Ph_golgi_5 |
| 508 | 0.022734 | Nuclei_Texture_InfoMeas2_Mito_3 |
| 509 | 0.022725 | Cytoplasm_Texture_Entropy_Syto_3 |
| 510 | 0.022525 | Cytoplasm_Texture_Gabor_Syto_3 |
| 511 | 0.022399 | Cytoplasm_Texture_SumEntropy_Mito_3 |
| 512 | 0.022322 | Nuclei_Texture_Gabor_Ph_golgi_3 |
| 513 | 0.021997 | Nuclei_Texture_InfoMeas2_ER_5 |
| 514 | 0.021846 | Nuclei_Texture_Contrast_Ph_golgi_5 |
| 515 | 0.021657 | Nuclei_AreaShape_Zernike_8_2 |
| 516 | 0.021576 | Nuclei_Texture_InfoMeas2_Ph_golgi_3 |
| 517 | 0.021226 | Nuclei_AreaShape_Zernike_3_1 |
| 518 | 0.021077 | Cytoplasm_Intensity_IntegratedIntensityEdge_ER |
| 519 | 0.021020 | Cells_Texture_Correlation_Mito_3 |
| 520 | 0.021008 | Nuclei_Texture_Gabor_Ph_golgi_5 |
| 521 | 0.020929 | Cytoplasm_Texture_InverseDifferenceMoment_Ph_golgi_5 |
| 522 | 0.020896 | Nuclei_AreaShape_Zernike_8_8 |
| 523 | 0.020840 | Cytoplasm_Texture_InverseDifferenceMoment_ER_3 |
| 524 | 0.020818 | Cells_Texture_Entropy_Syto_5 |
| 525 | 0.020764 | Nuclei_AreaShape_Zernike_7_3 |
| 526 | 0.020652 | Cytoplasm_Intensity_MeanIntensityEdge_Hoechst |
| 527 | 0.020632 | Cells_Texture_DifferenceEntropy_Syto_5 |
| 528 | 0.020539 | Cytoplasm_Texture_Entropy_Ph_golgi_3 |
| 529 | 0.020474 | Nuclei_Texture_Contrast_Ph_golgi_3 |
| 530 | 0.020431 | Cytoplasm_Texture_DifferenceEntropy_Mito_5 |
| 531 | 0.020412 | Cytoplasm_Texture_SumEntropy_Mito_5 |
| 532 | 0.020389 | Cytoplasm_Texture_InverseDifferenceMoment_Hoechst_5 |
| 533 | 0.020211 | Cytoplasm_Texture_Entropy_Syto_5 |
| 534 | 0.019958 | Nuclei_Texture_Entropy_ER_5 |
| 535 | 0.019944 | Nuclei_Texture_Gabor_Hoechst_5 |
| 536 | 0.019893 | Cells_Texture_InfoMeas2_Mito_5 |
| 537 | 0.019862 | Cells_RadialDistribution_MeanFrac_ER_4of4 |
| 538 | 0.019807 | Cytoplasm_AreaShape_Zernike_4_2 |
| 539 | 0.019708 | Cytoplasm_Texture_InfoMeas2_Ph_golgi_5 |
| 540 | 0.019701 | Cells_Neighbors_NumberOfNeighbors_5 |
| 541 | 0.019599 | Nuclei_AreaShape_Zernike_4_0 |
| 542 | 0.019492 | Nuclei_Texture_InfoMeas2_Ph_golgi_5 |
| 543 | 0.019436 | Nuclei_Texture_InfoMeas2_ER_3 |
| 544 | 0.019418 | Cytoplasm_Texture_Correlation_Mito_5 |
| 545 | 0.019418 | Cytoplasm_Texture_InfoMeas2_Ph_golgi_3 |
| 546 | 0.019359 | Nuclei_Texture_Entropy_ER_3 |
| 547 | 0.019338 | Nuclei_Texture_Gabor_Mito_3 |
| 548 | 0.019287 | Cytoplasm_Texture_Entropy_Ph_golgi_5 |
| 549 | 0.019262 | Cells_Texture_InverseDifferenceMoment_Syto_5 |
| 550 | 0.019240 | Cells_RadialDistribution_RadialCV_ER_4of4 |
| 551 | 0.019197 | Nuclei_Texture_DifferenceVariance_Mito_3 |
| 552 | 0.019186 | Cells_Texture_InverseDifferenceMoment_ER_5 |
| 553 | 0.019181 | Cells_Texture_InverseDifferenceMoment_Hoechst_3 |
| 554 | 0.019094 | Cells_Texture_DifferenceEntropy_Ph_golgi_3 |
| 555 | 0.018994 | Cytoplasm_AreaShape_Zernike_3_1 |
| 556 | 0.018975 | Cytoplasm_Texture_InverseDifferenceMoment_Ph_golgi_3 |
| 557 | 0.018847 | Nuclei_Texture_Contrast_Mito_5 |
| 558 | 0.018822 | Nuclei_Texture_SumAverage_ER_5 |
| 559 | 0.018815 | Cytoplasm_Texture_DifferenceEntropy_Syto_5 |
| 560 | 0.018630 | Cells_Intensity_LowerQuartileIntensity_Hoechst |
| 561 | 0.018616 | Nuclei_Texture_SumEntropy_Mito_3 |
| 562 | 0.018591 | Nuclei_Texture_Entropy_Mito_5 |
| 563 | 0.018570 | Cells_Texture_Correlation_Syto_3 |
| 564 | 0.018505 | Nuclei_Texture_SumAverage_ER_3 |
| 565 | 0.018498 | Nuclei_Texture_Correlation_Syto_3 |
| 566 | 0.018328 | Cytoplasm_Texture_InverseDifferenceMoment_Syto_5 |
| 567 | 0.018233 | Cells_Texture_SumAverage_Ph_golgi_5 |
| 568 | 0.018083 | Cells_RadialDistribution_MeanFrac_Mito_2of4 |
| 569 | 0.018043 | Nuclei_Texture_SumAverage_Syto_3 |
| 570 | 0.018016 | Nuclei_Texture_SumAverage_Syto_5 |
| 571 | 0.018002 | Cytoplasm_Texture_InverseDifferenceMoment_Mito_5 |
| 572 | 0.017893 | Cytoplasm_Intensity_MedianIntensity_Hoechst |
| 573 | 0.017825 | Nuclei_AreaShape_Zernike_6_0 |
| 574 | 0.017806 | Nuclei_Texture_SumEntropy_Mito_5 |
| 575 | 0.017798 | Cells_RadialDistribution_MeanFrac_Syto_2of4 |
| 576 | 0.017724 | Nuclei_Texture_InfoMeas1_Syto_5 |
| 577 | 0.017707 | Nuclei_Texture_Entropy_Mito_3 |
| 578 | 0.017653 | Cells_RadialDistribution_MeanFrac_Ph_golgi_2of4 |
| 579 | 0.017634 | Cells_Texture_DifferenceEntropy_Mito_3 |
| 580 | 0.017498 | Cells_Texture_SumAverage_Ph_golgi_3 |
| 581 | 0.017464 | Cytoplasm_Intensity_StdIntensityEdge_Hoechst |
| 582 | 0.017436 | Nuclei_AreaShape_Zernike_9_5 |
| 583 | 0.017436 | Nuclei_Texture_DifferenceVariance_Ph_golgi_5 |
| 584 | 0.017336 | Cells_Texture_InfoMeas2_ER_5 |
| 585 | 0.017259 | Cells_Texture_InverseDifferenceMoment_Syto_3 |
| 586 | 0.017216 | Cells_RadialDistribution_FracAtD_Syto_4of4 |
| 587 | 0.017108 | Nuclei_Texture_InfoMeas1_Hoechst_3 |
| 588 | 0.016993 | Cytoplasm_Texture_Correlation_Syto_5 |
| 589 | 0.016869 | Nuclei_Texture_SumEntropy_ER_3 |
| 590 | 0.016787 | Cytoplasm_Texture_InverseDifferenceMoment_Hoechst_3 |
| 591 | 0.016762 | Cells_RadialDistribution_FracAtD_Mito_4of4 |
| 592 | 0.016645 | Cells_Texture_Correlation_ER_3 |
| 593 | 0.016560 | Nuclei_Texture_Correlation_Hoechst_5 |
| 594 | 0.016544 | Cells_AreaShape_Perimeter |
| 595 | 0.016351 | Cytoplasm_Texture_InverseDifferenceMoment_Syto_3 |
| 596 | 0.016289 | Nuclei_Texture_SumEntropy_ER_5 |
| 597 | 0.016201 | Cells_Texture_SumEntropy_Ph_golgi_5 |
| 598 | 0.016190 | Cytoplasm_Texture_SumEntropy_Syto_3 |
| 599 | 0.016046 | Cytoplasm_Intensity_MaxIntensity_Hoechst |
| 600 | 0.016042 | Cells_AreaShape_Zernike_2_2 |
| 601 | 0.016010 | Cytoplasm_Intensity_MaxIntensityEdge_Hoechst |
| 602 | 0.015982 | Cytoplasm_AreaShape_Zernike_1_1 |
| 603 | 0.015881 | Cells_Texture_InverseDifferenceMoment_ER_3 |
| 604 | 0.015833 | Nuclei_Texture_SumVariance_Hoechst_3 |
| 605 | 0.015831 | Nuclei_Intensity_MaxIntensityEdge_Hoechst |
| 606 | 0.015629 | Nuclei_Texture_DifferenceVariance_Ph_golgi_3 |
| 607 | 0.015520 | Cytoplasm_AreaShape_Zernike_9_1 |
| 608 | 0.015389 | Cytoplasm_Texture_InverseDifferenceMoment_Mito_3 |
| 609 | 0.015385 | Cytoplasm_AreaShape_Zernike_8_2 |
| 610 | 0.015273 | Cells_Texture_SumEntropy_Syto_3 |
| 611 | 0.015226 | Cytoplasm_Texture_Correlation_Mito_3 |
| 612 | 0.015172 | Cells_Texture_InfoMeas2_Syto_5 |
| 613 | 0.015166 | Cells_Texture_DifferenceEntropy_Ph_golgi_5 |
| 614 | 0.015132 | Cells_Intensity_IntegratedIntensityEdge_Hoechst |
| 615 | 0.015100 | Cells_AreaShape_Zernike_3_1 |
| 616 | 0.015095 | Nuclei_Intensity_StdIntensity_Hoechst |
| 617 | 0.015083 | Cells_Texture_InfoMeas2_Mito_3 |
| 618 | 0.015042 | Nuclei_Intensity_IntegratedIntensityEdge_Hoechst |
| 619 | 0.014814 | Nuclei_AreaShape_Zernike_2_2 |
| 620 | 0.014505 | Cells_Texture_Entropy_Mito_3 |
| 621 | 0.014423 | Nuclei_Texture_SumVariance_Hoechst_5 |
| 622 | 0.014363 | Cells_Texture_SumEntropy_Mito_3 |
| 623 | 0.014307 | Cytoplasm_Texture_SumEntropy_Syto_5 |
| 624 | 0.014246 | Nuclei_Texture_DifferenceVariance_ER_3 |
| 625 | 0.013974 | Nuclei_Intensity_MinIntensityEdge_Hoechst |
| 626 | 0.013968 | Nuclei_Texture_DifferenceVariance_ER_5 |
| 627 | 0.013965 | Nuclei_AreaShape_Zernike_6_4 |
| 628 | 0.013954 | Cells_RadialDistribution_FracAtD_Ph_golgi_4of4 |
| 629 | 0.013926 | Cytoplasm_Texture_Correlation_Syto_3 |
| 630 | 0.013914 | Cytoplasm_Intensity_LowerQuartileIntensity_Hoechst |
| 631 | 0.013893 | Cells_Texture_SumEntropy_Ph_golgi_3 |
| 632 | 0.013744 | Cytoplasm_Texture_SumEntropy_Ph_golgi_3 |
| 633 | 0.013680 | Nuclei_Intensity_MinIntensity_Hoechst |
| 634 | 0.013625 | Nuclei_Texture_Contrast_Hoechst_5 |
| 635 | 0.013624 | Cells_Texture_Entropy_Mito_5 |
| 636 | 0.013490 | Nuclei_Texture_Contrast_Mito_3 |
| 637 | 0.013457 | Nuclei_AreaShape_Eccentricity |
| 638 | 0.013438 | Cells_Texture_SumEntropy_Mito_5 |
| 639 | 0.013383 | Cytoplasm_Texture_InfoMeas2_Mito_5 |
| 640 | 0.013315 | Nuclei_Texture_InfoMeas1_Hoechst_5 |
| 641 | 0.013283 | Nuclei_AreaShape_Zernike_9_3 |
| 642 | 0.013256 | Cytoplasm_Texture_SumAverage_Ph_golgi_3 |
| 643 | 0.013218 | Cells_RadialDistribution_FracAtD_ER_3of4 |
| 644 | 0.013173 | Cytoplasm_Texture_SumAverage_Ph_golgi_5 |
| 645 | 0.012994 | Cytoplasm_Texture_SumEntropy_Ph_golgi_5 |
| 646 | 0.012974 | Nuclei_Texture_Variance_Hoechst_5 |
| 647 | 0.012958 | Cells_Texture_InverseDifferenceMoment_Ph_golgi_3 |
| 648 | 0.012927 | Nuclei_AreaShape_Zernike_0_0 |
| 649 | 0.012926 | Nuclei_Texture_Variance_Hoechst_3 |
| 650 | 0.012872 | Cells_RadialDistribution_MeanFrac_Syto_4of4 |
| 651 | 0.012859 | Cells_Texture_DifferenceEntropy_Mito_5 |
| 652 | 0.012833 | Nuclei_Texture_DifferenceVariance_Mito_5 |
| 653 | 0.012688 | Nuclei_Texture_InfoMeas2_Syto_3 |
| 654 | 0.012683 | Cytoplasm_Texture_InfoMeas2_ER_5 |
| 655 | 0.012595 | Cells_Texture_InverseDifferenceMoment_Ph_golgi_5 |
| 656 | 0.012437 | Cytoplasm_Intensity_MinIntensity_Hoechst |
| 657 | 0.012436 | Cells_Intensity_MinIntensity_Hoechst |
| 658 | 0.012393 | Cells_AreaShape_Zernike_6_4 |
| 659 | 0.012277 | Nuclei_Texture_AngularSecondMoment_Hoechst_5 |
| 660 | 0.012272 | Nuclei_AreaShape_Zernike_2_0 |
| 661 | 0.012205 | Cytoplasm_AreaShape_Zernike_5_1 |
| 662 | 0.012145 | Nuclei_Texture_DifferenceVariance_Hoechst_5 |
| 663 | 0.012062 | Cytoplasm_AreaShape_Zernike_4_4 |
| 664 | 0.012057 | Cells_Texture_SumEntropy_Syto_5 |
| 665 | 0.012041 | Nuclei_Intensity_MeanIntensityEdge_Hoechst |
| 666 | 0.012036 | Cytoplasm_AreaShape_Perimeter |
| 667 | 0.012032 | Nuclei_AreaShape_Zernike_7_1 |
| 668 | 0.011794 | Cells_Intensity_MinIntensityEdge_Hoechst |
| 669 | 0.011794 | Cytoplasm_Intensity_MinIntensityEdge_Hoechst |
| 670 | 0.011785 | Cells_RadialDistribution_MeanFrac_Mito_4of4 |
| 671 | 0.011773 | Cells_Texture_InfoMeas2_Hoechst_3 |
| 672 | 0.011759 | Cells_Texture_InfoMeas2_ER_3 |
| 673 | 0.011599 | Cytoplasm_Texture_InfoMeas2_Mito_3 |
| 674 | 0.011368 | Cells_Intensity_MaxIntensity_Hoechst |
| 675 | 0.011360 | Nuclei_Intensity_IntegratedIntensity_Hoechst |
| 676 | 0.011260 | Nuclei_Intensity_MaxIntensity_Hoechst |
| 677 | 0.011227 | Cytoplasm_Texture_InfoMeas2_Syto_5 |
| 678 | 0.011106 | Cytoplasm_Texture_Correlation_ER_5 |
| 679 | 0.011098 | Nuclei_Texture_InfoMeas2_Syto_5 |
| 680 | 0.011043 | Nuclei_Texture_InverseDifferenceMoment_Mito_5 |
| 681 | 0.010974 | Nuclei_Texture_AngularSecondMoment_Hoechst_3 |
| 682 | 0.010847 | Nuclei_AreaShape_MinorAxisLength |
| 683 | 0.010679 | Cells_RadialDistribution_FracAtD_Mito_3of4 |
| 684 | 0.010632 | Cells_Texture_InfoMeas2_Syto_3 |
| 685 | 0.010623 | Cytoplasm_AreaShape_Zernike_6_2 |
| 686 | 0.010605 | Cytoplasm_AreaShape_Zernike_9_7 |
| 687 | 0.010565 | Nuclei_Texture_DifferenceEntropy_ER_3 |
| 688 | 0.010521 | Cytoplasm_Texture_InfoMeas2_ER_3 |
| 689 | 0.010470 | Cytoplasm_Texture_InfoMeas2_Syto_3 |
| 690 | 0.010463 | Nuclei_Texture_DifferenceEntropy_ER_5 |
| 691 | 0.010275 | Cells_AreaShape_Zernike_9_9 |
| 692 | 0.010264 | Cells_Texture_InverseDifferenceMoment_Mito_5 |
| 693 | 0.010252 | Nuclei_Texture_InverseDifferenceMoment_ER_5 |
| 694 | 0.010246 | Nuclei_Texture_Correlation_Hoechst_3 |
| 695 | 0.010228 | Cells_Texture_Entropy_Ph_golgi_5 |
| 696 | 0.010220 | Cells_Texture_InfoMeas2_Hoechst_5 |
| 697 | 0.010147 | Cytoplasm_Texture_Correlation_ER_3 |
| 698 | 0.009997 | Cytoplasm_AreaShape_Zernike_9_9 |
| 699 | 0.009917 | Nuclei_Texture_Entropy_Ph_golgi_5 |
| 700 | 0.009832 | Nuclei_AreaShape_Zernike_9_1 |
| 701 | 0.009757 | Cells_AreaShape_Zernike_4_4 |
| 702 | 0.009741 | Nuclei_Texture_DifferenceVariance_Hoechst_3 |
| 703 | 0.009502 | Nuclei_Texture_SumEntropy_Ph_golgi_3 |
| 704 | 0.009498 | Nuclei_Texture_Contrast_Hoechst_3 |
| 705 | 0.009494 | Nuclei_Texture_Entropy_Ph_golgi_3 |
| 706 | 0.009461 | Cells_Texture_Correlation_Hoechst_5 |
| 707 | 0.009450 | Nuclei_Intensity_UpperQuartileIntensity_Hoechst |
| 708 | 0.009376 | Cells_AreaShape_Solidity |
| 709 | 0.009358 | Cells_Texture_Entropy_Ph_golgi_3 |
| 710 | 0.009352 | Cytoplasm_AreaShape_Zernike_7_3 |
| 711 | 0.009329 | Cells_RadialDistribution_MeanFrac_Ph_golgi_4of4 |
| 712 | 0.009155 | Nuclei_Texture_InverseDifferenceMoment_Syto_5 |
| 713 | 0.009125 | Cytoplasm_AreaShape_Zernike_9_5 |
| 714 | 0.009090 | Nuclei_Texture_DifferenceEntropy_Syto_5 |
| 715 | 0.009067 | Nuclei_Texture_SumEntropy_Ph_golgi_5 |
| 716 | 0.009000 | Cells_AreaShape_Zernike_8_6 |
| 717 | 0.008995 | Cells_AreaShape_Eccentricity |
| 718 | 0.008957 | Cells_Texture_InverseDifferenceMoment_Mito_3 |
| 719 | 0.008949 | Cytoplasm_AreaShape_Zernike_5_3 |
| 720 | 0.008904 | Nuclei_Intensity_MeanIntensity_Hoechst |
| 721 | 0.008900 | Cells_Intensity_MassDisplacement_Hoechst |
| 722 | 0.008805 | Cytoplasm_AreaShape_Zernike_6_4 |
| 723 | 0.008791 | Cells_RadialDistribution_FracAtD_Ph_golgi_3of4 |
| 724 | 0.008714 | Cells_AreaShape_Zernike_9_7 |
| 725 | 0.008701 | Nuclei_Intensity_LowerQuartileIntensity_Hoechst |
| 726 | 0.008688 | Nuclei_Intensity_MedianIntensity_Hoechst |
| 727 | 0.008615 | Cells_AreaShape_Zernike_7_7 |
| 728 | 0.008605 | Cytoplasm_AreaShape_Zernike_8_4 |
| 729 | 0.008550 | Nuclei_Texture_InverseDifferenceMoment_ER_3 |
| 730 | 0.008174 | Cytoplasm_Intensity_IntegratedIntensityEdge_Hoechst |
| 731 | 0.008122 | Cytoplasm_AreaShape_Zernike_7_7 |
| 732 | 0.007893 | Nuclei_Texture_DifferenceEntropy_Mito_5 |
| 733 | 0.007847 | Nuclei_AreaShape_Zernike_8_6 |
| 734 | 0.007794 | Cytoplasm_AreaShape_Zernike_6_6 |
| 735 | 0.007781 | Cells_AreaShape_Zernike_4_0 |
| 736 | 0.007730 | Cytoplasm_AreaShape_Zernike_8_6 |
| 737 | 0.007594 | Cytoplasm_AreaShape_Zernike_7_5 |
| 738 | 0.007488 | Cells_RadialDistribution_FracAtD_Syto_3of4 |
| 739 | 0.007478 | Nuclei_Texture_InfoMeas2_Hoechst_3 |
| 740 | 0.007434 | Nuclei_AreaShape_Zernike_6_2 |
| 741 | 0.007409 | Cytoplasm_AreaShape_Zernike_9_3 |
| 742 | 0.007390 | Nuclei_AreaShape_MajorAxisLength |
| 743 | 0.007367 | Nuclei_Texture_DifferenceEntropy_Syto_3 |
| 744 | 0.007151 | Cells_RadialDistribution_MeanFrac_ER_3of4 |
| 745 | 0.007086 | Nuclei_Texture_InverseDifferenceMoment_Ph_golgi_5 |
| 746 | 0.007068 | Nuclei_AreaShape_Area |
| 747 | 0.007028 | Nuclei_Texture_DifferenceEntropy_Ph_golgi_5 |
| 748 | 0.007006 | Cells_AreaShape_Extent |
| 749 | 0.006923 | Nuclei_Texture_Entropy_Syto_5 |
| 750 | 0.006820 | Nuclei_Texture_SumEntropy_Syto_3 |
| 751 | 0.006783 | Nuclei_Texture_DifferenceEntropy_Ph_golgi_3 |
| 752 | 0.006774 | Cells_AreaShape_Zernike_6_6 |
| 753 | 0.006748 | Cells_AreaShape_Zernike_8_0 |
| 754 | 0.006655 | Cytoplasm_AreaShape_Eccentricity |
| 755 | 0.006582 | Nuclei_Texture_InverseDifferenceMoment_Mito_3 |
| 756 | 0.006526 | Cells_AreaShape_Zernike_5_1 |
| 757 | 0.006465 | Nuclei_Texture_InfoMeas2_Hoechst_5 |
| 758 | 0.006443 | Cells_AreaShape_Zernike_3_3 |
| 759 | 0.006412 | Cells_AreaShape_Zernike_0_0 |
| 760 | 0.006374 | Cells_AreaShape_Zernike_5_5 |
| 761 | 0.006242 | Cytoplasm_AreaShape_Zernike_8_8 |
| 762 | 0.006214 | Cells_AreaShape_Zernike_5_3 |
| 763 | 0.006141 | Nuclei_Texture_SumEntropy_Syto_5 |
| 764 | 0.006132 | Cells_AreaShape_Zernike_6_2 |
| 765 | 0.006060 | Cells_AreaShape_Zernike_7_5 |
| 766 | 0.005968 | Nuclei_Texture_InverseDifferenceMoment_Ph_golgi_3 |
| 767 | 0.005875 | Cytoplasm_AreaShape_Zernike_3_3 |
| 768 | 0.005853 | Cells_AreaShape_Zernike_6_0 |
| 769 | 0.005705 | Cytoplasm_AreaShape_Zernike_5_5 |
| 770 | 0.005650 | Cells_AreaShape_Zernike_8_8 |
| 771 | 0.005597 | Nuclei_Texture_DifferenceEntropy_Mito_3 |
| 772 | 0.005581 | Nuclei_Texture_Entropy_Syto_3 |
| 773 | 0.005495 | Nuclei_Texture_InverseDifferenceMoment_Hoechst_5 |
| 774 | 0.005054 | Cells_AreaShape_Zernike_7_1 |
| 775 | 0.004795 | Nuclei_AreaShape_Perimeter |
| 776 | 0.004438 | Cells_AreaShape_Zernike_7_3 |
| 777 | 0.004410 | Cells_RadialDistribution_MeanFrac_Mito_3of4 |
| 778 | 0.004334 | Cells_AreaShape_Zernike_9_5 |
| 779 | 0.004223 | Cells_AreaShape_Zernike_8_4 |
| 780 | 0.004197 | Cells_Texture_Correlation_Hoechst_3 |
| 781 | 0.004065 | Cells_AreaShape_Zernike_9_1 |
| 782 | 0.003981 | Cells_AreaShape_Zernike_2_0 |
| 783 | 0.003940 | Cells_AreaShape_Zernike_1_1 |
| 784 | 0.003807 | Cells_AreaShape_Zernike_9_3 |
| 785 | 0.003760 | Nuclei_Texture_DifferenceEntropy_Hoechst_5 |
| 786 | 0.003688 | Cells_AreaShape_Zernike_8_2 |
| 787 | 0.003389 | Nuclei_Texture_InverseDifferenceMoment_Hoechst_3 |
| 788 | 0.003294 | Nuclei_Texture_SumAverage_Hoechst_5 |
| 789 | 0.003291 | Nuclei_Texture_SumAverage_Hoechst_3 |
| 790 | 0.003291 | Nuclei_Texture_InverseDifferenceMoment_Syto_3 |
| 791 | 0.003220 | Cells_RadialDistribution_MeanFrac_Ph_golgi_3of4 |
| 792 | 0.002860 | Nuclei_Texture_SumEntropy_Hoechst_3 |
| 793 | 0.002842 | Nuclei_Texture_DifferenceEntropy_Hoechst_3 |
| 794 | 0.002806 | Cells_RadialDistribution_MeanFrac_Syto_3of4 |
| 795 | 0.002802 | Nuclei_AreaShape_FormFactor |
| 796 | 0.002715 | Nuclei_Texture_SumEntropy_Hoechst_5 |
| 797 | 0.002462 | Nuclei_Texture_Entropy_Hoechst_5 |
| 798 | 0.002412 | Nuclei_AreaShape_Solidity |
| 799 | 0.002054 | Nuclei_Texture_Entropy_Hoechst_3 |
| 800 | 0.002041 | Nuclei_AreaShape_Extent |
| 801 | 0.000049 | Cells_AreaShape_EulerNumber |
| 802 | 0.000000 | Cells_Children_Cytoplasm_Count |
| 803 | 0.000000 | Nuclei_Children_Cytoplasm_Count |
| 804 | 0.000000 | Nuclei_AreaShape_EulerNumber |
| 805 | 0.000000 | Nuclei_Children_Cells_Count |
