# Supplementary material for: Multiplex Cytological Profiling Assay to Measure Diverse Cellular States
Source: PLoS One. 2013 Dec 2;8(12):e80999. doi: 10.1371/journal.pone.0080999 (PMC3847047; doi:10.1371/journal.pone.0080999)
Supplement: Table S5 — Features ranked by maximal value across the compounds. (DOCX) [file pone.0080999.s013.docx]

**Table S5:** Compound magnitudes

| **Rank** | **Magnitude** | **Feature name** |
| --- | --- | --- |
| 1 | 0.130296 | Nuclei_AreaShape_MinorAxisLength |
| 2 | 0.174928 | Cells_AreaShape_Zernike_7_5 |
| 3 | 0.189972 | Cells_AreaShape_Zernike_7_3 |
| 4 | 0.193624 | Cytoplasm_AreaShape_EulerNumber |
| 5 | 0.196063 | Cells_AreaShape_Zernike_6_0 |
| 6 | 0.201819 | Cells_AreaShape_Zernike_5_1 |
| 7 | 0.211106 | Cells_AreaShape_Zernike_6_2 |
| 8 | 0.242795 | Cells_AreaShape_Zernike_8_6 |
| 9 | 0.243702 | Cells_AreaShape_Extent |
| 10 | 0.251078 | Cells_AreaShape_Zernike_7_1 |
| 11 | 0.251911 | Cells_AreaShape_Zernike_8_4 |
| 12 | 0.288307 | Cells_AreaShape_Zernike_4_4 |
| 13 | 0.293333 | Cells_AreaShape_Zernike_6_4 |
| 14 | 0.299274 | Cells_AreaShape_Zernike_8_2 |
| 15 | 0.300223 | Nuclei_AreaShape_Zernike_8_0 |
| 16 | 0.303871 | Cells_AreaShape_Zernike_3_3 |
| 17 | 0.305447 | Nuclei_AreaShape_Zernike_8_4 |
| 18 | 0.307596 | Cells_AreaShape_Zernike_7_7 |
| 19 | 0.312863 | Nuclei_AreaShape_Zernike_7_7 |
| 20 | 0.330232 | Cells_AreaShape_Zernike_8_8 |
| 21 | 0.334381 | Cells_AreaShape_Zernike_5_3 |
| 22 | 0.339484 | Cells_AreaShape_Zernike_4_0 |
| 23 | 0.350255 | Cells_AreaShape_Zernike_0_0 |
| 24 | 0.366824 | Cells_AreaShape_Zernike_2_2 |
| 25 | 0.374064 | Nuclei_AreaShape_Zernike_3_3 |
| 26 | 0.389986 | Nuclei_AreaShape_Zernike_5_5 |
| 27 | 0.411627 | Cells_AreaShape_Zernike_8_0 |
| 28 | 0.412302 | Cells_AreaShape_Zernike_9_1 |
| 29 | 0.448365 | Nuclei_Intensity_UpperQuartileIntensity_Ph_golgi |
| 30 | 0.452148 | Cells_AreaShape_Zernike_4_2 |
| 31 | 0.452948 | Cytoplasm_AreaShape_Zernike_8_8 |
| 32 | 0.464298 | Nuclei_AreaShape_Zernike_7_1 |
| 33 | 0.483789 | Cells_AreaShape_Zernike_6_6 |
| 34 | 0.485006 | Cells_AreaShape_Zernike_5_5 |
| 35 | 0.492327 | Cells_AreaShape_Zernike_1_1 |
| 36 | 0.493493 | Nuclei_AreaShape_Zernike_6_2 |
| 37 | 0.499792 | Nuclei_AreaShape_Zernike_4_4 |
| 38 | 0.539543 | Cells_Texture_Variance_Ph_golgi_3 |
| 39 | 0.543570 | Nuclei_AreaShape_Zernike_6_0 |
| 40 | 0.546700 | Nuclei_AreaShape_Zernike_6_6 |
| 41 | 0.560931 | Cytoplasm_AreaShape_Zernike_6_4 |
| 42 | 0.562566 | Nuclei_Texture_Variance_Syto_3 |
| 43 | 0.566147 | Nuclei_AreaShape_Zernike_9_1 |
| 44 | 0.567851 | Nuclei_AreaShape_Zernike_3_1 |
| 45 | 0.579179 | Cytoplasm_AreaShape_Zernike_8_6 |
| 46 | 0.583783 | Nuclei_AreaShape_Zernike_5_3 |
| 47 | 0.603910 | Nuclei_AreaShape_Zernike_0_0 |
| 48 | 0.616412 | Nuclei_AreaShape_Zernike_9_3 |
| 49 | 0.629481 | Nuclei_AreaShape_Zernike_8_2 |
| 50 | 0.629953 | Cells_AreaShape_Zernike_3_1 |
| 51 | 0.630488 | Nuclei_AreaShape_Zernike_6_4 |
| 52 | 0.637814 | Cells_AreaShape_Solidity |
| 53 | 0.643180 | Nuclei_AreaShape_Zernike_8_8 |
| 54 | 0.644663 | Cytoplasm_AreaShape_Zernike_8_4 |
| 55 | 0.649820 | Cells_AreaShape_Orientation |
| 56 | 0.656441 | Nuclei_AreaShape_Zernike_4_0 |
| 57 | 0.657456 | Nuclei_AreaShape_Zernike_5_1 |
| 58 | 0.675427 | Nuclei_AreaShape_Zernike_9_5 |
| 59 | 0.681884 | Cytoplasm_AreaShape_Zernike_7_7 |
| 60 | 0.687926 | Cytoplasm_AreaShape_Zernike_4_0 |
| 61 | 0.713524 | Nuclei_AreaShape_Eccentricity |
| 62 | 0.738235 | Nuclei_AreaShape_Zernike_7_3 |
| 63 | 0.741022 | Nuclei_AreaShape_Zernike_7_5 |
| 64 | 0.744127 | Nuclei_Texture_InfoMeas1_Ph_golgi_3 |
| 65 | 0.767947 | Cytoplasm_Texture_SumVariance_Mito_3 |
| 66 | 0.775836 | Cytoplasm_AreaShape_Zernike_6_2 |
| 67 | 0.806393 | Cells_AreaShape_Zernike_2_0 |
| 68 | 0.813826 | Cytoplasm_Texture_SumEntropy_Mito_3 |
| 69 | 0.817803 | Cytoplasm_AreaShape_Zernike_7_5 |
| 70 | 0.847168 | Cytoplasm_AreaShape_Zernike_6_6 |
| 71 | 0.864052 | Cytoplasm_AreaShape_Zernike_7_3 |
| 72 | 0.880869 | Cytoplasm_Texture_SumEntropy_Hoechst_5 |
| 73 | 0.883633 | Cytoplasm_AreaShape_Zernike_0_0 |
| 74 | 0.892285 | Cytoplasm_Texture_SumAverage_Ph_golgi_3 |
| 75 | 0.909244 | Nuclei_Texture_InfoMeas1_ER_3 |
| 76 | 0.910989 | Cytoplasm_AreaShape_Zernike_5_3 |
| 77 | 0.931623 | Cytoplasm_AreaShape_Zernike_8_2 |
| 78 | 0.940682 | Cytoplasm_Texture_InfoMeas2_Mito_3 |
| 79 | 0.949354 | Cytoplasm_AreaShape_Zernike_8_0 |
| 80 | 0.964849 | Nuclei_Texture_Gabor_Mito_3 |
| 81 | 0.984413 | Cytoplasm_Texture_SumAverage_Mito_5 |
| 82 | 0.988000 | Cytoplasm_AreaShape_Zernike_3_3 |
| 83 | 0.998868 | Nuclei_Texture_Gabor_Ph_golgi_5 |
| 84 | 1.003127 | Cytoplasm_Texture_AngularSecondMoment_Hoechst_5 |
| 85 | 1.003320 | Cytoplasm_Texture_AngularSecondMoment_Mito_3 |
| 86 | 1.018260 | Cytoplasm_Texture_SumVariance_Hoechst_5 |
| 87 | 1.021168 | Cytoplasm_AreaShape_Zernike_6_0 |
| 88 | 1.028139 | Cytoplasm_Intensity_UpperQuartileIntensity_Hoechst |
| 89 | 1.030821 | Nuclei_Texture_Gabor_Syto_3 |
| 90 | 1.039702 | Nuclei_Texture_Gabor_Ph_golgi_3 |
| 91 | 1.039811 | Nuclei_AreaShape_Zernike_8_6 |
| 92 | 1.041806 | Cytoplasm_Texture_InfoMeas1_Ph_golgi_3 |
| 93 | 1.050061 | Cytoplasm_AreaShape_Zernike_5_1 |
| 94 | 1.052357 | Nuclei_Texture_InfoMeas1_Hoechst_3 |
| 95 | 1.074085 | Nuclei_Texture_SumVariance_Ph_golgi_3 |
| 96 | 1.087645 | Nuclei_Neighbors_NumberOfNeighbors_1 |
| 97 | 1.099142 | Cytoplasm_AreaShape_Zernike_7_1 |
| 98 | 1.099514 | Cells_RadialDistribution_MeanFrac_Ph_golgi_4of4 |
| 99 | 1.102919 | Nuclei_AreaShape_Zernike_4_2 |
| 100 | 1.122292 | Nuclei_Texture_SumEntropy_Mito_3 |
| 101 | 1.131711 | Cells_RadialDistribution_RadialCV_Syto_1of4 |
| 102 | 1.132439 | Nuclei_Texture_Correlation_Mito_3 |
| 103 | 1.146729 | Nuclei_Texture_InfoMeas1_Syto_3 |
| 104 | 1.148146 | Cells_RadialDistribution_FracAtD_Ph_golgi_4of4 |
| 105 | 1.154723 | Cytoplasm_Intensity_UpperQuartileIntensity_ER |
| 106 | 1.156255 | Nuclei_Intensity_UpperQuartileIntensity_Syto |
| 107 | 1.157152 | Nuclei_Texture_InfoMeas2_Mito_3 |
| 108 | 1.160893 | Nuclei_Intensity_UpperQuartileIntensity_Mito |
| 109 | 1.162402 | Cytoplasm_Texture_InfoMeas1_ER_3 |
| 110 | 1.173220 | Cytoplasm_AreaShape_Zernike_2_2 |
| 111 | 1.181537 | Nuclei_Texture_SumEntropy_Hoechst_5 |
| 112 | 1.183392 | Nuclei_Texture_InfoMeas2_Hoechst_5 |
| 113 | 1.185871 | Nuclei_Texture_InfoMeas2_Ph_golgi_3 |
| 114 | 1.202759 | Cells_RadialDistribution_FracAtD_Syto_1of4 |
| 115 | 1.208862 | Nuclei_Texture_InfoMeas1_Mito_3 |
| 116 | 1.213381 | Nuclei_AreaShape_Zernike_9_7 |
| 117 | 1.218347 | Nuclei_Texture_Contrast_Syto_3 |
| 118 | 1.218868 | Nuclei_Texture_InfoMeas2_Hoechst_3 |
| 119 | 1.226778 | Nuclei_Texture_InfoMeas1_Ph_golgi_5 |
| 120 | 1.230109 | Nuclei_Texture_InfoMeas1_Hoechst_5 |
| 121 | 1.230673 | Cytoplasm_Texture_DifferenceVariance_Ph_golgi_3 |
| 122 | 1.234794 | Cytoplasm_AreaShape_Zernike_4_2 |
| 123 | 1.234905 | Cytoplasm_AreaShape_MinorAxisLength |
| 124 | 1.245697 | Cytoplasm_AreaShape_Zernike_4_4 |
| 125 | 1.257406 | Cells_RadialDistribution_RadialCV_Ph_golgi_4of4 |
| 126 | 1.266055 | Nuclei_Intensity_LowerQuartileIntensity_Mito |
| 127 | 1.287748 | Cells_Texture_SumEntropy_ER_5 |
| 128 | 1.288984 | Cells_Texture_SumEntropy_Mito_3 |
| 129 | 1.292840 | Cytoplasm_AreaShape_Solidity |
| 130 | 1.299141 | Cytoplasm_Texture_SumEntropy_Ph_golgi_3 |
| 131 | 1.299687 | Nuclei_Texture_Entropy_Mito_3 |
| 132 | 1.308033 | Cytoplasm_Intensity_MinIntensityEdge_Hoechst |
| 133 | 1.308057 | Cells_Intensity_MinIntensityEdge_Hoechst |
| 134 | 1.320450 | Cells_Texture_SumEntropy_Hoechst_5 |
| 135 | 1.322421 | Nuclei_AreaShape_Zernike_2_2 |
| 136 | 1.330602 | Cytoplasm_AreaShape_Zernike_5_5 |
| 137 | 1.332640 | Cells_Texture_SumAverage_Ph_golgi_3 |
| 138 | 1.333868 | Cytoplasm_Texture_Entropy_Mito_3 |
| 139 | 1.346407 | Cytoplasm_AreaShape_Zernike_2_0 |
| 140 | 1.361068 | Cytoplasm_AreaShape_Zernike_1_1 |
| 141 | 1.369359 | Cytoplasm_Intensity_MedianIntensity_Hoechst |
| 142 | 1.369359 | Cells_Intensity_MedianIntensity_Hoechst |
| 143 | 1.370115 | Cells_Texture_SumEntropy_Hoechst_3 |
| 144 | 1.371872 | Cells_Texture_SumAverage_Mito_5 |
| 145 | 1.385069 | Cytoplasm_Texture_InfoMeas2_ER_5 |
| 146 | 1.385444 | Cytoplasm_Texture_Gabor_Ph_golgi_3 |
| 147 | 1.395727 | Nuclei_Texture_Entropy_Hoechst_5 |
| 148 | 1.404032 | Cells_RadialDistribution_MeanFrac_Mito_4of4 |
| 149 | 1.404112 | Cytoplasm_Texture_DifferenceEntropy_Syto_3 |
| 150 | 1.407041 | Nuclei_Texture_DifferenceEntropy_Syto_3 |
| 151 | 1.417767 | Cytoplasm_AreaShape_Perimeter |
| 152 | 1.433522 | Nuclei_Texture_Correlation_Ph_golgi_3 |
| 153 | 1.434726 | Cytoplasm_Texture_SumEntropy_Mito_5 |
| 154 | 1.441116 | Nuclei_Texture_SumAverage_Hoechst_5 |
| 155 | 1.444599 | Cytoplasm_Texture_SumVariance_Ph_golgi_3 |
| 156 | 1.446309 | Nuclei_Texture_SumAverage_Mito_3 |
| 157 | 1.456752 | Cells_RadialDistribution_RadialCV_Mito_4of4 |
| 158 | 1.457005 | Nuclei_Texture_SumAverage_Syto_3 |
| 159 | 1.460440 | Nuclei_Children_Cells_Count |
| 160 | 1.473803 | Cells_Texture_Entropy_Hoechst_3 |
| 161 | 1.478598 | Nuclei_Texture_SumVariance_Mito_5 |
| 162 | 1.480133 | Cytoplasm_Intensity_LowerQuartileIntensity_ER |
| 163 | 1.480880 | Cytoplasm_Intensity_LowerQuartileIntensity_Syto |
| 164 | 1.501776 | Cells_Texture_AngularSecondMoment_Hoechst_5 |
| 165 | 1.509078 | Cells_Texture_AngularSecondMoment_Hoechst_3 |
| 166 | 1.513387 | Cytoplasm_Intensity_LowerQuartileIntensity_Mito |
| 167 | 1.515089 | Nuclei_AreaShape_Zernike_1_1 |
| 168 | 1.526381 | Cells_RadialDistribution_RadialCV_Ph_golgi_1of4 |
| 169 | 1.527513 | Cells_RadialDistribution_MeanFrac_Syto_1of4 |
| 170 | 1.534187 | Cytoplasm_Texture_SumEntropy_Hoechst_3 |
| 171 | 1.535252 | Nuclei_Texture_SumVariance_Hoechst_3 |
| 172 | 1.537480 | Nuclei_Intensity_IntegratedIntensityEdge_Hoechst |
| 173 | 1.540564 | Cytoplasm_Texture_DifferenceVariance_Mito_5 |
| 174 | 1.544514 | Cytoplasm_Texture_Variance_ER_3 |
| 175 | 1.546464 | Cells_Texture_Entropy_ER_5 |
| 176 | 1.564435 | Cytoplasm_AreaShape_Orientation |
| 177 | 1.566564 | Cells_Texture_AngularSecondMoment_Mito_3 |
| 178 | 1.570852 | Cells_RadialDistribution_RadialCV_Ph_golgi_3of4 |
| 179 | 1.578347 | Cytoplasm_AreaShape_Zernike_3_1 |
| 180 | 1.579018 | Nuclei_Texture_SumAverage_Ph_golgi_5 |
| 181 | 1.581115 | Cells_Texture_SumVariance_ER_3 |
| 182 | 1.581506 | Cells_Intensity_LowerQuartileIntensity_ER |
| 183 | 1.584257 | Nuclei_Texture_InverseDifferenceMoment_Ph_golgi_3 |
| 184 | 1.587155 | Cells_Texture_AngularSecondMoment_ER_5 |
| 185 | 1.594936 | Nuclei_Texture_AngularSecondMoment_Mito_3 |
| 186 | 1.598196 | Nuclei_Intensity_MassDisplacement_Hoechst |
| 187 | 1.608140 | Cytoplasm_Texture_SumVariance_ER_3 |
| 188 | 1.610982 | Nuclei_Intensity_IntegratedIntensityEdge_Ph_golgi |
| 189 | 1.622358 | Cytoplasm_Texture_Gabor_Ph_golgi_5 |
| 190 | 1.625699 | Cytoplasm_Texture_Correlation_Hoechst_3 |
| 191 | 1.626459 | Cytoplasm_Texture_Entropy_Ph_golgi_5 |
| 192 | 1.631711 | Nuclei_Texture_AngularSecondMoment_ER_3 |
| 193 | 1.631977 | Cells_Texture_SumEntropy_Ph_golgi_3 |
| 194 | 1.633062 | Cells_AreaShape_MinorAxisLength |
| 195 | 1.634591 | Nuclei_Texture_InfoMeas1_ER_5 |
| 196 | 1.634837 | Cytoplasm_Texture_Contrast_Syto_3 |
| 197 | 1.635733 | Cytoplasm_Texture_InfoMeas1_Ph_golgi_5 |
| 198 | 1.636210 | Cytoplasm_Texture_SumVariance_Hoechst_3 |
| 199 | 1.639777 | Cytoplasm_Texture_SumEntropy_ER_5 |
| 200 | 1.644259 | Nuclei_Texture_AngularSecondMoment_Hoechst_5 |
| 201 | 1.645466 | Nuclei_Texture_InfoMeas1_Mito_5 |
| 202 | 1.647342 | Cytoplasm_Texture_SumEntropy_Ph_golgi_5 |
| 203 | 1.647850 | Cells_Texture_SumEntropy_Syto_5 |
| 204 | 1.648824 | Nuclei_Neighbors_PercentTouching_1 |
| 205 | 1.651907 | Cytoplasm_Texture_SumVariance_Syto_5 |
| 206 | 1.652262 | Cells_RadialDistribution_MeanFrac_Ph_golgi_1of4 |
| 207 | 1.655342 | Nuclei_Texture_SumVariance_ER_5 |
| 208 | 1.655523 | Cells_Texture_Variance_ER_3 |
| 209 | 1.657167 | Cytoplasm_Texture_InfoMeas2_Hoechst_3 |
| 210 | 1.657618 | Nuclei_Texture_Entropy_ER_3 |
| 211 | 1.658508 | Nuclei_Texture_InfoMeas2_ER_3 |
| 212 | 1.667193 | Cells_Intensity_LowerQuartileIntensity_Syto |
| 213 | 1.668583 | Nuclei_Texture_SumEntropy_Ph_golgi_3 |
| 214 | 1.669886 | Cytoplasm_Texture_SumVariance_Mito_5 |
| 215 | 1.673356 | Cells_Intensity_UpperQuartileIntensity_Mito |
| 216 | 1.676744 | Cytoplasm_Texture_Entropy_Hoechst_5 |
| 217 | 1.679346 | Nuclei_Texture_SumVariance_Mito_3 |
| 218 | 1.681924 | Cells_Intensity_MassDisplacement_ER |
| 219 | 1.682603 | Nuclei_Intensity_LowerQuartileIntensity_Syto |
| 220 | 1.683994 | Cytoplasm_Texture_DifferenceEntropy_Ph_golgi_5 |
| 221 | 1.691230 | Cells_Intensity_LowerQuartileIntensity_Mito |
| 222 | 1.691722 | Cytoplasm_Texture_Entropy_Syto_3 |
| 223 | 1.696467 | Cells_Texture_SumVariance_Mito_3 |
| 224 | 1.698194 | Nuclei_Intensity_IntegratedIntensityEdge_ER |
| 225 | 1.699922 | Cytoplasm_Texture_SumEntropy_Syto_5 |
| 226 | 1.700645 | Cells_Texture_SumVariance_Syto_5 |
| 227 | 1.702448 | Nuclei_Texture_InverseDifferenceMoment_Mito_3 |
| 228 | 1.710182 | Cytoplasm_Texture_InfoMeas1_Mito_3 |
| 229 | 1.716230 | Cytoplasm_Texture_SumEntropy_Syto_3 |
| 230 | 1.716722 | Nuclei_Texture_SumVariance_Syto_3 |
| 231 | 1.732825 | Cytoplasm_Texture_Entropy_Hoechst_3 |
| 232 | 1.740652 | Cells_Texture_DifferenceEntropy_Syto_3 |
| 233 | 1.741984 | Cytoplasm_Texture_InfoMeas2_Hoechst_5 |
| 234 | 1.745519 | Cells_Intensity_MinIntensity_Mito |
| 235 | 1.753020 | Nuclei_Texture_SumEntropy_Mito_5 |
| 236 | 1.753796 | Nuclei_Texture_DifferenceEntropy_Mito_3 |
| 237 | 1.763560 | Nuclei_Texture_Correlation_Syto_3 |
| 238 | 1.767422 | Cytoplasm_Texture_InfoMeas1_Hoechst_5 |
| 239 | 1.767670 | Cells_Intensity_LowerQuartileIntensity_Hoechst |
| 240 | 1.771500 | Cells_Texture_SumVariance_Hoechst_5 |
| 241 | 1.772337 | Cytoplasm_Texture_Entropy_ER_5 |
| 242 | 1.779696 | Cells_Texture_InfoMeas2_Hoechst_3 |
| 243 | 1.789507 | Cells_RadialDistribution_MeanFrac_Syto_4of4 |
| 244 | 1.795838 | Cells_RadialDistribution_RadialCV_ER_3of4 |
| 245 | 1.805373 | Cells_RadialDistribution_RadialCV_ER_4of4 |
| 246 | 1.813296 | Nuclei_Texture_SumVariance_Hoechst_5 |
| 247 | 1.817328 | Cells_AreaShape_Area |
| 248 | 1.819913 | Cells_RadialDistribution_RadialCV_Ph_golgi_2of4 |
| 249 | 1.829491 | Cells_Texture_DifferenceVariance_Ph_golgi_3 |
| 250 | 1.831624 | Nuclei_Texture_Correlation_ER_3 |
| 251 | 1.833489 | Nuclei_Texture_DifferenceVariance_Syto_3 |
| 252 | 1.839693 | Nuclei_Texture_SumVariance_Ph_golgi_5 |
| 253 | 1.864313 | Cells_Texture_SumEntropy_Mito_5 |
| 254 | 1.875289 | Cytoplasm_Texture_Gabor_Syto_5 |
| 255 | 1.876052 | Cytoplasm_Texture_AngularSecondMoment_Hoechst_3 |
| 256 | 1.884689 | Cytoplasm_AreaShape_Zernike_9_3 |
| 257 | 1.887433 | Cells_Intensity_MinIntensity_Syto |
| 258 | 1.889373 | Cells_RadialDistribution_FracAtD_Syto_3of4 |
| 259 | 1.893079 | Cytoplasm_Texture_SumVariance_ER_5 |
| 260 | 1.903038 | Cytoplasm_Intensity_MinIntensityEdge_Mito |
| 261 | 1.903040 | Cells_Texture_SumAverage_Ph_golgi_5 |
| 262 | 1.905695 | Cytoplasm_Texture_Gabor_Syto_3 |
| 263 | 1.913510 | Nuclei_Texture_Correlation_Hoechst_3 |
| 264 | 1.914540 | Nuclei_Texture_DifferenceEntropy_Ph_golgi_5 |
| 265 | 1.916352 | Cells_Texture_SumAverage_Hoechst_3 |
| 266 | 1.917147 | Cells_RadialDistribution_MeanFrac_Syto_3of4 |
| 267 | 1.920279 | Cytoplasm_Texture_AngularSecondMoment_ER_5 |
| 268 | 1.943623 | Nuclei_Texture_DifferenceEntropy_Mito_5 |
| 269 | 1.949462 | Nuclei_Texture_DifferenceEntropy_Ph_golgi_3 |
| 270 | 1.949584 | Cytoplasm_Texture_InfoMeas1_Mito_5 |
| 271 | 1.954220 | Cells_Texture_SumAverage_ER_5 |
| 272 | 1.960390 | Cells_Intensity_MinIntensityEdge_Mito |
| 273 | 1.960919 | Nuclei_Texture_Correlation_Hoechst_5 |
| 274 | 1.970504 | Cells_Texture_DifferenceVariance_Mito_5 |
| 275 | 1.971314 | Cytoplasm_Texture_Variance_Mito_3 |
| 276 | 1.976603 | Cells_Intensity_MinIntensity_Ph_golgi |
| 277 | 1.991896 | Cells_Texture_SumVariance_Ph_golgi_3 |
| 278 | 1.995741 | Cells_RadialDistribution_FracAtD_ER_4of4 |
| 279 | 2.001755 | Cells_Intensity_MedianIntensity_Mito |
| 280 | 2.003899 | Cytoplasm_Intensity_MedianIntensity_Mito |
| 281 | 2.010293 | Nuclei_Texture_DifferenceVariance_Ph_golgi_5 |
| 282 | 2.010411 | Cells_Texture_Gabor_Ph_golgi_3 |
| 283 | 2.011363 | Nuclei_Texture_InfoMeas2_ER_5 |
| 284 | 2.015989 | Nuclei_Texture_AngularSecondMoment_Mito_5 |
| 285 | 2.017677 | Cells_AreaShape_Zernike_9_5 |
| 286 | 2.019632 | Cytoplasm_Texture_InfoMeas1_ER_5 |
| 287 | 2.029527 | Cells_Texture_SumAverage_Syto_3 |
| 288 | 2.031260 | Cytoplasm_Texture_Variance_Hoechst_5 |
| 289 | 2.034759 | Cells_RadialDistribution_RadialCV_ER_2of4 |
| 290 | 2.040220 | Nuclei_Intensity_IntegratedIntensity_Hoechst |
| 291 | 2.043646 | Cells_Intensity_MassDisplacement_Mito |
| 292 | 2.044448 | Cells_Texture_SumVariance_Mito_5 |
| 293 | 2.051161 | Nuclei_Texture_DifferenceVariance_Syto_5 |
| 294 | 2.051640 | Cells_Texture_Variance_Hoechst_5 |
| 295 | 2.057079 | Cells_Intensity_MassDisplacement_Syto |
| 296 | 2.060639 | Cytoplasm_Intensity_LowerQuartileIntensity_Ph_golgi |
| 297 | 2.061258 | Cells_Texture_Entropy_Mito_3 |
| 298 | 2.064271 | Nuclei_Intensity_MassDisplacement_ER |
| 299 | 2.065902 | Nuclei_Texture_Entropy_Hoechst_3 |
| 300 | 2.070171 | Nuclei_Texture_SumVariance_ER_3 |
| 301 | 2.072604 | Cells_Texture_DifferenceEntropy_Ph_golgi_5 |
| 302 | 2.072778 | Cells_Intensity_MinIntensity_ER |
| 303 | 2.080813 | Nuclei_AreaShape_FormFactor |
| 304 | 2.082587 | Cells_Texture_InfoMeas2_ER_5 |
| 305 | 2.084702 | Cells_RadialDistribution_FracAtD_Mito_4of4 |
| 306 | 2.087191 | Cytoplasm_Texture_InverseDifferenceMoment_Hoechst_3 |
| 307 | 2.098310 | Nuclei_Texture_DifferenceVariance_Mito_5 |
| 308 | 2.102280 | Nuclei_AreaShape_Zernike_2_0 |
| 309 | 2.103429 | Cells_Texture_Variance_Mito_3 |
| 310 | 2.106978 | Cells_Texture_DifferenceVariance_Syto_3 |
| 311 | 2.107461 | Cells_RadialDistribution_RadialCV_Syto_3of4 |
| 312 | 2.108602 | Cells_RadialDistribution_MeanFrac_Syto_2of4 |
| 313 | 2.112577 | Cytoplasm_Texture_DifferenceEntropy_Hoechst_3 |
| 314 | 2.115871 | Cells_RadialDistribution_RadialCV_ER_1of4 |
| 315 | 2.116655 | Nuclei_Texture_SumEntropy_Ph_golgi_5 |
| 316 | 2.120408 | Nuclei_Texture_Entropy_ER_5 |
| 317 | 2.123383 | Nuclei_Texture_DifferenceEntropy_Hoechst_3 |
| 318 | 2.144931 | Nuclei_Texture_SumEntropy_Syto_3 |
| 319 | 2.149379 | Nuclei_Texture_DifferenceEntropy_Hoechst_5 |
| 320 | 2.152337 | Nuclei_Texture_Entropy_Mito_5 |
| 321 | 2.157538 | Cells_RadialDistribution_MeanFrac_Ph_golgi_3of4 |
| 322 | 2.166170 | Nuclei_Texture_Correlation_Syto_5 |
| 323 | 2.170328 | Nuclei_Texture_Entropy_Ph_golgi_3 |
| 324 | 2.173647 | Cytoplasm_Texture_SumVariance_Syto_3 |
| 325 | 2.175306 | Nuclei_Texture_DifferenceVariance_Ph_golgi_3 |
| 326 | 2.177522 | Nuclei_Texture_Variance_ER_5 |
| 327 | 2.177643 | Nuclei_Texture_Variance_Syto_5 |
| 328 | 2.182782 | Nuclei_Texture_InverseDifferenceMoment_Hoechst_3 |
| 329 | 2.183582 | Nuclei_Texture_Correlation_Ph_golgi_5 |
| 330 | 2.192551 | Cells_RadialDistribution_RadialCV_Mito_3of4 |
| 331 | 2.193309 | Cytoplasm_Texture_Gabor_Mito_5 |
| 332 | 2.195672 | Nuclei_Texture_Variance_Mito_5 |
| 333 | 2.203025 | Nuclei_Texture_Variance_Hoechst_3 |
| 334 | 2.207033 | Nuclei_Intensity_StdIntensity_Hoechst |
| 335 | 2.210462 | Nuclei_Texture_AngularSecondMoment_Ph_golgi_3 |
| 336 | 2.212649 | Nuclei_AreaShape_MajorAxisLength |
| 337 | 2.220034 | Nuclei_Intensity_LowerQuartileIntensity_Ph_golgi |
| 338 | 2.223140 | Nuclei_Texture_Gabor_Syto_5 |
| 339 | 2.223221 | Cells_Texture_Correlation_Syto_3 |
| 340 | 2.229684 | Cells_Texture_Variance_Syto_5 |
| 341 | 2.232042 | Cells_Texture_InfoMeas1_ER_3 |
| 342 | 2.246565 | Cells_Texture_Entropy_Hoechst_5 |
| 343 | 2.249949 | Nuclei_Texture_Variance_Ph_golgi_3 |
| 344 | 2.251416 | Cells_RadialDistribution_RadialCV_Mito_2of4 |
| 345 | 2.255363 | Nuclei_Texture_SumEntropy_Hoechst_3 |
| 346 | 2.264962 | Cells_Intensity_UpperQuartileIntensity_Ph_golgi |
| 347 | 2.268344 | Cytoplasm_Texture_DifferenceEntropy_ER_3 |
| 348 | 2.272439 | Nuclei_Texture_InfoMeas2_Syto_3 |
| 349 | 2.273725 | Nuclei_Texture_SumEntropy_ER_5 |
| 350 | 2.275584 | Nuclei_Texture_Contrast_Ph_golgi_5 |
| 351 | 2.279915 | Nuclei_Texture_DifferenceEntropy_ER_3 |
| 352 | 2.282281 | Cells_Texture_Correlation_Hoechst_3 |
| 353 | 2.282748 | Nuclei_Texture_InverseDifferenceMoment_Hoechst_5 |
| 354 | 2.303763 | Cytoplasm_Texture_AngularSecondMoment_Syto_3 |
| 355 | 2.307747 | Nuclei_Texture_SumEntropy_Syto_5 |
| 356 | 2.312817 | Cells_Children_Cytoplasm_Count |
| 357 | 2.317106 | Cells_AreaShape_FormFactor |
| 358 | 2.326139 | Nuclei_Texture_InverseDifferenceMoment_ER_3 |
| 359 | 2.328486 | Nuclei_Texture_DifferenceEntropy_Syto_5 |
| 360 | 2.331824 | Cells_Neighbors_PercentTouching_5 |
| 361 | 2.336126 | Cells_RadialDistribution_MeanFrac_ER_4of4 |
| 362 | 2.339023 | Cytoplasm_Texture_SumVariance_Ph_golgi_5 |
| 363 | 2.339973 | Cytoplasm_Texture_DifferenceEntropy_ER_5 |
| 364 | 2.345429 | Cells_Intensity_MaxIntensity_Mito |
| 365 | 2.345826 | Cytoplasm_AreaShape_Extent |
| 366 | 2.350755 | Cytoplasm_Texture_SumAverage_ER_5 |
| 367 | 2.352775 | Cells_AreaShape_Zernike_9_7 |
| 368 | 2.356739 | Cells_AreaShape_Perimeter |
| 369 | 2.360371 | Cytoplasm_Texture_SumAverage_Hoechst_3 |
| 370 | 2.362347 | Nuclei_Texture_SumAverage_Ph_golgi_3 |
| 371 | 2.366366 | Cells_Texture_InverseDifferenceMoment_Hoechst_3 |
| 372 | 2.370656 | Cytoplasm_Intensity_LowerQuartileIntensity_Hoechst |
| 373 | 2.375037 | Cells_RadialDistribution_RadialCV_Mito_1of4 |
| 374 | 2.378818 | Cells_Intensity_StdIntensityEdge_Syto |
| 375 | 2.387483 | Cytoplasm_Texture_InfoMeas1_Hoechst_3 |
| 376 | 2.399148 | Cells_Texture_InfoMeas1_Hoechst_3 |
| 377 | 2.407628 | Nuclei_Intensity_IntegratedIntensity_ER |
| 378 | 2.412117 | Cells_Texture_SumAverage_Mito_3 |
| 379 | 2.414397 | Cytoplasm_Texture_InfoMeas2_ER_3 |
| 380 | 2.423318 | Cells_AreaShape_Zernike_9_9 |
| 381 | 2.428692 | Cells_AreaShape_Zernike_9_3 |
| 382 | 2.433804 | Cytoplasm_AreaShape_Zernike_9_5 |
| 383 | 2.438553 | Nuclei_Texture_SumAverage_Mito_5 |
| 384 | 2.444111 | Nuclei_Texture_Variance_Hoechst_5 |
| 385 | 2.456488 | Cells_Texture_InfoMeas1_ER_5 |
| 386 | 2.465727 | Cells_RadialDistribution_RadialCV_Syto_2of4 |
| 387 | 2.465817 | Cells_Intensity_MassDisplacement_Ph_golgi |
| 388 | 2.467899 | Nuclei_Texture_Contrast_Ph_golgi_3 |
| 389 | 2.481672 | Cytoplasm_Texture_DifferenceVariance_Syto_5 |
| 390 | 2.482027 | Cytoplasm_Texture_Correlation_Ph_golgi_3 |
| 391 | 2.485085 | Nuclei_Texture_InfoMeas2_Syto_5 |
| 392 | 2.489250 | Cells_RadialDistribution_FracAtD_ER_1of4 |
| 393 | 2.498095 | Nuclei_Texture_InfoMeas2_Mito_5 |
| 394 | 2.503329 | Cytoplasm_Texture_Entropy_ER_3 |
| 395 | 2.505171 | Cytoplasm_AreaShape_Zernike_9_1 |
| 396 | 2.509215 | Cytoplasm_AreaShape_Zernike_9_9 |
| 397 | 2.523573 | Cytoplasm_Texture_InverseDifferenceMoment_ER_3 |
| 398 | 2.524143 | Cells_Intensity_MaxIntensity_ER |
| 399 | 2.532094 | Cytoplasm_Texture_Correlation_Syto_3 |
| 400 | 2.532456 | Nuclei_Texture_DifferenceVariance_Hoechst_3 |
| 401 | 2.532570 | Nuclei_Texture_Variance_Mito_3 |
| 402 | 2.539110 | Nuclei_Texture_Entropy_Syto_3 |
| 403 | 2.547594 | Cells_Texture_SumAverage_Hoechst_5 |
| 404 | 2.561443 | Nuclei_Intensity_StdIntensityEdge_Hoechst |
| 405 | 2.565060 | Nuclei_Texture_DifferenceVariance_ER_3 |
| 406 | 2.566804 | Cells_Texture_DifferenceEntropy_Hoechst_3 |
| 407 | 2.567664 | Cytoplasm_Intensity_MassDisplacement_Syto |
| 408 | 2.570842 | Nuclei_Intensity_MaxIntensity_ER |
| 409 | 2.571373 | Cells_Intensity_UpperQuartileIntensity_Hoechst |
| 410 | 2.574516 | Nuclei_Texture_SumAverage_Hoechst_3 |
| 411 | 2.576618 | Cytoplasm_Texture_SumAverage_Mito_3 |
| 412 | 2.577787 | Cytoplasm_Intensity_MaxIntensityEdge_ER |
| 413 | 2.583747 | Cells_RadialDistribution_FracAtD_Mito_1of4 |
| 414 | 2.584302 | Nuclei_Texture_SumAverage_ER_5 |
| 415 | 2.597712 | Cytoplasm_Texture_SumAverage_Hoechst_5 |
| 416 | 2.614903 | Cells_RadialDistribution_FracAtD_Syto_2of4 |
| 417 | 2.616951 | Nuclei_AreaShape_Solidity |
| 418 | 2.619781 | Cytoplasm_Intensity_MinIntensityEdge_ER |
| 419 | 2.623168 | Nuclei_Intensity_MaxIntensityEdge_Hoechst |
| 420 | 2.626498 | Cells_Intensity_LowerQuartileIntensity_Ph_golgi |
| 421 | 2.631618 | Nuclei_Texture_InverseDifferenceMoment_Mito_5 |
| 422 | 2.634133 | Cytoplasm_Intensity_MassDisplacement_Ph_golgi |
| 423 | 2.662649 | Nuclei_Texture_InverseDifferenceMoment_Ph_golgi_5 |
| 424 | 2.665115 | Cells_Texture_DifferenceEntropy_ER_3 |
| 425 | 2.665213 | Cells_Intensity_MinIntensityEdge_ER |
| 426 | 2.666290 | Nuclei_Texture_InverseDifferenceMoment_Syto_3 |
| 427 | 2.668164 | Cells_Intensity_IntegratedIntensity_Mito |
| 428 | 2.668514 | Cytoplasm_Texture_InfoMeas2_Ph_golgi_3 |
| 429 | 2.672258 | Cells_Texture_Correlation_Ph_golgi_3 |
| 430 | 2.672829 | Cells_Intensity_MaxIntensityEdge_Syto |
| 431 | 2.675569 | Nuclei_Texture_AngularSecondMoment_Syto_3 |
| 432 | 2.685330 | Cells_Intensity_MaxIntensityEdge_ER |
| 433 | 2.690422 | Cells_Intensity_MaxIntensityEdge_Ph_golgi |
| 434 | 2.697607 | Cytoplasm_AreaShape_MajorAxisLength |
| 435 | 2.697744 | Nuclei_Texture_SumVariance_Syto_5 |
| 436 | 2.698342 | Cytoplasm_AreaShape_Zernike_9_7 |
| 437 | 2.703203 | Nuclei_Texture_Gabor_ER_3 |
| 438 | 2.703462 | Cytoplasm_Intensity_IntegratedIntensity_Mito |
| 439 | 2.718528 | Nuclei_Texture_SumEntropy_ER_3 |
| 440 | 2.726670 | Cytoplasm_Intensity_MinIntensityEdge_Syto |
| 441 | 2.729373 | Cells_Intensity_MedianIntensity_ER |
| 442 | 2.729740 | Cytoplasm_Texture_SumAverage_Syto_5 |
| 443 | 2.730870 | Nuclei_Intensity_MaxIntensityEdge_ER |
| 444 | 2.734274 | Cytoplasm_Intensity_MedianIntensity_ER |
| 445 | 2.745326 | Cells_Texture_DifferenceEntropy_ER_5 |
| 446 | 2.751250 | Cytoplasm_Texture_SumEntropy_ER_3 |
| 447 | 2.754539 | Cytoplasm_Texture_InverseDifferenceMoment_ER_5 |
| 448 | 2.754545 | Cells_Intensity_MinIntensityEdge_Syto |
| 449 | 2.754951 | Cells_Texture_AngularSecondMoment_Syto_3 |
| 450 | 2.755944 | Nuclei_Texture_DifferenceVariance_Hoechst_5 |
| 451 | 2.760771 | Cells_Texture_SumVariance_Ph_golgi_5 |
| 452 | 2.778035 | Cells_Texture_InfoMeas1_Mito_3 |
| 453 | 2.783303 | Cells_Intensity_MassDisplacement_Hoechst |
| 454 | 2.798814 | Cells_Texture_DifferenceVariance_Ph_golgi_5 |
| 455 | 2.804550 | Cells_Intensity_MedianIntensity_Syto |
| 456 | 2.807807 | Cytoplasm_Intensity_MedianIntensity_Syto |
| 457 | 2.809038 | Cytoplasm_Texture_Variance_Hoechst_3 |
| 458 | 2.812284 | Cells_RadialDistribution_FracAtD_Ph_golgi_1of4 |
| 459 | 2.816212 | Nuclei_Texture_Variance_ER_3 |
| 460 | 2.821582 | Nuclei_Intensity_MaxIntensity_Hoechst |
| 461 | 2.823719 | Cells_RadialDistribution_FracAtD_Syto_4of4 |
| 462 | 2.842757 | Cytoplasm_Texture_InverseDifferenceMoment_Mito_3 |
| 463 | 2.847110 | Cells_Texture_SumAverage_ER_3 |
| 464 | 2.857345 | Nuclei_Intensity_UpperQuartileIntensity_Hoechst |
| 465 | 2.869107 | Cytoplasm_Texture_Gabor_Mito_3 |
| 466 | 2.874553 | Cells_Texture_SumVariance_Syto_3 |
| 467 | 2.881225 | Cells_Intensity_MinIntensity_Hoechst |
| 468 | 2.884447 | Nuclei_Intensity_IntegratedIntensityEdge_Syto |
| 469 | 2.886824 | Nuclei_Texture_InfoMeas2_Ph_golgi_5 |
| 470 | 2.887977 | Cytoplasm_Intensity_MaxIntensityEdge_Ph_golgi |
| 471 | 2.889754 | Cells_Texture_SumVariance_Hoechst_3 |
| 472 | 2.891497 | Cytoplasm_Texture_Correlation_Syto_5 |
| 473 | 2.904662 | Cytoplasm_Texture_Variance_ER_5 |
| 474 | 2.948222 | Cytoplasm_Texture_InfoMeas1_Syto_3 |
| 475 | 2.950266 | Cells_Texture_InverseDifferenceMoment_Syto_5 |
| 476 | 2.954865 | Cytoplasm_Texture_Entropy_Ph_golgi_3 |
| 477 | 2.957869 | Nuclei_Texture_InverseDifferenceMoment_Syto_5 |
| 478 | 2.970898 | Cells_Intensity_UpperQuartileIntensity_ER |
| 479 | 2.980463 | Nuclei_Texture_Contrast_Mito_5 |
| 480 | 2.996112 | Cytoplasm_Intensity_MaxIntensityEdge_Syto |
| 481 | 3.003542 | Cytoplasm_Intensity_UpperQuartileIntensity_Syto |
| 482 | 3.003958 | Cells_Texture_InfoMeas2_ER_3 |
| 483 | 3.017936 | Nuclei_Texture_SumAverage_ER_3 |
| 484 | 3.017949 | Nuclei_Texture_Correlation_ER_5 |
| 485 | 3.022118 | Cells_Texture_InfoMeas1_Ph_golgi_3 |
| 486 | 3.040439 | Cells_Texture_InfoMeas2_Ph_golgi_3 |
| 487 | 3.044351 | Nuclei_Texture_Contrast_Mito_3 |
| 488 | 3.048307 | Nuclei_Intensity_StdIntensityEdge_ER |
| 489 | 3.050878 | Nuclei_Texture_AngularSecondMoment_ER_5 |
| 490 | 3.051280 | Cytoplasm_Intensity_MinIntensity_Syto |
| 491 | 3.057673 | Cytoplasm_Texture_InfoMeas1_Syto_5 |
| 492 | 3.059955 | Cytoplasm_Texture_AngularSecondMoment_ER_3 |
| 493 | 3.063136 | Cytoplasm_Texture_Correlation_Mito_5 |
| 494 | 3.068217 | Cytoplasm_Intensity_StdIntensityEdge_Syto |
| 495 | 3.085630 | Cytoplasm_Texture_SumAverage_ER_3 |
| 496 | 3.086190 | Nuclei_Texture_AngularSecondMoment_Hoechst_3 |
| 497 | 3.089733 | Nuclei_Texture_AngularSecondMoment_Syto_5 |
| 498 | 3.094598 | Cells_Texture_DifferenceVariance_Syto_5 |
| 499 | 3.096216 | Cells_Texture_SumVariance_ER_5 |
| 500 | 3.098164 | Cells_Texture_Gabor_Mito_5 |
| 501 | 3.099844 | Cells_Intensity_IntegratedIntensityEdge_Hoechst |
| 502 | 3.104418 | Cytoplasm_Texture_DifferenceEntropy_Mito_3 |
| 503 | 3.114478 | Cytoplasm_AreaShape_Eccentricity |
| 504 | 3.128139 | Cells_RadialDistribution_MeanFrac_Mito_1of4 |
| 505 | 3.135244 | Nuclei_Intensity_UpperQuartileIntensity_ER |
| 506 | 3.135560 | Cytoplasm_Intensity_MeanIntensity_Mito |
| 507 | 3.137984 | Cytoplasm_Texture_Gabor_Hoechst_3 |
| 508 | 3.142098 | Cells_Texture_InverseDifferenceMoment_Syto_3 |
| 509 | 3.143950 | Cells_Texture_Entropy_ER_3 |
| 510 | 3.145114 | Nuclei_Intensity_StdIntensity_ER |
| 511 | 3.146574 | Cytoplasm_Intensity_MaxIntensity_Mito |
| 512 | 3.147553 | Nuclei_Intensity_MedianIntensity_Hoechst |
| 513 | 3.152666 | Cells_Intensity_MeanIntensity_Syto |
| 514 | 3.155240 | Cells_Texture_Correlation_Ph_golgi_5 |
| 515 | 3.157649 | Cells_Texture_InverseDifferenceMoment_ER_3 |
| 516 | 3.161456 | Cells_Intensity_StdIntensity_Syto |
| 517 | 3.163208 | Nuclei_Texture_DifferenceEntropy_ER_5 |
| 518 | 3.167990 | Cytoplasm_Intensity_MassDisplacement_ER |
| 519 | 3.171166 | Cells_Texture_Variance_Syto_3 |
| 520 | 3.172984 | Cytoplasm_Texture_InverseDifferenceMoment_Syto_5 |
| 521 | 3.177964 | Nuclei_Intensity_MedianIntensity_Mito |
| 522 | 3.191554 | Cells_Texture_InverseDifferenceMoment_ER_5 |
| 523 | 3.192409 | Nuclei_Children_Cytoplasm_Count |
| 524 | 3.197291 | Cytoplasm_Texture_Gabor_ER_3 |
| 525 | 3.205634 | Cytoplasm_AreaShape_Area |
| 526 | 3.228656 | Cells_Texture_Variance_ER_5 |
| 527 | 3.232229 | Cells_Texture_Variance_Hoechst_3 |
| 528 | 3.242768 | Nuclei_Texture_Contrast_ER_3 |
| 529 | 3.247768 | Cells_RadialDistribution_MeanFrac_Ph_golgi_2of4 |
| 530 | 3.265645 | Nuclei_Texture_Correlation_Mito_5 |
| 531 | 3.267377 | Cells_Intensity_MaxIntensity_Syto |
| 532 | 3.273199 | Nuclei_Intensity_MeanIntensity_Hoechst |
| 533 | 3.287898 | Nuclei_Texture_SumAverage_Syto_5 |
| 534 | 3.290117 | Nuclei_Texture_DifferenceVariance_Mito_3 |
| 535 | 3.295036 | Cytoplasm_Texture_Correlation_ER_5 |
| 536 | 3.297374 | Cytoplasm_Intensity_StdIntensity_Syto |
| 537 | 3.298232 | Cells_Texture_InverseDifferenceMoment_Mito_3 |
| 538 | 3.308192 | Nuclei_Texture_Contrast_Hoechst_5 |
| 539 | 3.312125 | Cytoplasm_Texture_Contrast_Ph_golgi_5 |
| 540 | 3.316153 | Cells_AreaShape_EulerNumber |
| 541 | 3.328950 | Cells_Texture_Correlation_Mito_5 |
| 542 | 3.342618 | Cells_Texture_Gabor_Syto_5 |
| 543 | 3.345633 | Cells_Texture_SumAverage_Syto_5 |
| 544 | 3.372941 | Cells_Texture_InverseDifferenceMoment_Ph_golgi_5 |
| 545 | 3.376697 | Cytoplasm_Texture_Correlation_Ph_golgi_5 |
| 546 | 3.382863 | Nuclei_Intensity_MedianIntensity_Syto |
| 547 | 3.393119 | Cells_AreaShape_Eccentricity |
| 548 | 3.395606 | Cells_Texture_SumEntropy_ER_3 |
| 549 | 3.401740 | Nuclei_AreaShape_Area |
| 550 | 3.410878 | Nuclei_Intensity_IntegratedIntensityEdge_Mito |
| 551 | 3.410889 | Nuclei_Intensity_MassDisplacement_Mito |
| 552 | 3.410975 | Cells_Intensity_IntegratedIntensity_Syto |
| 553 | 3.425962 | Cytoplasm_Texture_SumAverage_Syto_3 |
| 554 | 3.431042 | Cells_Neighbors_NumberOfNeighbors_5 |
| 555 | 3.435734 | Cells_Texture_InfoMeas2_Syto_3 |
| 556 | 3.438291 | Nuclei_Texture_InverseDifferenceMoment_ER_5 |
| 557 | 3.460530 | Nuclei_Intensity_MinIntensityEdge_Syto |
| 558 | 3.467201 | Nuclei_Intensity_MinIntensityEdge_Mito |
| 559 | 3.467445 | Cells_Texture_Gabor_Mito_3 |
| 560 | 3.482442 | Nuclei_Texture_DifferenceVariance_ER_5 |
| 561 | 3.497063 | Cells_Texture_Correlation_Syto_5 |
| 562 | 3.501974 | Cytoplasm_Texture_Gabor_Hoechst_5 |
| 563 | 3.505998 | Cells_RadialDistribution_FracAtD_Mito_3of4 |
| 564 | 3.513398 | Cells_Texture_SumEntropy_Ph_golgi_5 |
| 565 | 3.525554 | Nuclei_Texture_Gabor_Hoechst_3 |
| 566 | 3.530261 | Nuclei_Texture_Gabor_Hoechst_5 |
| 567 | 3.533789 | Nuclei_Intensity_MinIntensity_Mito |
| 568 | 3.545925 | Nuclei_Texture_Contrast_Hoechst_3 |
| 569 | 3.553250 | Nuclei_AreaShape_Zernike_9_9 |
| 570 | 3.554686 | Cells_RadialDistribution_MeanFrac_ER_1of4 |
| 571 | 3.556921 | Nuclei_Intensity_LowerQuartileIntensity_Hoechst |
| 572 | 3.568495 | Cells_RadialDistribution_FracAtD_ER_3of4 |
| 573 | 3.570337 | Cells_AreaShape_MajorAxisLength |
| 574 | 3.572264 | Cells_Texture_SumEntropy_Syto_3 |
| 575 | 3.574106 | Nuclei_Intensity_MeanIntensity_ER |
| 576 | 3.584262 | Nuclei_Intensity_MaxIntensityEdge_Mito |
| 577 | 3.589608 | Nuclei_Texture_AngularSecondMoment_Ph_golgi_5 |
| 578 | 3.595377 | Nuclei_Intensity_MedianIntensity_ER |
| 579 | 3.600250 | Cytoplasm_Intensity_MeanIntensity_Syto |
| 580 | 3.651469 | Cytoplasm_Texture_InverseDifferenceMoment_Ph_golgi_3 |
| 581 | 3.663854 | Cytoplasm_Texture_DifferenceEntropy_Hoechst_5 |
| 582 | 3.665524 | Cytoplasm_Intensity_MinIntensityEdge_Ph_golgi |
| 583 | 3.665726 | Cytoplasm_Intensity_IntegratedIntensity_Syto |
| 584 | 3.671142 | Cytoplasm_Texture_AngularSecondMoment_Ph_golgi_5 |
| 585 | 3.688000 | Cells_Intensity_MinIntensityEdge_Ph_golgi |
| 586 | 3.712303 | Nuclei_Texture_InfoMeas1_Syto_5 |
| 587 | 3.716566 | Cells_RadialDistribution_MeanFrac_ER_3of4 |
| 588 | 3.718556 | Cells_Intensity_MeanIntensityEdge_Syto |
| 589 | 3.720968 | Cytoplasm_Texture_SumAverage_Ph_golgi_5 |
| 590 | 3.726308 | Cytoplasm_Texture_InverseDifferenceMoment_Mito_5 |
| 591 | 3.727262 | Nuclei_Intensity_MeanIntensityEdge_Hoechst |
| 592 | 3.734124 | Cytoplasm_Intensity_IntegratedIntensity_Hoechst |
| 593 | 3.743947 | Cells_Texture_Correlation_ER_5 |
| 594 | 3.780132 | Cytoplasm_Texture_Correlation_Mito_3 |
| 595 | 3.790842 | Nuclei_Intensity_MeanIntensityEdge_ER |
| 596 | 3.792472 | Cytoplasm_Texture_DifferenceVariance_Hoechst_3 |
| 597 | 3.793047 | Cells_Texture_InverseDifferenceMoment_Ph_golgi_3 |
| 598 | 3.800290 | Cells_RadialDistribution_RadialCV_Syto_4of4 |
| 599 | 3.811557 | Cytoplasm_Intensity_MeanIntensityEdge_Syto |
| 600 | 3.818203 | Cells_Texture_Gabor_Syto_3 |
| 601 | 3.818270 | Cytoplasm_Intensity_MaxIntensity_Syto |
| 602 | 3.818952 | Cytoplasm_Texture_InfoMeas2_Syto_5 |
| 603 | 3.826428 | Cells_Intensity_StdIntensity_Ph_golgi |
| 604 | 3.827755 | Cytoplasm_Texture_Contrast_ER_3 |
| 605 | 3.839051 | Cytoplasm_Intensity_UpperQuartileIntensity_Ph_golgi |
| 606 | 3.856665 | Cytoplasm_Texture_InverseDifferenceMoment_Hoechst_5 |
| 607 | 3.860828 | Cells_Texture_InverseDifferenceMoment_Mito_5 |
| 608 | 3.868143 | Cytoplasm_Texture_InfoMeas2_Mito_5 |
| 609 | 3.870238 | Cytoplasm_Texture_DifferenceVariance_ER_3 |
| 610 | 3.884280 | Cells_Texture_DifferenceVariance_ER_3 |
| 611 | 3.887572 | Cells_Intensity_StdIntensityEdge_Ph_golgi |
| 612 | 3.891471 | Cells_Texture_DifferenceEntropy_Mito_3 |
| 613 | 3.896397 | Cells_Texture_DifferenceEntropy_Ph_golgi_3 |
| 614 | 3.897588 | Cells_Texture_Gabor_Hoechst_3 |
| 615 | 3.898358 | Cells_Texture_AngularSecondMoment_ER_3 |
| 616 | 3.920579 | Cytoplasm_Intensity_MaxIntensityEdge_Mito |
| 617 | 3.929297 | Cytoplasm_Intensity_MinIntensity_Ph_golgi |
| 618 | 3.946459 | Cytoplasm_Intensity_MaxIntensity_ER |
| 619 | 3.952274 | Cells_RadialDistribution_FracAtD_Mito_2of4 |
| 620 | 3.955401 | Cells_RadialDistribution_MeanFrac_Mito_3of4 |
| 621 | 3.957787 | Cells_Intensity_UpperQuartileIntensity_Syto |
| 622 | 3.966569 | Cytoplasm_Intensity_IntegratedIntensity_ER |
| 623 | 3.971294 | Nuclei_Intensity_MinIntensity_Ph_golgi |
| 624 | 4.015907 | Nuclei_Intensity_MinIntensityEdge_Hoechst |
| 625 | 4.027360 | Cells_Texture_InfoMeas1_Hoechst_5 |
| 626 | 4.039012 | Nuclei_Intensity_MinIntensity_Hoechst |
| 627 | 4.047058 | Nuclei_Intensity_LowerQuartileIntensity_ER |
| 628 | 4.050280 | Cytoplasm_Texture_Correlation_ER_3 |
| 629 | 4.050907 | Cytoplasm_AreaShape_FormFactor |
| 630 | 4.072474 | Cytoplasm_Intensity_StdIntensityEdge_Ph_golgi |
| 631 | 4.088194 | Nuclei_Intensity_MinIntensityEdge_ER |
| 632 | 4.088688 | Cytoplasm_Texture_DifferenceVariance_ER_5 |
| 633 | 4.121023 | Cytoplasm_Intensity_StdIntensity_Ph_golgi |
| 634 | 4.134435 | Cells_RadialDistribution_FracAtD_ER_2of4 |
| 635 | 4.158060 | Cells_Intensity_IntegratedIntensity_ER |
| 636 | 4.174748 | Cells_RadialDistribution_FracAtD_Ph_golgi_3of4 |
| 637 | 4.188146 | Cells_Texture_DifferenceEntropy_Hoechst_5 |
| 638 | 4.197400 | Nuclei_Intensity_MinIntensity_ER |
| 639 | 4.228593 | Cells_Intensity_MedianIntensity_Ph_golgi |
| 640 | 4.239595 | Cells_Texture_Correlation_ER_3 |
| 641 | 4.259525 | Cytoplasm_Intensity_MedianIntensity_Ph_golgi |
| 642 | 4.261660 | Cytoplasm_Intensity_UpperQuartileIntensity_Mito |
| 643 | 4.304520 | Cytoplasm_Intensity_MassDisplacement_Mito |
| 644 | 4.309213 | Cells_Texture_InfoMeas2_Mito_5 |
| 645 | 4.342728 | Cells_Texture_DifferenceVariance_ER_5 |
| 646 | 4.373730 | Nuclei_Texture_Contrast_Syto_5 |
| 647 | 4.375870 | Cells_Intensity_MaxIntensityEdge_Mito |
| 648 | 4.395222 | Cells_Intensity_IntegratedIntensityEdge_Syto |
| 649 | 4.431090 | Cytoplasm_Texture_Contrast_Hoechst_3 |
| 650 | 4.438034 | Cells_Intensity_MaxIntensity_Ph_golgi |
| 651 | 4.468900 | Cells_Intensity_IntegratedIntensityEdge_Ph_golgi |
| 652 | 4.472008 | Cytoplasm_Texture_DifferenceEntropy_Ph_golgi_3 |
| 653 | 4.494323 | Cells_Texture_Contrast_Ph_golgi_3 |
| 654 | 4.504051 | Cells_Intensity_MaxIntensity_Hoechst |
| 655 | 4.507490 | Cells_Intensity_MeanIntensityEdge_Ph_golgi |
| 656 | 4.512059 | Cytoplasm_Texture_DifferenceEntropy_Syto_5 |
| 657 | 4.514544 | Cytoplasm_Texture_Contrast_ER_5 |
| 658 | 4.520896 | Cells_Intensity_IntegratedIntensityEdge_Mito |
| 659 | 4.555293 | Cytoplasm_Texture_DifferenceVariance_Syto_3 |
| 660 | 4.560677 | Cytoplasm_Intensity_MaxIntensity_Ph_golgi |
| 661 | 4.575821 | Cytoplasm_Texture_Gabor_ER_5 |
| 662 | 4.581451 | Cells_Intensity_IntegratedIntensity_Hoechst |
| 663 | 4.595537 | Cytoplasm_Texture_Entropy_Mito_5 |
| 664 | 4.611660 | Cytoplasm_Intensity_MeanIntensityEdge_Mito |
| 665 | 4.614905 | Cytoplasm_Intensity_MeanIntensityEdge_Ph_golgi |
| 666 | 4.618727 | Cytoplasm_Intensity_StdIntensity_Mito |
| 667 | 4.631114 | Cells_Intensity_MeanIntensity_Ph_golgi |
| 668 | 4.637781 | Cells_Texture_Contrast_ER_3 |
| 669 | 4.656937 | Cytoplasm_Texture_Contrast_Ph_golgi_3 |
| 670 | 4.666366 | Cells_Intensity_StdIntensity_Hoechst |
| 671 | 4.674492 | Cells_Texture_InverseDifferenceMoment_Hoechst_5 |
| 672 | 4.722999 | Cells_Texture_InfoMeas2_Syto_5 |
| 673 | 4.730819 | Cells_Texture_DifferenceVariance_Hoechst_3 |
| 674 | 4.742953 | Cytoplasm_Intensity_MaxIntensityEdge_Hoechst |
| 675 | 4.749007 | Cells_Intensity_IntegratedIntensityEdge_ER |
| 676 | 4.774223 | Cytoplasm_Intensity_MeanIntensity_Ph_golgi |
| 677 | 4.787916 | Cells_Texture_InfoMeas1_Syto_5 |
| 678 | 4.798734 | Cytoplasm_Intensity_IntegratedIntensityEdge_Hoechst |
| 679 | 4.815302 | Nuclei_Intensity_StdIntensityEdge_Mito |
| 680 | 4.864978 | Cells_RadialDistribution_FracAtD_Ph_golgi_2of4 |
| 681 | 4.873779 | Cytoplasm_Intensity_IntegratedIntensityEdge_Ph_golgi |
| 682 | 4.900000 | Cytoplasm_Texture_Entropy_Syto_5 |
| 683 | 4.905866 | Cells_Texture_Entropy_Syto_3 |
| 684 | 4.934433 | Cells_Texture_DifferenceEntropy_Syto_5 |
| 685 | 4.941268 | Cytoplasm_Intensity_IntegratedIntensityEdge_Mito |
| 686 | 4.951082 | Cells_Intensity_IntegratedIntensity_Ph_golgi |
| 687 | 4.958291 | Cells_Texture_InfoMeas2_Mito_3 |
| 688 | 4.996691 | Nuclei_Texture_Gabor_Mito_5 |
| 689 | 4.997323 | Cells_Intensity_MeanIntensity_Hoechst |
| 690 | 5.006480 | Cytoplasm_Intensity_IntegratedIntensityEdge_Syto |
| 691 | 5.009907 | Cells_Texture_DifferenceEntropy_Mito_5 |
| 692 | 5.018185 | Cytoplasm_Intensity_MeanIntensity_ER |
| 693 | 5.037413 | Cytoplasm_Texture_DifferenceVariance_Ph_golgi_5 |
| 694 | 5.046734 | Cells_RadialDistribution_MeanFrac_ER_2of4 |
| 695 | 5.050025 | Nuclei_Texture_Variance_Ph_golgi_5 |
| 696 | 5.051756 | Cells_Texture_Contrast_Hoechst_3 |
| 697 | 5.056535 | Cytoplasm_Intensity_IntegratedIntensity_Ph_golgi |
| 698 | 5.059797 | Nuclei_Intensity_MassDisplacement_Syto |
| 699 | 5.139087 | Nuclei_AreaShape_EulerNumber |
| 700 | 5.146062 | Cytoplasm_Intensity_IntegratedIntensityEdge_ER |
| 701 | 5.150765 | Cytoplasm_Intensity_MassDisplacement_Hoechst |
| 702 | 5.179528 | Cells_Texture_AngularSecondMoment_Ph_golgi_3 |
| 703 | 5.257004 | Cytoplasm_Intensity_StdIntensityEdge_ER |
| 704 | 5.273162 | Cells_Texture_Entropy_Ph_golgi_3 |
| 705 | 5.288610 | Cells_Texture_Variance_Mito_5 |
| 706 | 5.296489 | Cytoplasm_Intensity_StdIntensity_ER |
| 707 | 5.331748 | Cells_Texture_AngularSecondMoment_Ph_golgi_5 |
| 708 | 5.340785 | Cells_Texture_Contrast_ER_5 |
| 709 | 5.345618 | Nuclei_Intensity_StdIntensityEdge_Ph_golgi |
| 710 | 5.360155 | Cytoplasm_Texture_AngularSecondMoment_Ph_golgi_3 |
| 711 | 5.391710 | Cytoplasm_Texture_DifferenceVariance_Mito_3 |
| 712 | 5.484200 | Cells_Texture_InfoMeas1_Mito_5 |
| 713 | 5.509040 | Cytoplasm_Texture_InfoMeas2_Syto_3 |
| 714 | 5.539473 | Cells_RadialDistribution_MeanFrac_Mito_2of4 |
| 715 | 5.548158 | Cytoplasm_Intensity_MeanIntensityEdge_ER |
| 716 | 5.551519 | Cytoplasm_Intensity_MinIntensity_Mito |
| 717 | 5.600404 | Cells_Texture_InfoMeas2_Ph_golgi_5 |
| 718 | 5.731535 | Nuclei_Intensity_MaxIntensityEdge_Syto |
| 719 | 5.761153 | Nuclei_Texture_Contrast_ER_5 |
| 720 | 5.802816 | Cytoplasm_Texture_DifferenceEntropy_Mito_5 |
| 721 | 5.818294 | Cells_Intensity_StdIntensityEdge_ER |
| 722 | 5.852395 | Cells_Intensity_MaxIntensityEdge_Hoechst |
| 723 | 5.867571 | Cells_Intensity_MeanIntensity_Mito |
| 724 | 5.913877 | Cytoplasm_Intensity_MinIntensity_ER |
| 725 | 5.920084 | Nuclei_Intensity_MaxIntensityEdge_Ph_golgi |
| 726 | 5.968994 | Nuclei_Intensity_MinIntensity_Syto |
| 727 | 6.099036 | Nuclei_Intensity_IntegratedIntensity_Mito |
| 728 | 6.195079 | Nuclei_Intensity_MaxIntensity_Mito |
| 729 | 6.211634 | Cytoplasm_Intensity_StdIntensityEdge_Mito |
| 730 | 6.228060 | Nuclei_Texture_Entropy_Ph_golgi_5 |
| 731 | 6.280433 | Nuclei_Texture_Entropy_Syto_5 |
| 732 | 6.337528 | Cytoplasm_Texture_Contrast_Mito_3 |
| 733 | 6.339673 | Cells_Texture_Correlation_Mito_3 |
| 734 | 6.359404 | Nuclei_Texture_Gabor_ER_5 |
| 735 | 6.406862 | Cells_Texture_Gabor_ER_3 |
| 736 | 6.496183 | Nuclei_Intensity_IntegratedIntensity_Ph_golgi |
| 737 | 6.544563 | Nuclei_Intensity_MaxIntensity_Syto |
| 738 | 6.580578 | Cells_Texture_Gabor_Ph_golgi_5 |
| 739 | 6.637890 | Cytoplasm_Texture_Correlation_Hoechst_5 |
| 740 | 6.653605 | Nuclei_Intensity_MassDisplacement_Ph_golgi |
| 741 | 6.664844 | Nuclei_Intensity_MeanIntensityEdge_Mito |
| 742 | 6.674214 | Cells_Texture_Contrast_Syto_5 |
| 743 | 6.686500 | Nuclei_Intensity_MeanIntensity_Mito |
| 744 | 6.748016 | Cytoplasm_Texture_InfoMeas2_Ph_golgi_5 |
| 745 | 6.813209 | Nuclei_Intensity_StdIntensity_Mito |
| 746 | 6.848803 | Cells_Texture_DifferenceVariance_Mito_3 |
| 747 | 6.859919 | Cytoplasm_Texture_AngularSecondMoment_Syto_5 |
| 748 | 6.900314 | Cytoplasm_Texture_Contrast_Syto_5 |
| 749 | 6.966251 | Nuclei_Intensity_StdIntensity_Ph_golgi |
| 750 | 7.047597 | Cytoplasm_Intensity_StdIntensityEdge_Hoechst |
| 751 | 7.085544 | Cytoplasm_Intensity_MeanIntensity_Hoechst |
| 752 | 7.156601 | Cytoplasm_Texture_DifferenceVariance_Hoechst_5 |
| 753 | 7.168886 | Cells_Texture_InfoMeas1_Syto_3 |
| 754 | 7.182610 | Nuclei_Intensity_MeanIntensity_Ph_golgi |
| 755 | 7.283436 | Cells_Intensity_MeanIntensityEdge_Mito |
| 756 | 7.287544 | Cells_Intensity_MeanIntensity_ER |
| 757 | 7.299877 | Cells_Intensity_StdIntensityEdge_Hoechst |
| 758 | 7.300276 | Cytoplasm_Intensity_MaxIntensity_Hoechst |
| 759 | 7.423608 | Cells_Intensity_StdIntensityEdge_Mito |
| 760 | 7.519817 | Cells_Intensity_MeanIntensityEdge_ER |
| 761 | 7.558575 | Cytoplasm_Texture_AngularSecondMoment_Mito_5 |
| 762 | 7.608856 | Nuclei_Intensity_MeanIntensityEdge_Ph_golgi |
| 763 | 7.612829 | Cells_Texture_Contrast_Mito_5 |
| 764 | 7.627925 | Cytoplasm_Intensity_MeanIntensityEdge_Hoechst |
| 765 | 7.654839 | Cells_Texture_Contrast_Mito_3 |
| 766 | 7.681289 | Cells_Texture_DifferenceVariance_Hoechst_5 |
| 767 | 7.683322 | Cells_Intensity_MeanIntensityEdge_Hoechst |
| 768 | 7.803564 | Cells_Texture_AngularSecondMoment_Mito_5 |
| 769 | 7.812403 | Nuclei_Intensity_IntegratedIntensity_Syto |
| 770 | 7.813252 | Nuclei_Intensity_StdIntensityEdge_Syto |
| 771 | 7.955655 | Cytoplasm_Texture_Contrast_Hoechst_5 |
| 772 | 7.985183 | Cells_Intensity_StdIntensity_Mito |
| 773 | 8.024409 | Nuclei_AreaShape_Extent |
| 774 | 8.073721 | Nuclei_Intensity_MeanIntensityEdge_Syto |
| 775 | 8.094091 | Cytoplasm_Texture_Contrast_Mito_5 |
| 776 | 8.153954 | Nuclei_Intensity_StdIntensity_Syto |
| 777 | 8.202199 | Cells_Intensity_StdIntensity_ER |
| 778 | 8.223805 | Cytoplasm_Intensity_StdIntensity_Hoechst |
| 779 | 8.376926 | Cells_Texture_InfoMeas2_Hoechst_5 |
| 780 | 8.396168 | Cells_Texture_AngularSecondMoment_Syto_5 |
| 781 | 8.475617 | Cytoplasm_Intensity_MinIntensity_Hoechst |
| 782 | 8.705260 | Nuclei_Intensity_MeanIntensity_Syto |
| 783 | 8.901477 | Cells_Texture_Contrast_Hoechst_5 |
| 784 | 9.873469 | Cells_Texture_Contrast_Syto_3 |
| 785 | 10.219001 | Cells_Texture_Correlation_Hoechst_5 |
| 786 | 10.554727 | Cells_Texture_Gabor_ER_5 |
| 787 | 11.998219 | Cells_Texture_InfoMeas1_Ph_golgi_5 |
| 788 | 12.641428 | Nuclei_AreaShape_Orientation |
| 789 | 13.168964 | Nuclei_AreaShape_Perimeter |
| 790 | 13.748158 | Nuclei_Intensity_MinIntensityEdge_Ph_golgi |
| 791 | 13.809637 | Cells_Texture_Gabor_Hoechst_5 |
| 792 | 14.659282 | Nuclei_Intensity_MedianIntensity_Ph_golgi |
| 793 | 15.022761 | Cytoplasm_Texture_InverseDifferenceMoment_Syto_3 |
| 794 | 15.250430 | Cytoplasm_Texture_InverseDifferenceMoment_Ph_golgi_5 |
| 795 | 17.183992 | Cells_Texture_Entropy_Mito_5 |
| 796 | 17.339777 | Nuclei_Intensity_MaxIntensity_Ph_golgi |
| 797 | 18.533604 | Cells_Texture_Entropy_Syto_5 |
| 798 | 19.349377 | Cells_Texture_Entropy_Ph_golgi_5 |
| 799 | 23.458262 | Cells_Texture_Contrast_Ph_golgi_5 |
| 800 | 132.682428 | Cells_Texture_Variance_Ph_golgi_5 |
