# Supplementary material for: Multiplex Cytological Profiling Assay to Measure Diverse Cellular States
Source: PLoS One. 2013 Dec 2;8(12):e80999. doi: 10.1371/journal.pone.0080999 (PMC3847047; doi:10.1371/journal.pone.0080999)
Supplement: Table S6 — Compounds that were annotated. (DOCX) [file pone.0080999.s014.docx]

**Table S6:** Compounds that were annotated.

| Compound name | Concentration [µM] | Source | Broad ID | Structure |
| --- | --- | --- | --- | --- |
| (+ -)-BACLOFEN | 5.00 | Biomol International Inc. | BRD-A84174873 | 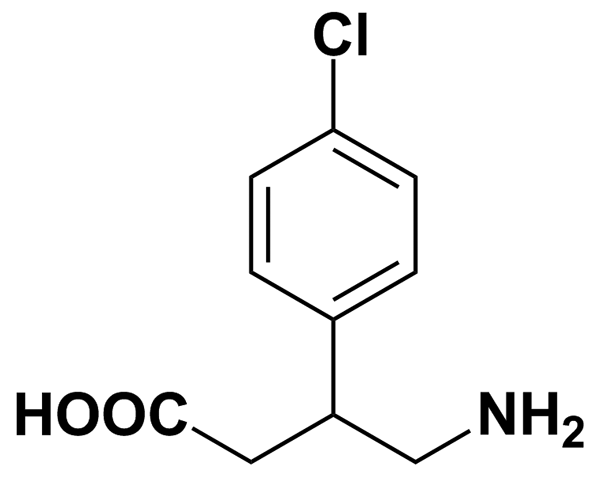 |
| (D,L)-TETRAHYDROBERBERINE | 2.95 | Prestwick Chemical Inc. | BRD-A69950438 | 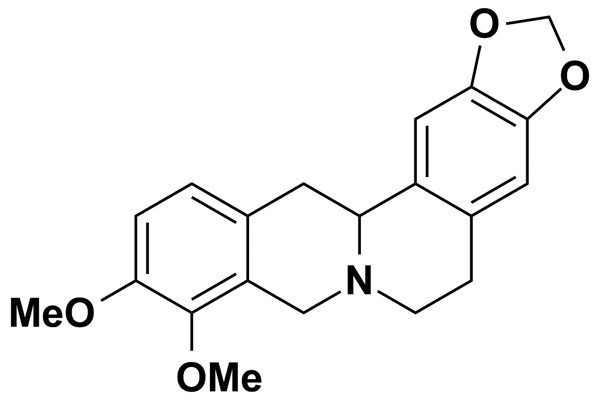 |
| (RS)-(+ -)-SULPIRIDE | 5.00 | Biomol International Inc. | BRD-A55272860 | 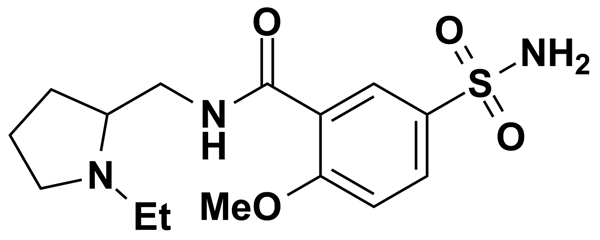 |
| 1,25-DIHYDROXYVITAMIN D3 | 0.50 | Biomol International Inc. | BRD-K27316855 | 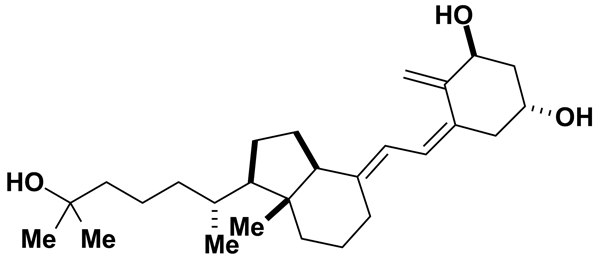 |
| 1,3-DIPROPYL-8-CYCLOPENTYLXANTHINE [DPCPX] | 5.00 | MicroSource Discovery Systems Inc. | BRD-K01824921 | 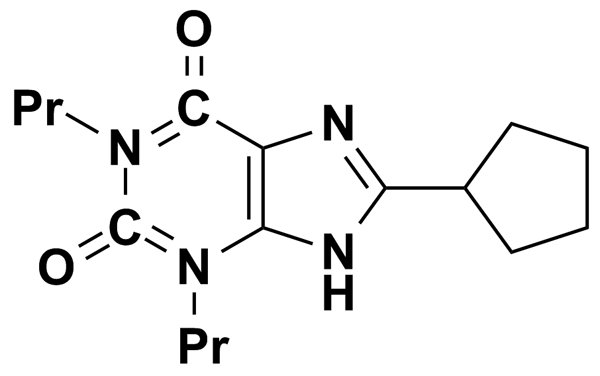 |
| 1-(3-TRIFLUOROMETHYL) PHENYLPIPERAZINE MONOHYDROCHLORIDE | 5.00 | Biomol International Inc. | BRD-K94887716 | 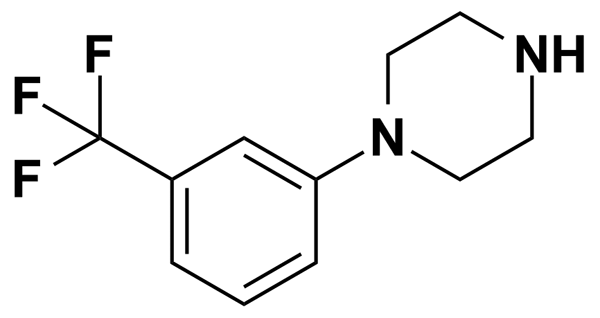 |
| 1-PHENYLBIGUANIDE HYDROCHLORIDE | 5.00 | MicroSource Discovery Systems Inc. | BRD-K31491153 | 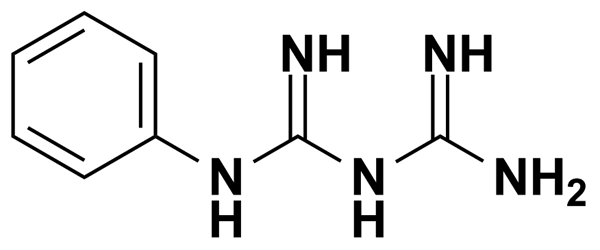 |
| 13-CIS RETINOIC ACID | 0.50 | Biomol International Inc. | BRD-K76723084 | 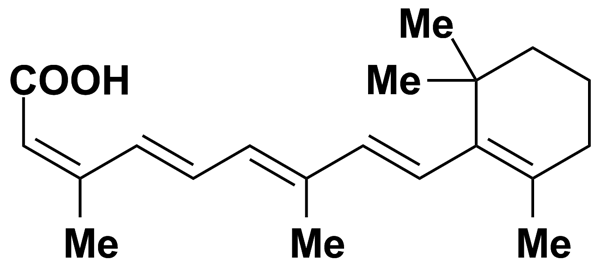 |
| 17-PHENYL-TRINOR-PGE2 | 0.50 | Biomol International Inc. | BRD-K90214371 | 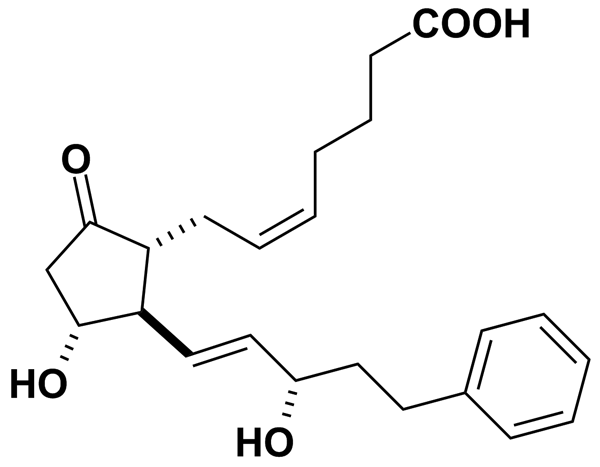 |
| 1R,9S-HYDRASTINE | 5.00 | MicroSource Discovery Systems Inc. | BRD-K77435797 | 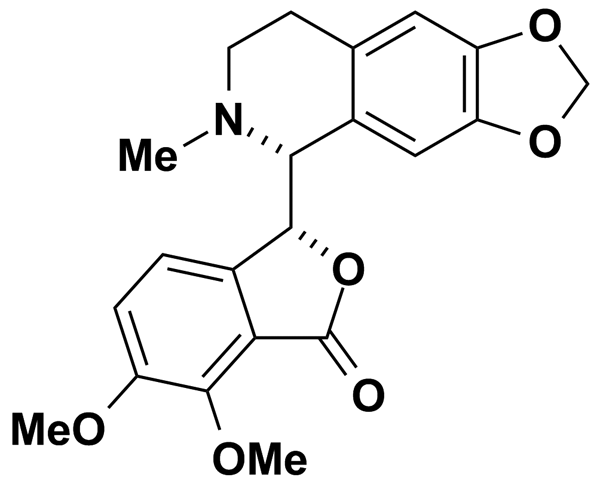 |
| 2,3-DIOXO-6-NITRO-1,2,3,4-TETRAHYDROBENZO[F]QUINOXALINE-7-SULFONAMIDE | 5.00 | Biomol International Inc. | BRD-K11796549 | 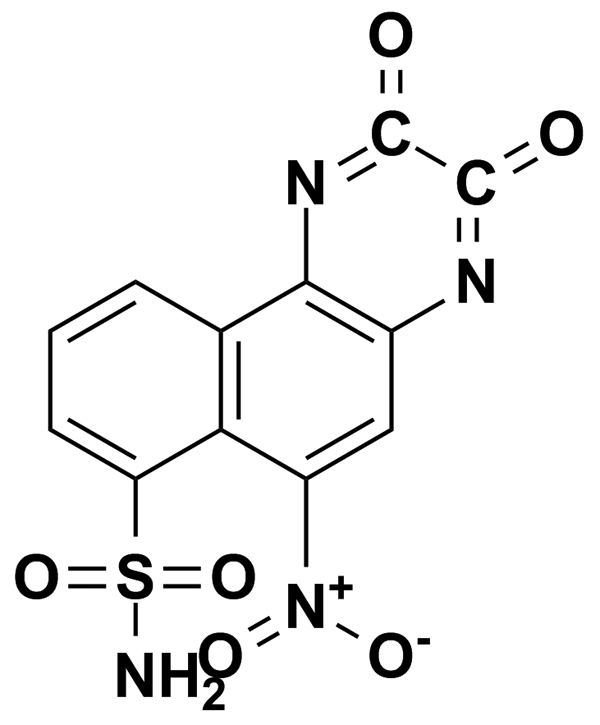 |
| 2,4,5-TRICHLOROPHENOXYACETIC ACID | 5.00 | MicroSource Discovery Systems Inc. | BRD-K92860374 | 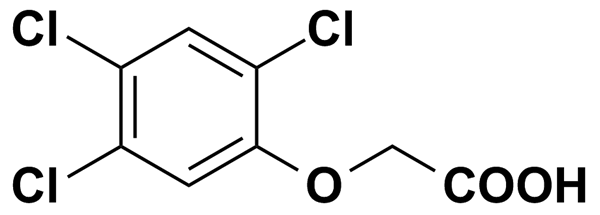 |
| 2,4-DICHLOROPHENOXYACETIC ACID | 5.00 | MicroSource Discovery Systems Inc. | BRD-K01473791 | 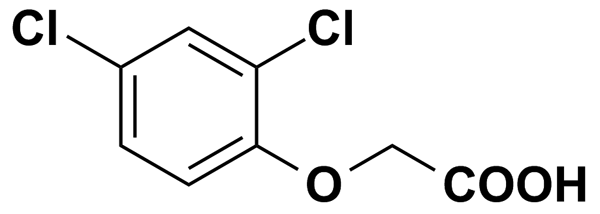 |
| 2,5-DITERTBUTYLHYDROQUINONE | 11.24 | Biomol International Inc. | BRD-K95603879 | 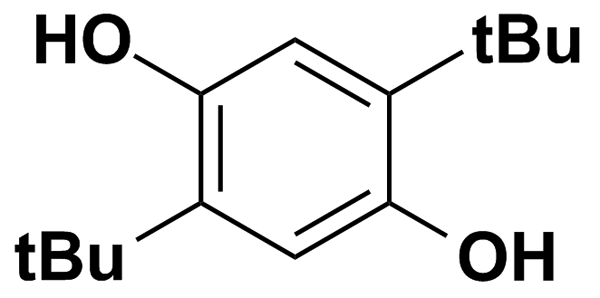 |
| 2-ARACHIDONOYLGLYCEROL | 0.50 | Biomol International Inc. | BRD-K71198913 | 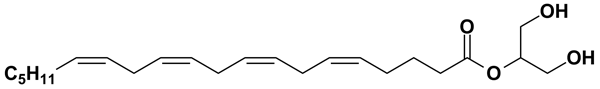 |
| 24,25-DIHYDROXYVITAMIN D3 | 0.50 | Biomol International Inc. | BRD-K02965577 | 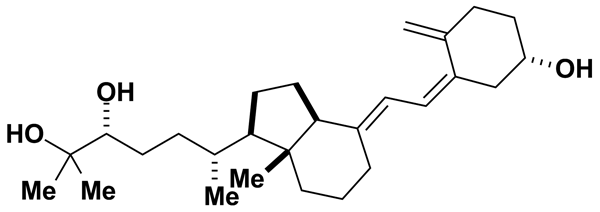 |
| 3,4-DICHLOROISOCOUMARIN | 11.63 | Biomol International Inc. | BRD-K23704908 | 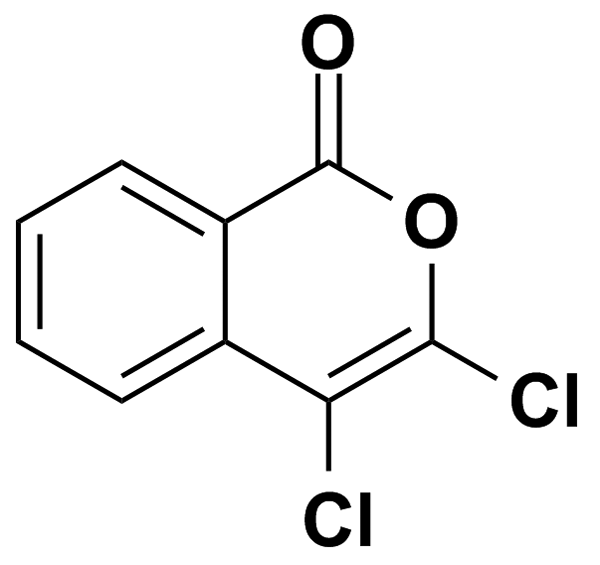 |
| 3-HYDROXYBENZYLHYDRAZINE DIHYDROCHLORIDE | 5.00 | MicroSource Discovery Systems Inc. | BRD-K66416915 | 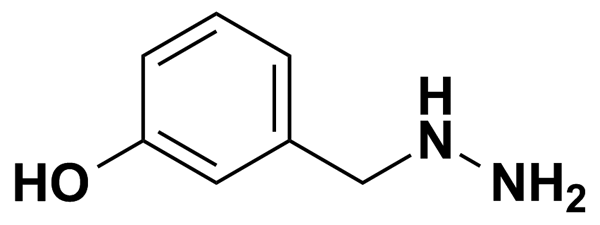 |
| 3-HYDROXYMETHYL-BETA-CARBOLINE | 5.00 | Biomol International Inc. | BRD-K69585439 | 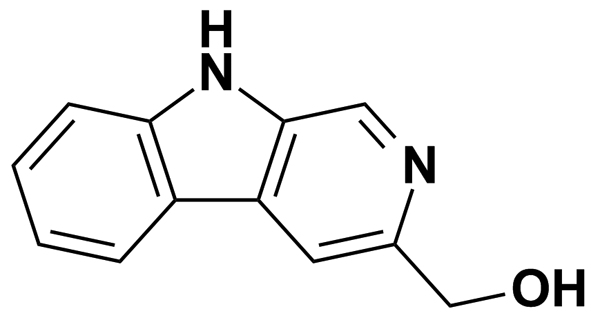 |
| 3-METHYLCHOLANTHRENE | 5.00 | MicroSource Discovery Systems Inc. | BRD-K61463582 | 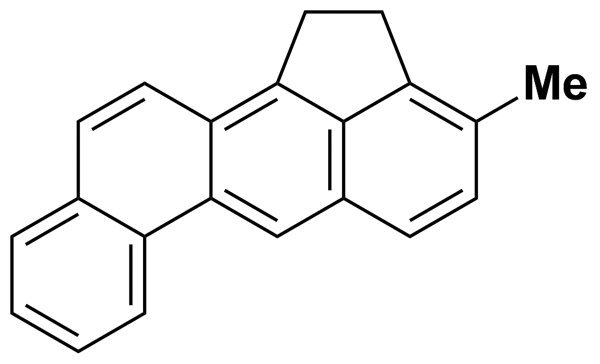 |
| 6-FORMYLINDOLO [3,2-B] CARBAZOLE | 0.50 | Biomol International Inc. | BRD-K00184207 | 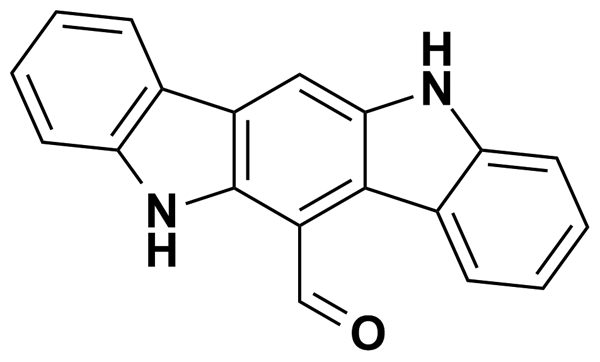 |
| 6-FURFURYLAMINOPURINE | 4.65 | Prestwick Chemical Inc. | BRD-K65667145 | 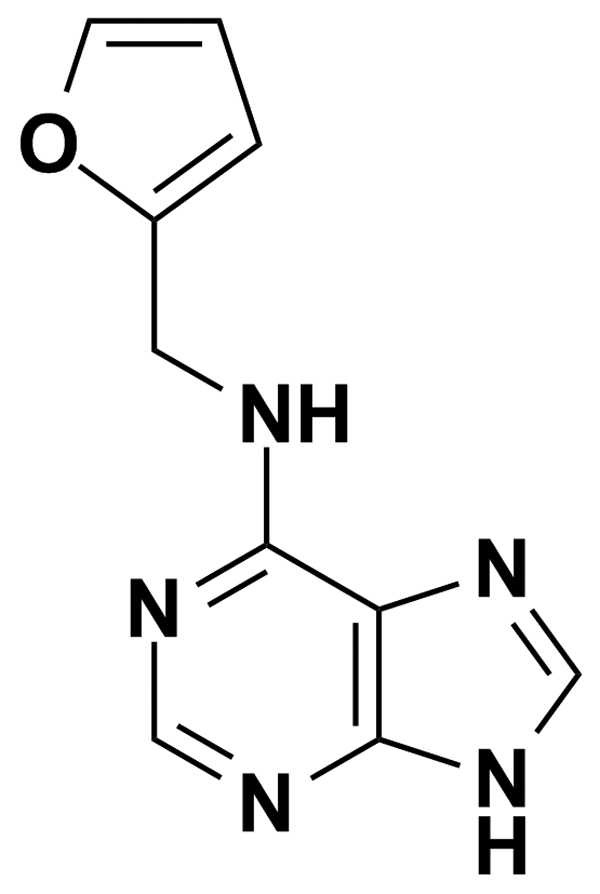 |
| 7,8-DIHYDROXYFLAVONE | 5.00 | MicroSource Discovery Systems Inc. | BRD-K49535716 | 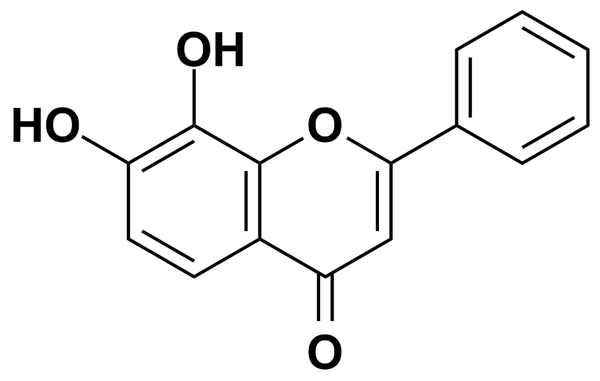 |
| 7-CHLORO-3-METHYL-3,4-DIHYDRO-2H-1,2,4-BENZOTHIADIAZINE-S,S-DIOXIDE | 5.00 | Biomol International Inc. | BRD-A14344385 | 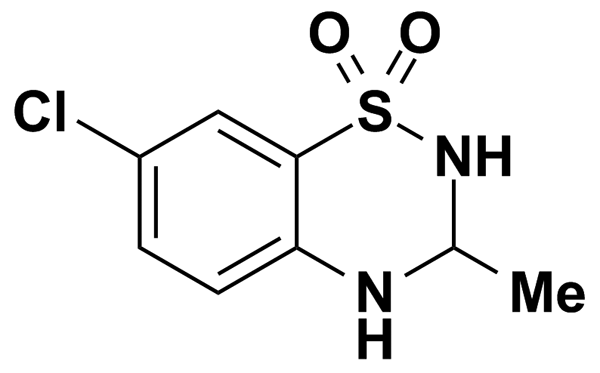 |
| 7-NITROINDAZOLE | 5.00 | MicroSource Discovery Systems Inc. | BRD-K04430056 | 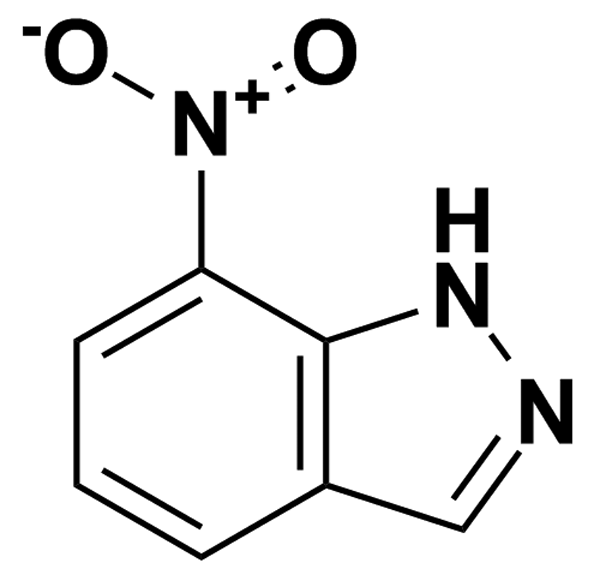 |
| 8-CYCLOPENTYLTHEOPHYLLINE | 5.00 | MicroSource Discovery Systems Inc. | BRD-K38347298 | 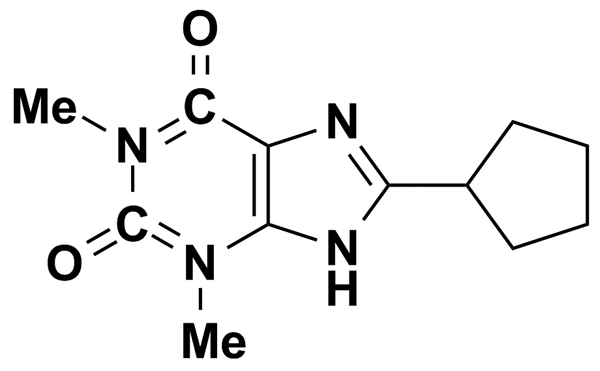 |
| 8-METHOXYMETHYL-IBMX | 9.39 | Biomol International Inc. | BRD-K56077740 | 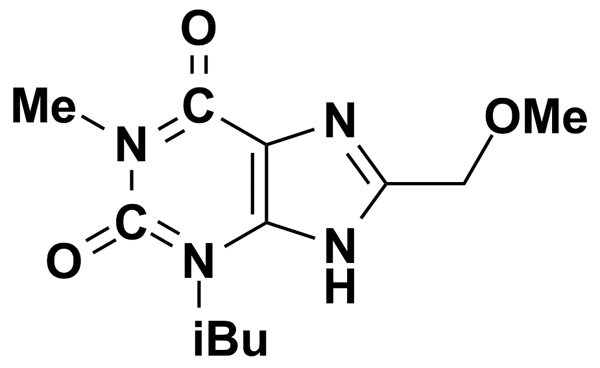 |
| A-23187 | 4.77 | Biomol International Inc. | BRD-A19134330 | 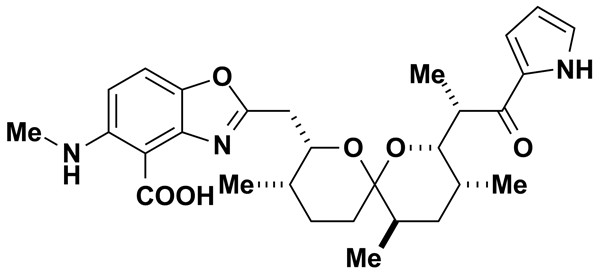 |
| A-3 | 8.78 | Biomol International Inc. | BRD-K51215422 | 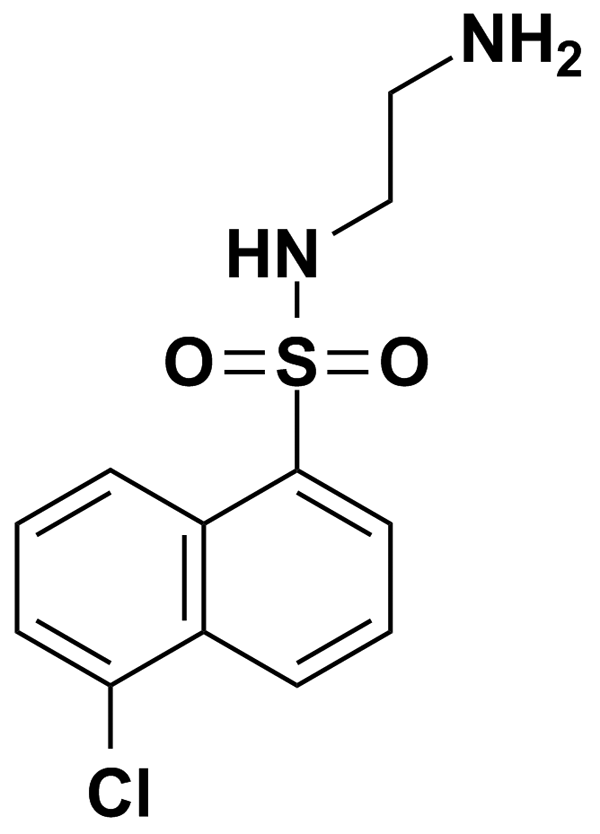 |
| ACACETIN | 3.52 | Prestwick Chemical Inc. | BRD-K77685744 | 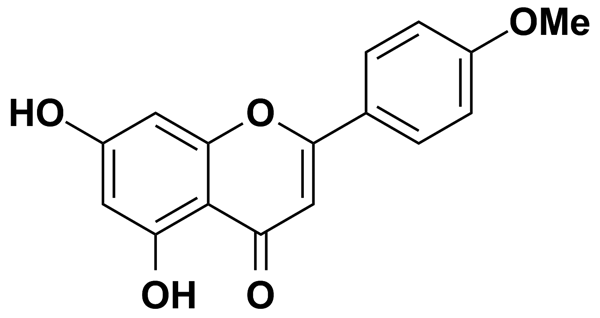 |
| ACEBUTOLOL HYDROCHLORIDE | 2.68 | Prestwick Chemical Inc. | BRD-A29260609 | 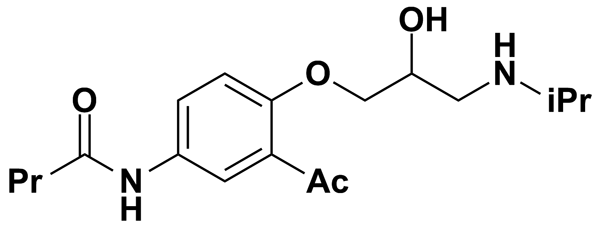 |
| ACECLIDINE HYDROCHLORIDE | 5.00 | MicroSource Discovery Systems Inc. | BRD-A32673558 | 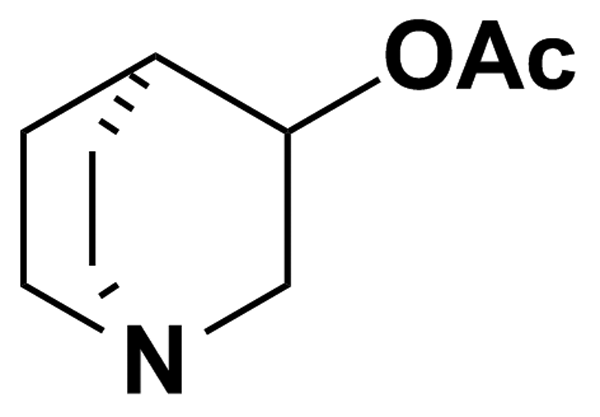 |
| ACEMETACIN | 2.40 | Prestwick Chemical Inc. | BRD-K67563174 | 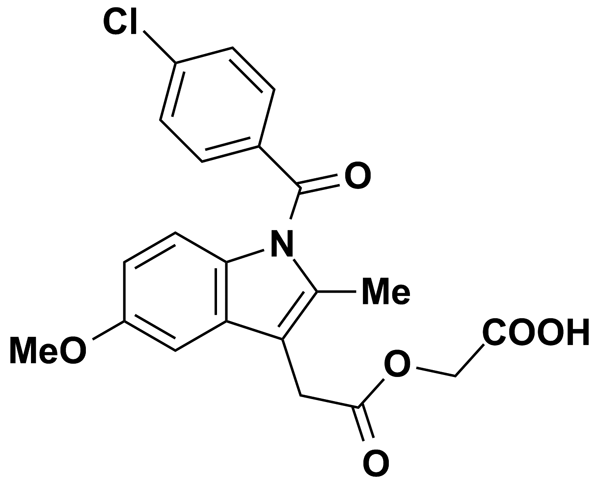 |
| ACETOCHLOR | 5.00 | MicroSource Discovery Systems Inc. | BRD-K16652251 | 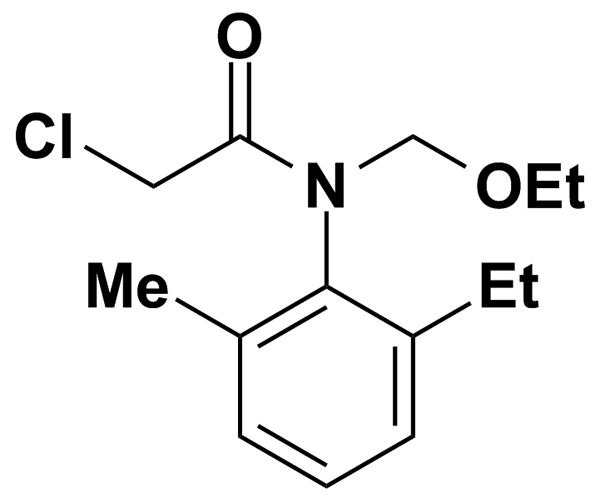 |
| ACETYL (N)-S-FARNESYL-L-CYSTEINE | 6.80 | Biomol International Inc. | BRD-K79437791 | 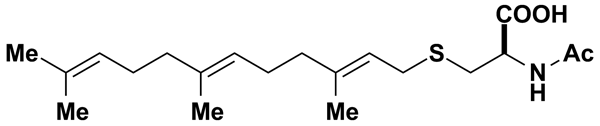 |
| ACETYLTRYPTOPHANAMIDE | 5.00 | MicroSource Discovery Systems Inc. | BRD-K10171338 | 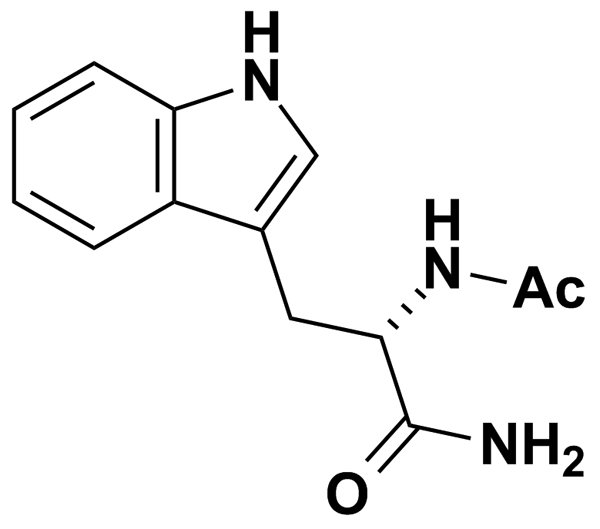 |
| ACTINONIN | 5.00 | MicroSource Discovery Systems Inc. | BRD-K24621118 | 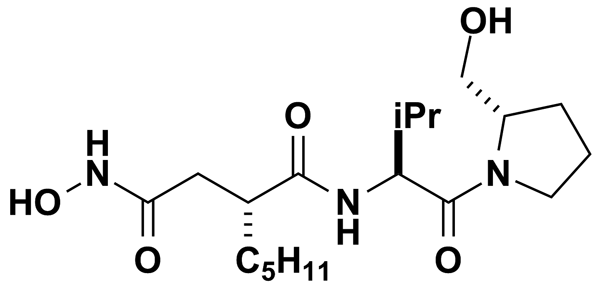 |
| ADIPHENINE HYDROCHLORIDE | 2.87 | Prestwick Chemical Inc. | BRD-K60907894 | 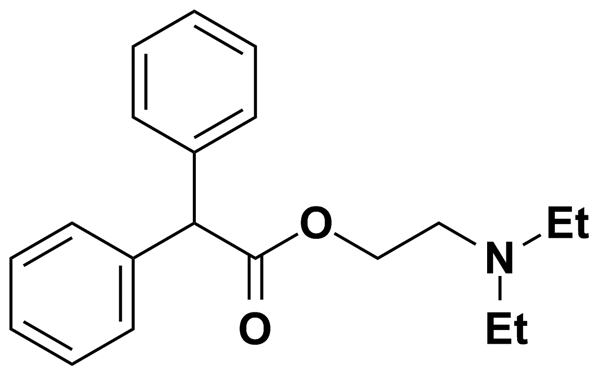 |
| AESCULIN | 5.00 | MicroSource Discovery Systems Inc. | BRD-K51742987 | 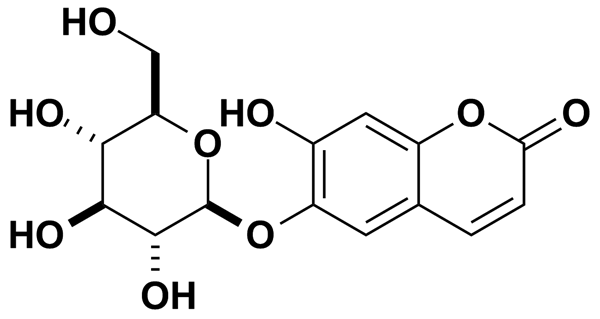 |
| AG-1296 | 9.39 | Biomol International Inc. | BRD-K76064317 | 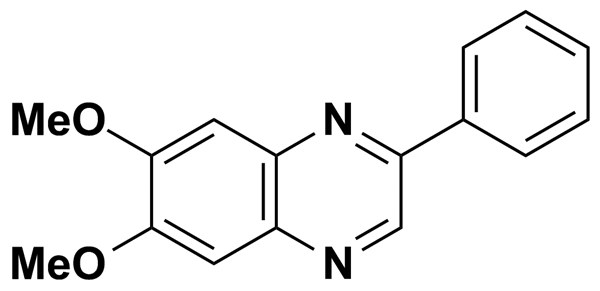 |
| AG-370 | 9.64 | Biomol International Inc. | BRD-K33204703 | 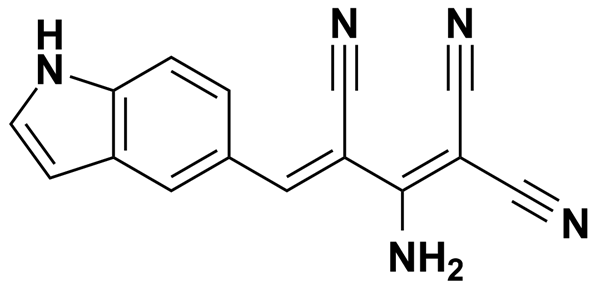 |
| AG-490 | 8.49 | Biomol International Inc. | BRD-K47105409 | 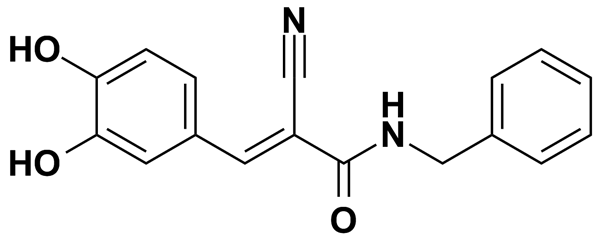 |
| AG-879 | 7.90 | Biomol International Inc. | BRD-K59469039 | 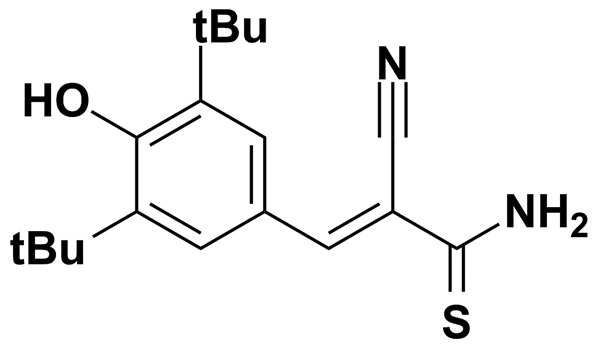 |
| AG1478 | 7.10 | Biomol International Inc. | BRD-K68336408 | 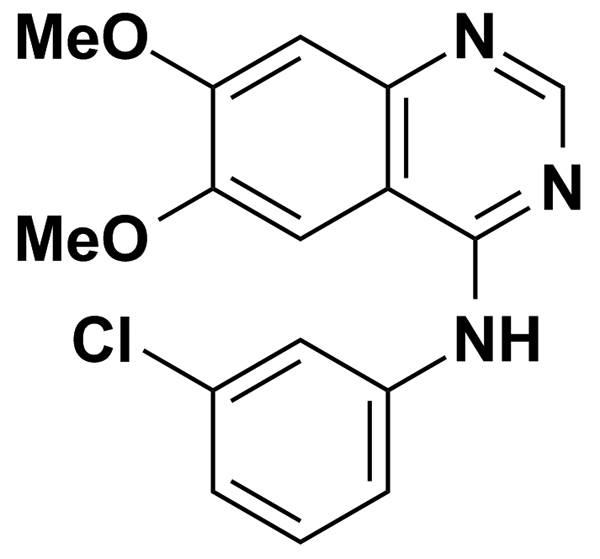 |
| AGGC | 0.50 | Biomol International Inc. | BRD-K75181824 | 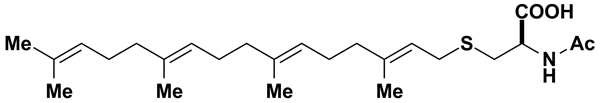 |
| ALACHLOR | 5.00 | MicroSource Discovery Systems Inc. | BRD-K02548315 | 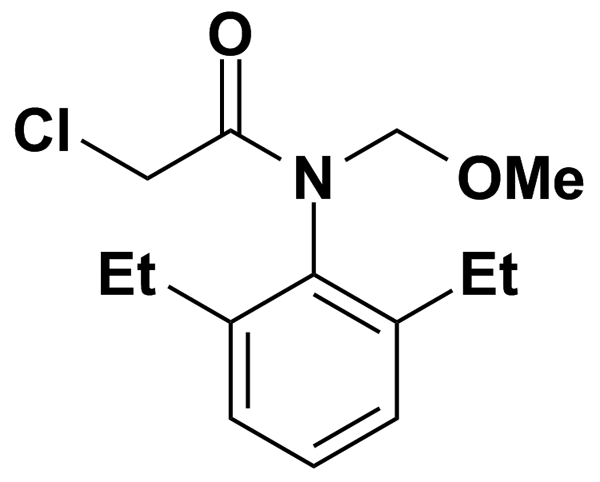 |
| ALMOTRIPTAN | 5.00 | MicroSource Discovery Systems Inc. | BRD-K67601717 | 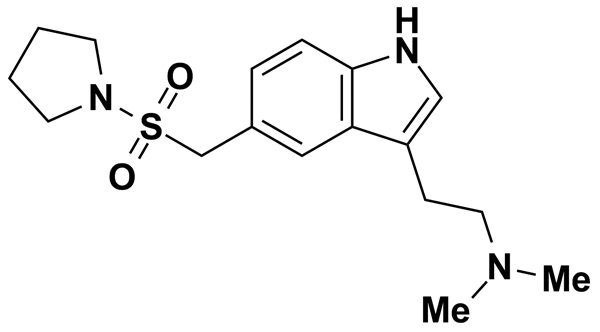 |
| ALPHA-CYANO-4-HYDROXYCINNAMIC ACID | 5.00 | MicroSource Discovery Systems Inc. | BRD-K60302405 | 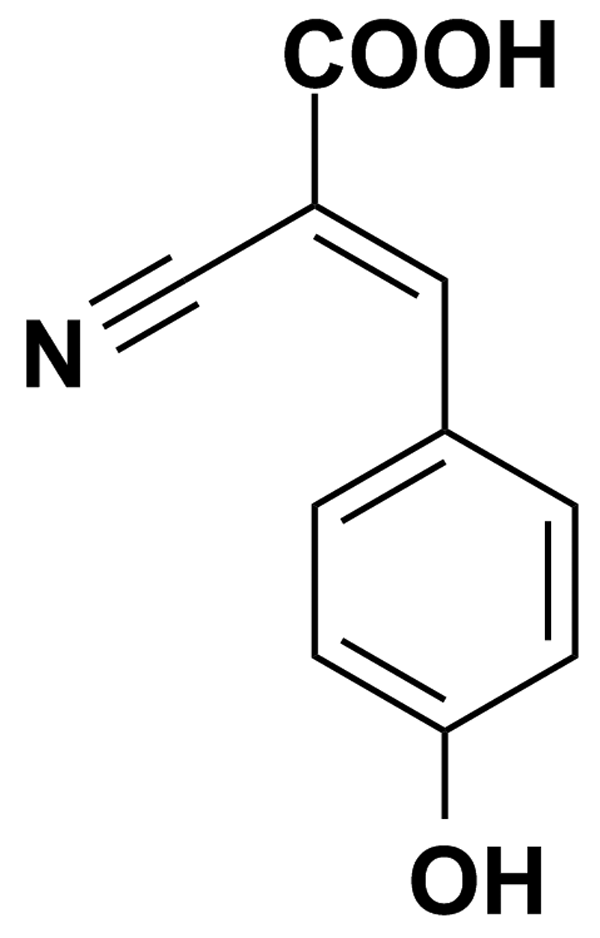 |
| ALPRENOLOL HYDROCHLORIDE | 3.50 | Prestwick Chemical Inc. | BRD-A00993607 | 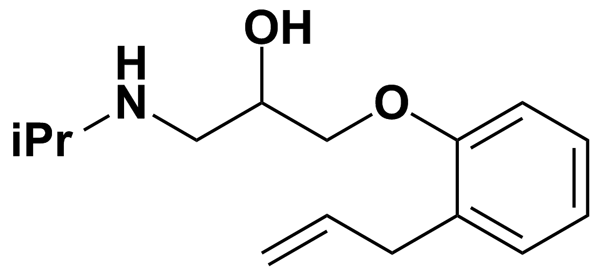 |
| ALTHIAZIDE | 2.60 | Prestwick Chemical Inc. | BRD-A56675431 | 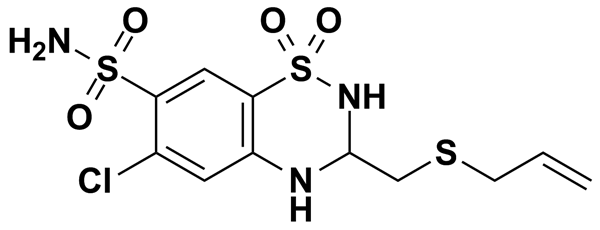 |
| ALVERINE CITRATE SALT | 2.11 | Prestwick Chemical Inc. | BRD-K89055274 | 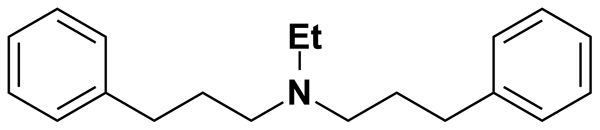 |
| AM 92016 | 5.59 | Biomol International Inc. | BRD-A11813248 | 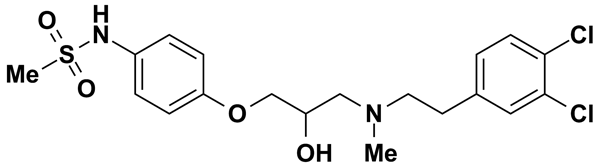 |
| AM-251 | 0.50 | Biomol International Inc. | BRD-K92000912 | 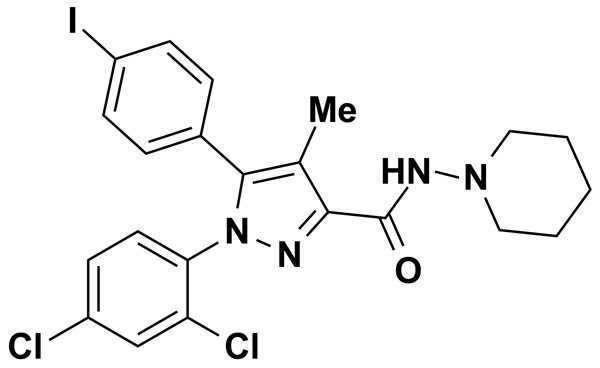 |
| AM-580 | 0.50 | Biomol International Inc. | BRD-K06854232 | 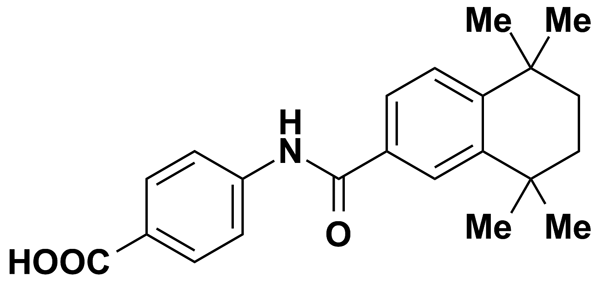 |
| AMCINONIDE | 5.00 | MicroSource Discovery Systems Inc. | BRD-A36010170 | 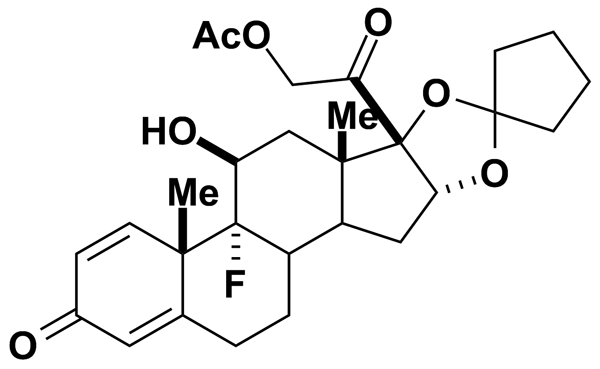 |
| AMILORIDE HYDROCHLORIDE DIHYDRATE | 3.31 | Prestwick Chemical Inc. | BRD-K97181089 | 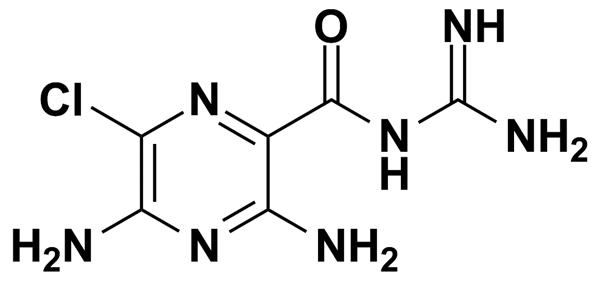 |
| AMITRYPTILINE HYDROCHLORIDE | 3.19 | Prestwick Chemical Inc. | BRD-K53737926 | 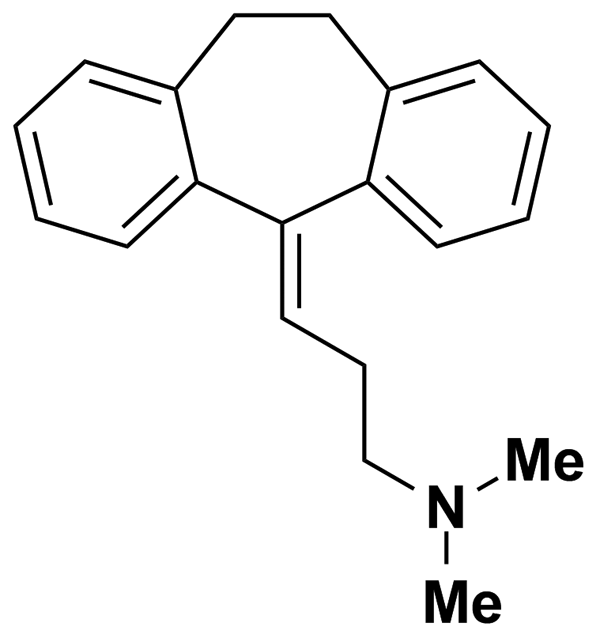 |
| AMOXAPINE | 3.19 | Prestwick Chemical Inc. | BRD-K02265150 | 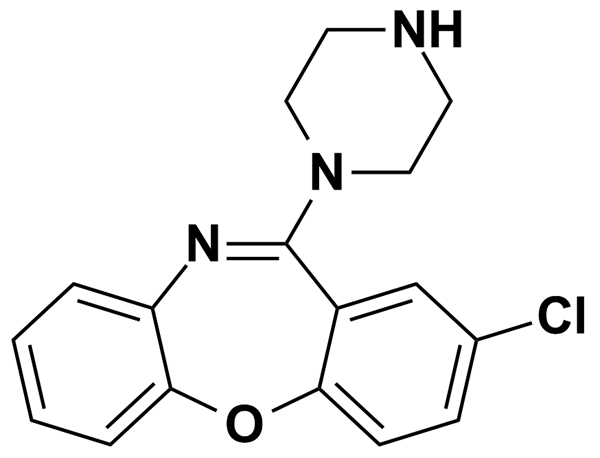 |
| AMPEROZIDE | 5.00 | Biomol International Inc. | BRD-K52397688 | 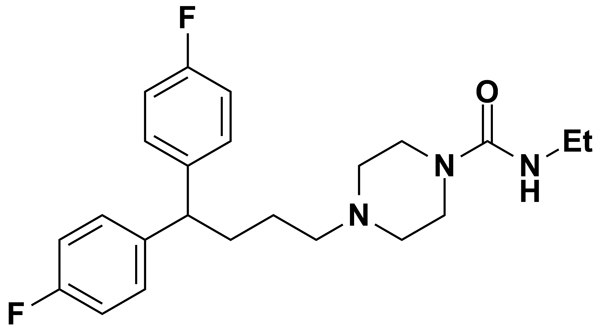 |
| ANANDAMIDE (20:3,N-6) | 0.50 | Biomol International Inc. | BRD-K78280988 | 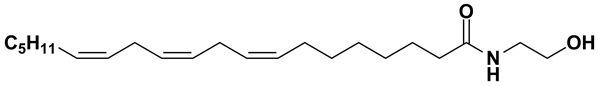 |
| ANANDAMIDE (22:4,N-6) | 0.50 | Biomol International Inc. | BRD-K30199352 | 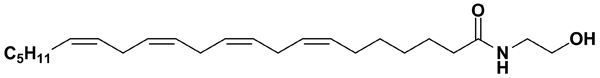 |
| ANISOMYCIN | 9.42 | Biomol International Inc. | BRD-K91370081 | 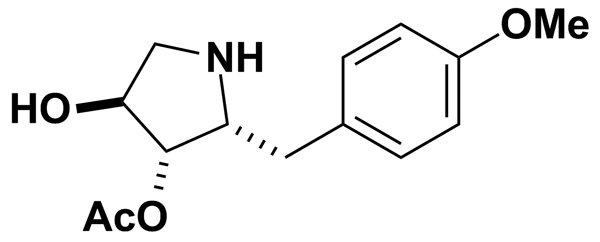 |
| ANTAZOLINE HYDROCHLORIDE | 3.31 | Prestwick Chemical Inc. | BRD-K99300445 | 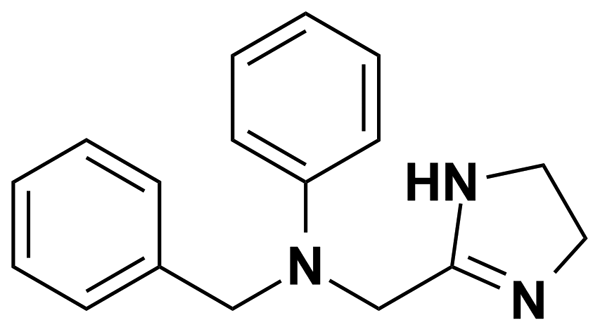 |
| ANTIPYRINE | 5.31 | Prestwick Chemical Inc. | BRD-K46937689 | 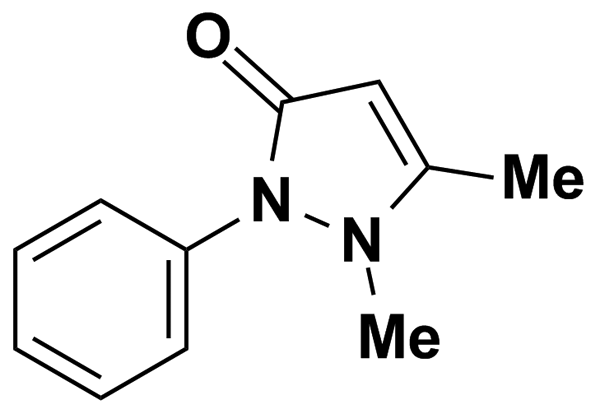 |
| ARACHIDONAMIDE | 0.50 | Biomol International Inc. | BRD-K29555132 | 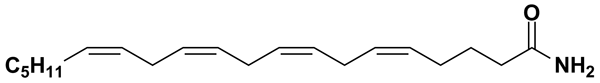 |
| ARECOLINE HYDROBROMIDE | 4.24 | Prestwick Chemical Inc. | BRD-K88646909 | 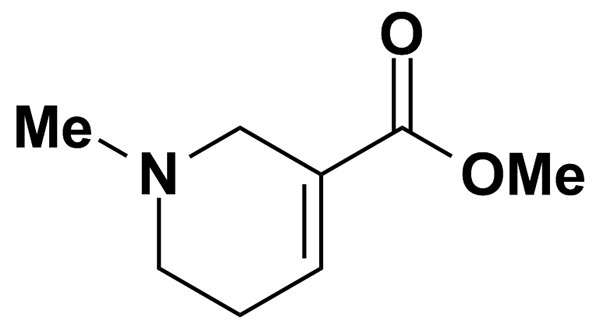 |
| ASTEMIZOLE | 2.18 | Prestwick Chemical Inc. | BRD-K37249724 | 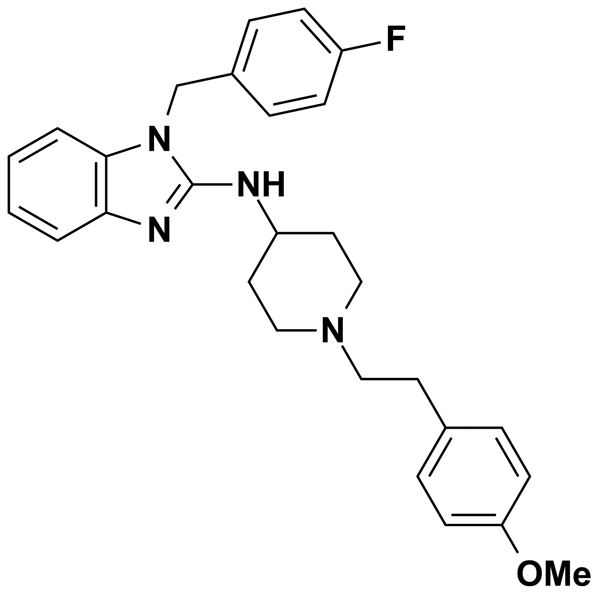 |
| ATENOLOL | 5.00 | MicroSource Discovery Systems Inc. | BRD-A20239487 | 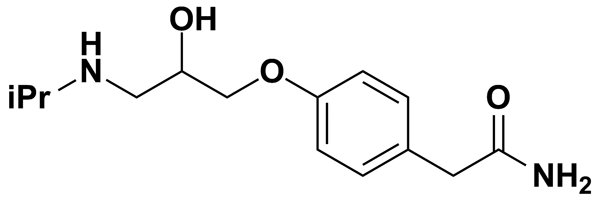 |
| ATOVAQUONE | 5.00 | MicroSource Discovery Systems Inc. | BRD-A19795905 | 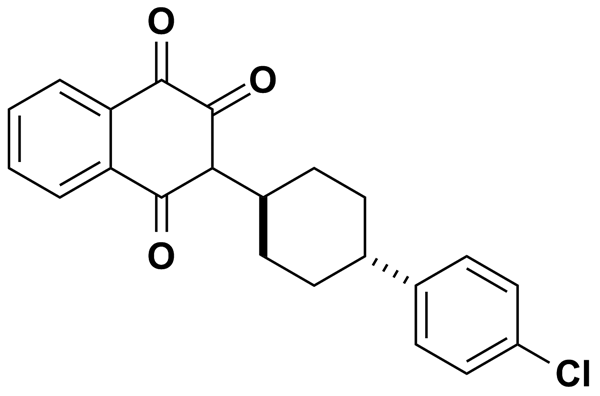 |
| AZATHIOPRINE | 3.61 | Prestwick Chemical Inc. | BRD-K32821942 | 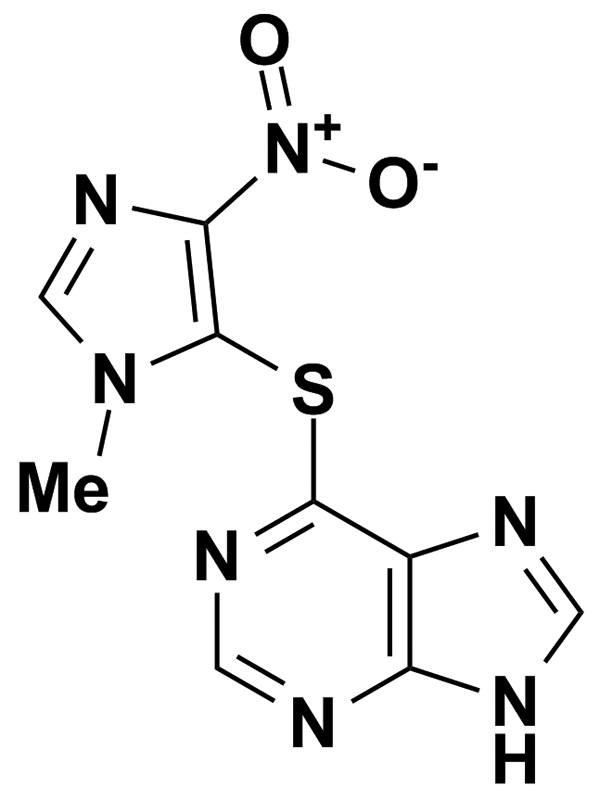 |
| AZINPHOS METHYL | 5.00 | MicroSource Discovery Systems Inc. | BRD-K21893543 | 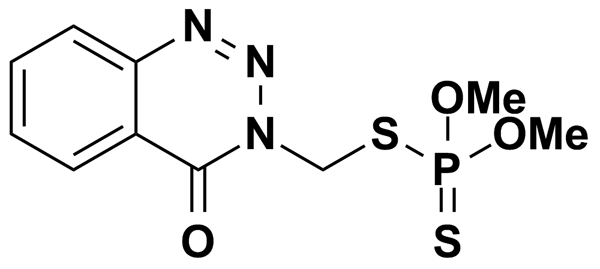 |
| B581 | 5.31 | Biomol International Inc. | BRD-K04877770 | 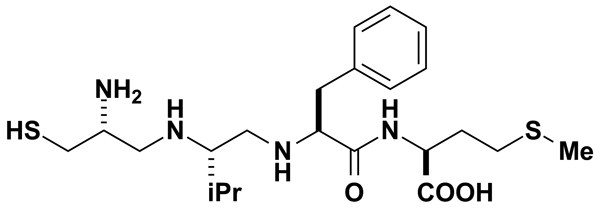 |
| BAPTA-AM | 3.27 | Biomol International Inc. | BRD-K40919711 | 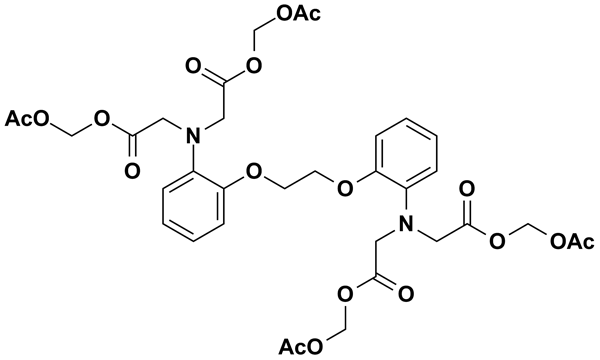 |
| BAY 11-7082 | 12.06 | Biomol International Inc. | BRD-K15025317 | 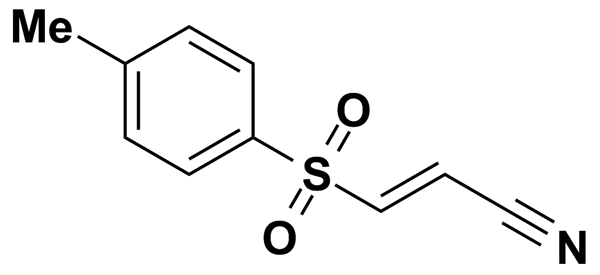 |
| BAY K-8644 | 7.02 | Biomol International Inc. | BRD-A05457250 | 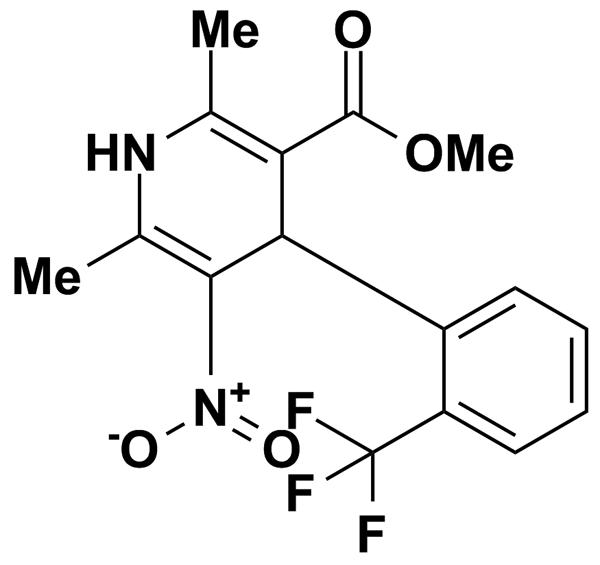 |
| BENDROFLUMETHIAZIDE | 2.37 | Prestwick Chemical Inc. | BRD-A80017228 | 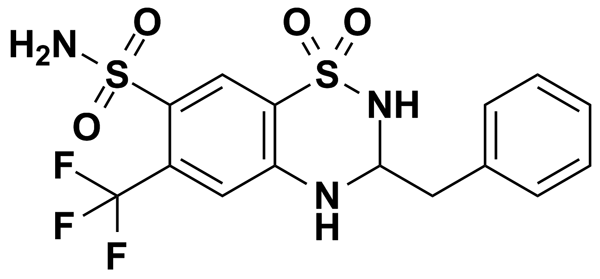 |
| BENFLUOREX HYDROCHLORIDE | 2.58 | Prestwick Chemical Inc. | BRD-A22305049 | 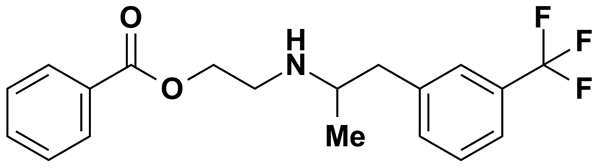 |
| BENTAZON | 5.00 | MicroSource Discovery Systems Inc. | BRD-K33986892 | 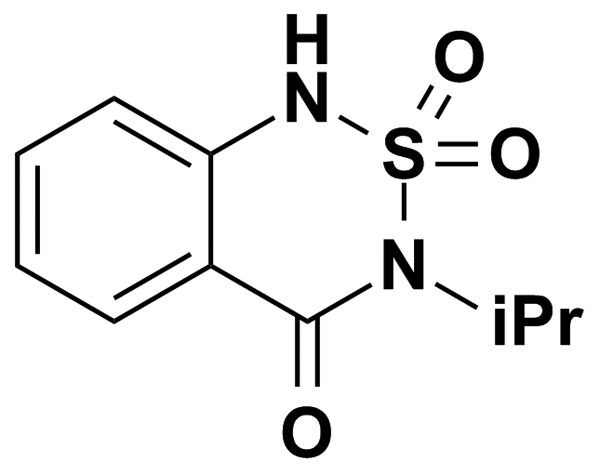 |
| BENZOCAINE | 6.05 | Prestwick Chemical Inc. | BRD-K75466013 | 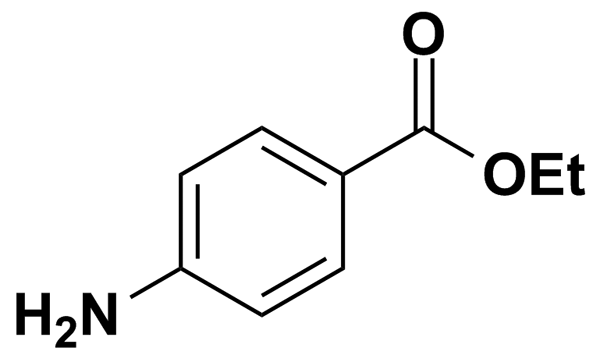 |
| BENZYL PENICILLIN POTASSIUM | 5.00 | MicroSource Discovery Systems Inc. | BRD-K55191674 | 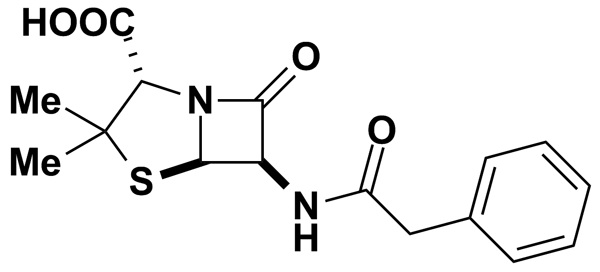 |
| BENZYLPENICILLIN SODIUM | 2.80 | Prestwick Chemical Inc. | BRD-K55191674 | 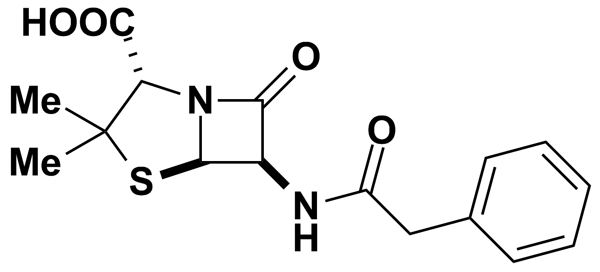 |
| BERBERINE CHLORIDE | 2.68 | Prestwick Chemical Inc. | BRD-K14796088 | 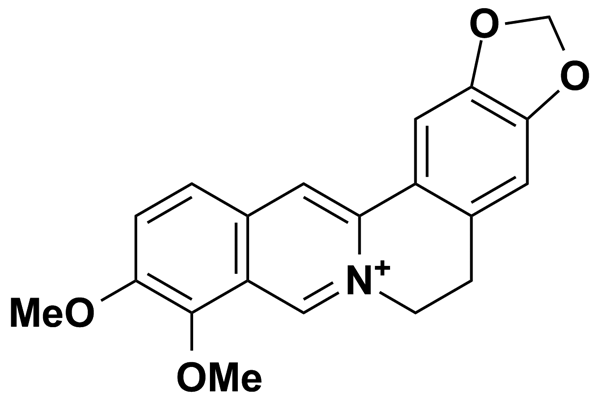 |
| BERGAPTEN | 5.00 | MicroSource Discovery Systems Inc. | BRD-K12968785 | 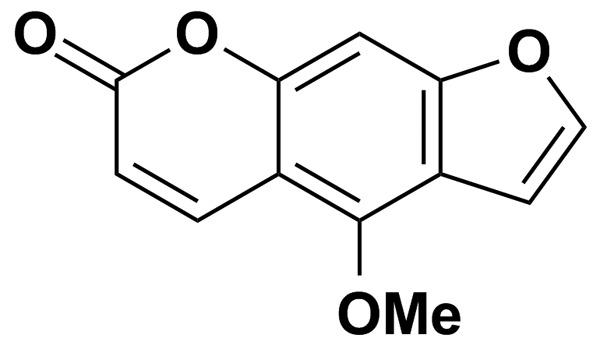 |
| BESTATIN | 8.11 | Biomol International Inc. | BRD-K59574735 | 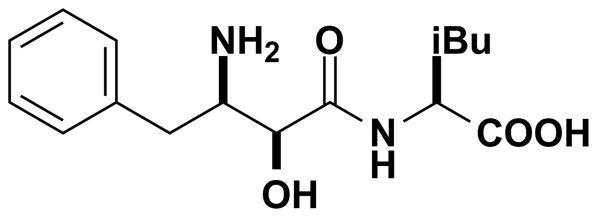 |
| BETAHISTINE HYDROCHLORIDE | 5.00 | MicroSource Discovery Systems Inc. | BRD-K91315211 | 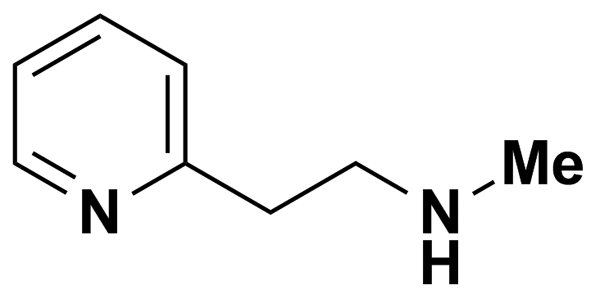 |
| BETAHISTINE MESYLATE | 3.05 | Prestwick Chemical Inc. | BRD-K91315211 | 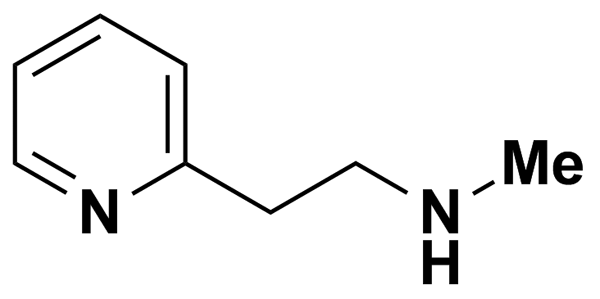 |
| BETULINIC ACID | 5.47 | Biomol International Inc. | BRD-K45401373 | 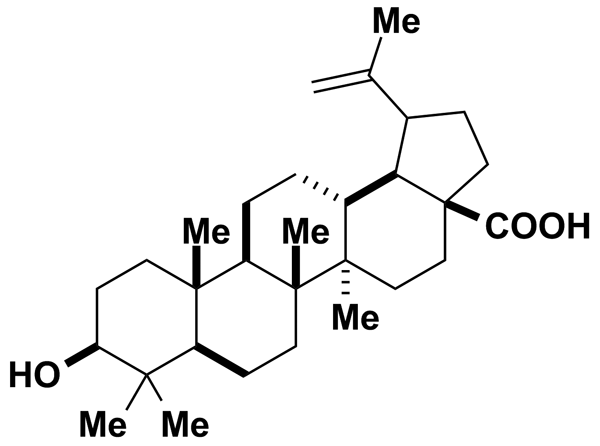 |
| BIOCHANIN A | 5.00 | MicroSource Discovery Systems Inc. | BRD-K73303757 | 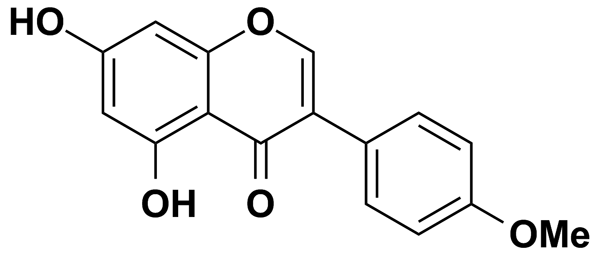 |
| BISACODYL | 2.77 | Prestwick Chemical Inc. | BRD-K39987650 | 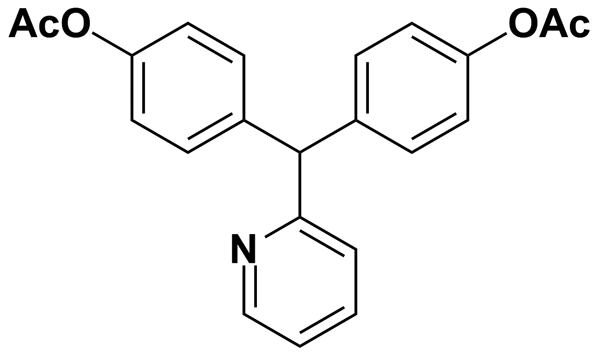 |
| BLEBBISTATIN | 8.55 | Biomol International Inc. | BRD-A75817871 | 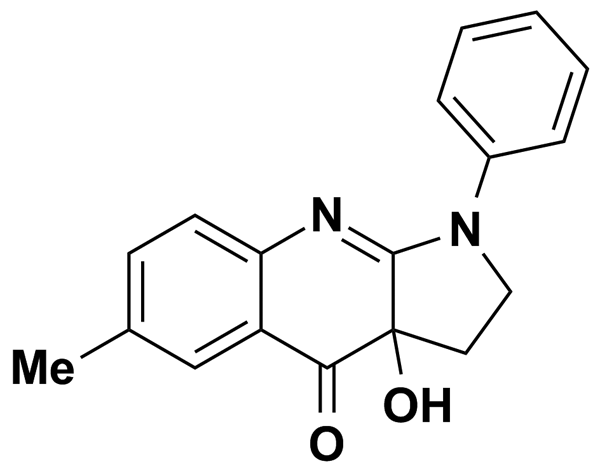 |
| BML-190 | 0.50 | Biomol International Inc. | BRD-K94379058 | 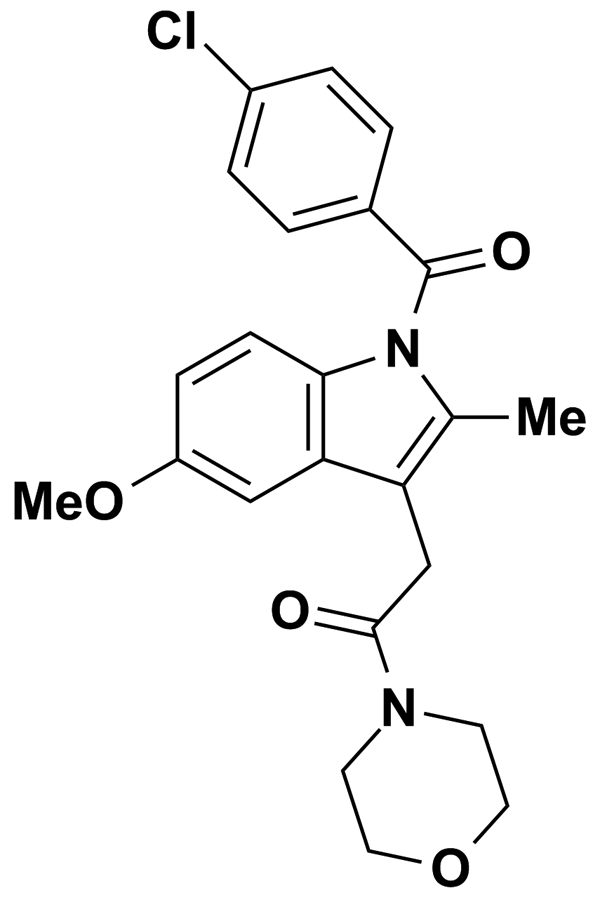 |
| BROMHEXINE HYDROCHLORIDE | 5.00 | MicroSource Discovery Systems Inc. | BRD-K47631482 | 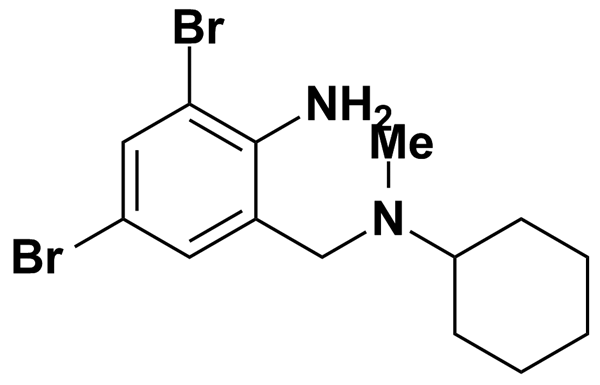 |
| BROMO-7-NITROINDAZOLE [3-BROMO-7-NITROINDAZOLE] | 10.33 | Biomol International Inc. | BRD-K24689407 | 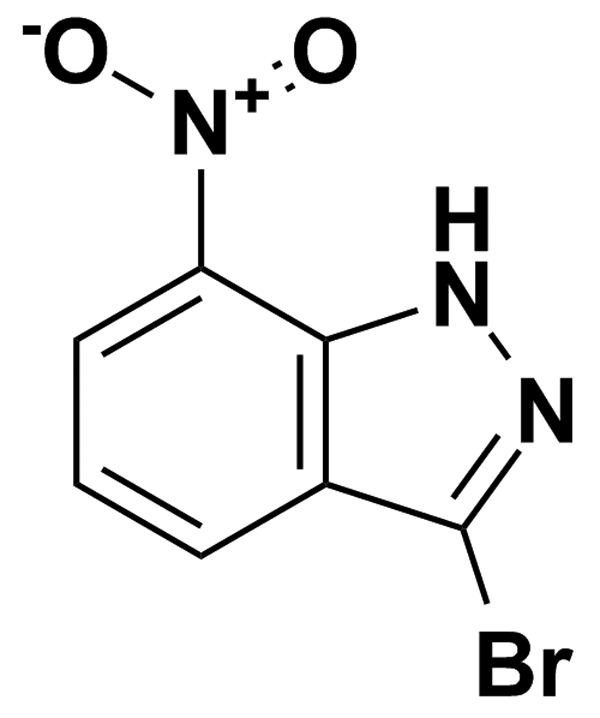 |
| BROMOCRIPTINE MESYLATE | 5.00 | MicroSource Discovery Systems Inc. | BRD-A80151636 | 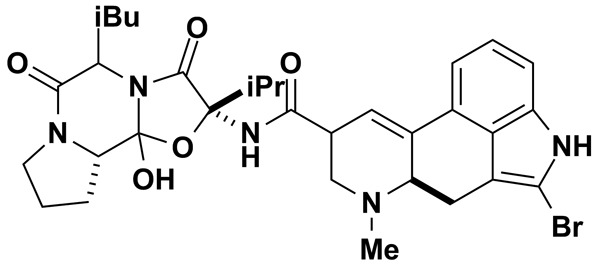 |
| BROMOPRIDE | 2.90 | Prestwick Chemical Inc. | BRD-K73642618 | 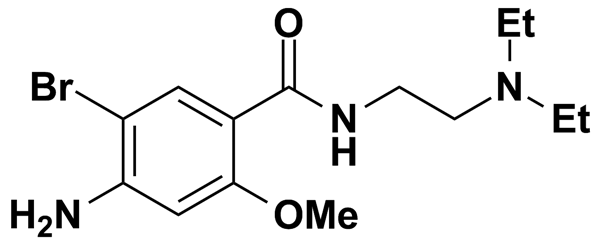 |
| BUMETANIDE | 6.86 | Biomol International Inc. | BRD-K38197229 | 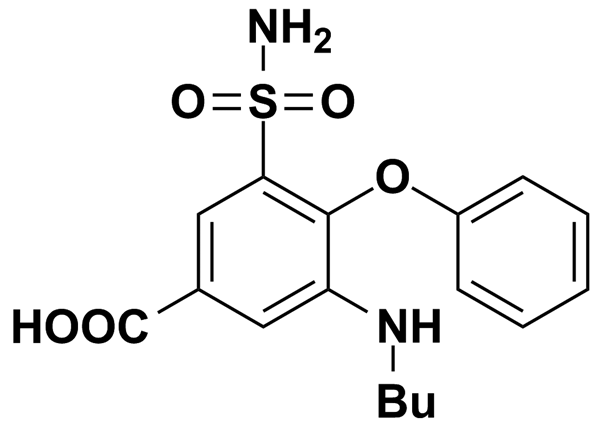 |
| BUPIVACAINE HYDROCHLORIDE | 3.08 | Prestwick Chemical Inc. | BRD-A01636364 | 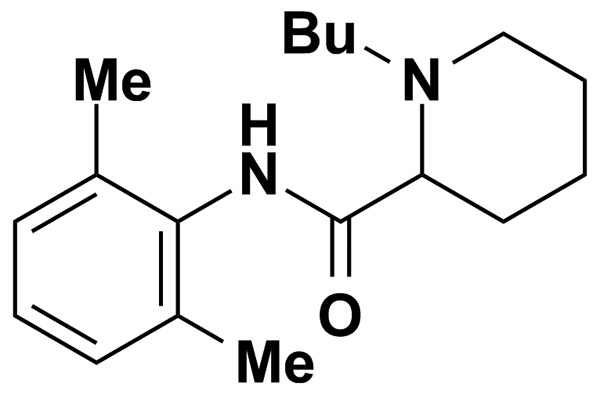 |
| BUSPIRONE | 5.00 | Biomol International Inc. | BRD-K93461745 | 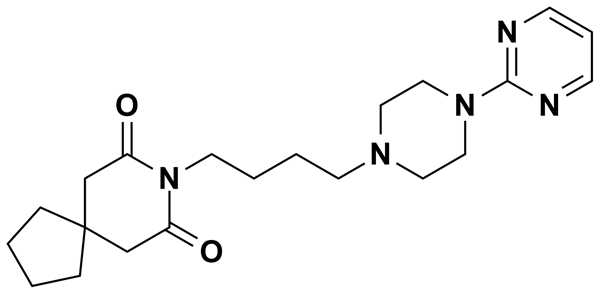 |
| BUSPIRONE HYDROCHLORIDE | 2.37 | Prestwick Chemical Inc. | BRD-K93461745 | 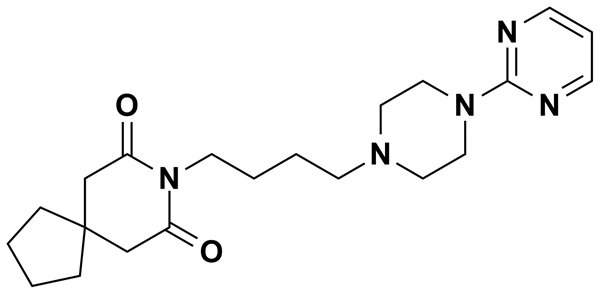 |
| BUTAMBEN | 5.17 | Prestwick Chemical Inc. | BRD-K27217864 | 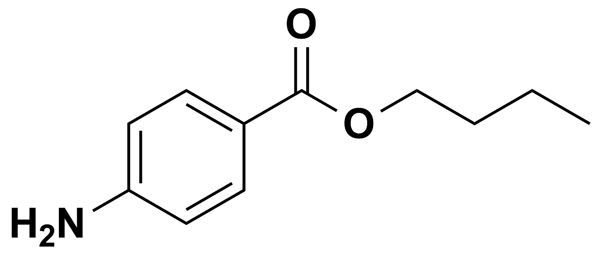 |
| BW-B 70C | 7.90 | Biomol International Inc. | BRD-A55946879 |  |
| C2 CERAMIDE | 0.50 | Biomol International Inc. | BRD-K41707108 |  |
| CA-074-ME | 6.29 | Biomol International Inc. | BRD-A56020723 |  |
| CAFFEINE | 5.00 | MicroSource Discovery Systems Inc. | BRD-K02404261 |  |
| CAPSAZEPINE | 6.63 | Biomol International Inc. | BRD-K44849676 |  |
| CAPTOPRIL | 4.60 | Prestwick Chemical Inc. | BRD-K54529596 |  |
| CARBAMAZEPINE | 4.23 | Prestwick Chemical Inc. | BRD-K71799949 |  |
| CARBETAPENTANE CITRATE | 1.90 | Prestwick Chemical Inc. | BRD-K06181161 |  |
| CARBINOXAMINE MALEATE SALT | 2.46 | Prestwick Chemical Inc. | BRD-A29426959 |  |
| CELECOXIB | 5.00 | MicroSource Discovery Systems Inc. | BRD-K02637541 |  |
| CEPHALOTHIN SODIUM SALT | 2.38 | Prestwick Chemical Inc. | BRD-K28210218 |  |
| CERULENIN | 11.20 | Biomol International Inc. | BRD-K52075040 |  |
| CGP-37157 | 7.71 | Biomol International Inc. | BRD-A35623999 |  |
| CHLORAMBUCIL | 3.29 | Prestwick Chemical Inc. | BRD-K29458283 |  |
| CHLORAMPHENICOL | 3.09 | Prestwick Chemical Inc. | BRD-K08111712 |  |
| CHLORMEZANONE | 5.00 | Biomol International Inc. | BRD-A20348246 |  |
| CHLOROXYLENOL | 5.00 | MicroSource Discovery Systems Inc. | BRD-K17223896 |  |
| CHLORPHENIRAMINE MALEATE | 2.56 | Prestwick Chemical Inc. | BRD-A04553218 |  |
| CHLORPROPAMIDE | 3.61 | Prestwick Chemical Inc. | BRD-K97746869 |  |
| CHLORPROPHAM | 5.00 | MicroSource Discovery Systems Inc. | BRD-K59590127 |  |
| CHLORPROTHIXENE HYDROCHLORIDE | 2.84 | Prestwick Chemical Inc. | BRD-K59058766 |  |
| CHLORPYRIFOS | 5.00 | MicroSource Discovery Systems Inc. | BRD-K08303368 |  |
| CHLORTHALIDONE | 2.95 | Prestwick Chemical Inc. | BRD-A26384407 |  |
| CHLORZOXAZONE | 5.90 | Prestwick Chemical Inc. | BRD-K98174813 |  |
| CHROMOCARB | 5.00 | MicroSource Discovery Systems Inc. | BRD-K94720315 |  |
| CIGLITAZONE | 0.50 | Biomol International Inc. | BRD-A93000692 |  |
| CIMATEROL | 11.40 | Biomol International Inc. | BRD-A65440446 |  |
| CINNARAZINE | 5.00 | MicroSource Discovery Systems Inc. | BRD-K32256916 |  |
| CINNARIZINE | 2.71 | Prestwick Chemical Inc. | BRD-K07220430 |  |
| CINOXACIN | 3.81 | Prestwick Chemical Inc. | BRD-K14704277 |  |
| CIPROFIBRATE | 3.46 | Prestwick Chemical Inc. | BRD-A49358627 |  |
| CIPROFLOXACIN HYDROCHLORIDE | 2.59 | Prestwick Chemical Inc. | BRD-K04804440 |  |
| CIRAZOLINE | 11.56 | Biomol International Inc. | BRD-K54142781 |  |
| CLEMASTINE FUMARATE | 2.17 | Prestwick Chemical Inc. | BRD-K30240666 |  |
| CLENBUTEROL HYDROCHLORIDE | 3.19 | Prestwick Chemical Inc. | BRD-A75726477 |  |
| CLOBETASOL PROPIONATE | 5.00 | MicroSource Discovery Systems Inc. | BRD-A26095496 |  |
| CLOFIBRIC ACID | 4.66 | Prestwick Chemical Inc. | BRD-K19111024 |  |
| CLOMIPHENE CITRATE | 5.00 | MicroSource Discovery Systems Inc. | BRD-K29950728 |  |
| CLOMIPRAMINE | 5.00 | Biomol International Inc. | BRD-K52989797 |  |
| CLOMIPRAMINE HYDROCHLORIDE | 2.85 | Prestwick Chemical Inc. | BRD-K52989797 |  |
| CLONIDINE | 10.87 | Biomol International Inc. | BRD-K98530306 |  |
| CLONIDINE HYDROCHLORIDE | 3.75 | Prestwick Chemical Inc. | BRD-K98530306 |  |
| CLOPAMIDE | 5.00 | MicroSource Discovery Systems Inc. | BRD-A85651701 |  |
| CLOPERASTINE HYDROCHLORIDE | 2.73 | Prestwick Chemical Inc. | BRD-A80908310 |  |
| CLOPIDOGREL SULFATE | 5.00 | MicroSource Discovery Systems Inc. | BRD-K27721098 |  |
| CLOPROSTENOL | 0.50 | Biomol International Inc. | BRD-K17850764 |  |
| CLOXACILLIN SODIUM SALT | 2.18 | Prestwick Chemical Inc. | BRD-K01244426 |  |
| CLOXYQUIN | 5.00 | MicroSource Discovery Systems Inc. | BRD-K46982791 |  |
| CLOZAPINE | 5.00 | Biomol International Inc. | BRD-K37289225 |  |
| COLCHICINE | 2.50 | Prestwick Chemical Inc. | BRD-K00259736 |  |
| CORTICOSTERONE | 2.89 | Prestwick Chemical Inc. | BRD-K73589401 |  |
| CORYNANTHINE HYDROCHLORIDE | 2.56 | Prestwick Chemical Inc. | BRD-K06467078 |  |
| COTININE (-) | 5.67 | Prestwick Chemical Inc. | BRD-K94144010 |  |
| COUMOPHOS | 5.00 | MicroSource Discovery Systems Inc. | BRD-K41567533 |  |
| CURCUMIN | 5.00 | MicroSource Discovery Systems Inc. | BRD-K07572174 |  |
| CYCLIZINE | 5.00 | MicroSource Discovery Systems Inc. | BRD-K79501723 |  |
| CYCLIZINE HYDROCHLORIDE | 3.30 | Prestwick Chemical Inc. | BRD-K79501723 |  |
| CYCLOBENZAPRINE HYDROCHLORIDE | 3.21 | Prestwick Chemical Inc. | BRD-K42348709 |  |
| CYCLOPENTOLATE HYDROCHLORIDE | 3.05 | Prestwick Chemical Inc. | BRD-A77291778 |  |
| CYCLOPIAZONIC ACID | 5.00 | MicroSource Discovery Systems Inc. | BRD-A26787626 |  |
| CYCLOTHIAZIDE | 5.00 | Biomol International Inc. | BRD-A38675539 |  |
| CYPROTERONE ACETATE | 5.00 | MicroSource Discovery Systems Inc. | BRD-A95207036 |  |
| DAIDZEIN | 5.00 | Biomol International Inc. | BRD-K42095107 |  |
| DANAZOL | 5.00 | MicroSource Discovery Systems Inc. | BRD-A92537424 |  |
| DANTRON | 5.00 | MicroSource Discovery Systems Inc. | BRD-K10065684 |  |
| DAPHNETIN | 5.00 | MicroSource Discovery Systems Inc. | BRD-K61269089 |  |
| DAPSONE | 4.03 | Prestwick Chemical Inc. | BRD-K62363391 |  |
| DAUNORUBICIN HYDROCHLORIDE | 1.77 | Prestwick Chemical Inc. | BRD-K43389675 |  |
| DEMECLOCYCLINE HYDROCHLORIDE | 1.99 | Prestwick Chemical Inc. | BRD-A34145622 |  |
| DEPRENYL | 13.35 | Biomol International Inc. | BRD-K86434416 |  |
| DEXTROMETHORPHAN HYDROBROMIDE | 5.00 | MicroSource Discovery Systems Inc. | BRD-K24053527 |  |
| DIAZEPAM | 5.00 | MicroSource Discovery Systems Inc. | BRD-K16508793 |  |
| DIAZOXIDE | 10.84 | Biomol International Inc. | BRD-K73109821 |  |
| DIBUCAINE | 2.91 | Prestwick Chemical Inc. | BRD-K99121711 |  |
| DIBUCAINE HYDROCHLORIDE | 5.00 | MicroSource Discovery Systems Inc. | BRD-K99121711 |  |
| DIBUTYRYLCYCLIC AMP | 5.08 | Biomol International Inc. | BRD-A94624445 |  |
| DICHLORODIPHENYLTRICHLOROETHANE | 5.00 | MicroSource Discovery Systems Inc. | BRD-K51799616 |  |
| DICLOFENAC SODIUM | 3.13 | Prestwick Chemical Inc. | BRD-K08252256 |  |
| DICLOXACILLIN SODIUM SALT | 1.96 | Prestwick Chemical Inc. | BRD-K05673000 |  |
| DICUMAROL | 2.97 | Prestwick Chemical Inc. | BRD-K82236179 |  |
| DIENESTROL | 3.75 | Prestwick Chemical Inc. | BRD-K95309561 |  |
| DIETHYLCARBAMAZINE CITRATE | 2.55 | Prestwick Chemical Inc. | BRD-K45542189 |  |
| DIFLUNISAL | 4.00 | Prestwick Chemical Inc. | BRD-K22031190 |  |
| DIGITOXIN | 5.00 | MicroSource Discovery Systems Inc. | BRD-A93236127 |  |
| DIGOXIN | 5.00 | MicroSource Discovery Systems Inc. | BRD-A94756469 |  |
| DIHYDROERGOTAMINE MESYLATE | 5.00 | MicroSource Discovery Systems Inc. | BRD-K72166146 |  |
| DIINDOLYLMETHANE | 0.50 | Biomol International Inc. | BRD-K37846922 |  |
| DILTIAZEM | 6.03 | Biomol International Inc. | BRD-K24023109 |  |
| DILTIAZEM HYDROCHLORIDE | 2.22 | Prestwick Chemical Inc. | BRD-K24023109 |  |
| DIMETHOATE | 5.00 | MicroSource Discovery Systems Inc. | BRD-K94763113 |  |
| DIMETHYL 4,4-O-PHENYLENE-BIS (3-THIOPHANATE) | 5.00 | MicroSource Discovery Systems Inc. | BRD-K90168339 |  |
| DIPHENHYDRAMINE HYDROCHLORIDE | 3.43 | Prestwick Chemical Inc. | BRD-K47278471 |  |
| DIPHENYLPYRALINE HYDROCHLORIDE | 3.15 | Prestwick Chemical Inc. | BRD-K22936972 |  |
| DIPROPHYLLINE | 3.93 | Prestwick Chemical Inc. | BRD-A00827783 |  |
| DIPYRONE | 2.99 | Prestwick Chemical Inc. | BRD-K76812510 |  |
| DISOPYRAMIDE | 2.95 | Prestwick Chemical Inc. | BRD-A29734509 |  |
| DISOPYRAMIDE PHOSPHATE | 5.00 | MicroSource Discovery Systems Inc. | BRD-A29734509 |  |
| DISULFIRAM | 3.37 | Prestwick Chemical Inc. | BRD-K32744045 |  |
| DL-PDMP | 0.50 | Biomol International Inc. | BRD-K05653692 |  |
| DOMPERIDONE | 5.00 | Biomol International Inc. | BRD-K38305202 |  |
| DOXEPIN HYDROCHLORIDE | 5.00 | MicroSource Discovery Systems Inc. | BRD-K37694030 |  |
| DOXYLAMINE SUCCINATE | 2.57 | Prestwick Chemical Inc. | BRD-A44008656 |  |
| DROFENINE HYDROCHLORIDE | 2.83 | Prestwick Chemical Inc. | BRD-A22267103 |  |
| DROPERIDOL | 2.64 | Prestwick Chemical Inc. | BRD-K97158071 |  |
| DROPROPIZINE (R,S) | 4.23 | Prestwick Chemical Inc. | BRD-A29349577 |  |
| DYCLONINE HYDROCHLORIDE | 3.07 | Prestwick Chemical Inc. | BRD-K72259270 |  |
| E-4031 | 6.23 | Biomol International Inc. | BRD-K41713976 |  |
| EBSELEN | 9.12 | Biomol International Inc. | BRD-K29359156 |  |
| EBURNAMONINE (-) | 3.40 | Prestwick Chemical Inc. | BRD-K40227168 |  |
| ECONAZOLE NITRATE | 2.25 | Prestwick Chemical Inc. | BRD-A51820102 |  |
| EHNA | 9.01 | Biomol International Inc. | BRD-K27450477 |  |
| EICOSAPENTAENOIC ACID (20:5 N-3) | 0.50 | Biomol International Inc. | BRD-K47192521 |  |
| ELLIPTICINE | 4.06 | Prestwick Chemical Inc. | BRD-K85985071 |  |
| ENOXACIN | 3.12 | Prestwick Chemical Inc. | BRD-K78113049 |  |
| ENOXOLONE | 5.00 | MicroSource Discovery Systems Inc. | BRD-A20200672 |  |
| EPICATECHIN-(-) | 3.45 | Prestwick Chemical Inc. | BRD-K58736316 |  |
| EPIVINCAMINE | 2.82 | Prestwick Chemical Inc. | BRD-K89704198 |  |
| ESTRADIOL | 9.18 | Biomol International Inc. | BRD-K18910433 |  |
| ESTRADIOL CYPIONATE | 5.00 | MicroSource Discovery Systems Inc. | BRD-A91452556 |  |
| ESTRADIOL DIACETATE | 5.00 | MicroSource Discovery Systems Inc. | BRD-A33614871 |  |
| ESTRADIOL VALERATE | 5.00 | MicroSource Discovery Systems Inc. | BRD-A39747742 |  |
| ESTRADIOL-3-SULFATE, SODIUM SALT | 5.00 | MicroSource Discovery Systems Inc. | BRD-A91702150 |  |
| ESTRIOL | 5.00 | MicroSource Discovery Systems Inc. | BRD-A18620900 |  |
| ETHACRYNIC ACID | 3.30 | Prestwick Chemical Inc. | BRD-K63630713 |  |
| ETHINYL ESTRADIOL | 5.00 | MicroSource Discovery Systems Inc. | BRD-A02367930 |  |
| ETHION | 5.00 | MicroSource Discovery Systems Inc. | BRD-K84268861 |  |
| ETHIONAMIDE | 6.02 | Prestwick Chemical Inc. | BRD-K33710385 |  |
| ETHOPROP | 5.00 | MicroSource Discovery Systems Inc. | BRD-K08556791 |  |
| ETHOSUXIMIDE | 7.08 | Prestwick Chemical Inc. | BRD-A99633051 |  |
| ETHOXYQUIN | 4.60 | Prestwick Chemical Inc. | BRD-K56792340 |  |
| ETODOLAC | 3.48 | Prestwick Chemical Inc. | BRD-A74667430 |  |
| ETOFYLLINE | 4.46 | Prestwick Chemical Inc. | BRD-K83064458 |  |
| ETOPOSIDE | 1.70 | Prestwick Chemical Inc. | BRD-K37798499 |  |
| EUCATROPINE HYDROCHLORIDE | 5.00 | MicroSource Discovery Systems Inc. | BRD-A69786436 |  |
| FAMCICLOVIR | 5.00 | MicroSource Discovery Systems Inc. | BRD-K45033733 |  |
| FCCP | 9.84 | Biomol International Inc. | BRD-K14821540 |  |
| FELBAMATE | 5.00 | Biomol International Inc. | BRD-K99107520 |  |
| FENBENDAZOLE | 3.34 | Prestwick Chemical Inc. | BRD-K51318897 |  |
| FENBUFEN | 3.93 | Prestwick Chemical Inc. | BRD-K12513978 |  |
| FENDILINE HYDROCHLORIDE | 2.84 | Prestwick Chemical Inc. | BRD-A71033472 |  |
| FENOFIBRATE | 2.77 | Prestwick Chemical Inc. | BRD-K50388907 |  |
| FENOLDOPAM | 5.00 | Biomol International Inc. | BRD-A50684349 |  |
| FENOTEROL HYDROBROMIDE | 2.60 | Prestwick Chemical Inc. | BRD-A97104540 |  |
| FENSPIRIDE HYDROCHLORIDE | 3.37 | Prestwick Chemical Inc. | BRD-K26739552 |  |
| FENTHION | 5.00 | MicroSource Discovery Systems Inc. | BRD-K67217586 |  |
| FGIN-1-27 | 5.00 | Biomol International Inc. | BRD-K09778810 |  |
| FIPEXIDE HYDROCHLORIDE | 2.35 | Prestwick Chemical Inc. | BRD-K37688416 |  |
| FIPRONIL | 5.72 | Biomol International Inc. | BRD-A50675702 |  |
| FLECAINIDE | 6.03 | Biomol International Inc. | BRD-A09472452 |  |
| FLECAINIDE ACETATE | 2.11 | Prestwick Chemical Inc. | BRD-A09472452 |  |
| FLOPROPIONE | 5.00 | MicroSource Discovery Systems Inc. | BRD-K43383936 |  |
| FLUCONAZOLE | 5.00 | MicroSource Discovery Systems Inc. | BRD-K05977355 |  |
| FLUFENAMIC ACID | 8.89 | Biomol International Inc. | BRD-K44067360 |  |
| FLUMEQUINE | 3.83 | Prestwick Chemical Inc. | BRD-A69777949 |  |
| FLUNARIZINE | 6.18 | Biomol International Inc. | BRD-K29582677 |  |
| FLUNARIZINE DIHYDROCHLORIDE | 2.09 | Prestwick Chemical Inc. | BRD-K29582677 |  |
| FLUNISOLIDE | 5.00 | MicroSource Discovery Systems Inc. | BRD-A65449987 |  |
| FLUOROMETHOLONE | 5.00 | MicroSource Discovery Systems Inc. | BRD-A13133631 |  |
| FLUPHENAZINE | 5.00 | Biomol International Inc. | BRD-K55127134 |  |
| FLUPHENAZINE DIHYDROCHLORIDE | 1.96 | Prestwick Chemical Inc. | BRD-K55127134 |  |
| FLUPROSTENOL | 0.50 | Biomol International Inc. | BRD-K31611373 |  |
| FLURANDRENOLIDE | 5.00 | MicroSource Discovery Systems Inc. | BRD-A49765801 |  |
| FLUTAMIDE | 3.62 | Prestwick Chemical Inc. | BRD-K28307902 |  |
| FORMONONETIN | 5.00 | MicroSource Discovery Systems Inc. | BRD-K55567017 |  |
| FUREGRELATE SODIUM | 5.00 | MicroSource Discovery Systems Inc. | BRD-K55529781 |  |
| FUROSEMIDE | 3.02 | Prestwick Chemical Inc. | BRD-K78010432 |  |
| FUSIDIC ACID | 5.00 | MicroSource Discovery Systems Inc. | BRD-A06935312 |  |
| GALANTHAMINE HYDROBROMIDE | 2.72 | Prestwick Chemical Inc. | BRD-K49481516 |  |
| GAMMA-LINOLENIC ACID (18:3 N-6) | 0.50 | Biomol International Inc. | BRD-K18059238 |  |
| GBR 12935 | 5.00 | Biomol International Inc. | BRD-K50135270 |  |
| GEDUNIN | 5.00 | MicroSource Discovery Systems Inc. | BRD-A48397526 |  |
| GEMFIBROZIL | 3.99 | Prestwick Chemical Inc. | BRD-K11129031 |  |
| GENISTEIN | 5.00 | MicroSource Discovery Systems Inc. | BRD-K43797669 |  |
| GENTIAN VIOLET | 5.00 | MicroSource Discovery Systems Inc. | BRD-K60025295 |  |
| GF-109203X | 6.06 | Biomol International Inc. | BRD-K31342827 |  |
| GINGEROL | 8.49 | Biomol International Inc. | BRD-K26117720 |  |
| GLAFENINE HYDROCHLORIDE | 2.44 | Prestwick Chemical Inc. | BRD-A38076815 |  |
| GLIMEPIRIDE | 2.04 | Prestwick Chemical Inc. | BRD-K34776109 |  |
| GLIPIZIDE | 5.61 | Biomol International Inc. | BRD-K12219985 |  |
| GLUTETHIMIDE, PARA-AMINO | 4.31 | Prestwick Chemical Inc. | BRD-A25234499 |  |
| GLYBURIDE | 5.06 | Biomol International Inc. | BRD-K36927236 |  |
| GO6976 | 6.61 | Biomol International Inc. | BRD-K59304176 |  |
| GOSSYPOL | 1.93 | Prestwick Chemical Inc. | BRD-K19295594 |  |
| GOSSYPOL-ACETIC ACID COMPLEX | 5.00 | MicroSource Discovery Systems Inc. | BRD-K19295594 |  |
| GRISEOFULVIN | 2.83 | Prestwick Chemical Inc. | BRD-K08273968 |  |
| GUAIFENESIN | 5.05 | Prestwick Chemical Inc. | BRD-A90515964 |  |
| H-89 | 5.60 | Biomol International Inc. | BRD-K27737647 |  |
| H7 | 8.58 | Biomol International Inc. | BRD-A55756846 |  |
| HA-1004 | 8.52 | Biomol International Inc. | BRD-K05434375 |  |
| HALOPERIDOL | 5.00 | Biomol International Inc. | BRD-K67783091 |  |
| HARMALINE | 5.00 | MicroSource Discovery Systems Inc. | BRD-K91317041 |  |
| HARMALINE HYDROCHLORIDE DIHYDRATE | 3.49 | Prestwick Chemical Inc. | BRD-K91317041 |  |
| HARMALOL HYDROCHLORIDE | 5.00 | MicroSource Discovery Systems Inc. | BRD-K14756138 |  |
| HARMALOL HYDROCHLORIDE DIHYDRATE | 3.67 | Prestwick Chemical Inc. | BRD-K14756138 |  |
| HESPERETIN | 3.31 | Prestwick Chemical Inc. | BRD-K30553453 |  |
| HEXACHLOROPHENE | 5.00 | MicroSource Discovery Systems Inc. | BRD-K99792991 |  |
| HEXESTROL | 3.70 | Prestwick Chemical Inc. | BRD-A01078468 |  |
| HEXYLRESORCINOL | 5.00 | MicroSource Discovery Systems Inc. | BRD-K99946902 |  |
| HOMATROPINE METHYLBROMIDE | 5.00 | MicroSource Discovery Systems Inc. | BRD-A43999749 |  |
| HYCANTHONE | 2.81 | Prestwick Chemical Inc. | BRD-K50406511 |  |
| HYDRALAZINE HYDROCHLORIDE | 5.00 | MicroSource Discovery Systems Inc. | BRD-K82103381 |  |
| HYDROCHLOROTHIAZIDE | 3.36 | Prestwick Chemical Inc. | BRD-K13078532 |  |
| HYDROCORTISONE | 5.00 | MicroSource Discovery Systems Inc. | BRD-A75172220 |  |
| HYDROCORTISONE ACETATE | 5.00 | MicroSource Discovery Systems Inc. | BRD-A65767837 |  |
| HYDROCORTISONE HEMISUCCINATE | 5.00 | MicroSource Discovery Systems Inc. | BRD-A07000685 |  |
| HYDROFLUMETHIAZIDE | 3.02 | Prestwick Chemical Inc. | BRD-K36862742 |  |
| HYDROXYPROGESTERONE CAPROATE | 5.00 | MicroSource Discovery Systems Inc. | BRD-A29731977 |  |
| HYDROXYTACRINE MALEATE (R,S) | 3.03 | Prestwick Chemical Inc. | BRD-A98299281 |  |
| HYOSCYAMINE | 5.00 | MicroSource Discovery Systems Inc. | BRD-A78303415 |  |
| IAA-94 | 7.00 | Biomol International Inc. | BRD-K85383046 |  |
| IB-MECA | 4.90 | Biomol International Inc. | BRD-A48809242 |  |
| IBMX | 11.25 | Biomol International Inc. | BRD-K94979336 |  |
| IMIPRAMINE HYDROCHLORIDE | 3.16 | Prestwick Chemical Inc. | BRD-K38436528 |  |
| INDAPAMIDE | 2.73 | Prestwick Chemical Inc. | BRD-A95869247 |  |
| INDOLE-3-CARBINOL | 5.00 | MicroSource Discovery Systems Inc. | BRD-K01815685 |  |
| IPRONIAZIDE PHOSPHATE | 3.61 | Prestwick Chemical Inc. | BRD-K88568253 |  |
| IRBESARTAN | 5.00 | MicroSource Discovery Systems Inc. | BRD-K60038276 |  |
| ISOCARBOXAZID | 4.32 | Prestwick Chemical Inc. | BRD-K93332168 |  |
| ISOXICAM | 2.98 | Prestwick Chemical Inc. | BRD-A75552914 |  |
| KAEMPFEROL | 3.49 | Prestwick Chemical Inc. | BRD-K12807006 |  |
| KETANSERIN | 5.00 | Biomol International Inc. | BRD-K49671696 |  |
| KETANSERIN TARTRATE HYDRATE | 1.77 | Prestwick Chemical Inc. | BRD-K49671696 |  |
| KETOCONAZOLE | 5.00 | MicroSource Discovery Systems Inc. | BRD-A38350138 |  |
| KETOPROFEN | 3.93 | Prestwick Chemical Inc. | BRD-A97739905 |  |
| KETOTIFEN FUMARATE | 2.35 | Prestwick Chemical Inc. | BRD-K28936863 |  |
| KHELLIN | 3.84 | Prestwick Chemical Inc. | BRD-K80353807 |  |
| KINETIN RIBOSIDE | 5.00 | MicroSource Discovery Systems Inc. | BRD-K94325918 |  |
| L-745,870 | 5.00 | Biomol International Inc. | BRD-K05528470 |  |
| LABETALOL HYDROCHLORIDE | 2.74 | Prestwick Chemical Inc. | BRD-A07440155 |  |
| LANATOSIDE C | 5.00 | MicroSource Discovery Systems Inc. | BRD-A64242993 |  |
| LANSOPRAZOLE | 2.71 | Prestwick Chemical Inc. | BRD-A49172652 |  |
| LAPACHOL | 5.00 | MicroSource Discovery Systems Inc. | BRD-A06912736 |  |
| LAUDANOSINE (R,S) | 2.80 | Prestwick Chemical Inc. | BRD-A24817035 |  |
| LAVENDUSTIN A | 6.56 | Biomol International Inc. | BRD-K23583188 |  |
| LEFLUNOMIDE | 3.70 | Prestwick Chemical Inc. | BRD-K78692225 |  |
| LETROZOLE | 3.51 | Prestwick Chemical Inc. | BRD-K88789588 |  |
| LEVAMISOLE HYDROCHLORIDE | 4.15 | Prestwick Chemical Inc. | BRD-A46393198 |  |
| LEVAMISOLE HYDROCHLORIDE | 5.00 | MicroSource Discovery Systems Inc. | BRD-K73107279 |  |
| LIDOCAINE | 10.67 | Biomol International Inc. | BRD-K52662033 |  |
| LIDOCAINE HYDROCHLORIDE | 3.69 | Prestwick Chemical Inc. | BRD-K52662033 |  |
| LINCOMYCIN HYDROCHLORIDE | 5.00 | MicroSource Discovery Systems Inc. | BRD-K08033334 |  |
| LIOTHYRONINE | 5.00 | MicroSource Discovery Systems Inc. | BRD-A74642112 |  |
| LISINOPRIL | 2.26 | Prestwick Chemical Inc. | BRD-K67966701 |  |
| LOMEFLOXACIN HYDROCHLORIDE | 2.58 | Prestwick Chemical Inc. | BRD-A75850590 |  |
| LONIDAMINE | 5.00 | MicroSource Discovery Systems Inc. | BRD-K96670504 |  |
| LOPERAMIDE | 5.24 | Biomol International Inc. | BRD-K61250553 |  |
| LOPERAMIDE HYDROCHLORIDE | 1.95 | Prestwick Chemical Inc. | BRD-K61250553 |  |
| LORATADINE | 5.00 | MicroSource Discovery Systems Inc. | BRD-K82795137 |  |
| LOSARTAN | 5.00 | MicroSource Discovery Systems Inc. | BRD-K76205745 |  |
| LUTEOLIN | 3.49 | Prestwick Chemical Inc. | BRD-K05236810 |  |
| LY-294002 | 8.13 | Biomol International Inc. | BRD-K27305650 |  |
| LY-83583 | 9.99 | Biomol International Inc. | BRD-K62792802 |  |
| LYCORINE | 5.00 | MicroSource Discovery Systems Inc. | BRD-A10335634 |  |
| MAPP, D-ERYTHRO | 0.50 | Biomol International Inc. | BRD-K76274772 |  |
| MCI-186 | 14.35 | Biomol International Inc. | BRD-K35458079 |  |
| MEAD ACID (20:3 N-9) | 0.50 | Biomol International Inc. | BRD-K96144918 |  |
| MEBEVERINE HYDROCHLORIDE | 2.15 | Prestwick Chemical Inc. | BRD-A09467419 |  |
| MECLOFENAMATE SODIUM | 5.00 | MicroSource Discovery Systems Inc. | BRD-K50398167 |  |
| MECLOFENAMIC ACID SODIUM SALT MONOHYDRATE | 2.97 | Prestwick Chemical Inc. | BRD-K50398167 |  |
| MECLOFENOXATE HYDROCHLORIDE | 3.40 | Prestwick Chemical Inc. | BRD-K10314788 |  |
| MECLOZINE DIHYDROCHLORIDE | 2.16 | Prestwick Chemical Inc. | BRD-A50311610 |  |
| MEDROXYPROGESTERONE ACETATE | 5.00 | MicroSource Discovery Systems Inc. | BRD-A61221616 |  |
| MEDRYSONE | 5.00 | MicroSource Discovery Systems Inc. | BRD-A20126139 |  |
| MEFENAMIC ACID | 4.14 | Prestwick Chemical Inc. | BRD-K92778217 |  |
| MEFEXAMIDE HYDROCHLORIDE | 3.16 | Prestwick Chemical Inc. | BRD-K20655524 |  |
| MEFLOQUINE HYDROCHLORIDE | 2.41 | Prestwick Chemical Inc. | BRD-A89585551 |  |
| MELATONIN | 4.31 | Prestwick Chemical Inc. | BRD-K97530723 |  |
| MELOXICAM | 5.00 | MicroSource Discovery Systems Inc. | BRD-A84174393 |  |
| MELPHALAN | 5.00 | MicroSource Discovery Systems Inc. | BRD-K87827419 |  |
| MEPENZOLATE BROMIDE | 2.37 | Prestwick Chemical Inc. | BRD-A62421304 |  |
| MEPHENYTOIN | 4.58 | Prestwick Chemical Inc. | BRD-A83937277 |  |
| METHACYCLINE HYDROCHLORIDE | 2.09 | Prestwick Chemical Inc. | BRD-A49035384 |  |
| METHAPYRILENE HYDROCHLORIDE | 3.36 | Prestwick Chemical Inc. | BRD-K47323024 |  |
| METHICILLIN SODIUM | 5.00 | MicroSource Discovery Systems Inc. | BRD-K34388247 |  |
| METHIOTHEPIN | 5.00 | Biomol International Inc. | BRD-A07932845 |  |
| METHIOTHEPIN MALEATE | 2.12 | Prestwick Chemical Inc. | BRD-A07932845 |  |
| METHOCARBAMOL | 4.15 | Prestwick Chemical Inc. | BRD-A31521121 |  |
| METHOPRENE ACID | 0.50 | Biomol International Inc. | BRD-A41145729 |  |
| METHOXY VERAPAMIL | 5.16 | Biomol International Inc. | BRD-A52922642 |  |
| METHOXY-8-PSORALEN | 4.63 | Prestwick Chemical Inc. | BRD-K63430059 |  |
| METHSCOPOLAMINE BROMIDE | 5.00 | MicroSource Discovery Systems Inc. | BRD-A03932035 |  |
| METHYLPREDNISOLONE | 5.00 | MicroSource Discovery Systems Inc. | BRD-A53176877 |  |
| METHYSERGIDE | 5.00 | Biomol International Inc. | BRD-K35941380 |  |
| METOCLOPRAMIDE | 5.00 | Biomol International Inc. | BRD-K75641298 |  |
| METOCLOPRAMIDE MONOHYDROCHLORIDE | 2.97 | Prestwick Chemical Inc. | BRD-K75641298 |  |
| METOLAZONE | 2.73 | Prestwick Chemical Inc. | BRD-A61793559 |  |
| MEVINOLIN (LOVASTATIN) | 6.18 | Biomol International Inc. | BRD-K09416995 |  |
| MEXILETINE HYDROCHLORIDE | 4.64 | Prestwick Chemical Inc. | BRD-A64092382 |  |
| MIANSERINE HYDROCHLORIDE | 3.32 | Prestwick Chemical Inc. | BRD-A19661776 |  |
| MICONAZOLE | 2.40 | Prestwick Chemical Inc. | BRD-A82396632 |  |
| MICONAZOLE NITRATE | 5.00 | MicroSource Discovery Systems Inc. | BRD-A82396632 |  |
| MIDODRINE HYDROCHLORIDE | 3.44 | Prestwick Chemical Inc. | BRD-A79981887 |  |
| MIFEPRISTONE | 2.33 | Prestwick Chemical Inc. | BRD-K37270826 |  |
| MINAPRINE DIHYDROCHLORIDE | 2.69 | Prestwick Chemical Inc. | BRD-K02867583 |  |
| MINOXIDIL | 11.95 | Biomol International Inc. | BRD-K06902185 |  |
| MITOXANTRONE DIHYDROCHLORIDE | 1.93 | Prestwick Chemical Inc. | BRD-K21680192 |  |
| ML7 | 6.01 | Biomol International Inc. | BRD-K93201660 |  |
| ML9 | 7.70 | Biomol International Inc. | BRD-K68402494 |  |
| MODAFINIL | 5.00 | MicroSource Discovery Systems Inc. | BRD-A16332958 |  |
| MOLSIDOMINE | 5.00 | MicroSource Discovery Systems Inc. | BRD-K63861289 |  |
| MONOCROTALINE | 3.07 | Prestwick Chemical Inc. | BRD-K65508953 |  |
| MY-5445 | 7.53 | Biomol International Inc. | BRD-K90524085 |  |
| MYCOPHENOLIC ACID | 3.12 | Prestwick Chemical Inc. | BRD-K63750851 |  |
| MYRICETIN | 3.14 | Prestwick Chemical Inc. | BRD-K43149758 |  |
| N- (3-TRIFLUOROMETHYLPHENYL)PIPERAZINE HYDROCHLORIDE (TFMPP) | 5.00 | MicroSource Discovery Systems Inc. | BRD-K94887716 |  |
| N- (9-FLUORENYLMETHOXYCARBONYL)-L-LEUCINE | 5.00 | MicroSource Discovery Systems Inc. | BRD-K65275554 |  |
| N-LINOLEOYLGLYCINE | 0.50 | Biomol International Inc. | BRD-K76293260 |  |
| N-METHYL (-)EPHEDRINE | 5.00 | MicroSource Discovery Systems Inc. | BRD-K82236108 |  |
| N-PHENYLANTHRANILIC | 11.72 | Biomol International Inc. | BRD-K80863915 |  |
| N9-ISOPROPYLOLOMOUCINE | 7.66 | Biomol International Inc. | BRD-K71726959 |  |
| NAFCILLIN SODIUM | 5.00 | MicroSource Discovery Systems Inc. | BRD-A99402294 |  |
| NAFRONYL OXALATE | 2.11 | Prestwick Chemical Inc. | BRD-A67862938 |  |
| NALBUPHINE HYDROCHLORIDE | 2.54 | Prestwick Chemical Inc. | BRD-K66404838 |  |
| NALIDIXIC ACID SODIUM SALT HYDRATE | 3.66 | Prestwick Chemical Inc. | BRD-K47886988 |  |
| NALOXONE HYDROCHLORIDE | 2.75 | Prestwick Chemical Inc. | BRD-K67511046 |  |
| NAN-190 | 5.00 | Biomol International Inc. | BRD-K69195780 |  |
| NAPHAZOLINE HYDROCHLORIDE | 4.05 | Prestwick Chemical Inc. | BRD-K77641333 |  |
| NAPROXEN(+) | 5.00 | MicroSource Discovery Systems Inc. | BRD-K59197931 |  |
| NARINGENIN | 5.00 | MicroSource Discovery Systems Inc. | BRD-K08832567 |  |
| NARINGIN HYDRATE | 1.67 | Prestwick Chemical Inc. | BRD-K02953697 |  |
| NEFOPAM HYDROCHLORIDE | 3.45 | Prestwick Chemical Inc. | BRD-A78877355 |  |
| NEOSTIGMINE BROMIDE | 3.29 | Prestwick Chemical Inc. | BRD-K18922609 |  |
| NERIIFOLIN | 5.00 | MicroSource Discovery Systems Inc. | BRD-A31385885 |  |
| NICARDIPINE | 5.21 | Biomol International Inc. | BRD-A26711594 |  |
| NICARDIPINE HYDROCHLORIDE | 1.94 | Prestwick Chemical Inc. | BRD-A26711594 |  |
| NICERGOLINE | 2.06 | Prestwick Chemical Inc. | BRD-K76810206 |  |
| NICLOSAMIDE | 3.06 | Prestwick Chemical Inc. | BRD-K35960502 |  |
| NICOTINE DITARTRATE | 5.00 | MicroSource Discovery Systems Inc. | BRD-K05395900 |  |
| NIFENAZONE | 3.24 | Prestwick Chemical Inc. | BRD-K47407372 |  |
| NIFLUMIC ACID | 8.86 | Biomol International Inc. | BRD-K98763141 |  |
| NIMODIPINE | 5.97 | Biomol International Inc. | BRD-A58048407 |  |
| NITRENDIPINE | 6.94 | Biomol International Inc. | BRD-A02006392 |  |
| NOMIFENSINE MALEATE | 2.82 | Prestwick Chemical Inc. | BRD-A29644307 |  |
| NORETHINDRONE | 5.00 | MicroSource Discovery Systems Inc. | BRD-A39415247 |  |
| NORFLOXACIN | 3.13 | Prestwick Chemical Inc. | BRD-K11196887 |  |
| NORTRIPTYLINE HYDROCHLORIDE | 3.34 | Prestwick Chemical Inc. | BRD-K91263825 |  |
| NOVOBIOCIN SODIUM | 5.00 | MicroSource Discovery Systems Inc. | BRD-K85307935 |  |
| NPPB | 8.32 | Biomol International Inc. | BRD-K89272762 |  |
| NS-1619 | 6.90 | Biomol International Inc. | BRD-K54210043 |  |
| NSC-95397 | 8.05 | Biomol International Inc. | BRD-K68143200 |  |
| OFLOXACIN | 2.77 | Prestwick Chemical Inc. | BRD-A24228527 |  |
| OXAPROZIN | 3.41 | Prestwick Chemical Inc. | BRD-K25394294 |  |
| OXETHAZAINE | 2.14 | Prestwick Chemical Inc. | BRD-K56940463 |  |
| OXIBENDAZOLE | 5.00 | MicroSource Discovery Systems Inc. | BRD-K52075715 |  |
| OXYMETAZOLINE | 5.00 | Biomol International Inc. | BRD-K16195444 |  |
| OXYPHENBUTAZONE | 3.08 | Prestwick Chemical Inc. | BRD-A33749298 |  |
| OXYQUINOLINE HEMISULFATE | 5.00 | MicroSource Discovery Systems Inc. | BRD-K66808046 |  |
| PACLITAXEL | 1.17 | Prestwick Chemical Inc. | BRD-K62008436 |  |
| PANTOPRAZOLE | 5.00 | MicroSource Discovery Systems Inc. | BRD-A22380646 |  |
| PAPAVERINE HYDROCHLORIDE | 2.66 | Prestwick Chemical Inc. | BRD-K15567136 |  |
| PARAXANTHINE | 5.00 | MicroSource Discovery Systems Inc. | BRD-K24084088 |  |
| PARGYLINE HYDROCHLORIDE | 5.11 | Prestwick Chemical Inc. | BRD-K83597974 |  |
| PAXILLINE | 5.74 | Biomol International Inc. | BRD-K38251852 |  |
| PCA 4248 | 6.92 | Biomol International Inc. | BRD-A29289453 |  |
| PCO-400 | 8.35 | Biomol International Inc. | BRD-K52721684 |  |
| PENICILLIN V POTASSIUM | 5.00 | MicroSource Discovery Systems Inc. | BRD-K43966364 |  |
| PENITREM A | 3.94 | Biomol International Inc. | BRD-K03842655 |  |
| PENTACHLOROPHENOL | 5.00 | MicroSource Discovery Systems Inc. | BRD-K50711164 |  |
| PENTOXIFYLLINE | 3.59 | Prestwick Chemical Inc. | BRD-K57569181 |  |
| PERGOLIDE MESYLATE | 2.44 | Prestwick Chemical Inc. | BRD-K60770992 |  |
| PERGOLIDE METHANESULFONATE | 5.00 | Biomol International Inc. | BRD-K60770992 |  |
| PERICIAZINE | 5.00 | MicroSource Discovery Systems Inc. | BRD-K89669299 |  |
| PERPHENAZINE | 2.48 | Prestwick Chemical Inc. | BRD-K10995081 |  |
| PERUVOSIDE | 5.00 | MicroSource Discovery Systems Inc. | BRD-A57089740 |  |
| PHENACEMIDE | 5.00 | MicroSource Discovery Systems Inc. | BRD-K40905133 |  |
| PHENACETIN | 5.58 | Prestwick Chemical Inc. | BRD-K38323065 |  |
| PHENAMIL | 8.18 | Biomol International Inc. | BRD-K21350491 |  |
| PHENELZINE SULFATE | 4.27 | Prestwick Chemical Inc. | BRD-K87024524 |  |
| PHENETHICILLIN POTASSIUM SALT | 2.48 | Prestwick Chemical Inc. | BRD-A66025870 |  |
| PHENIRAMINE MALEATE | 2.81 | Prestwick Chemical Inc. | BRD-A23072235 |  |
| PHENOLPHTHALEIN | 5.00 | MicroSource Discovery Systems Inc. | BRD-K19227686 |  |
| PHENOTHRIN | 5.00 | MicroSource Discovery Systems Inc. | BRD-A22106989 |  |
| PHENYLBUTYRIC ACID | 5.00 | MicroSource Discovery Systems Inc. | BRD-K67102207 |  |
| PHENYTOIN | 9.91 | Biomol International Inc. | BRD-K55930204 |  |
| PHLORETIN | 5.00 | MicroSource Discovery Systems Inc. | BRD-K15563106 |  |
| PHOSALONE | 5.00 | MicroSource Discovery Systems Inc. | BRD-K71671197 |  |
| PHYSOSTIGMINE SALICYLATE | 5.00 | MicroSource Discovery Systems Inc. | BRD-K25650355 |  |
| PICOTAMIDE MONOHYDRATE | 2.54 | Prestwick Chemical Inc. | BRD-K67277431 |  |
| PIFITHRIN | 8.73 | Biomol International Inc. | BRD-K66874953 |  |
| PILOCARPINE NITRATE | 3.69 | Prestwick Chemical Inc. | BRD-K85090592 |  |
| PIMETHIXENE MALEATE | 2.44 | Prestwick Chemical Inc. | BRD-K88090157 |  |
| PIMPINELLIN | 5.00 | MicroSource Discovery Systems Inc. | BRD-K93197368 |  |
| PINDOLOL | 4.03 | Prestwick Chemical Inc. | BRD-A97701745 |  |
| PIPERACILLIN SODIUM SALT | 1.85 | Prestwick Chemical Inc. | BRD-K86873305 |  |
| PIPERIDOLATE HYDROCHLORIDE | 2.78 | Prestwick Chemical Inc. | BRD-A97479839 |  |
| PIPERLONGUMINE | 3.15 | Prestwick Chemical Inc. | BRD-K24132293 |  |
| PIRENZEPINE DIHYDROCHLORIDE | 2.36 | Prestwick Chemical Inc. | BRD-K89375097 |  |
| PIRETANIDE | 2.76 | Prestwick Chemical Inc. | BRD-K87990216 |  |
| PIRIBEDIL | 5.00 | Biomol International Inc. | BRD-K47936004 |  |
| PIRIBEDIL HYDROCHLORIDE | 2.99 | Prestwick Chemical Inc. | BRD-K47936004 |  |
| PIROMIDIC ACID | 3.47 | Prestwick Chemical Inc. | BRD-K37682401 |  |
| PONALRESTAT | 5.00 | MicroSource Discovery Systems Inc. | BRD-K68332390 |  |
| PP1 | 8.89 | Biomol International Inc. | BRD-K47598052 |  |
| PRACTOLOL | 3.75 | Prestwick Chemical Inc. | BRD-A41304429 |  |
| PRAMOXINE HYDROCHLORIDE | 3.03 | Prestwick Chemical Inc. | BRD-K46523383 |  |
| PREDNISOLONE | 5.00 | MicroSource Discovery Systems Inc. | BRD-A27887842 |  |
| PREDNISOLONE ACETATE | 5.00 | MicroSource Discovery Systems Inc. | BRD-A01643550 |  |
| PREDNISONE | 5.00 | MicroSource Discovery Systems Inc. | BRD-A62525898 |  |
| PRIDINOL METHANESULFONATE SALT | 2.55 | Prestwick Chemical Inc. | BRD-K17565903 |  |
| PRILOCAINE HYDROCHLORIDE | 3.89 | Prestwick Chemical Inc. | BRD-A53952395 |  |
| PRIMIDONE | 5.00 | Biomol International Inc. | BRD-K32247306 |  |
| PROADIFEN HYDROCHLORIDE | 2.56 | Prestwick Chemical Inc. | BRD-K46317332 |  |
| PROBENECID | 3.50 | Prestwick Chemical Inc. | BRD-K95237249 |  |
| PROCAINAMIDE | 10.62 | Biomol International Inc. | BRD-K75089421 |  |
| PROCAINAMIDE HYDROCHLORIDE | 3.68 | Prestwick Chemical Inc. | BRD-K75089421 |  |
| PROCAINE HYDROCHLORIDE | 3.67 | Prestwick Chemical Inc. | BRD-K24616672 |  |
| PROCHLORPERAZINE DIMALEATE | 1.65 | Prestwick Chemical Inc. | BRD-K19352500 |  |
| PROCHLORPERAZINE EDISYLATE | 5.00 | MicroSource Discovery Systems Inc. | BRD-K19352500 |  |
| PROGESTERONE | 3.18 | Prestwick Chemical Inc. | BRD-K64994968 |  |
| PROGLUMIDE | 2.99 | Prestwick Chemical Inc. | BRD-A44863528 |  |
| PRONETHALOL HYDROCHLORIDE | 3.76 | Prestwick Chemical Inc. | BRD-A87715314 |  |
| PROPACHLOR | 5.00 | MicroSource Discovery Systems Inc. | BRD-K50343025 |  |
| PROPAFENONE | 7.32 | Biomol International Inc. | BRD-A26334849 |  |
| PROPAFENONE HYDROCHLORIDE | 2.65 | Prestwick Chemical Inc. | BRD-A26334849 |  |
| PROPIOMAZINE MALEATE | 5.00 | MicroSource Discovery Systems Inc. | BRD-A10471441 |  |
| PROPOFOL | 5.00 | Biomol International Inc. | BRD-K82255054 |  |
| PROPYLTHIOURACIL | 5.87 | Prestwick Chemical Inc. | BRD-K48168960 |  |
| PROSTAGLANDIN A1 | 0.50 | Biomol International Inc. | BRD-K04010869 |  |
| PROSTAGLANDIN A2 | 0.50 | Biomol International Inc. | BRD-K34782918 |  |
| PSEUDOEPHEDRINE HYDROCHLORIDE | 5.00 | MicroSource Discovery Systems Inc. | BRD-K84175871 |  |
| PURPUROGALLIN | 5.00 | MicroSource Discovery Systems Inc. | BRD-K31023358 |  |
| PYRILAMINE MALEATE | 2.49 | Prestwick Chemical Inc. | BRD-K97564742 |  |
| PYRIMETHAMINE | 4.02 | Prestwick Chemical Inc. | BRD-K88429204 |  |
| PYRITHYLDIONE | 5.98 | Prestwick Chemical Inc. | BRD-K36116267 |  |
| QUERCETINE DIHYDRATE | 2.96 | Prestwick Chemical Inc. | BRD-K97399794 |  |
| QUINACRINE DIHYDROCHLORIDE DIHYDRATE | 1.96 | Prestwick Chemical Inc. | BRD-A45889380 |  |
| QUIPAZINE | 5.00 | Biomol International Inc. | BRD-K77925998 |  |
| QUIPAZINE DIMALEATE SALT | 2.25 | Prestwick Chemical Inc. | BRD-K77925998 |  |
| QUIPAZINE MALEATE | 5.00 | MicroSource Discovery Systems Inc. | BRD-K77925998 |  |
| R(-)-APOMORPHINE | 5.00 | Biomol International Inc. | BRD-K76022557 |  |
| RACEPHEDRINE HYDROCHLORIDE | 5.00 | MicroSource Discovery Systems Inc. | BRD-A54236247 |  |
| RAUWOLSCINE HYDROCHLORIDE | 2.56 | Prestwick Chemical Inc. | BRD-K77474816 |  |
| RESVERATROL | 10.95 | Biomol International Inc. | BRD-K80738081 |  |
| REV-5901 | 0.50 | Biomol International Inc. | BRD-A68281735 |  |
| RILUZOLE | 5.00 | Biomol International Inc. | BRD-K21283037 |  |
| RILUZOLE HYDROCHLORIDE | 3.69 | Prestwick Chemical Inc. | BRD-K21283037 |  |
| RISPERIDONE | 5.00 | Biomol International Inc. | BRD-K53857191 |  |
| RITANSERIN | 5.00 | MicroSource Discovery Systems Inc. | BRD-K40887525 |  |
| RO 20-1724 | 8.98 | Biomol International Inc. | BRD-A07207424 |  |
| RO 31-8220 | 5.46 | Biomol International Inc. | BRD-K06543683 |  |
| ROFECOXIB | 5.00 | MicroSource Discovery Systems Inc. | BRD-K21733600 |  |
| ROPINIROLE HCL | 5.00 | Biomol International Inc. | BRD-K15933101 |  |
| ROSIGLITAZONE | 5.00 | MicroSource Discovery Systems Inc. | BRD-A97437073 |  |
| S,S,S,-TRIBUTYLPHOSPHOROTRITHIOATE | 5.00 | MicroSource Discovery Systems Inc. | BRD-K02837237 |  |
| SALBUTAMOL | 4.18 | Prestwick Chemical Inc. | BRD-A88254928 |  |
| SALICYL ALCOHOL | 5.00 | MicroSource Discovery Systems Inc. | BRD-K08493205 |  |
| SALICYLAMIDE | 5.00 | MicroSource Discovery Systems Inc. | BRD-K81130846 |  |
| SB 202190 | 7.55 | Biomol International Inc. | BRD-K54330070 |  |
| SB-415286 | 6.95 | Biomol International Inc. | BRD-K76805682 |  |
| SCOPOLETIN | 5.20 | Prestwick Chemical Inc. | BRD-K96163925 |  |
| SDZ-201106 | 5.36 | Biomol International Inc. | BRD-A64553394 |  |
| SELEGILINE HYDROCHLORIDE | 4.47 | Prestwick Chemical Inc. | BRD-K86434416 |  |
| SILDENAFIL | 5.00 | MicroSource Discovery Systems Inc. | BRD-K79759585 |  |
| SKF-96365 | 6.82 | Biomol International Inc. | BRD-A72703248 |  |
| SPIPERONE | 2.53 | Prestwick Chemical Inc. | BRD-K55468218 |  |
| STAUROSPORINE | 5.36 | Biomol International Inc. | BRD-K17953061 |  |
| SUCCINYLSULFATHIAZOLE | 2.81 | Prestwick Chemical Inc. | BRD-K01950558 |  |
| SULFABENZAMIDE | 3.62 | Prestwick Chemical Inc. | BRD-K59983611 |  |
| SULFADIAZINE | 4.00 | Prestwick Chemical Inc. | BRD-K32273377 |  |
| SULFADIMETHOXINE | 3.22 | Prestwick Chemical Inc. | BRD-K71125014 |  |
| SULFAMERAZINE | 3.78 | Prestwick Chemical Inc. | BRD-K93524252 |  |
| SULFAMETER | 3.57 | Prestwick Chemical Inc. | BRD-K87492696 |  |
| SULFAMETHAZINE | 5.00 | MicroSource Discovery Systems Inc. | BRD-K11640013 |  |
| SULFAMETHAZINE SODIUM SALT | 3.32 | Prestwick Chemical Inc. | BRD-K11640013 |  |
| SULFAMETHIZOLE | 3.70 | Prestwick Chemical Inc. | BRD-K31682896 |  |
| SULFAMETHOXAZOLE | 3.95 | Prestwick Chemical Inc. | BRD-K28494619 |  |
| SULFAMETHOXYPYRIDAZINE | 3.57 | Prestwick Chemical Inc. | BRD-K00938507 |  |
| SULFANITRAN | 5.00 | MicroSource Discovery Systems Inc. | BRD-K76845197 |  |
| SULFAPHENAZOLE | 3.18 | Prestwick Chemical Inc. | BRD-K10671814 |  |
| SULFAPYRIDINE | 4.01 | Prestwick Chemical Inc. | BRD-K41406082 |  |
| SULFAQUINOXALINE SODIUM SALT | 3.09 | Prestwick Chemical Inc. | BRD-K71133585 |  |
| SULFATHIAZOLE | 3.92 | Prestwick Chemical Inc. | BRD-K14705039 |  |
| SULFINPYRAZONE | 2.47 | Prestwick Chemical Inc. | BRD-A36217750 |  |
| SULFISOXAZOLE | 3.74 | Prestwick Chemical Inc. | BRD-K50859149 |  |
| SULINDAC | 2.81 | Prestwick Chemical Inc. | BRD-A13946108 |  |
| SUMATRIPTAN | 5.00 | MicroSource Discovery Systems Inc. | BRD-K50938287 |  |
| SUXIBUZONE | 2.28 | Prestwick Chemical Inc. | BRD-K78815826 |  |
| TACRINE HYDROCHLORIDE HYDRATE | 4.26 | Prestwick Chemical Inc. | BRD-K81473089 |  |
| TAMOXIFEN CITRATE | 1.77 | Prestwick Chemical Inc. | BRD-K93754473 |  |
| TEGASEROD | 5.00 | MicroSource Discovery Systems Inc. | BRD-K88743730 |  |
| TENIPOSIDE | 5.00 | MicroSource Discovery Systems Inc. | BRD-A35588707 |  |
| TENOXICAM | 2.96 | Prestwick Chemical Inc. | BRD-A22844106 |  |
| TERBUTALINE HEMISULFATE | 5.00 | MicroSource Discovery Systems Inc. | BRD-A50157456 |  |
| TERFENADINE | 2.12 | Prestwick Chemical Inc. | BRD-A06352418 |  |
| TETRACAINE HYDROCHLORIDE | 3.32 | Prestwick Chemical Inc. | BRD-K45071273 |  |
| TETRAHYDROPALMATINE | 5.00 | MicroSource Discovery Systems Inc. | BRD-A43940795 |  |
| TETRAHYDROZOLINE HYDROCHLORIDE | 4.22 | Prestwick Chemical Inc. | BRD-A28856712 |  |
| TETRANDRINE | 4.01 | Biomol International Inc. | BRD-K08078237 |  |
| THEOPHYLLINE | 5.00 | MicroSource Discovery Systems Inc. | BRD-K97799481 |  |
| THIAMPHENICOL | 2.81 | Prestwick Chemical Inc. | BRD-K79711234 |  |
| THIAMYLAL SODIUM | 5.00 | MicroSource Discovery Systems Inc. | BRD-A23970436 |  |
| THIMEROSAL | 5.00 | MicroSource Discovery Systems Inc. | BRD-K61443650 |  |
| THIOTHIXENE | 5.00 | MicroSource Discovery Systems Inc. | BRD-K97309399 |  |
| THONZYLAMINE HYDROCHLORIDE | 5.00 | MicroSource Discovery Systems Inc. | BRD-K88405679 |  |
| TIABENDAZOLE | 4.97 | Prestwick Chemical Inc. | BRD-K77695569 |  |
| TIAPRIDE HYDROCHLORIDE | 2.74 | Prestwick Chemical Inc. | BRD-K57432881 |  |
| TICLOPIDINE HYDROCHLORIDE | 3.33 | Prestwick Chemical Inc. | BRD-K00603606 |  |
| TIMOLOL MALEATE | 5.00 | MicroSource Discovery Systems Inc. | BRD-K30421593 |  |
| TINIDAZOLE | 4.04 | Prestwick Chemical Inc. | BRD-K89125793 |  |
| TODRALAZINE HYDROCHLORIDE | 3.72 | Prestwick Chemical Inc. | BRD-K68553471 |  |
| TOLAZAMIDE | 8.03 | Biomol International Inc. | BRD-K32164935 |  |
| TOLAZOLINE HYDROCHLORIDE | 5.08 | Prestwick Chemical Inc. | BRD-K46211610 |  |
| TOLBUTAMIDE | 9.25 | Biomol International Inc. | BRD-K85119730 |  |
| TOLMETIN SODIUM | 5.00 | MicroSource Discovery Systems Inc. | BRD-K82562631 |  |
| TOLNAFTATE | 3.25 | Prestwick Chemical Inc. | BRD-K44273375 |  |
| TOLPERISONE HYDROCHLORIDE | 5.00 | MicroSource Discovery Systems Inc. | BRD-A27732521 |  |
| TRANYLCYPROMINE SULFATE | 5.00 | MicroSource Discovery Systems Inc. | BRD-A43974575 |  |
| TRAZODONE HYDROCHLORIDE | 2.45 | Prestwick Chemical Inc. | BRD-K70778732 |  |
| TRIALLATE | 5.00 | MicroSource Discovery Systems Inc. | BRD-K64698045 |  |
| TRIAMCINOLONE ACETONIDE | 5.00 | MicroSource Discovery Systems Inc. | BRD-A92439610 |  |
| TRIAMTERENE | 3.95 | Prestwick Chemical Inc. | BRD-K92049597 |  |
| TRIFLUOPERAZINE | 5.00 | Biomol International Inc. | BRD-K89732114 |  |
| TRIFLUOPERAZINE DIHYDROCHLORIDE | 2.08 | Prestwick Chemical Inc. | BRD-K89732114 |  |
| TRIHEXYPHENIDYL-D,L HYDROCHLORIDE | 2.96 | Prestwick Chemical Inc. | BRD-A48180038 |  |
| TRIM | 11.78 | Biomol International Inc. | BRD-K36851334 |  |
| TRIMETHOBENZAMIDE HYDROCHLORIDE | 2.35 | Prestwick Chemical Inc. | BRD-K34415467 |  |
| TRIMETHOPRIM | 3.44 | Prestwick Chemical Inc. | BRD-K07208025 |  |
| TRIMIPRAMINE MALEATE SALT | 2.44 | Prestwick Chemical Inc. | BRD-A19195498 |  |
| TRIPELENNAMINE CITRATE | 5.00 | MicroSource Discovery Systems Inc. | BRD-K57033106 |  |
| TRIPROLIDINE HYDROCHLORIDE | 3.18 | Prestwick Chemical Inc. | BRD-K11742128 |  |
| TRIPTOLIDE | 6.94 | Biomol International Inc. | BRD-K39484304 |  |
| TROPICAMIDE | 3.52 | Prestwick Chemical Inc. | BRD-A79672927 |  |
| TROPISETRON | 5.00 | Biomol International Inc. | BRD-A83859836 |  |
| TTNPB | 0.50 | Biomol International Inc. | BRD-K49685476 |  |
| TULOBUTEROL | 5.00 | MicroSource Discovery Systems Inc. | BRD-A37441042 |  |
| TYRPHOSTIN 1 | 13.57 | Biomol International Inc. | BRD-K41996876 |  |
| TYRPHOSTIN 46 | 5.00 | Biomol International Inc. | BRD-K60184833 |  |
| U-0126 | 5.00 | Biomol International Inc. | BRD-K18787491 |  |
| U-74389G | 4.09 | Biomol International Inc. | BRD-A96897502 |  |
| U-99194A | 5.00 | Biomol International Inc. | BRD-K70281171 |  |
| UMBELLIFERONE | 5.00 | MicroSource Discovery Systems Inc. | BRD-K87991767 |  |
| VALACYCLOVIR HYDROCHLORIDE | 5.00 | MicroSource Discovery Systems Inc. | BRD-K46435977 |  |
| VALDECOXIB | 5.00 | MicroSource Discovery Systems Inc. | BRD-K12994359 |  |
| VERAPAMIL | 5.50 | Biomol International Inc. | BRD-A09533288 |  |
| VERAPAMYL HYDROCHLORIDE | 2.04 | Prestwick Chemical Inc. | BRD-A09533288 |  |
| VESAMICOL HYDROCHLORIDE | 5.00 | MicroSource Discovery Systems Inc. | BRD-A76904477 |  |
| VINPOCETINE | 2.85 | Prestwick Chemical Inc. | BRD-K53318339 |  |
| VULPINIC ACID | 5.00 | MicroSource Discovery Systems Inc. | BRD-A89237309 |  |
| WY-14643 | 0.50 | Biomol International Inc. | BRD-K01902415 |  |
| XYLAZINE | 4.54 | Prestwick Chemical Inc. | BRD-K21565985 |  |
| YOHIMBINE HYDROCHLORIDE | 5.00 | MicroSource Discovery Systems Inc. | BRD-A87445400 |  |
| YS035 | 6.95 | Biomol International Inc. | BRD-K06208435 |  |
| ZAPRINAST | 9.22 | Biomol International Inc. | BRD-K16542329 |  |
| ZARDAVERINE | 9.32 | Biomol International Inc. | BRD-K37561857 |  |
| ZIDOVUDINE, AZT | 3.74 | Prestwick Chemical Inc. | BRD-K72903603 |  |
| ZM336372 | 6.42 | Biomol International Inc. | BRD-K73789395 |  |
| ZOLPIDEM | 5.00 | MicroSource Discovery Systems Inc. | BRD-K44876623 |  |
| ZOMEPIRAC SODIUM | 5.00 | MicroSource Discovery Systems Inc. | BRD-K81326768 |  |
| ZOMEPIRAC SODIUM SALT | 3.18 | Prestwick Chemical Inc. | BRD-K81326768 |  |
