# Supplementary material for: Multiplex Cytological Profiling Assay to Measure Diverse Cellular States
Source: PLoS One. 2013 Dec 2;8(12):e80999. doi: 10.1371/journal.pone.0080999 (PMC3847047; doi:10.1371/journal.pone.0080999)
Supplement: Table S7 — The compounds that were both active and annotated. (DOCX) [file pone.0080999.s015.docx]

**Table S7:** Compounds that were both active and annotated.

| Compound name | Concentration [µM] | Source | Broad ID | Structure |
| --- | --- | --- | --- | --- |
| (D,L)-TETRAHYDROBERBERINE | 2.95 | Prestwick Chemical Inc. | BRD-A69950438 | 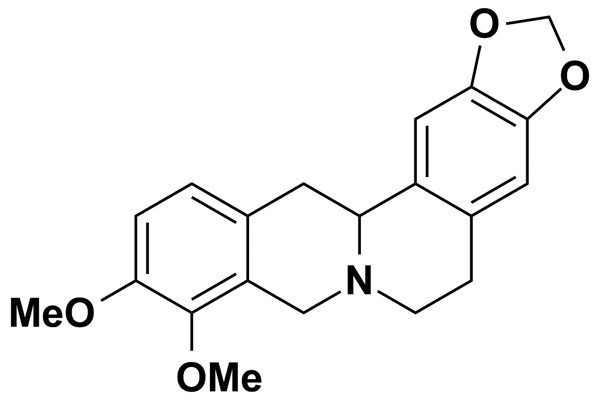 |
| 2,5-DITERTBUTYLHYDROQUINONE | 11.24 | Biomol International Inc. | BRD-K95603879 | 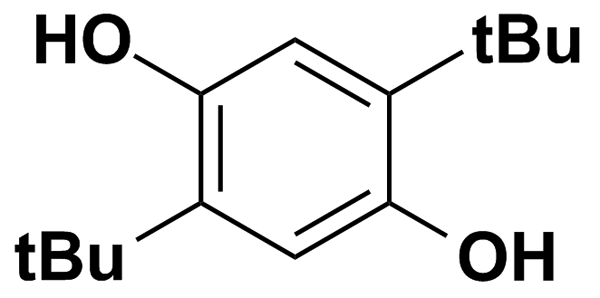 |
| AG-879 | 7.90 | Biomol International Inc. | BRD-K59469039 | 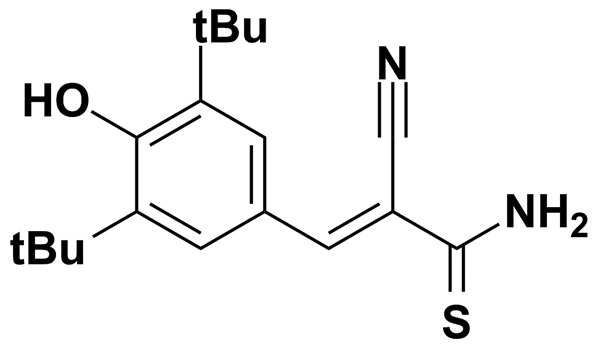 |
| ANISOMYCIN | 9.42 | Biomol International Inc. | BRD-K91370081 | 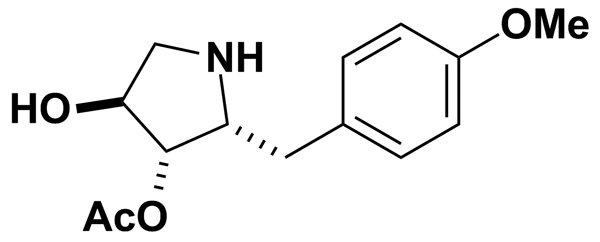 |
| BAY 11-7082 | 12.06 | Biomol International Inc. | BRD-K15025317 | 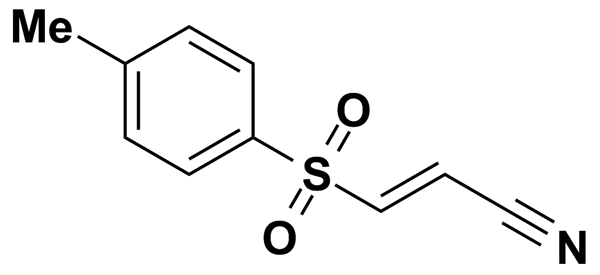 |
| BW-B 70C | 7.90 | Biomol International Inc. | BRD-A55946879 | 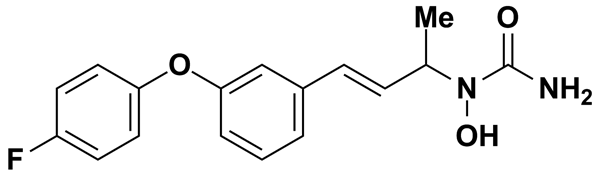 |
| CA-074-ME | 6.29 | Biomol International Inc. | BRD-A56020723 | 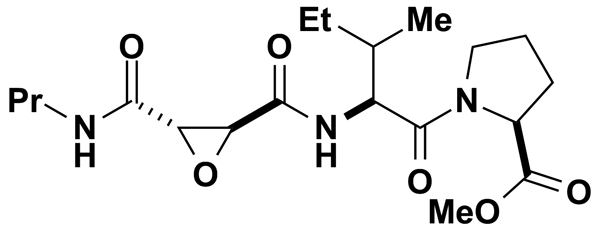 |
| CELECOXIB | 5.00 | MicroSource Discovery Systems Inc. | BRD-K02637541 | 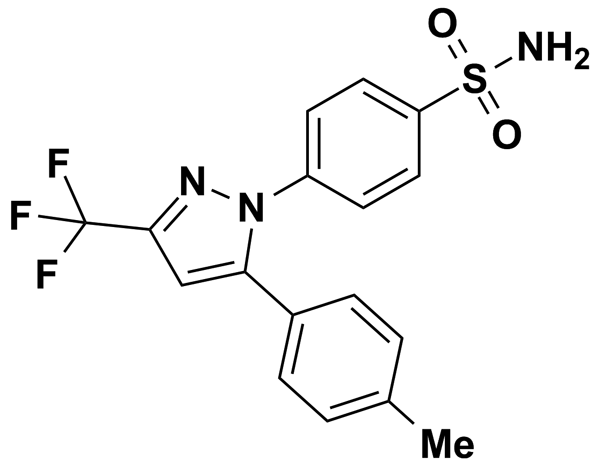 |
| CERULENIN | 11.20 | Biomol International Inc. | BRD-K52075040 | 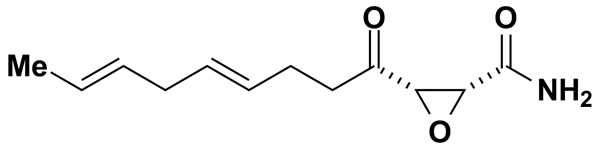 |
| COLCHICINE | 2.50 | Prestwick Chemical Inc. | BRD-K00259736 | 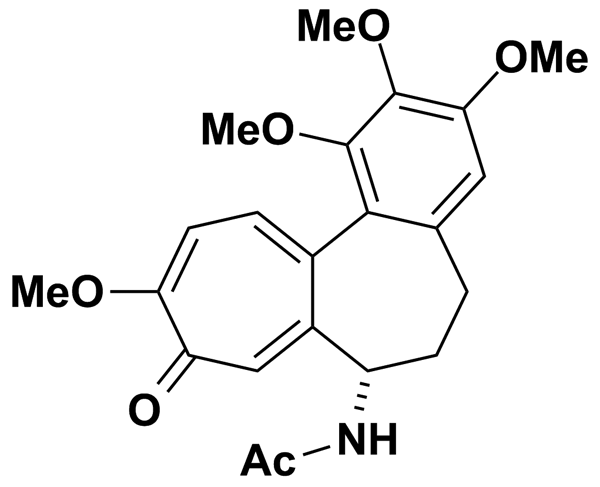 |
| CYCLIZINE | 5.00 | MicroSource Discovery Systems Inc. | BRD-K79501723 | 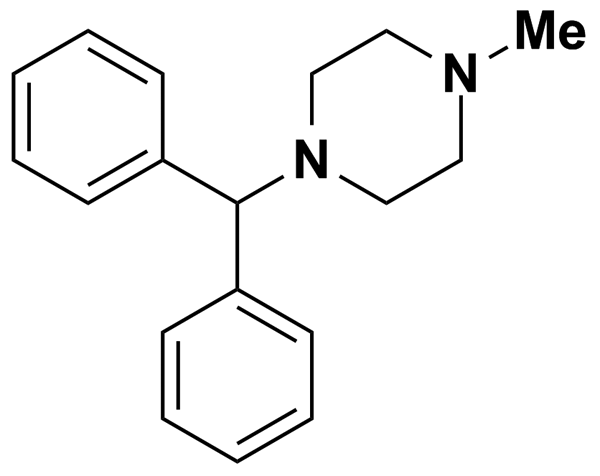 |
| CYCLIZINE HYDROCHLORIDE | 3.30 | Prestwick Chemical Inc. | BRD-K79501723 | 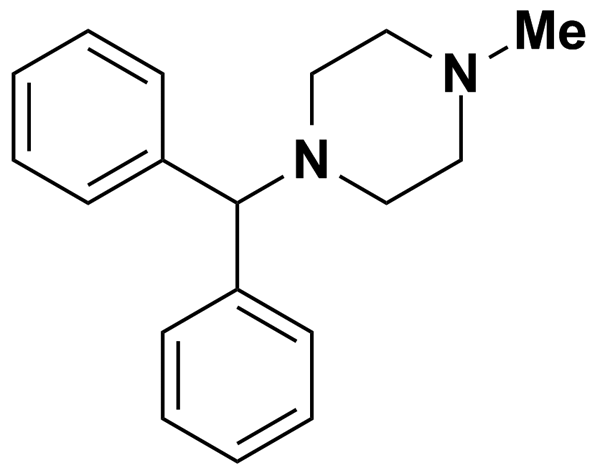 |
| DAUNORUBICIN HYDROCHLORIDE | 1.77 | Prestwick Chemical Inc. | BRD-K43389675 | 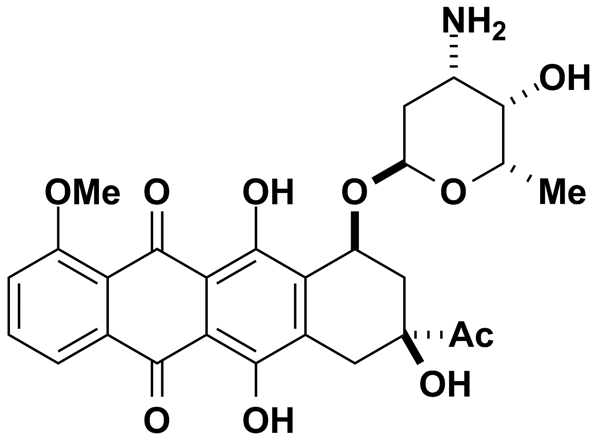 |
| DIGITOXIN | 5.00 | MicroSource Discovery Systems Inc. | BRD-A93236127 | 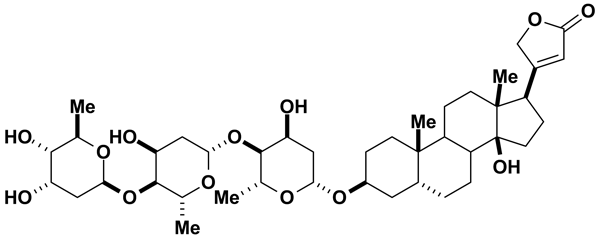 |
| DIGOXIN | 5.00 | MicroSource Discovery Systems Inc. | BRD-A94756469 | 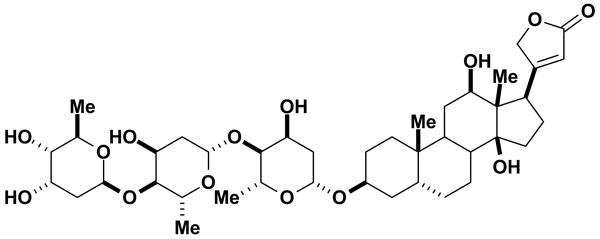 |
| DISULFIRAM | 3.37 | Prestwick Chemical Inc. | BRD-K32744045 | 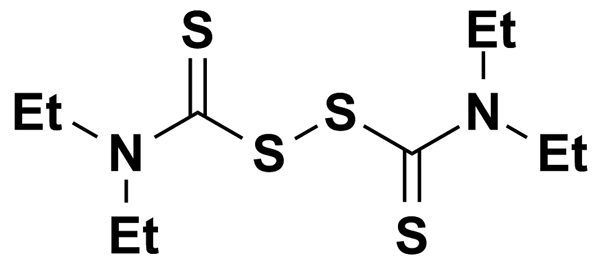 |
| ELLIPTICINE | 4.06 | Prestwick Chemical Inc. | BRD-K85985071 | 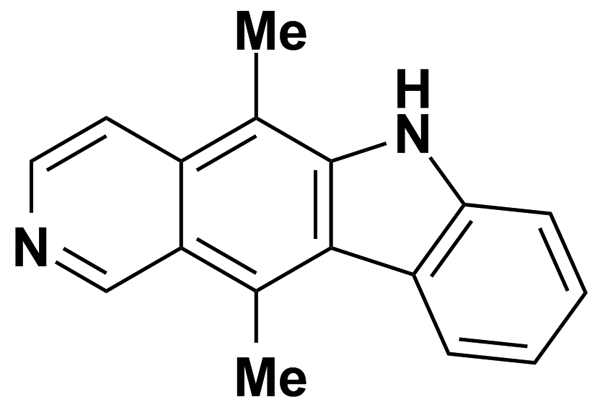 |
| ESTRADIOL | 9.18 | Biomol International Inc. | BRD-K18910433 | 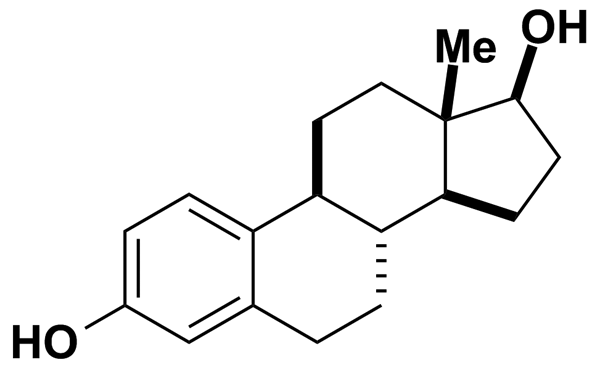 |
| ETHINYL ESTRADIOL | 5.00 | MicroSource Discovery Systems Inc. | BRD-A02367930 | 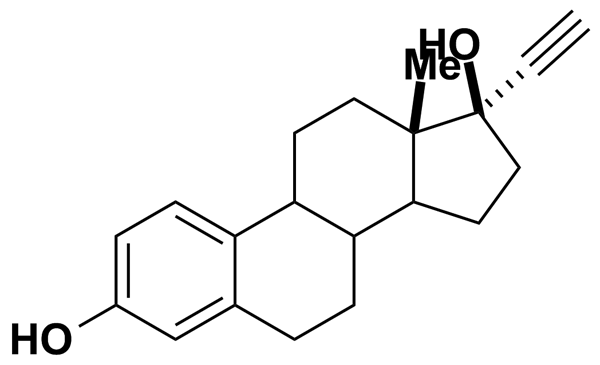 |
| ETOPOSIDE | 1.70 | Prestwick Chemical Inc. | BRD-K37798499 | 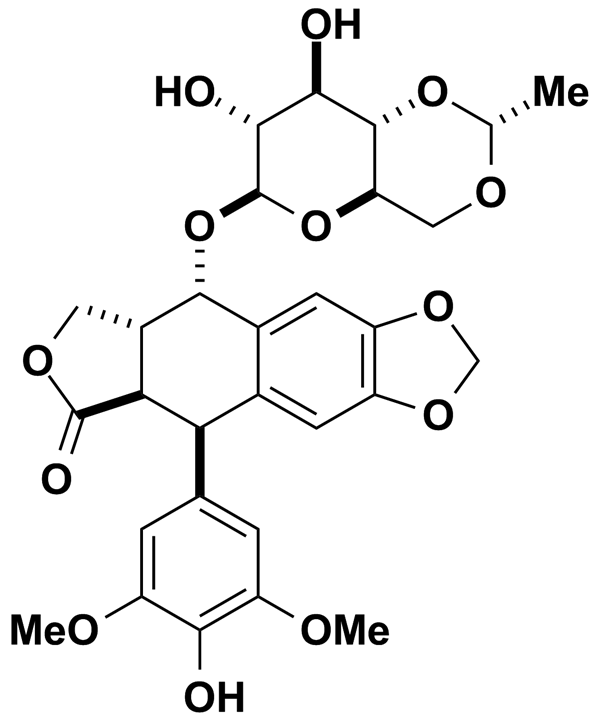 |
| FCCP | 9.84 | Biomol International Inc. | BRD-K14821540 | 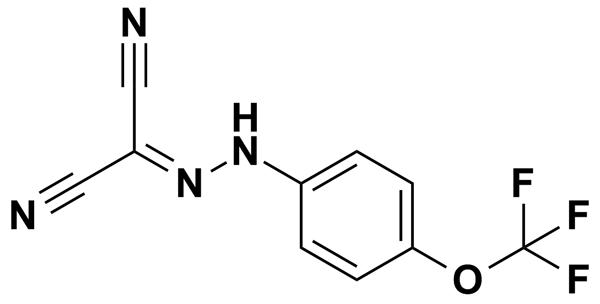 |
| FENBENDAZOLE | 3.34 | Prestwick Chemical Inc. | BRD-K51318897 | 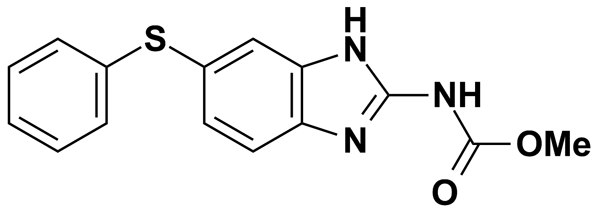 |
| FENDILINE HYDROCHLORIDE | 2.84 | Prestwick Chemical Inc. | BRD-A71033472 | 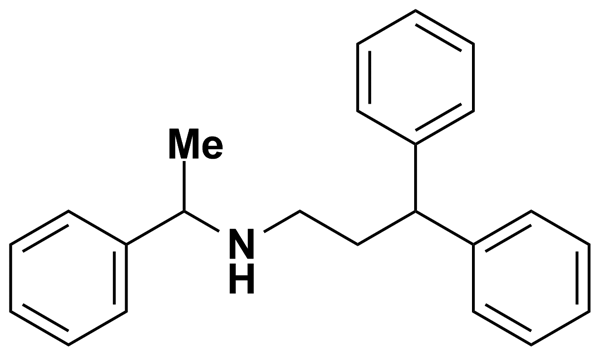 |
| FLUPHENAZINE | 5.00 | Biomol International Inc. | BRD-K55127134 | 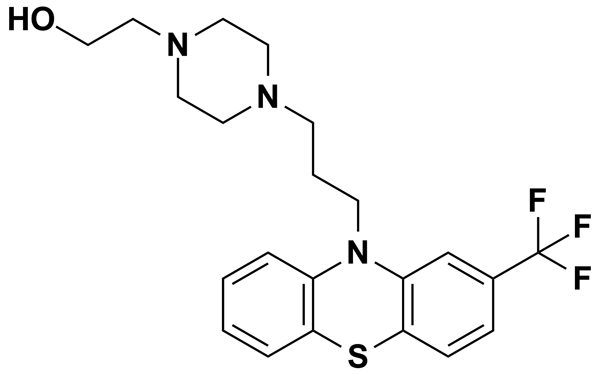 |
| FLUPHENAZINE DIHYDROCHLORIDE | 1.96 | Prestwick Chemical Inc. | BRD-K55127134 | 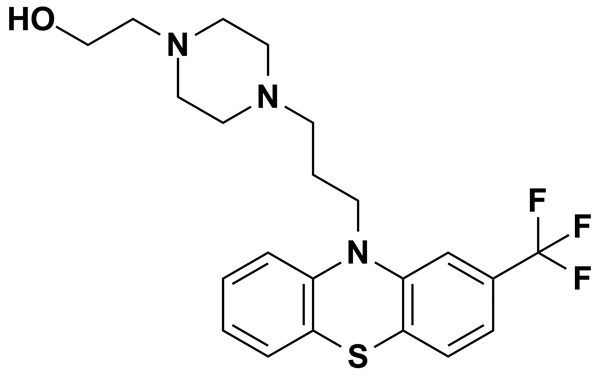 |
| GBR 12935 | 5.00 | Biomol International Inc. | BRD-K50135270 | 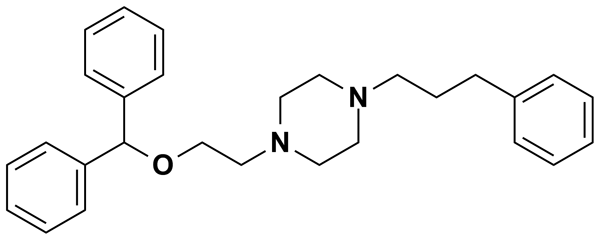 |
| GENTIAN VIOLET | 5.00 | MicroSource Discovery Systems Inc. | BRD-K60025295 | 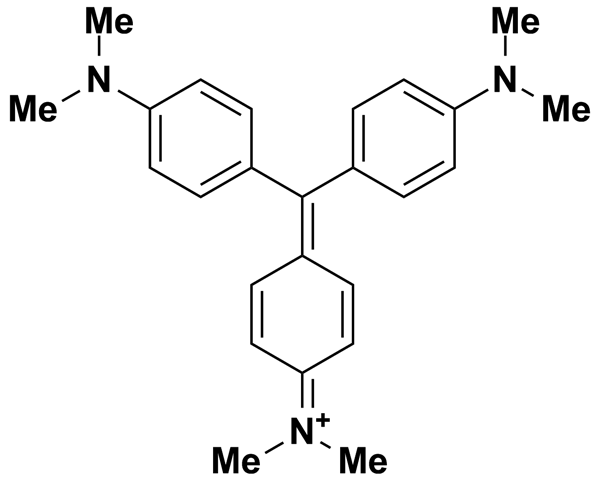 |
| GF-109203X | 6.06 | Biomol International Inc. | BRD-K31342827 | 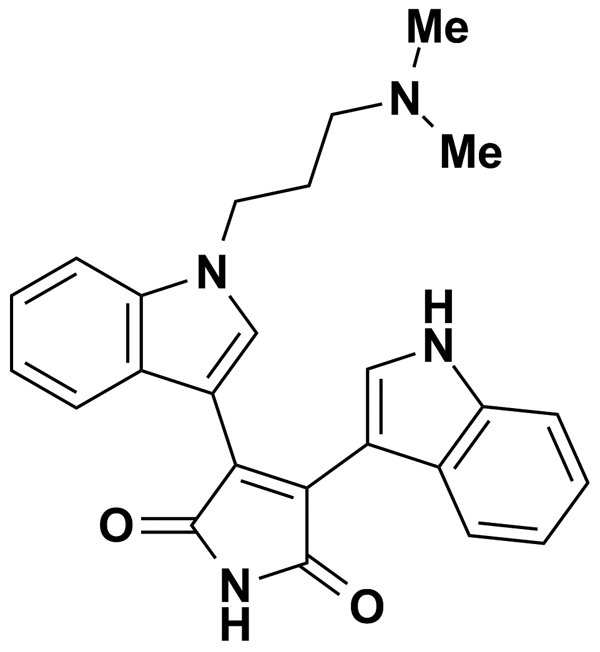 |
| GO6976 | 6.61 | Biomol International Inc. | BRD-K59304176 | 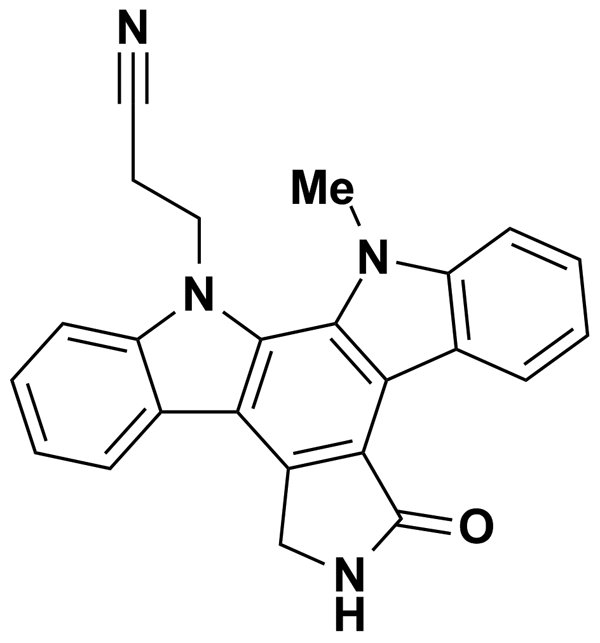 |
| HYCANTHONE | 2.81 | Prestwick Chemical Inc. | BRD-K50406511 | 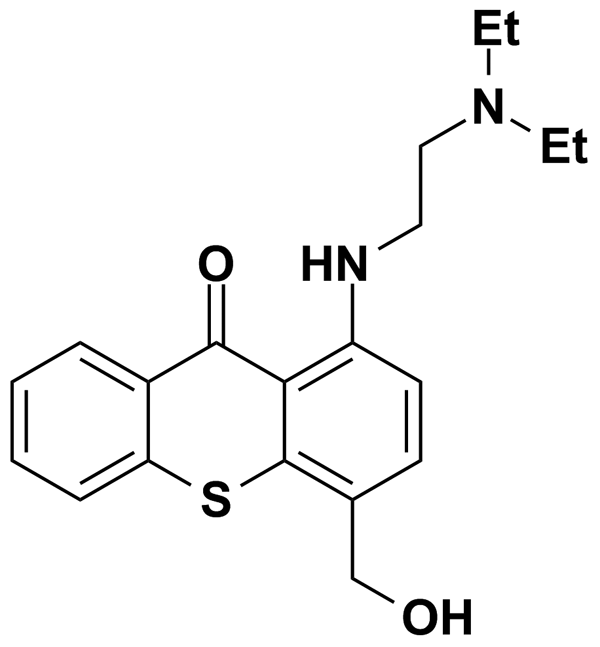 |
| HYDROCHLOROTHIAZIDE | 3.36 | Prestwick Chemical Inc. | BRD-K13078532 | 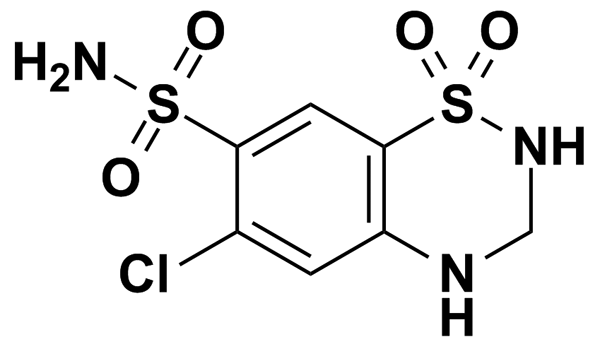 |
| KINETIN RIBOSIDE | 5.00 | MicroSource Discovery Systems Inc. | BRD-K94325918 | 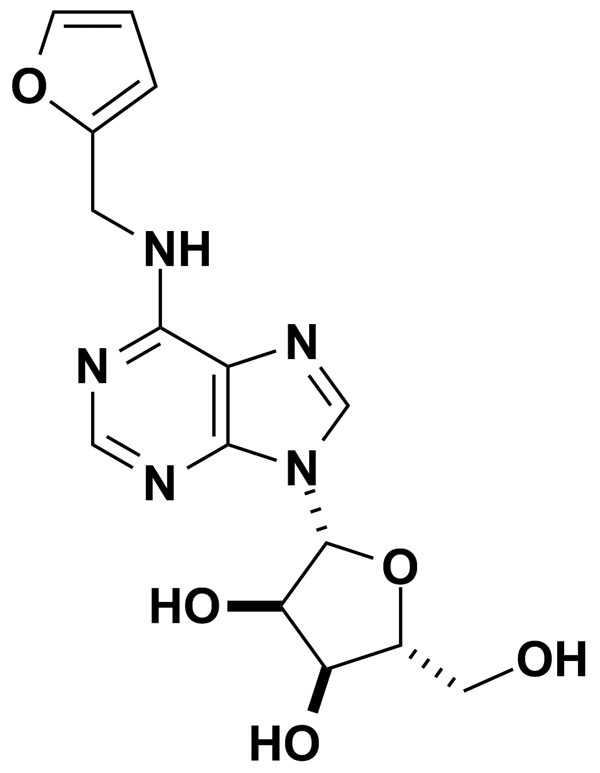 |
| LANATOSIDE C | 5.00 | MicroSource Discovery Systems Inc. | BRD-A64242993 | 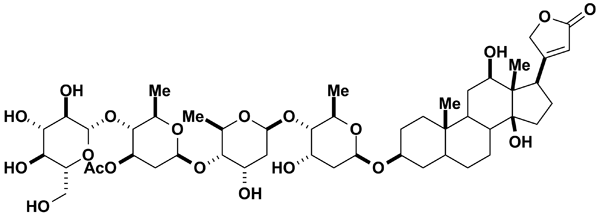 |
| LAPACHOL | 5.00 | MicroSource Discovery Systems Inc. | BRD-A06912736 | 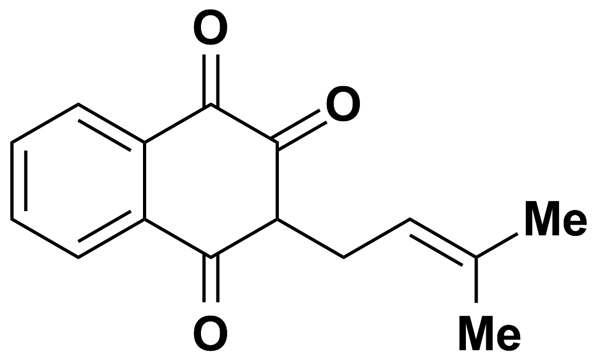 |
| LEVAMISOLE HYDROCHLORIDE | 5.00 | MicroSource Discovery Systems Inc. | BRD-K73107279 | 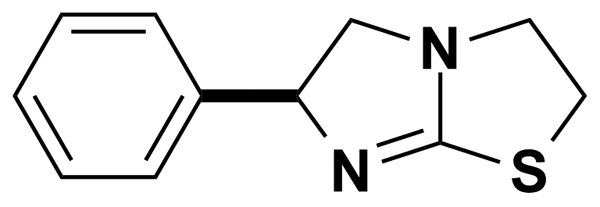 |
| LY-294002 | 8.13 | Biomol International Inc. | BRD-K27305650 | 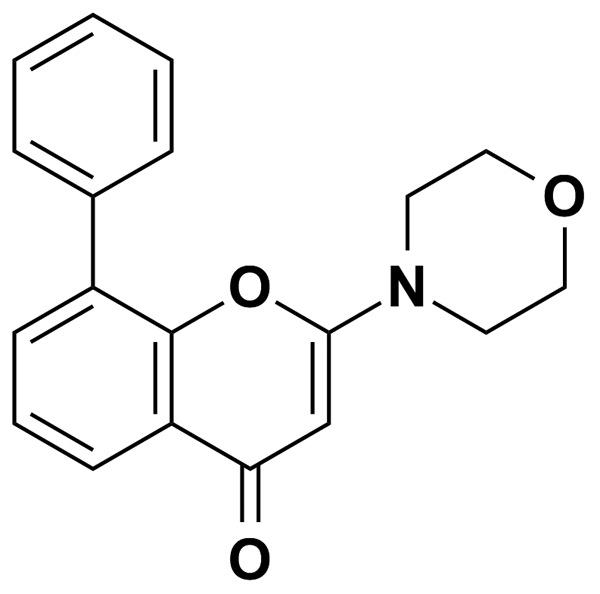 |
| LY-83583 | 9.99 | Biomol International Inc. | BRD-K62792802 | 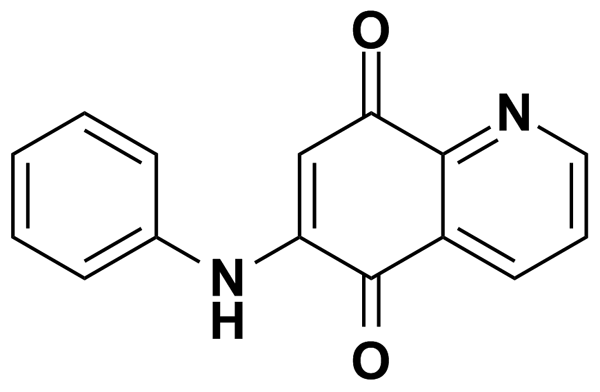 |
| LYCORINE | 5.00 | MicroSource Discovery Systems Inc. | BRD-A10335634 | 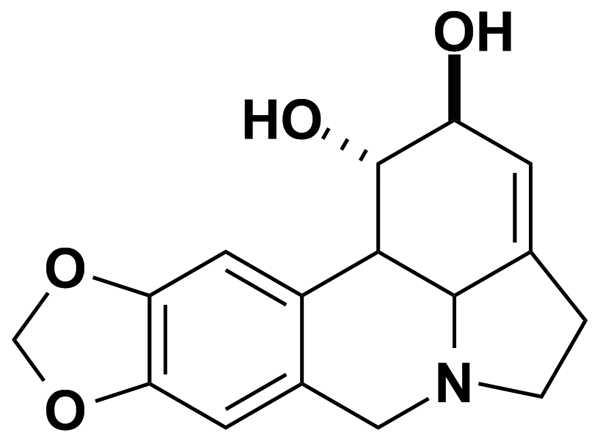 |
| MCI-186 | 14.35 | Biomol International Inc. | BRD-K35458079 | 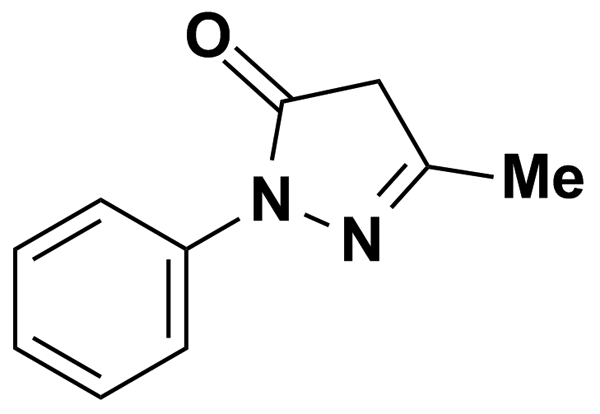 |
| MELPHALAN | 5.00 | MicroSource Discovery Systems Inc. | BRD-K87827419 | 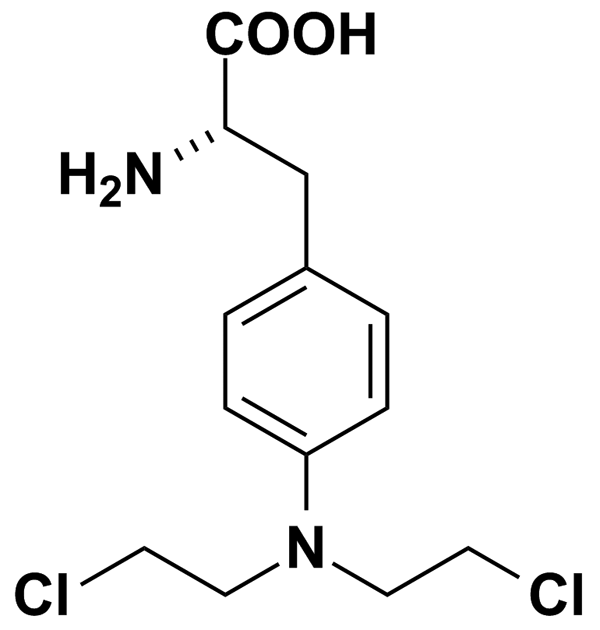 |
| METOCLOPRAMIDE | 5.00 | Biomol International Inc. | BRD-K75641298 | 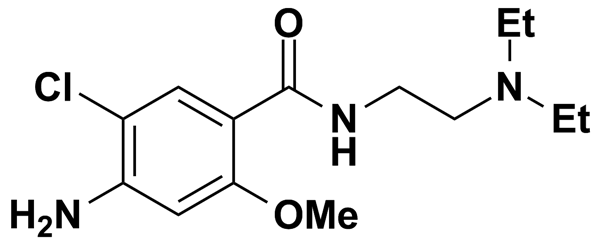 |
| METOCLOPRAMIDE MONOHYDROCHLORIDE | 2.97 | Prestwick Chemical Inc. | BRD-K75641298 | 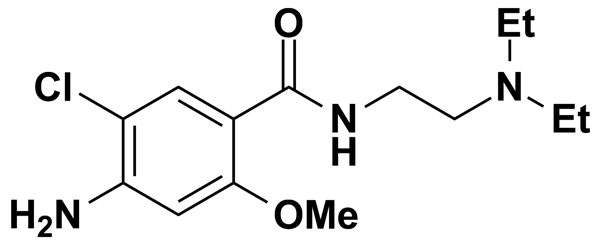 |
| MITOXANTRONE DIHYDROCHLORIDE | 1.93 | Prestwick Chemical Inc. | BRD-K21680192 | 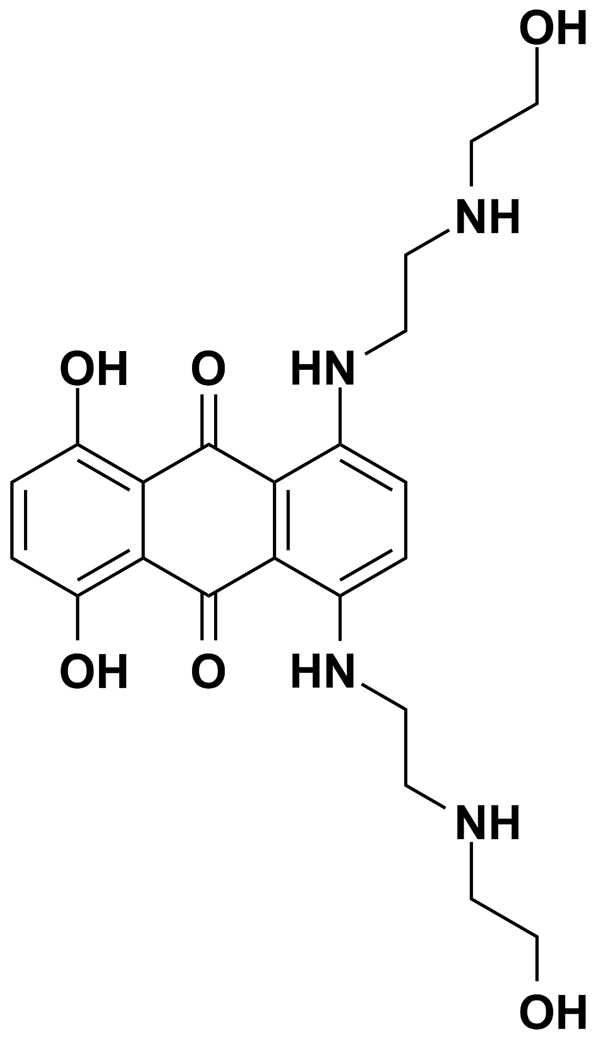 |
| NERIIFOLIN | 5.00 | MicroSource Discovery Systems Inc. | BRD-A31385885 | 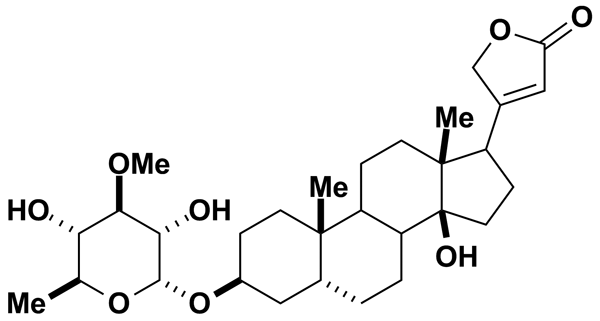 |
| NIFLUMIC ACID | 8.86 | Biomol International Inc. | BRD-K98763141 | 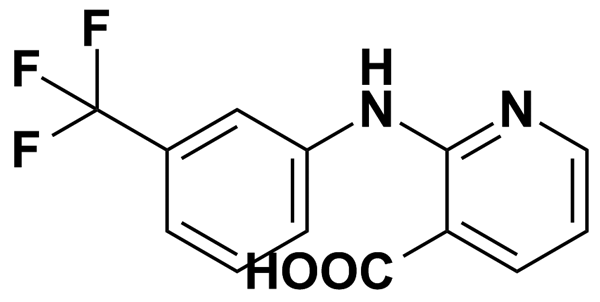 |
| NITRENDIPINE | 6.94 | Biomol International Inc. | BRD-A02006392 | 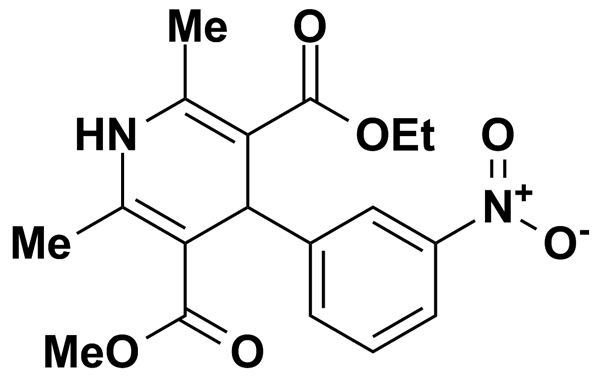 |
| NOVOBIOCIN SODIUM | 5.00 | MicroSource Discovery Systems Inc. | BRD-K85307935 | 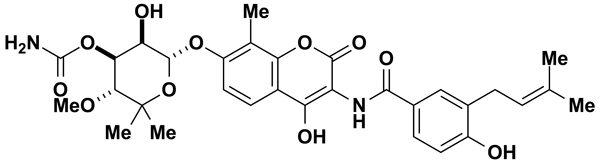 |
| NSC-95397 | 8.05 | Biomol International Inc. | BRD-K68143200 | 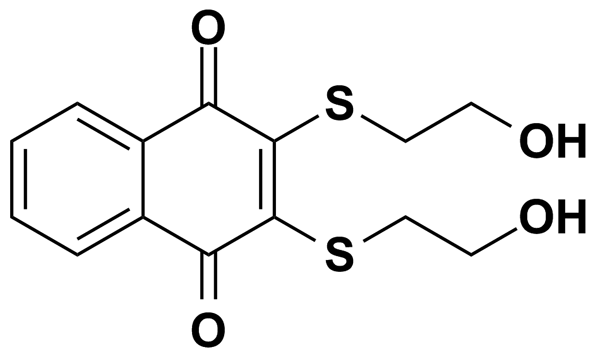 |
| OXIBENDAZOLE | 5.00 | MicroSource Discovery Systems Inc. | BRD-K52075715 | 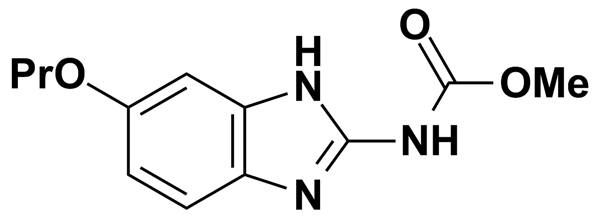 |
| OXYPHENBUTAZONE | 3.08 | Prestwick Chemical Inc. | BRD-A33749298 | 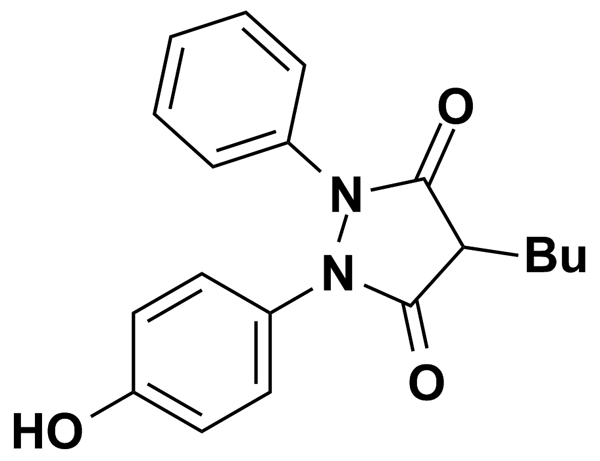 |
| OXYQUINOLINE HEMISULFATE | 5.00 | MicroSource Discovery Systems Inc. | BRD-K66808046 | 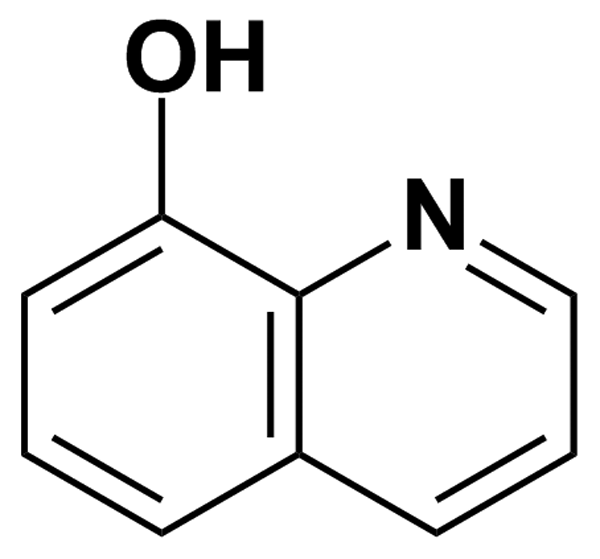 |
| PACLITAXEL | 1.17 | Prestwick Chemical Inc. | BRD-K62008436 | 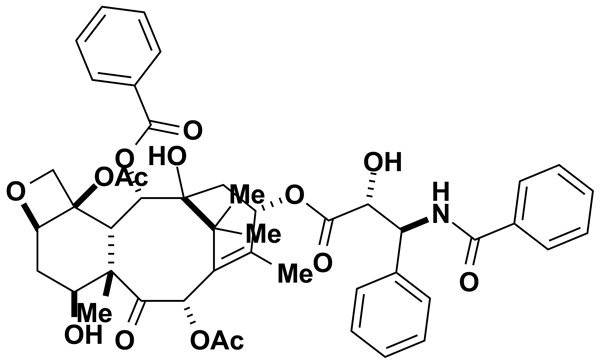 |
| PCA 4248 | 6.92 | Biomol International Inc. | BRD-A29289453 | 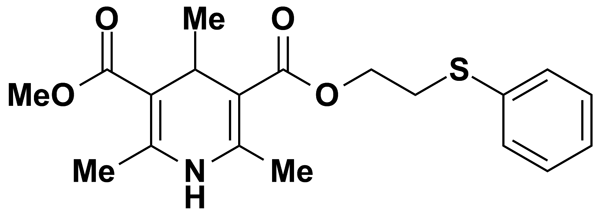 |
| PENICILLIN V POTASSIUM | 5.00 | MicroSource Discovery Systems Inc. | BRD-K43966364 | 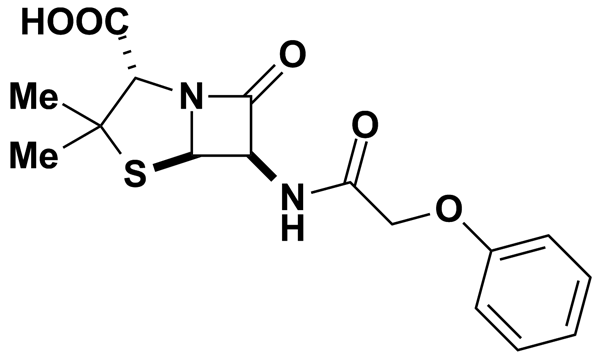 |
| PENITREM A | 3.94 | Biomol International Inc. | BRD-K03842655 | 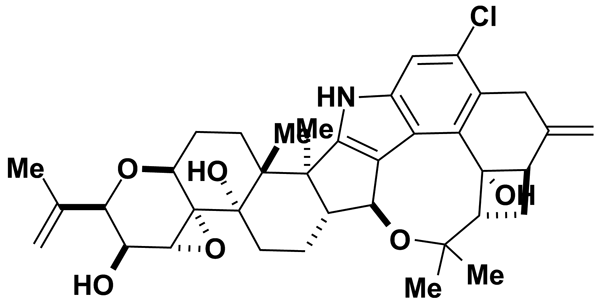 |
| PERUVOSIDE | 5.00 | MicroSource Discovery Systems Inc. | BRD-A57089740 | 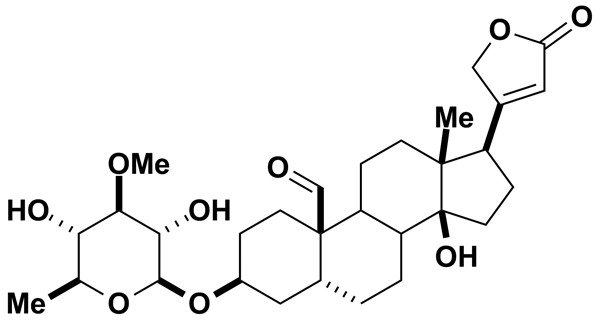 |
| PHENACEMIDE | 5.00 | MicroSource Discovery Systems Inc. | BRD-K40905133 | 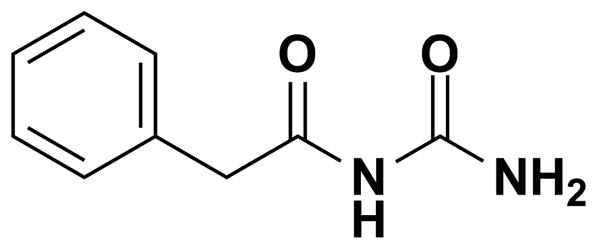 |
| PIPERLONGUMINE | 3.15 | Prestwick Chemical Inc. | BRD-K24132293 | 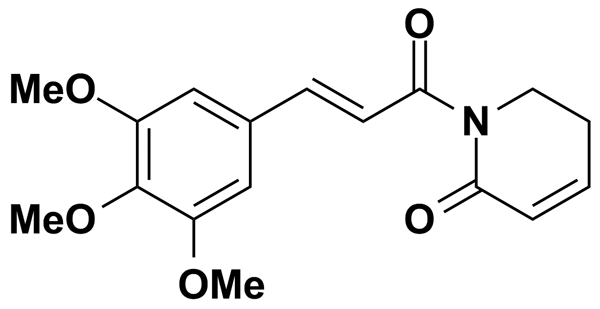 |
| PROCAINE HYDROCHLORIDE | 3.67 | Prestwick Chemical Inc. | BRD-K24616672 | 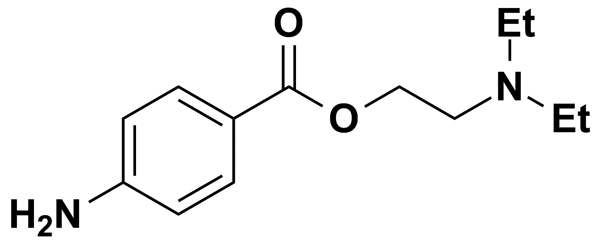 |
| PROPAFENONE | 7.32 | Biomol International Inc. | BRD-A26334849 | 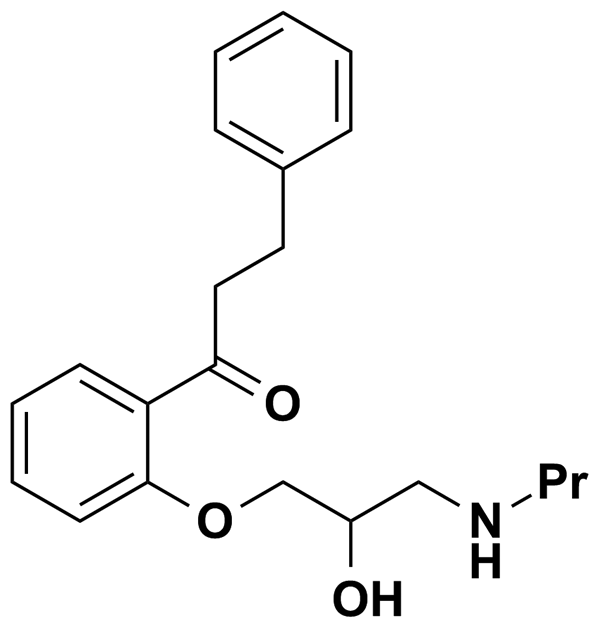 |
| PROPAFENONE HYDROCHLORIDE | 2.65 | Prestwick Chemical Inc. | BRD-A26334849 | 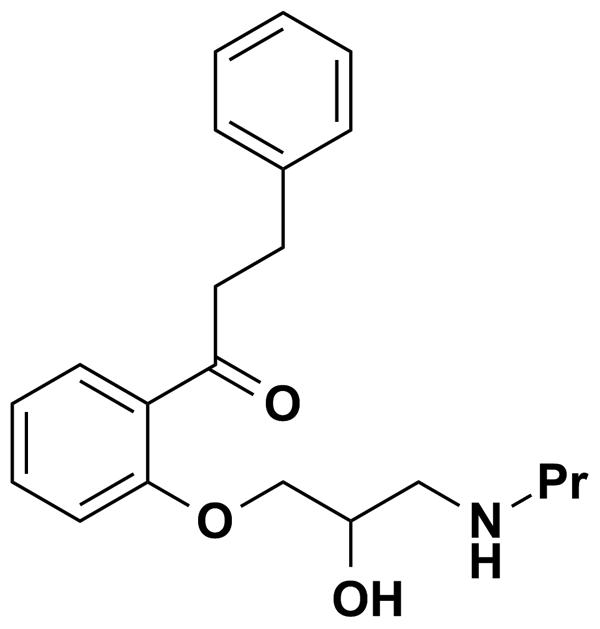 |
| PROPIOMAZINE MALEATE | 5.00 | MicroSource Discovery Systems Inc. | BRD-A10471441 | 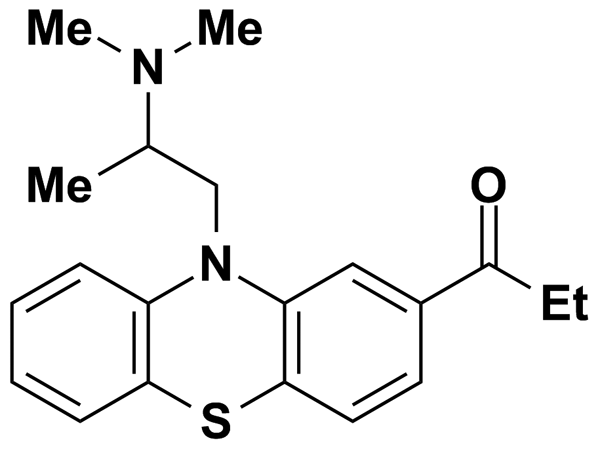 |
| QUINACRINE DIHYDROCHLORIDE DIHYDRATE | 1.96 | Prestwick Chemical Inc. | BRD-A45889380 | 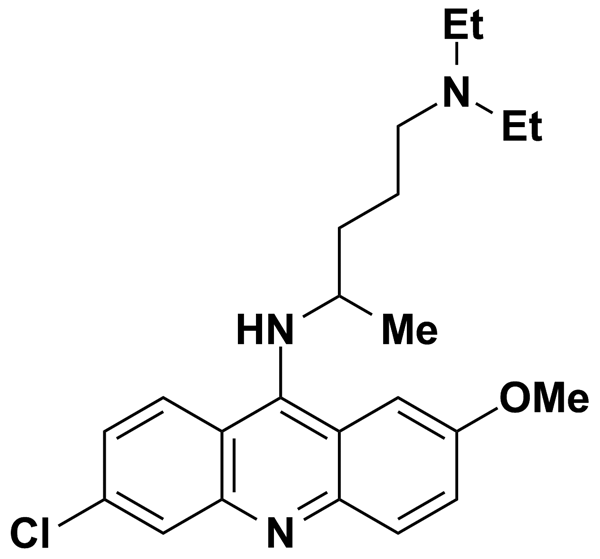 |
| R(-)-APOMORPHINE | 5.00 | Biomol International Inc. | BRD-K76022557 | 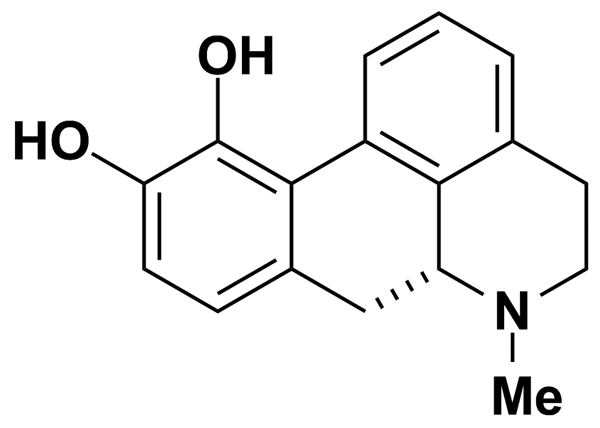 |
| RESVERATROL | 10.95 | Biomol International Inc. | BRD-K80738081 | 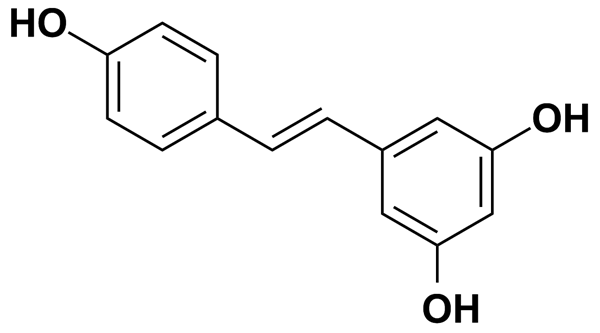 |
| RO 31-8220 | 5.46 | Biomol International Inc. | BRD-K06543683 | 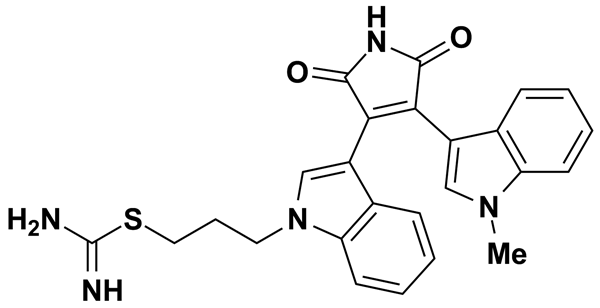 |
| SDZ-201106 | 5.36 | Biomol International Inc. | BRD-A64553394 | 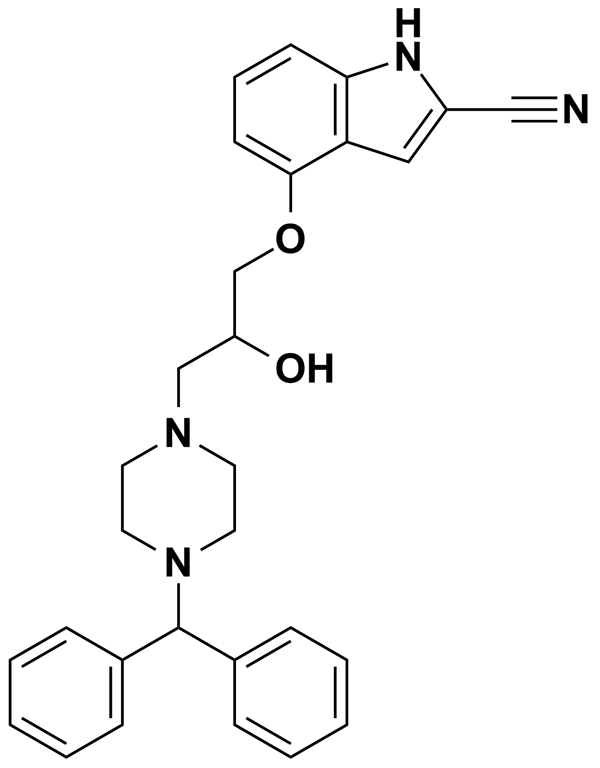 |
| SKF-96365 | 6.82 | Biomol International Inc. | BRD-A72703248 | 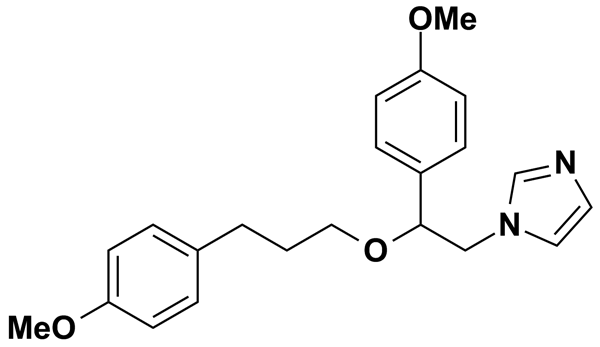 |
| STAUROSPORINE | 5.36 | Biomol International Inc. | BRD-K17953061 | 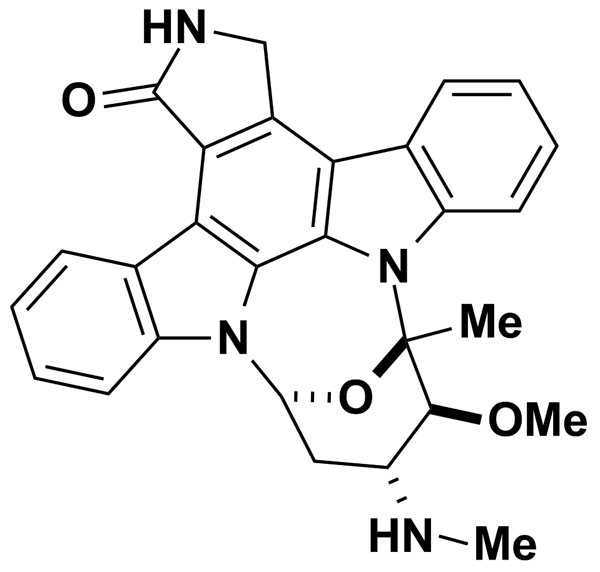 |
| TACRINE HYDROCHLORIDE HYDRATE | 4.26 | Prestwick Chemical Inc. | BRD-K81473089 | 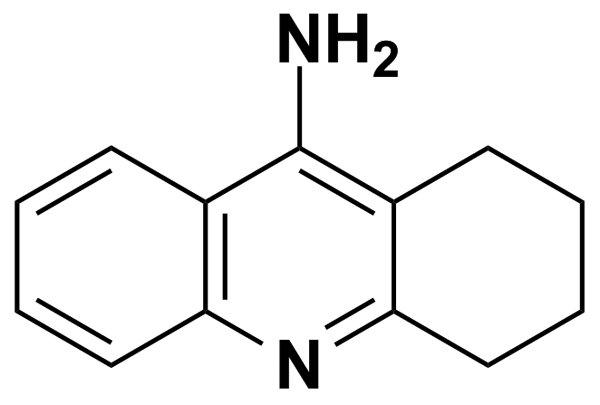 |
| TENIPOSIDE | 5.00 | MicroSource Discovery Systems Inc. | BRD-A35588707 | 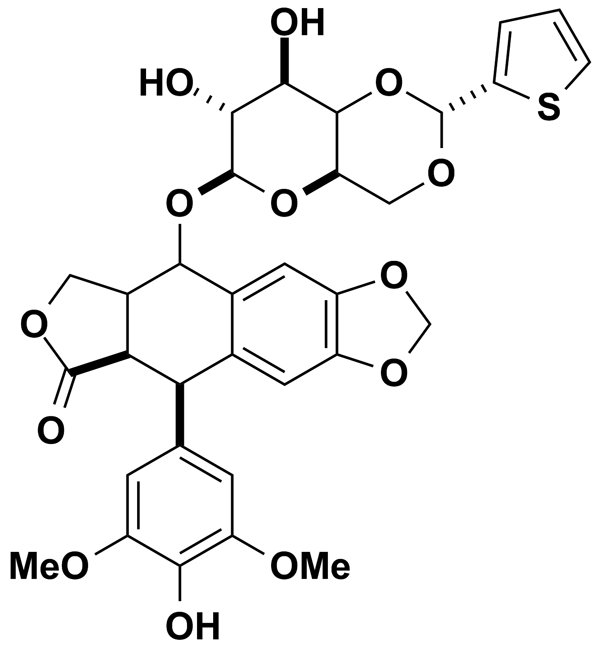 |
| TERFENADINE | 2.12 | Prestwick Chemical Inc. | BRD-A06352418 | 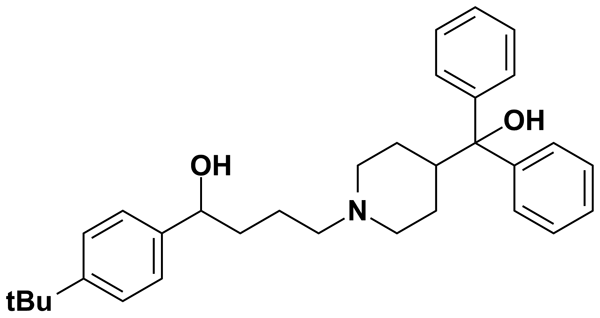 |
| TETRAHYDROPALMATINE | 5.00 | MicroSource Discovery Systems Inc. | BRD-A43940795 | 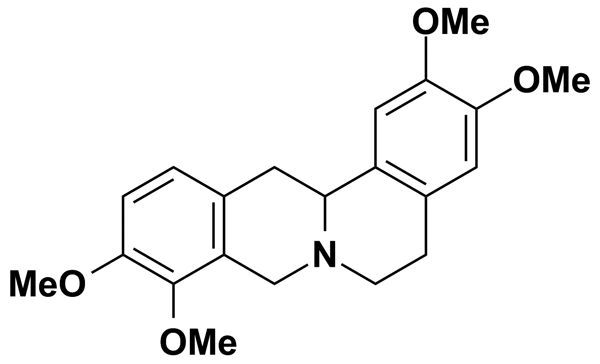 |
| TETRANDRINE | 4.01 | Biomol International Inc. | BRD-K08078237 | 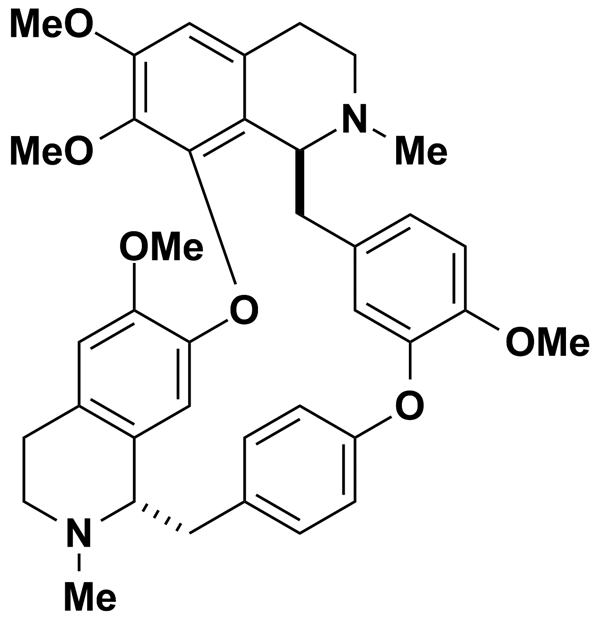 |
| THIMEROSAL | 5.00 | MicroSource Discovery Systems Inc. | BRD-K61443650 | 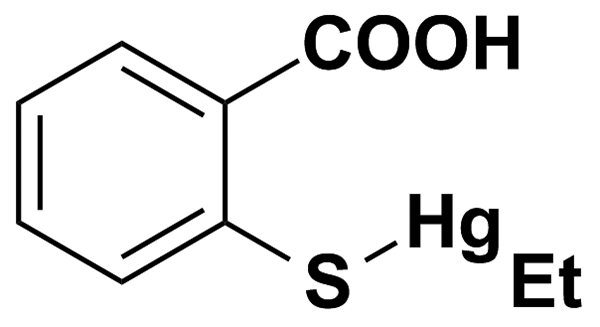 |
| TICLOPIDINE HYDROCHLORIDE | 3.33 | Prestwick Chemical Inc. | BRD-K00603606 | 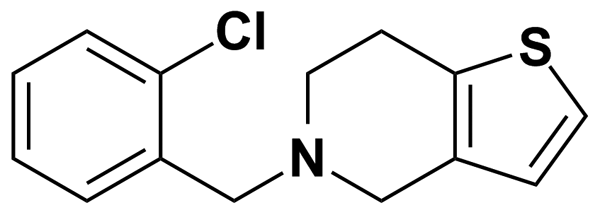 |
| TRIFLUOPERAZINE | 5.00 | Biomol International Inc. | BRD-K89732114 | 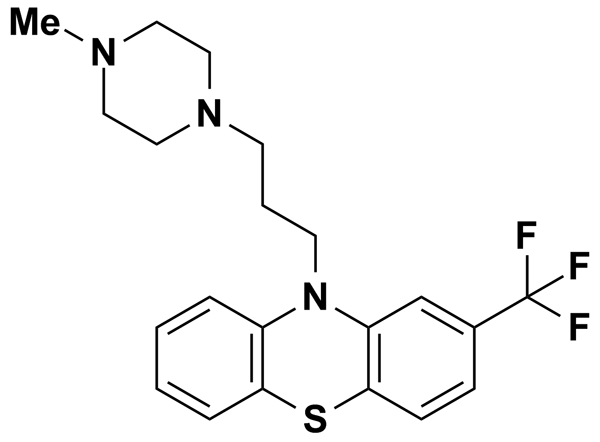 |
| TRIFLUOPERAZINE DIHYDROCHLORIDE | 2.08 | Prestwick Chemical Inc. | BRD-K89732114 | 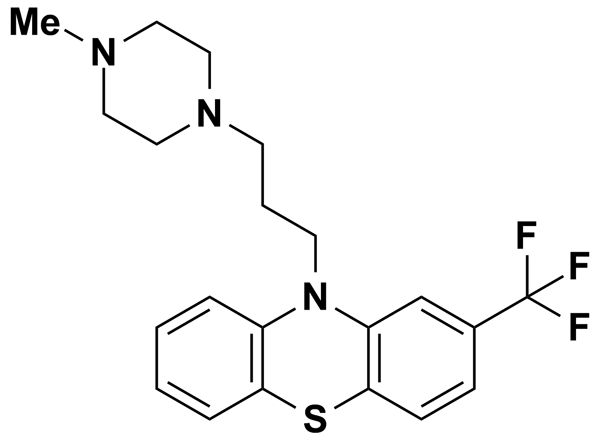 |
| TRIPTOLIDE | 6.94 | Biomol International Inc. | BRD-K39484304 | 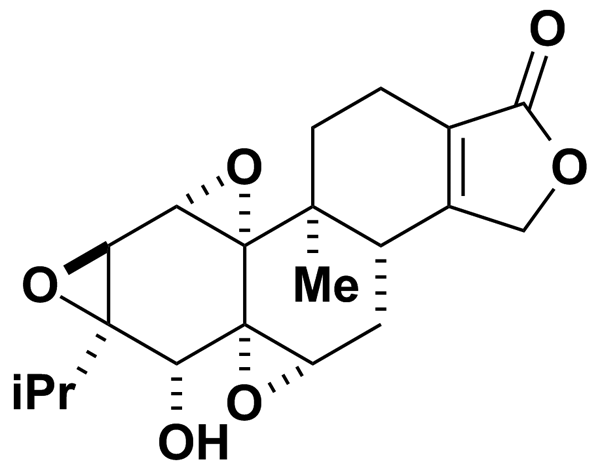 |
| U-0126 | 5.00 | Biomol International Inc. | BRD-K18787491 | 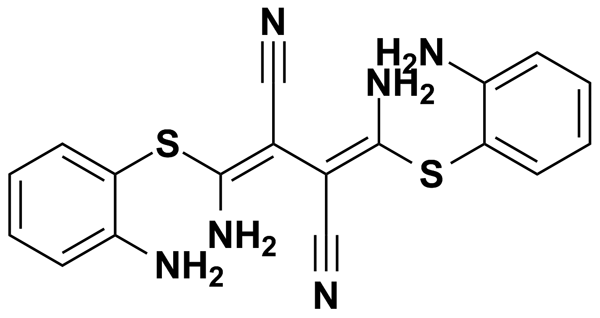 |
