# Supplementary material for: Multiplex Cytological Profiling Assay to Measure Diverse Cellular States
Source: PLoS One. 2013 Dec 2;8(12):e80999. doi: 10.1371/journal.pone.0080999 (PMC3847047; doi:10.1371/journal.pone.0080999)
Supplement: Table S8 — The clusters of compounds most highly enriched for annotation terms. (DOCX) [file pone.0080999.s016.docx]

**Table S8:** The clusters of compounds most highly enriched for annotation terms.

| Rank | Score | Compounds | Term occ. | Has term | Compound name | Broad ID |
| --- | --- | --- | --- | --- | --- | --- |
| 1 | 0.0002 | 5 | 5 of 11 | Yes | LANATOSIDE C | BRD-A64242993 |
|  |  |  |  | Yes | PERUVOSIDE | BRD-A57089740 |
|  |  |  |  | Yes | NERIIFOLIN | BRD-A31385885 |
|  |  |  |  | Yes | DIGITOXIN | BRD-A93236127 |
|  |  |  |  | Yes | DIGOXIN | BRD-A94756469 |
| 2 | 0.0025 | 3 | 3 of 11 | Yes | LANATOSIDE C | BRD-A64242993 |
|  |  |  |  | Yes | PERUVOSIDE | BRD-A57089740 |
|  |  |  |  | Yes | NERIIFOLIN | BRD-A31385885 |
| 3 | 0.0039 | 2 | 2 of 5 | Yes | Fenbendazole | BRD-K51318897 |
|  |  |  |  | Yes | OXIBENDAZOLE | BRD-K52075715 |
| 4 | 0.0078 | 2 | 2 of 7 | Yes | PENITREM A | BRD-K03842655 |
|  |  |  |  | Yes | PROPIOMAZINE MALEATE | BRD-A10471441 |
| 5 | 0.0104 | 3 | 2 of 5 |  | Ellipticine | BRD-K85985071 |
|  |  |  |  | Yes | Quinacrine dihydrochloride dihydrate | BRD-A45889380 |
|  |  |  |  | Yes | Hycanthone | BRD-K50406511 |
| 6 | 0.0126 | 3 | 2 of 5 | Yes | LYCORINE | BRD-A10335634 |
|  |  |  |  |  | GENTIAN VIOLET | BRD-K60025295 |
|  |  |  |  | Yes | KINETIN RIBOSIDE | BRD-K94325918 |
| 7 | 0.0132 | 3 | 2 of 5 |  | Paclitaxel | BRD-K62008436 |
|  |  |  |  | Yes | Fenbendazole | BRD-K51318897 |
|  |  |  |  | Yes | OXIBENDAZOLE | BRD-K52075715 |
| 8 | 0.0190 | 2 | 2 of 11 | Yes | DIGITOXIN | BRD-A93236127 |
|  |  |  |  | Yes | DIGOXIN | BRD-A94756469 |
| 9 | 0.0232 | 4 | 2 of 5 |  | Ellipticine | BRD-K85985071 |
|  |  |  |  | Yes | Quinacrine dihydrochloride dihydrate | BRD-A45889380 |
|  |  |  |  |  | resveratrol | BRD-K80738081 |
|  |  |  |  | Yes | Hycanthone | BRD-K50406511 |
| 10 | 0.0232 | 2 | 1 of 1 | Yes | MCI-186 | BRD-K35458079 |
|  |  |  |  |  | LAPACHOL | BRD-A06912736 |
| 11 | 0.0233 | 2 | 2 of 11 | Yes | LANATOSIDE C | BRD-A64242993 |
|  |  |  |  | Yes | NERIIFOLIN | BRD-A31385885 |
| 12 | 0.0239 | 2 | 1 of 1 |  | U-0126 | BRD-K18787491 |
|  |  |  |  | Yes | ESTRADIOL | BRD-K18910433 |
| 13 | 0.0239 | 2 | 1 of 1 |  | U-0126 | BRD-K18787491 |
|  |  |  |  | Yes | ESTRADIOL | BRD-K18910433 |
| 14 | 0.0241 | 3 | 2 of 7 |  | Fendiline hydrochloride | BRD-A71033472 |
|  |  |  |  | Yes | PENITREM A | BRD-K03842655 |
|  |  |  |  | Yes | PROPIOMAZINE MALEATE | BRD-A10471441 |
| 15 | 0.0242 | 2 | 1 of 1 |  | AG-879 | BRD-K59469039 |
|  |  |  |  | Yes | FCCP | BRD-K14821540 |
| 16 | 0.0243 | 2 | 1 of 1 |  | Cerulenin | BRD-K52075040 |
|  |  |  |  | Yes | (d,l)-Tetrahydroberberine | BRD-A69950438 |
| 17 | 0.0247 | 4 | 2 of 5 |  | Paclitaxel | BRD-K62008436 |
|  |  |  |  |  | Piperlongumine | BRD-K24132293 |
|  |  |  |  | Yes | Fenbendazole | BRD-K51318897 |
|  |  |  |  | Yes | OXIBENDAZOLE | BRD-K52075715 |
| 18 | 0.0247 | 3 | 2 of 7 |  | METOCLOPRAMIDE | BRD-K75641298 |
|  |  |  |  | Yes | Procaine hydrochloride | BRD-K24616672 |
|  |  |  |  | Yes | Fluphenazine dihydrochloride | BRD-K55127134 |
| 19 | 0.0251 | 2 | 1 of 1 | Yes | Ro 31-8220 | BRD-K06543683 |
|  |  |  |  |  | Colchicine | BRD-K00259736 |
| 20 | 0.0257 | 2 | 1 of 1 | Yes | U-0126 | BRD-K18787491 |
|  |  |  |  |  | ESTRADIOL | BRD-K18910433 |
| 21 | 0.0257 | 2 | 1 of 1 | Yes | U-0126 | BRD-K18787491 |
|  |  |  |  |  | ESTRADIOL | BRD-K18910433 |
| 22 | 0.0257 | 2 | 1 of 1 | Yes | U-0126 | BRD-K18787491 |
|  |  |  |  |  | ESTRADIOL | BRD-K18910433 |
| 23 | 0.0257 | 2 | 1 of 1 | Yes | U-0126 | BRD-K18787491 |
|  |  |  |  |  | ESTRADIOL | BRD-K18910433 |
| 24 | 0.0258 | 2 | 1 of 1 | Yes | GBR 12935 | BRD-K50135270 |
|  |  |  |  |  | Terfenadine | BRD-A06352418 |
| 25 | 0.0258 | 2 | 1 of 1 |  | GBR 12935 | BRD-K50135270 |
|  |  |  |  | Yes | Terfenadine | BRD-A06352418 |
| 26 | 0.0261 | 2 | 1 of 1 |  | Ro 31-8220 | BRD-K06543683 |
|  |  |  |  | Yes | Colchicine | BRD-K00259736 |
| 27 | 0.0261 | 2 | 1 of 1 |  | Ro 31-8220 | BRD-K06543683 |
|  |  |  |  | Yes | Colchicine | BRD-K00259736 |
| 28 | 0.0267 | 2 | 1 of 1 | Yes | Hydrochlorothiazide | BRD-K13078532 |
|  |  |  |  |  | Tacrine hydrochloride hydrate | BRD-K81473089 |
| 29 | 0.0267 | 2 | 1 of 1 | Yes | NSC-95397 | BRD-K68143200 |
|  |  |  |  |  | LY-83583 | BRD-K62792802 |
| 30 | 0.0267 | 2 | 1 of 1 | Yes | NSC-95397 | BRD-K68143200 |
|  |  |  |  |  | LY-83583 | BRD-K62792802 |
| 31 | 0.0267 | 2 | 1 of 1 | Yes | NSC-95397 | BRD-K68143200 |
|  |  |  |  |  | LY-83583 | BRD-K62792802 |
| 32 | 0.0267 | 2 | 1 of 1 | Yes | Hydrochlorothiazide | BRD-K13078532 |
|  |  |  |  |  | Tacrine hydrochloride hydrate | BRD-K81473089 |
| 33 | 0.0274 | 2 | 1 of 1 |  | METOCLOPRAMIDE | BRD-K75641298 |
|  |  |  |  | Yes | Fluphenazine dihydrochloride | BRD-K55127134 |
| 34 | 0.0280 | 2 | 1 of 1 | Yes | LYCORINE | BRD-A10335634 |
|  |  |  |  |  | GENTIAN VIOLET | BRD-K60025295 |
| 35 | 0.0280 | 2 | 1 of 1 | Yes | LYCORINE | BRD-A10335634 |
|  |  |  |  |  | GENTIAN VIOLET | BRD-K60025295 |
| 36 | 0.0282 | 2 | 1 of 1 | Yes | Cerulenin | BRD-K52075040 |
|  |  |  |  |  | (d,l)-Tetrahydroberberine | BRD-A69950438 |
| 37 | 0.0284 | 2 | 1 of 1 | Yes | NOVOBIOCIN SODIUM | BRD-K85307935 |
|  |  |  |  |  | NITRENDIPINE | BRD-A02006392 |
| 38 | 0.0301 | 2 | 2 of 13 | Yes | Fenbendazole | BRD-K51318897 |
|  |  |  |  | Yes | OXIBENDAZOLE | BRD-K52075715 |
| 39 | 0.0333 | 3 | 2 of 8 | Yes | LYCORINE | BRD-A10335634 |
|  |  |  |  |  | GENTIAN VIOLET | BRD-K60025295 |
|  |  |  |  | Yes | KINETIN RIBOSIDE | BRD-K94325918 |
| 40 | 0.0353 | 3 | 1 of 1 | Yes | Cerulenin | BRD-K52075040 |
|  |  |  |  |  | CYCLIZINE | BRD-K79501723 |
|  |  |  |  |  | (d,l)-Tetrahydroberberine | BRD-A69950438 |
| 41 | 0.0363 | 3 | 1 of 1 |  | PHENACEMIDE | BRD-K40905133 |
|  |  |  |  | Yes | NOVOBIOCIN SODIUM | BRD-K85307935 |
|  |  |  |  |  | NITRENDIPINE | BRD-A02006392 |
| 42 | 0.0366 | 3 | 1 of 1 |  | LYCORINE | BRD-A10335634 |
|  |  |  |  |  | GENTIAN VIOLET | BRD-K60025295 |
|  |  |  |  | Yes | KINETIN RIBOSIDE | BRD-K94325918 |
| 43 | 0.0366 | 3 | 1 of 1 |  | LYCORINE | BRD-A10335634 |
|  |  |  |  |  | GENTIAN VIOLET | BRD-K60025295 |
|  |  |  |  | Yes | KINETIN RIBOSIDE | BRD-K94325918 |
| 44 | 0.0366 | 3 | 1 of 1 |  | LYCORINE | BRD-A10335634 |
|  |  |  |  |  | GENTIAN VIOLET | BRD-K60025295 |
|  |  |  |  | Yes | KINETIN RIBOSIDE | BRD-K94325918 |
| 45 | 0.0366 | 3 | 1 of 1 |  | LYCORINE | BRD-A10335634 |
|  |  |  |  |  | GENTIAN VIOLET | BRD-K60025295 |
|  |  |  |  | Yes | KINETIN RIBOSIDE | BRD-K94325918 |
| 46 | 0.0373 | 5 | 2 of 5 |  | Ellipticine | BRD-K85985071 |
|  |  |  |  | Yes | Quinacrine dihydrochloride dihydrate | BRD-A45889380 |
|  |  |  |  |  | resveratrol | BRD-K80738081 |
|  |  |  |  |  | MELPHALAN | BRD-K87827419 |
|  |  |  |  | Yes | Hycanthone | BRD-K50406511 |
| 47 | 0.0374 | 3 | 1 of 1 |  | METOCLOPRAMIDE | BRD-K75641298 |
|  |  |  |  |  | Procaine hydrochloride | BRD-K24616672 |
|  |  |  |  | Yes | Fluphenazine dihydrochloride | BRD-K55127134 |
| 48 | 0.0383 | 3 | 1 of 1 |  | Cerulenin | BRD-K52075040 |
|  |  |  |  | Yes | CYCLIZINE | BRD-K79501723 |
|  |  |  |  |  | (d,l)-Tetrahydroberberine | BRD-A69950438 |
| 49 | 0.0383 | 3 | 1 of 1 |  | Cerulenin | BRD-K52075040 |
|  |  |  |  | Yes | CYCLIZINE | BRD-K79501723 |
|  |  |  |  |  | (d,l)-Tetrahydroberberine | BRD-A69950438 |
| 50 | 0.0393 | 3 | 1 of 1 |  | Cerulenin | BRD-K52075040 |
|  |  |  |  |  | CYCLIZINE | BRD-K79501723 |
|  |  |  |  | Yes | (d,l)-Tetrahydroberberine | BRD-A69950438 |
