# Supplementary material for: Multiplex Cytological Profiling Assay to Measure Diverse Cellular States
Source: PLoS One. 2013 Dec 2;8(12):e80999. doi: 10.1371/journal.pone.0080999 (PMC3847047; doi:10.1371/journal.pone.0080999)
Supplement: Text S1 — Data and software. (DOCX) [file pone.0080999.s017.docx]

Supplement to “Multiplex cytological profiling assay to measure diverse cellular states” by Sigrun M Gustafsdottir et al.

# Text S1: Data and software

We provide the complete set of images from our experiment as well as source code for computer programs that reproduce our results.

## Microscopy images

The microscopy images from our experiment have been deposited into the Broad Bioimage Benchmark Collection (<http://www.broadinstitute.org/bbbc/>), accession number BBBC022. [Ljosa V, Sokolnicki KL, Carpenter AE (2012) Annotated high-throughput microscopy image sets for validation. Nature Methods 9: 637]

## CellProfiler pipelines and illumination functions

There are two CellProfiler pipelines: one for calculating illumination correction functions, and one for applying the functions to the images and analyzing them (**Dataset S1**). They work with CellProfiler version 2.0.9925 and above. We also provide the illumination function computed by the first pipeline (**Dataset S2**).

## Image features

We provide the image features extracted by CellProfiler from the images so that the subsequent analyses can be reproduced without downloading the images and rerunning the image-analysis pipeline (**Dataset S3, http://www.broadinstitute.org/pubs/gustafsdottir_plosone_2013/**). The files are in CSV (comma-separated values) format, compressed with ZIP. The file supplement_Image.csv, which is 149 MB uncompressed, contains the per-image features of each of the 69,120 fields of view. Because the collection of per-cell features is so large (5.9 million cells, 68 GB uncompressed), it has been divided into 20 files, one per plate, with names of the form supplement_PLATEID.csv. Each file of per-cell features is between 3 and 4 GB uncompressed.

The fields (columns) are the standard output from CellProfiler’s ExportToDatabase module (see the CellProfiler documentation for details), so we describe them only briefly:

– per-object table: one row for each of the 5.9 million cells. The primary key (ImageNumber, ObjectNumber) identifies a cell. The column ImageNumber is a foreign key into the per-image table. The remaining 824 columns are the 805 measurements as well as 19 measurements that were not used for profiling (e.g., horizontal and vertical position of the cell in the field of view).

– per-image table: one row for each of the 69,120 fields of view. The primary key ImageNumber identifies an image (field of view). The remaining columns contain per-image metadata and whole-image features. The various metadata fields are described in the Broad Bioimage Benchmark Collection, where the image set has been submitted. (See the section *Microscopy images* above.)

## Data analysis software

We provide the source code to the programs that analyze the image features (**Dataset S4**). The zip archive expands to a folder called “inputs” that contains the compounds’ annotations and a folder called *code* that contains the source code.

The script *load_database.py* loads the image features into a MySQL database from the CSV files previously described. The file *Makefile* encodes all the analysis steps and how they depend on each other. Typing *make* in the *code* folder should recreate the results.

The programs require the following software and libraries:

- MySQL
- Python 2.6 or 2.7
- Matlab R2012b
- CellProfiler Analyst (<https://github.com/CellProfiler/CellProfiler-Analyst>) revision 1eedf363537113325f57409c53a190d9c5b9d52b
- python-docx (<http://github.com/mikemaccana/python-docx>) revision 647ee9753136b3a8a1ec1aa5f3cbe679d495bc6c for generating tables in Supporting Information
- lxml version 2.3.4
- progressbar version 2.3
- numpy version 1.5.1
- xlrd version 0.7.1
- scipy version 0.7.1

Unless otherwise noted, the python libraries are available through the Python Package Index (PyPi, http://pypi.python.org/pypi/).
