# Supplementary material for: Multiplex Cytological Profiling Assay to Measure Diverse Cellular States
Source: PLoS One. 2013 Dec 2;8(12):e80999. doi: 10.1371/journal.pone.0080999 (PMC3847047; doi:10.1371/journal.pone.0080999)
Supplement: Text S2 — Cytotoxicity. (DOC) [file pone.0080999.s018.doc]

Supplement to “Multiplex cytological profiling assay to measure diverse cellular states” by Sigrun M Gustafsdottir et al.

# Text S2: Cytotoxicity

On average, the imaged parts of the wells contain 766 cells. The standard deviation is 226. For each of the compounds that were both active and annotated, we counted the cells in each well and computed the median cell count across the compound’s four replicate wells. There were 14 compounds that had median cell counts below 88 (three standard deviations below the mean), which we used as a significance threshold for cytotoxicity: the five members of cluster C (lanatoside C, peruvoside, neriifolin, digitoxin, and digoxin) and the following compounds: Bay 11-7082, NSC-95397, anisomycin, fendiline hydrochloride, thimerosal, penitrem A, staurosporine, LY-83583, and teniposide.
